# Genetically predicted causal link between the plasma lipidome and pancreatic diseases: a bidirectional Mendelian randomization study

**DOI:** 10.3389/fnut.2024.1466509

**Published:** 2025-01-15

**Authors:** Liaoyi Lin, Yingbao Huang, Songzan Qian, Lifang Chen, Houzhang Sun

**Affiliations:** ^1^Department of Radiology, The First Affiliated Hospital of Wenzhou Medical University, Wenzhou, Zhejiang, China; ^2^Department of Intensive Care Unit, The First Affiliated Hospital of Wenzhou Medical University, Wenzhou, Zhejiang, China

**Keywords:** lipids, pancreatitis, pancreatic cancer, Mendelian randomization, causal relation

## Abstract

**Background:**

Recent studies have increasingly emphasized the strong correlation between the lipidome and the risk of pancreatic diseases. To determine causality, a Mendelian randomization (MR) analysis was performed to identify connections between the lipidome and pancreatic diseases.

**Methods:**

Statistics from a genome-wide association study of the plasma lipidome, which included a diverse array of 179 lipid species, were obtained from the GeneRISK cohort study with 7,174 participants. Genetic associations with four types of pancreatitis and pancreatic cancer were sourced from the R11 release of the FinnGen consortium. Two pancreatitis datasets from UK Biobank were employed as the validation cohort. MR analysis was conducted to assess the relationship between the genetically predicted plasma lipidome and these pancreatic diseases. Inverse variance weighted was adopted as the main statistical method. Bayesian weighted MR was employed for further verification. The MR-Egger intercept test for pleiotropy and Cochrane's Q statistics test for heterogeneity were performed to ensure the robustness.

**Results:**

MR analysis yielded significant evidence that 26, 25, 2, and 19 lipid species were correlated with diverse outcomes of pancreatitis, and 8 lipid species were correlated with pancreatic cancer. Notably, sterol ester (27:1/20:2) levels (OR: 0.84, 95% CI: 0.78–0.90, *P* = 5.79 × 10^−7^) were significantly associated with acute pancreatitis, and phosphatidylcholine (17:0_20:4) levels (OR: 0.89, 95% CI: 0.84–0.94, *P* = 1.78 × 10^−4^) and sterol ester (27:1/20:4) levels (OR: 0.90, 95% CI: 0.86–0.95, *P* = 2.71 × 10^−4^) levels were significantly associated with chronic pancreatitis after the Bonferroni-corrected test. As for validation, 14 and 9 lipid species were correlated with acute and chronic pancreatitis of UK Biobank. Some lipid classes showed significant effects both in the FinnGen consortium and UK Biobank datasets.

**Conclusions:**

The findings of this study indicate a potential genetic predisposition linking the plasma lipidome to pancreatic diseases and good prospects for future pancreatic disease clinical trials.

## Introduction

The prevalent gastrointestinal pancreatic illnesses in Europe include acute and chronic pancreatitis and pancreatic cancer (1). Pancreatitis is a complex, progressive inflammatory condition that affects the pancreas; its prevalence is increasing globally, and it is gradually becoming the main cause of hospitalization-related gastrointestinal diseases. Pancreatitis can cause abdominal pain, insufficient pancreatic function, and reduced quality of life (2–4). Acute pancreatitis (AP) and chronic pancreatitis (CP) are the major clinical diagnoses. Some people with pancreatitis, mainly due to excessive alcohol consumption, can be diagnosed with alcohol-induced AP (AAP) or CP (ACP) (5, 6). Among malignant tumors, pancreatic cancer has the poorest prognosis, and its survival rate is much worse than that of other cancers (7). A recent study indicated that the 5-year survival rate for all patients with malignant pancreatic tumors in the US between 2012 and 2018 was only 11.5% (8). The incidence of malignant pancreatic tumors will increase annually, and the incidence of this condition is increasing at a rate of 0.5% to 1.0% per year in the US (9). Therefore, it is important to explore the etiology of pancreatitis and pancreatic tumors to understand these diseases.

The role of lipid metabolism in the development of pancreatic disease has garnered increasing attention. Recent studies have highlighted the dysregulation of lipid metabolism in pancreatic disease and indicated that alterations in the blood lipidome may be associated with inflammation and tumor growth in the pancreas (10–12). Lipids play multiple roles in human metabolism, and lipidomics is a new tool for lipid analysis (13, 14). A previous study comparing malignant pancreatic tumor patients with normal controls revealed that the levels of lysophosphatidylcholine 22:0, phosphatidylcholine (P-14:0/22:2) and phosphatidylethanolamine are related to tumor stage (15); another study comparing malignant pancreatic tumor patients with normal controls revealed that the most dysregulated lipids in the cancer stage were sphingomyelin 41:1 and sulfatides/sulfated hexosyl ceramides 41:1 (OH) (12). However, past studies have been based on observational data, and these studies are vulnerable to challenges from residual confounding factors. In addition, reverse causality may be of concern in these studies because the underlying disease may influence certain biomarkers and behaviors. The effects of the plasma lipidome on pancreatic disease are still unclear.

Mendelian randomization (MR) analysis is increasingly being used to explore relationships between risk factors and disease outcomes by employing genetic variants as genetic instruments (16). Because genetic variations are randomly distributed at conception, MR analysis can emulate the conditions of a controlled randomized experiment to assess the impact of these instrumental variables (IVs) on specific diseases (17). The strength of MR lies in its reduced susceptibility to biases from confounding factors, reverse causation, and measurement errors, positioning it at a higher level of evidence than traditional observational studies, albeit not as conclusive as randomized controlled trials (18). To date, several studies using MR method to investigate the associations between various kinds factors and the risk of pancreatic diseases have been published. Mao et al. revealed causal associations between 18 dietary habits and pancreatitis (19). Some scholars have investigated the causal effects of 30 genetically relevant potential risk factors on the risk of pancreatitis (20). Wang et al. elucidated the causal connection between the gut microbiota and the risk of pancreatitis (21). Zhong et al. (22) reported that genetically elevated blood metabolites had potential causal effects on pancreatic cancer risk. These studies revealed important influencing factors for pancreatic diseases through MR methods and demonstrated the practicability and feasibility of MR methods.

Currently, some researchers have conducted a lipidomic study of 179 lipid species via univariate and multivariate genome-wide analyses (23). The summary data from this study facilitate an MR analytical approach to examine the effects of the plasma lipidome on pancreatitis and pancreatic cancer. This study aimed to determine the causal effects of the plasma lipidome on the risk of developing pancreatic disease using a two-sample MR framework, and the Bayesian weighted MR (BWMR) was applied to further verify the results. Additionally, reverse MR analysis was carried out to assess whether pancreatic diseases could exert any causal effects on the plasma lipidome.

## Materials and methods

### Study design

**Figure 1:**
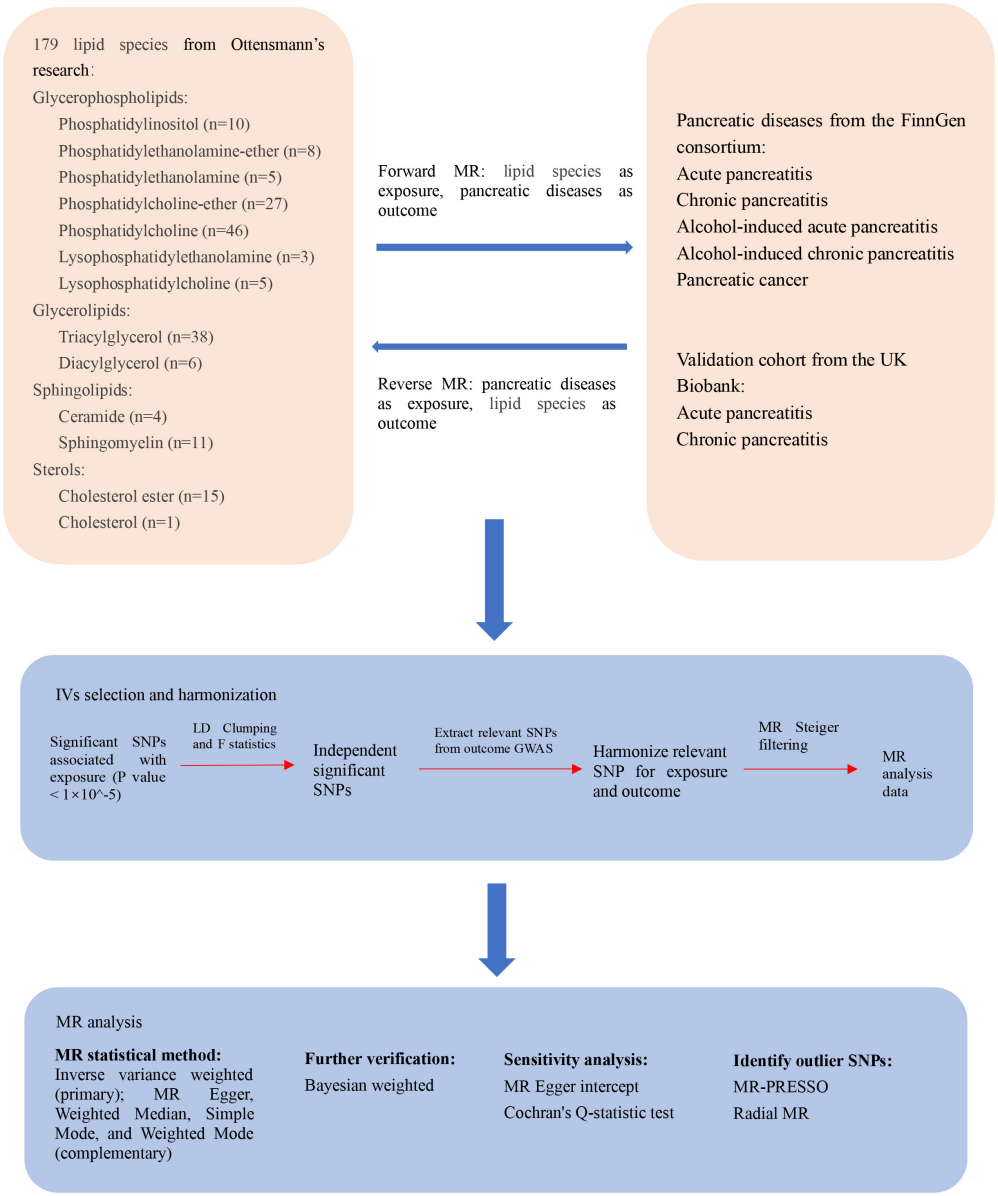
The flowchart of the study design of the bidirectional Mendelian randomization analysis between the plasma lipidome and pancreatic diseases. MR, Mendelian randomization; IV, instrumental variable; SNP, single nucleotide polymorphism; LD, linkage disequilibrium; GWAS, genome-wide association studies; MR-PRESSO, Mendelian randomization pleiotropy residual sum and outlier; Radial MR, Radial regression of MR.

An MR study was conducted to determine the relationship between the plasma lipidome and pancreatic diseases. To ensure the reliability of our results, MR analysis is based on three critical assumptions: IVs must be associated with the risk factors; IVs must not be related to any confounding variables that could affect the diseases under study; and IVs must influence the outcomes solely through their association with the risk factors (24). Additional ethics approval or informed consent was not required due to the characteristics of public database research, and the data used in this study were public, anonymized, and deidentified (25). The flowchart of this bidirectional MR study design is presented in Figure 1.

### Data sources

#### GWAS data for lipidome

Single nucleotide polymorphisms (SNPs) of the human plasma lipidome were identified as IVs for the MR study. Ottensmann et al. (23) conducted a comprehensive lipidomic analysis by the shotgun method on the GeneRISK cohort with 179 lipid species. The lipid classes were detected included cholesterol ester (CE), cholesterol (Chol), ceramide (Cer), sphingomyelin (SM), lysophosphatidylcholine (LPC), lysophosphatidylethanolamine (LPE), phosphatidylinositol (PI), phosphatidylcholine (PC), phosphatidylcholine-ether (PCO), phosphatidylethanolamine (PE), phosphatidylethanolamine-ether (PEO), diacylglycerol (DAG), and triacylglycerol (TAG), as detailed in Supplementary Table S1. Data on genome-wide association studies (GWAS) summary statistics for the plasma lipidome are accessible online in the GWAS catalog (https://www.ebi.ac.uk/gwas/) with the study accession numbers GCST90277238 to GCST90277416 (23).

#### GWAS data for pancreatic diseases

Summary GWAS statistics for pancreatitis and pancreatic cancer outcomes were obtained from the FinnGen consortium. The R11 release of the FinnGen research program (https://r11.finngen.fi/), which draws on genetic data from more than 500,000 individuals associated with the Finnish Biobank, was utilized for this analysis. This dataset comprises AP with 7,562 cases and 397,583 controls, CP with 4,222 cases and 397,583 controls, AAP with 1,144 cases and 452,589 controls, ACP with 2,158 cases and 451,575 controls, and pancreatic cancer with 1,992 cases and 345,118 controls. The genetic linkages were carefully adjusted to consider various factors, such as sex, age, genetic background, and variations of genotyping batches. This meticulous approach ensures the reliability and validity of findings from genetic linkages within this extensive dataset.

#### GWAS data for validation cohort

Although the main results are dominated by the FinnGen consortium R11 dataset, we did a replication study by using the AP and CP of UK Biobank datasets for validation, for a more comprehensive understanding of the differences in MR analysis between different data. Summary GWAS statistics for validation cohort of AP and CP were obtained from the UK Biobank GWAS round 2 (https://www.nealelab.is/uk-biobank). UK Biobank was approved by the North West Multi-Center Research Ethics Committee in Scotland and was approved by the Community Health Index Advisory Group. This dataset comprises AP with 1,292 cases and 359,902 controls, CP with 246 cases and 360,948 controls.

### Instrumental variable selection

To enhance the credibility and accuracy of conclusions about the relationship between the plasma lipidome and pancreatic diseases, we implemented several quality control measures. We used a more inclusive threshold of *P* < 1 × 10^−5^ at the genome-wide significance level to secure a greater number of SNPs rather than a stricter threshold of *P* < 5 × 10^−8^ (21). This threshold allows for a sufficient set of SNPs to be considered. Additionally, to address the effects of linkage disequilibrium, SNPs were chosen with a *r*^2^ of < 0.001 and at least spaced within a genomic window of 10,000 kilobases, ensuring minimal overlap and independence among genetic variants. These measures collectively aim to solidify the reliability of our findings by using appropriately strong and independent IVs. Generally, SNPs with *F*-statistic values > 10 cannot be considered weak IVs and are recommended for subsequent MR analyses. F-statistics utilizing the subsequent equation: *F* = beta^2^/se^2^ (26). Palindromic SNPs and SNPs absent from outcome data and with incomplete information were removed from the IVs during the harmonization process. MR Steiger filtering was also applied to test whether the IVs were more strongly associated with outcomes than with exposure, and the SNPs that did not pass the test were excluded to avoid reverse causality (27).

### Analysis for MR

We evaluated the relationships between 179 lipid species and various forms of pancreatic disease, including AP, CP, AAP, ACP, and pancreatic cancer. To ensure the robustness of our findings, we utilized five popular MR methods: inverse variance weighting (IVW), weighted median, simple modal, MR–Egger regression, and weighted modal methods. Our main method was IVW, and the other four methods were used as supplemental methods. The IVW relies on an asymptotic estimate of the standard error of the causal ratio estimate for each variable (28), while the weighted median model can provide an unbiased estimate even if there are many unqualified IVs (29). MR–Egger regression can provide a valid test for the null causality hypothesis and a valid causality estimate even if genetic variants are invalid (30). When the set of maximally similar causal effect estimates from valid instruments, the weighted modal model result is consistent regardless of most of the instruments are invalid (31).

Heterogeneity was assessed using Cochrane's Q statistic, and there was no significant heterogeneity if the *P* value exceeded 0.05 (28). Horizontal pleiotropy was assessed using the MR–Egger intercept test, and there was no presence of horizontal pleiotropy if the *P* value exceeded 0.05 (30). If pleiotropy existed, the MR Pleiotropy Residual Sum and Outlier (MR-PRESSO) method and radial regression of MR (Radial MR) were performed to identify abnormal SNPs as outliers, and then we removed these outliers to obtain valid causal effect estimates (32, 33).

Furthermore, the BWMR was employed to further verify the causal effects of exposure and outcome. The advantage of this approach is to reduce potential bias because it can solve the strong horizontal pleiotropy problem by a weighting scheme. This method also takes into account the uncertainty of weak effects due to polygenicity, further enhancing the robustness of causal inference (34).

### Reverse MR analysis

Reverse MR was utilized to determine whether pancreatic disease has any causal effect on the plasma lipidome using SNPs associated with pancreatic diseases; in other words, this statistic included pancreatic diseases as exposures and 179 lipid species as outcomes. We still used a threshold of *P* < 1 × 10^−5^ at the genome-wide significance level for selecting instrumental variables for pancreatic disease. And the SNPs with a considerable distance in genomic window (≥10,000 kilobases) and less probability of linkage disequilibrium (*r*^2^ < 0.001) were included. The *F*-statistic and MR Steiger filtering was also applied in IVs selection of reverse MR analysis as mentioned above.

### Validation analysis for AP and CP

We used a same standard for selecting instrumental variables for 179 lipid species as mentioned above. We adopted a *P* < 1 × 10^−5^ in selecting SNPs, and we employed a linkage disequilibrium threshold where *r*^2^ < 0.001, coupled with clumping distances ≥10,000 kilobases. The F-statistic and MR Steiger filtering was also applied in IVs selection. Then the harmonization process was performed and the two-sample MR was proceeded subsequently. The AP and CP of UK Biobank datasets were used for MR analysis as outcomes. Reverse MR analysis of UK Biobank datasets was done according to the same standards for IVs selection with a *P* < 1 × 10^−5^, *r*^2^ < 0.001 and clumping distances ≥10,000 kilobases, this statistic included the UK Biobank datasets as exposures and 179 lipid species as outcomes.

### Statistical analyses

All research used R Software version 4.3.2 for statistical analysis, with the R packages “TwoSampleMR,” “psych::r.test,” “MR-PRESSO,” and “RadialMR.” The results from IVW and the accompanying BWMR tests were visualized by forest plots. The scatter plots, funnel plots, leave-one-out plots and MR effect size plots were generated for visual inspection. Data visualization was carried out using the R packages “ggplot2” and “forestploter.” To address multiple hypothesis testing, we applied Bonferroni correction to the IVW results, setting the significance level at *P* < 2.79 × 10^−4^ (0.05/179). *P* values between 2.79 × 10^−4^ and 0.05 were considered suggestive causal associations. This method helps ensure robust control of false positives in our analysis.

## Results

### Overview

A total of 179 lipid species were included in the analyses as potential risk factors. After the genome-wide significance threshold and linkage disequilibrium test screening steps, 4,506 eligible SNPs were included for subsequent MR analysis. The *F*-statistic of all included SNPs >10 ranged from 19.54 to 1946.15, indicating that there were no weak IVs in our study (Supplementary Table S2). Then the two-sample MR was performed after the harmonization process and Steiger filtering.

### Causative effects of the plasma lipidome on pancreatic diseases

#### Plasma lipidome and AP

**Figure 2:**
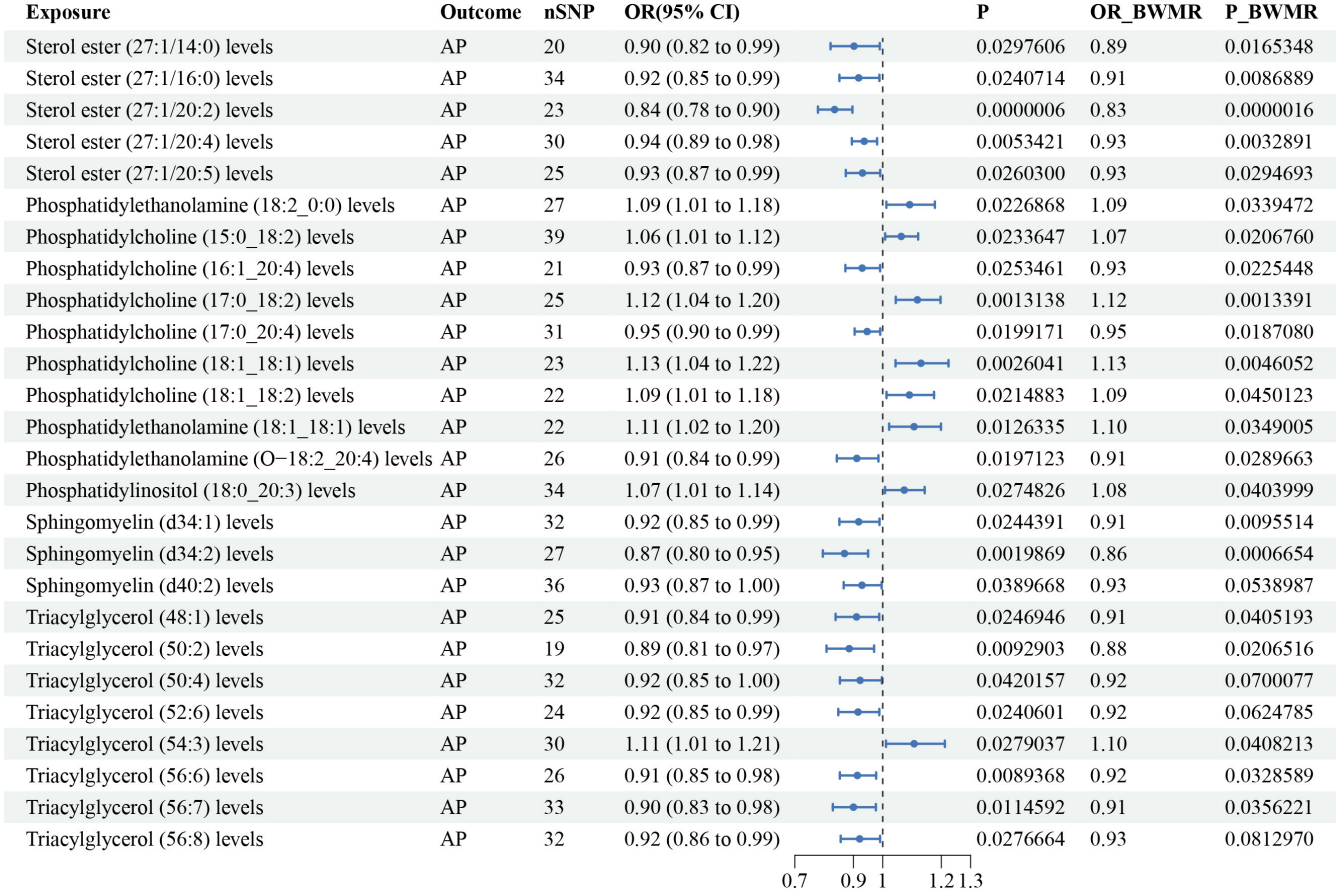
Forest plot to visualize the causal effect of plasma lipidome on AP using the inverse variance weighted method. The accompanying BWMR tests are shown together. AP, acute pancreatitis; SNP, single nucleotide polymorphisms; OR, odds ratio; CI, confidence interval; BWMR, Bayesian weighted Mendelian randomization.

According to the IVW method, 26 lipid species were correlated with AP risk (*P* < 0.05), and the lipid classes included CE, LPE, PC, PE, PEO, PI, SM, and TAG (Supplementary Table S3). Eight lipid species showed an increased risk of AP, while 18 lipid species were linked to a reduced risk. However, we found that sphingomyelin (d40:2) (*P-BWMR* = 0.054), triacylglycerol (50:4) (*P-BWMR* = 0.07), triacylglycerol (52:6) (*P-BWMR* = 0.062) and triacylglycerol (56:8) (*P-BWMR* = 0.081) did not pass further BWMR verification. Possible pleiotropy was observed for sterol ester (27:1/20:2) and AP, MR-PRESSO analysis and Radical MR analyses were conducted to remove outliers. Finally, the correlation remained significant for the corrected result (OR: 0.84, 95% CI: 0.78–0.90, *P* = 5.79 × 10^−7^; Supplementary Table S4). Bonferroni correction (*P* < 2.79 × 10^−4^) for this corrected result indicated that sterol ester (27:1/20:2) were notably linked to a reduced risk of AP. The estimate of the BWMR analysis showed a consistent significant result for sterol ester (27:1/20:20) (OR: 0.83, 95% CI:0.77–0.90, *P-BWMR* = 1.59 × 10^−6^). Some heterogeneities were observed for sterol ester (27:1/16:0), sterol ester (27:1/20:2), phosphatidylethanolamine (18:2_0:0), phosphatidylethanolamine (18:1_18:1), phosphatidylinositol (18:0_20:3), sphingomyelin (d34:2), triacylglycerol (54:3) and triacylglycerol (56:7) with AP (Supplementary Table S3). The corrected result of sterol ester (27:1/20:2) showed no heterogeneity with a *P* value > 0.05 according to Cochran's Q tests (Supplementary Table S4). The results from IVW are our primary criterion, and the accompanying BWMR tests are shown together in the forest plot (Figure 2). The data visualization of the leave-one-out analysis, MR effect size, scatter plot and funnel plot are shown in Supplementary Figures S1–S27.

#### Plasma lipidome and CP

**Figure 3:**
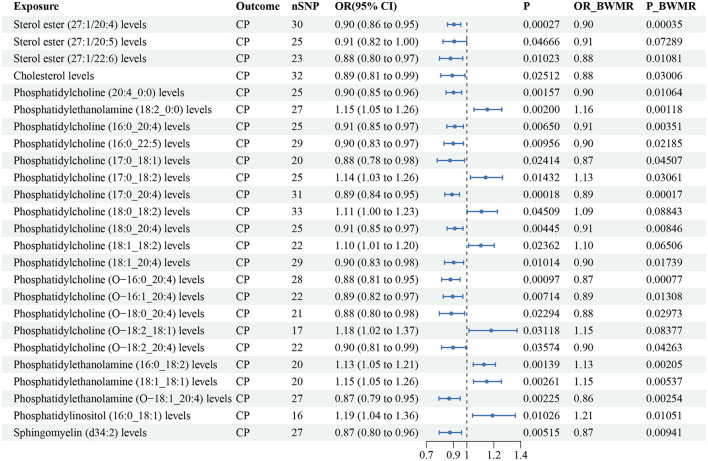
Forest plot to visualize the causal effect of plasma lipidome on CP using the inverse variance weighted method. The accompanying BWMR tests are shown together. CP, chronic pancreatitis; SNP, single nucleotide polymorphisms; OR, odds ratio; CI, confidence interval; BWMR, Bayesian weighted Mendelian randomization.

According to the IVW method, 25 lipid species were correlated with CP risk (*P* < 0.05), and the lipid classes included CE, Chol, LPC, LPE, PC, PCO, PE, PEO, PI and SM (Supplementary Table S5). Eight lipid species exhibited an increased risk of CP, while 17 lipid species were linked to a reduced risk. Phosphatidylcholine (17:0_20:4) (OR: 0.89, 95% CI: 0.84–0.94, *P* = 1.78 × 10^−4^) and sterol ester (27:1/20:4) (OR: 0.90, 95% CI:0.86–0.95, *P* = 2.71 × 10^−4^) decreased the risk of CP which passing the Bonferroni correction. The estimate of the BWMR analysis of sterol ester (27:1/20:5), phosphatidylcholine (18:0_18:2), phosphatidylcholine (18:1_18:2) and phosphatidylcholine (O-18:2_18:1) had *P* values > 0.05. A pleiotropy was observed for phosphatidylethanolamine (18:1_18:1), but the relationship remained significant after correction (OR: 1.15, 95% CI: 1.05–1.26; *P* = 0.003). The estimate of the BWMR analysis showed a consistent significant result (OR: 1.15, 95% CI: 1.04–1.26, *P-BWMR* = 0.005; Supplementary Table S4). Similar adjustments for pleiotropy confirmed a remained significant correlation between phosphatidylinositol (16:0_18:1) and increased CP (OR: 1.19, 95% CI: 1.04–1.36, *P* = 0.01), and similar results were obtained from the BWMR analysis (OR: 1.21, 95% CI: 1.04–1.39, *P-BWMR* = 0.01; Supplementary Table S4). Some heterogeneities were noted in the effects of phosphatidylcholine (18:1_20:4), sterol ester (27:1/20:5), phosphatidylcholine (18:0_18:2) and phosphatidylinositol (16:0_18:1) on CP (Supplementary Table S5). The corrected result for phosphatidylinositol (16:0_18:1) showed no heterogeneity (Supplementary Table S4). The results from IVW are our primary criterion, and the accompanying BWMR tests are shown together in the forest plot (Figure 3). The data visualization of the leave-one-out analysis, MR effect size, scatter plot and funnel plot are shown in Supplementary Figures S28–S54.

#### Plasma lipidome and AAP

**Figure 4:**
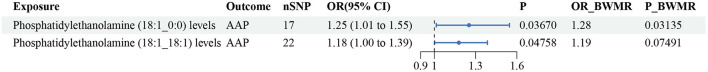
Forest plot to visualize the causal effect of plasma lipidome on AAP using the inverse variance weighted method. The accompanying BWMR tests are shown together. AAP, alcohol-induced acute pancreatitis; SNP, single nucleotide polymorphisms; OR, odds ratio; CI, confidence interval; BWMR, Bayesian weighted Mendelian randomization.

According to the IVW method, 2 lipid species, phosphatidylethanolamine (18:1_0:0) (OR: 1.25, 95% CI: 1.01–1.55; *P* = 0.037) and phosphatidylethanolamine (18:1_18:1) (OR: 1.18, 95% CI: 1.00–1.39; *P* = 0.048), were correlated with an increased risk of AAP (Supplementary Table S6). As for the BWMR analysis, there was a genetic association between the phosphatidylethanolamine (18:1_0:0) and an increased risk of CP (OR = 1.28; 95% CI: 1.02–1.61; *P-BWMR* = 0.031), but phosphatidylethanolamine (18:1_18:1) did not pass the BWMR verification (*P-BWMR* = 0.075). Substantial heterogeneities or pleiotropies were not observed for these two lipids on AAP (Supplementary Table S6). The results from IVW are our primary criterion, and the accompanying BWMR tests are shown together in the forest plot (Figure 4). The data visualization of the leave-one-out analysis, MR effect size, scatter plot and funnel plot are shown in Supplementary Figures S55, S56.

#### Plasma lipidome and ACP

**Figure 5:**
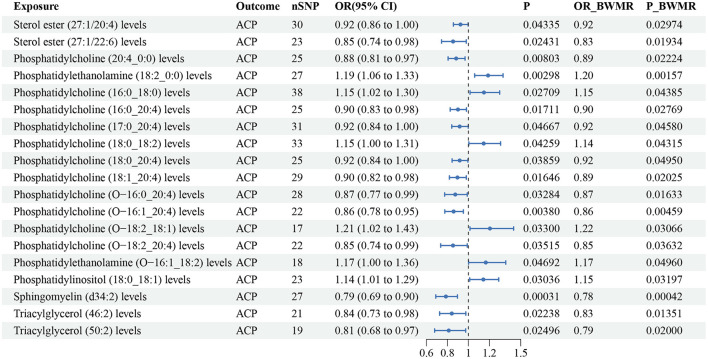
Forest plot to visualize the causal effect of plasma lipidome on ACP using the inverse variance weighted method. The accompanying BWMR tests are shown together. ACP, alcohol-induced chronic pancreatitis; SNP, single nucleotide polymorphisms; OR, odds ratio; CI, confidence interval; BWMR, Bayesian weighted Mendelian randomization.

According to the IVW method, 20 lipid species were correlated with ACP risk (*P* < 0.05), and the lipid classes included CE, LPC, LPE, PC, PCO, PEO, PI, SM, and TAG (Supplementary Table S7). Six lipid species were linked to an increased risk of ACP, while 14 lipid species were linked to a reduced risk. In addition, these lipids all passed the BWMR verification with a *P* value < 0.05. Notably, sterol ester (27:1/16:0) showed potential pleiotropy with ACP, the possible pleiotropy remained significant after correction, and the cause effect of sterol ester (27:1/16:0) for ACP was lost on the basis of the IVW result (OR: 0.91, 95% CI: 0.81–1.01, *P* = 0.078; *P*_intercept_ = 0.048; Supplementary Table S4). We decided to eliminate the correlation result of sterol ester (27:1/16:0) due to pleiotropy and the corrected IVW result; ultimately, 19 lipid species were correlated with ACP risk. Cochran's Q tests showed no evidence of heterogeneity. The results from IVW are our primary criterion, and the accompanying BWMR tests are shown together in the forest plot (Figure 5). The data visualization of the leave-one-out analysis, MR effect size, scatter plot and funnel plot are shown in Supplementary Figures S57–S77.

#### Plasma lipidome and pancreatic cancer

**Figure 6:**
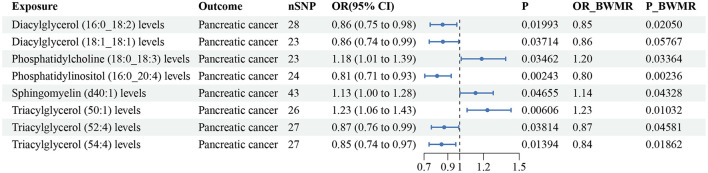
Forest plot to visualize the causal effect of plasma lipidome on pancreatic cancer using the inverse variance weighted method. The accompanying BWMR tests are shown together. SNP, single nucleotide polymorphisms; OR, odds ratio; CI, confidence interval; BWMR, Bayesian weighted Mendelian randomization.

According to the IVW method, eight lipid species were related to pancreatic cancer risk (*P* < 0.05), and the lipid classes included DAG, PC, PI, SM, and TAG (Supplementary Table S8). Three lipid species increased the risk of pancreatic cancer, while five lipid species reduced the risk. Diacylglycerol (18:1_18:1) (*P-BWMR* = 0.058) did not pass further BWMR verification. The MR–Egger intercept tests showed no notable pleiotropy for these eight lipid species, and Cochran's *Q* tests detected heterogeneity only for Sphingomyelin (d40:1) (Supplementary Table S8). The results from IVW are our primary criterion, and the accompanying BWMR tests are shown together in the forest plot (Figure 6). The data visualization of the leave-one-out analysis, MR effect size, scatter plot and funnel plot are shown in Supplementary Figures S78–S85.

### Reverse MR analysis

After filtering the IVs in the same way, 37 AP associated SNPs, 40 CP associated SNPs, 26 AAP associated SNPs, 35 ACP associated SNPs and 27 pancreatic cancer associated SNPs were included in the reverse MR analysis as eligible IVs, all *F*-statistic >10 ranged from 19.58 to 158.98 (Supplementary Table S9). After the harmonization process and Steiger filtering with 179 lipid species, we performed two-sample MR for pancreatic diseases and 179 lipid species. By the IVW method, we identified there are no potential causal associations between pancreatic disease and the plasma lipidome (all *P* > 0.05). Furthermore, according to the BWMR, pancreatic disease still had no causal relationship with the plasma lipidome (all *P* > 0.05). For sensitivity analysis, the absence of substantial heterogeneity or pleiotropy was observed in the entire reverse MR analysis, and all MR–Egger intercept tests and Cochran's *Q* tests yielded a *P* value > 0.05.

### Validation analysis for AP and CP

The IVs for 179 lipid species was same as mentioned above after the genome-wide significance threshold and linkage disequilibrium test screening steps (Supplementary Table S2). According to the IVW method, 14 lipid species were correlated with AP risk (*P* < 0.05), and the lipid classes included CE, DAG, PC, PI, SM, and TAG. Triacylglycerol (48:0) (*P-BWMR* = 0.054) did not pass further BWMR verification. A pleiotropy was observed for Triacylglycerol (52:5) for AP, but the relationship remained significant after correction (OR: 0.999, 95% CI: 0.998–0.999; *P* = 8.62 × 10^−5^). Cochran's *Q* tests showed no evidence of heterogeneity for AP. According to the IVW method, 10 lipid species were correlated with CP risk (*P* < 0.05), and the lipid classes included CE, Cer, PC, PCO, PI, SM. Ceramide (d40:2) (*P-BWMR* = 0.062) did not pass further BWMR verification. A pleiotropy and a heterogeneity were observed for Sphingomyelin (d40:2) for CP, the possible pleiotropy remained significant after correction (*P*_intercept_ = 0.008), but the heterogeneity was removed. We decided to eliminate the correlation result of Sphingomyelin (d40:2) for CP due to pleiotropy; ultimately, nine lipid species were correlated with CP risk. The detail data for AP and CP of UK Biobank are shown in Supplementary Table S10 and Supplementary Figures S86, S87. The data visualization of the leave-one-out analysis, MR effect size, scatter plot and funnel plot are shown in Supplementary Figures S88–S113. After filtering the IVs by the genome-wide significance threshold and linkage disequilibrium test, 38 AP associated SNPs and 80 CP associated SNPs were included in the reverse MR analysis as eligible IVs for UK Biobank, all *F*-statistic >10 ranged from 19.52 to 33.01 (Supplementary Table S11). After the harmonization process and Steiger filtering with 179 lipid species, we performed two-sample MR for pancreatitis and 179 lipid species. By the IVW method, we identified there are no potential causal associations between pancreatitis and the plasma lipidome (all *P* > 0.05). No heterogeneity or pleiotropy was observed in the reverse MR analysis for AP and CP of UK Biobank.

## Discussion

This study is the first extensive and profound analysis of the relationship between the plasma lipidome and pancreatic diseases at a genetic prediction scale. Previous investigations of lipids and pancreatic diseases have focused primarily on conventional lipids, such as high-density lipoprotein cholesterol (35, 36), low-density lipoprotein cholesterol (37), triglycerides (38), and total cholesterol (39). Our study builds upon lipid research by utilizing lipidomics, providing a more comprehensive understanding of the variability in circulating lipids. Specifically, our analysis encompassed four major lipid categories based on Ottensmann et al.'s (23) lipidomic GWAS data. Notably, we identified significant associations between sterol ester (27:1/20:2) levels and a reduced risk of AP, and between phosphatidylcholine (17:0_20:4) and sterol ester (27:1/20:4) levels and a reduced risk of CP, highlighting their potential role in mitigating pancreatic diseases. It cannot be ignored that other lipid species presented in result part of our research also have a suggestive causal association with pancreatic diseases.

A potential correlation was identified between the specific lipid species and the incidence of AP and CP in our study, and it was proven that lipid metabolism plays a role in pancreatitis. This research reported that some sterol esters are stable protective factors for AP and CP. Notably, sterol ester (27:1/20:2) and sterol ester (27:1/20:4) are critical factors for AP and CP according to the Bonferroni correction. A study reported the changes in lipids in acute pancreatitis patients, the result displayed that cholesterol ester (20:1) and cholesterol ester (18:2) levels were significantly lower in acute pancreatitis patients than in normal controls (10). Sterol lipids may alleviate inflammation and tissue damage considering the critical function of these lipids in maintaining membrane integrity and modulating immune responses (40). By passing the Bonferroni correction, phosphatidylcholine (17:0_20:4) was shown to have a significant protective effect on CP. PC is an important membrane constituent and accounts for approximately half of all membrane lipids in eukaryotic cells, it plays an important part in signaling and immune regulation (41). A study compared the composition of plasma PCs in CP patients with that in controls, and found that PC was significantly and markedly altered in CP patients (42). Although the composition of PC in their study differed from that in our study, the effect of PC on the risk of CP was prominent in both studies, and our study revealed a genetically predicted causal relationship between phosphatidylcholine (17:0_20:4) and the risk of CP. The results of our study provide a more comprehensive and abundant lipid species than most previous studies, and we also found many highly valuable liposomes that were associated with AP and CP. Some sphingolipids were found to be stable protective factors for AP and CP, and certain glycerophospholipid and TAG levels were found to affect pancreatic inflammation. In a prospective study, serum triglycerides were found to be linked to an increased risk of AP, with a hazard ratio of 1.21 (43). This finding revealed a role for triglycerides in AP, but it only reflects the effect of serum total triglyceride levels on AP; research on triglyceride subtypes is lacking. Other studies have shown that lipid metabolism potentially plays a role in modulating the progression of pancreatitis or tissue inflammation through glycerophospholipid (44), triacylglycerol (20, 45), and sphingomyelin (46) levels. Some of the findings are similar to ours, but we present results at the genetic level through MR analysis, and the subtypes of lipids were further analyzed in order to better understand their role. The double MR analysis reflected the effect of liposome on pancreatitis more profoundly, our additional validation using UK Biobank datasets coordinating with the results of FinnGen consortium shows that CE, PC, PI, SM, and TAG are some relatively stable influencing factors for AP, and CE, PC, PCO, PI, and SM are some relatively stable influencing factors for CP.

Alcohol exposure is known to contribute to the onset and progression of pancreatitis, and the risks of AAP and ACP also need attention. Sterol esters and SMs were still protective factors for ACP like the results of AP and CP, and certain glycerophospholipids and TAGs were also associated with AAP and ACP. Alcohol intake has complex effects on lipid metabolism, for example, high alcohol intake may elevate plasma triglycerides, and alcohol-associated hypertriglyceridemia can aggravate the risk of pancreatitis, but plasma triglycerides may decrease with light to moderate alcohol consumption (47). A previous study revealed that patients with ACP had significant changes in plasma PL, TAG and sterol esters compared with healthy controls (48), and the effects of different lipid subtypes may help us to better understand the role of lipids in alcoholic pancreatitis. In our study, we were able to identify the role of each lipid by further classifying them, and the use of lipidomics can reveal subtype information that traditional lipid studies have not been able to obtain. By traditional lipid metabolism MR analysis, Mao et al. (20) found that triglycerides (OR = 1.367, 95% CI: 1.075–1.737) are a risk factor for ACP; however, they did not find other associations between lipid metabolism and alcohol-induced pancreatitis, possibly because they were studying conventional lipid elements only.

Previous studies exploring the connection between lipid metabolites and pancreatic cancer have been limited due to the frequent use of postdiagnosis blood samples and inconsistent findings (15, 49, 50). We conducted a full-scale lipidomic analysis to identify lipid species linked to the risk of malignant pancreatic tumors. Some lipid classes, such as SM, PC, PI, DAG, and TAG, were associated with pancreatic cancer in our study. The dysregulation of lipid metabolism in tumor cells suggests that changes in lipid groups may affect tumor growth, and the significant differences between pancreatic cancer patients and healthy controls are the basic research method of traditional observational research in the past (12). Zhou et al. (49) studied 1,206 plasma lipids of molecules harvested from 20 patients and identified 88 lipids existing differences that contained PC, PI, TAG and PE lipid classes. Lipidomic analysis of prediagnostic serum samples has revealed lipids associated with the risk of ductal carcinoma of the pancreas (51), and researchers have found that 43 lipid species from eight lipid classes are linked to malignant tumors. Notably, some LPC, PE, PC, CE, DAG and TAG lipid classes showed the strong associations with pancreatic cancer. Our study used a much larger sample of pancreatic cancer patients (1,992 cases and 345,118 controls), MR was also used to exclude confounding factors from observational studies, and it is helpful to refine the results on the relationship between lipids and pancreatic cancer. However, LPC, CE and PE were not linked to pancreatic cancer in our study, and this difference may be due to differences in patient cohorts and study methods.

Our study focuses on the association between the lipidome and pancreatic diseases, by identifying specific lipid types and even subtypes, we have a more accurate regulatory target for controlling the occurrence and development of pancreatic diseases in clinical practice. It is helpful to early predict and intervene the pancreatic diseases by monitoring the abnormality of liposome metabolism. Regulating lipid metabolism through drugs or diet may be effective in controlling the occurrence of pancreatic diseases in the future. The use of drug assistance to appropriately reduce harmful lipids and increase beneficial lipids is a promising approach for the management of patients with pancreatic diseases. Otherwise, the lipid species may influence specific biological pathways associated with pancreatic diseases. Other studies have revealed that nuclear receptor subfamily 5 group A member 2 (NR5A2) haploinsufficiency has been seen associated with chronic pancreatitis and pancreatic cancer (52, 53). Nuclear receptors can act as regulatory molecules, interact with specific DNA sequences, and mediate cell signaling. And specific phospholipids such as dilauroyl phosphatidylcholine (DLPC) and diundecanoyl phosphatidylcholine (DUPC) identified as NR5A2 direct ligands, NR5A2-specific promoters are activated by DLPC and DUPC to regulate receptor activity (54). For our future research, focusing on the underlying mechanisms of lipids can help to explore drug targets for therapeutic applications.

Lipid biology is important both in normal digestive processes and in diseases that affect the pancreas, such as pancreatitis and pancreatic cancer. Previous studies have investigated the role and risk of lipids in pancreatic diseases (10, 51), but the effect of pancreatic disease on lipid metabolites is still unclear. The causal relationship between two subjects is sometimes difficult to determine in traditional research, and the MR analysis employed in our study helps mitigate potential confounders and reverse causality, thereby enhancing the robustness of our findings and allowing for more confident inferences regarding causality in the observed associations. On the basis of reverse MR analysis, pancreatic diseases had no causal relationship with liposomes. Some researchers have investigated lipid dysregulation characteristics in AP in rat models (55). By employing a time-course lipidomic method, they observed an overall decrease in glycerophospholipids within the pancreas and a significant reduction in serum TAGs at 24 h. The differences may be due to distinctions in animal testing and tissue sample selection, and most importantly, the MR Steiger filtering algorithm, which is used in the MR studies, greatly avoids the occurrence of false causality with the IVs. Our additional validation using UK Biobank datasets further proofed that pancreatitis has no causal relationship with liposomes.

The current study offers several advantages from its data sources and MR research design. First, MR design circumvents biases commonly found in traditional observational studies, and we conducted multiple sensitivity analyses to ensure reliability. Second, the GWAS data used in the present study were sourced from a sizable cohort of 7,174 individuals, encompassing 179 lipid species. Genetic associations and large-sample data may provide new insights into lipid metabolism and new lipid-associated risk factors for pancreatic diseases. Third, we applied the BWMR method to verify our results, which improved the accuracy of our findings. Some results did not pass the BWMR verification, so it is necessary to treat these results with caution, and further research can be carried out in the future. Fourth, our study included different types of pancreatitis, and examining different subgroups allowed us to more accurately apply our conclusions to the population.

Research limitations are also unavoidable. First, the GWAS database is publicly available, and the details of the participants are lacking; therefore, further subgroup analysis is not possible. Second, European ancestry limits the generalisability of results to the wider population, since the liposome data used in this study came from European populations, and there is a lack of corresponding liposome big data in other populations (such as Asian populations), the future research in this area needs to be carried out and further studies of Asian ancestry may be conducted to complement these conclusions in the future. Third, lipidomics involves a variety of lipids, and the analysis of the plasma lipidome belonging to 13 lipid classes was confined to four lipid types in this study. Due to the selection of different lipids and the differences in the study population, the results of our study are not exactly same as those of former observational studies. We used double MR analysis to reflect the impact of different datasets on MR analysis, and the results of FinnGen consortium and UK Biobank datasets have some similarities but there are also many differences. Through the double analysis, we found that certain lipid classes had relatively stable effects for AP and CP. FinnGen consortium R11 has more comprehensive and updated data with a larger number of cases, so our study is mainly based on R11 results. Fourth, some of the results of the five MR methods had different beta values, but most of the horizontal pleiotropy was not abnormal, and our results were mainly based on the IVW method. Fifth, despite using deleting outlier SNPs method for dealing with horizontal pleiotropy, caution should be exercised in interpreting causal associations derived from MR analysis due to the presence of horizontal pleiotropy in some positive results.

## Conclusion

Overall, this MR study have systematically elucidated causal associations between 179 lipid species and pancreatic diseases, such as four types of pancreatitis and pancreatic cancer. Sterol esters and phosphatidylcholine have effectively protective effects on acute and chronic pancreatitis. Many other lipids also play a potential role in pancreatic diseases. This work enhances our comprehension of lipid risk factors for the onset and progression of pancreatic diseases, potentially guiding the identification of new targets for therapeutic interventions. Future studies on the regulation of lipid metabolism may help to control the occurrence and development of pancreatic diseases.

## Data Availability

The datasets presented in this study can be found in online repositories. The names of the repository/repositories and accession number(s) can be found in the article/Supplementary material.
