# Supplementary material for: Genetically predicted causal link between the plasma lipidome and pancreatic diseases: a bidirectional Mendelian randomization study
Source: Front Nutr. 2025 Jan 15;11:1466509. doi: 10.3389/fnut.2024.1466509 (PMC11774697; doi:10.3389/fnut.2024.1466509)
Supplement: Supplementary file 12 [file Image_1.pdf]

Figure S1 Leave-one-out analysis (A), MR effect size (B), scatter plot (C) and funnel plot(D) for Sterol ester (27:1/14:0) levels on acute pancreatitis

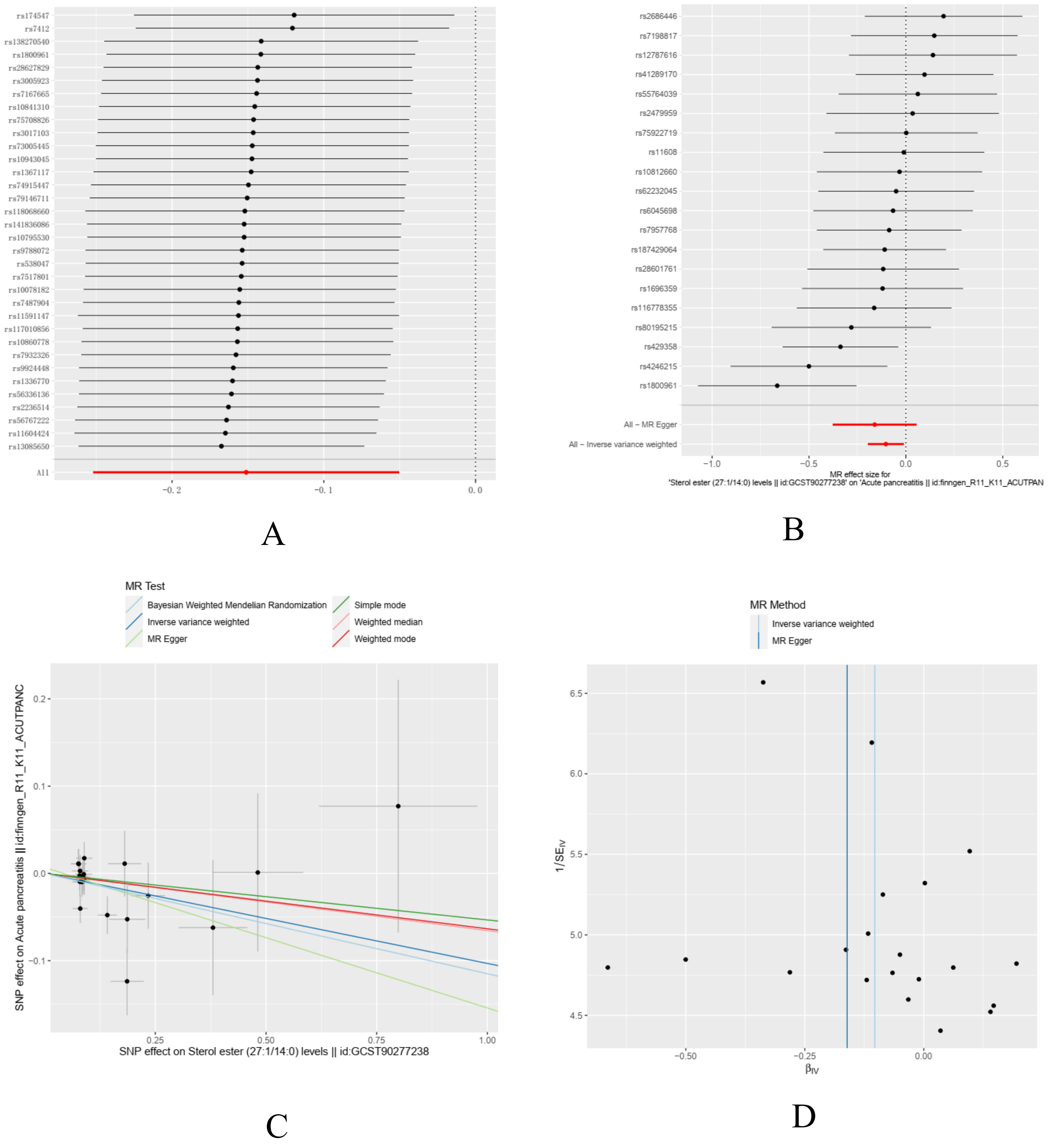

Figure S2 Leave-one-out analysis (A), MR effect size (B), scatter plot (C) and funnel plot(D) for Sterol ester (27:1/16:0) levels on acute pancreatitis

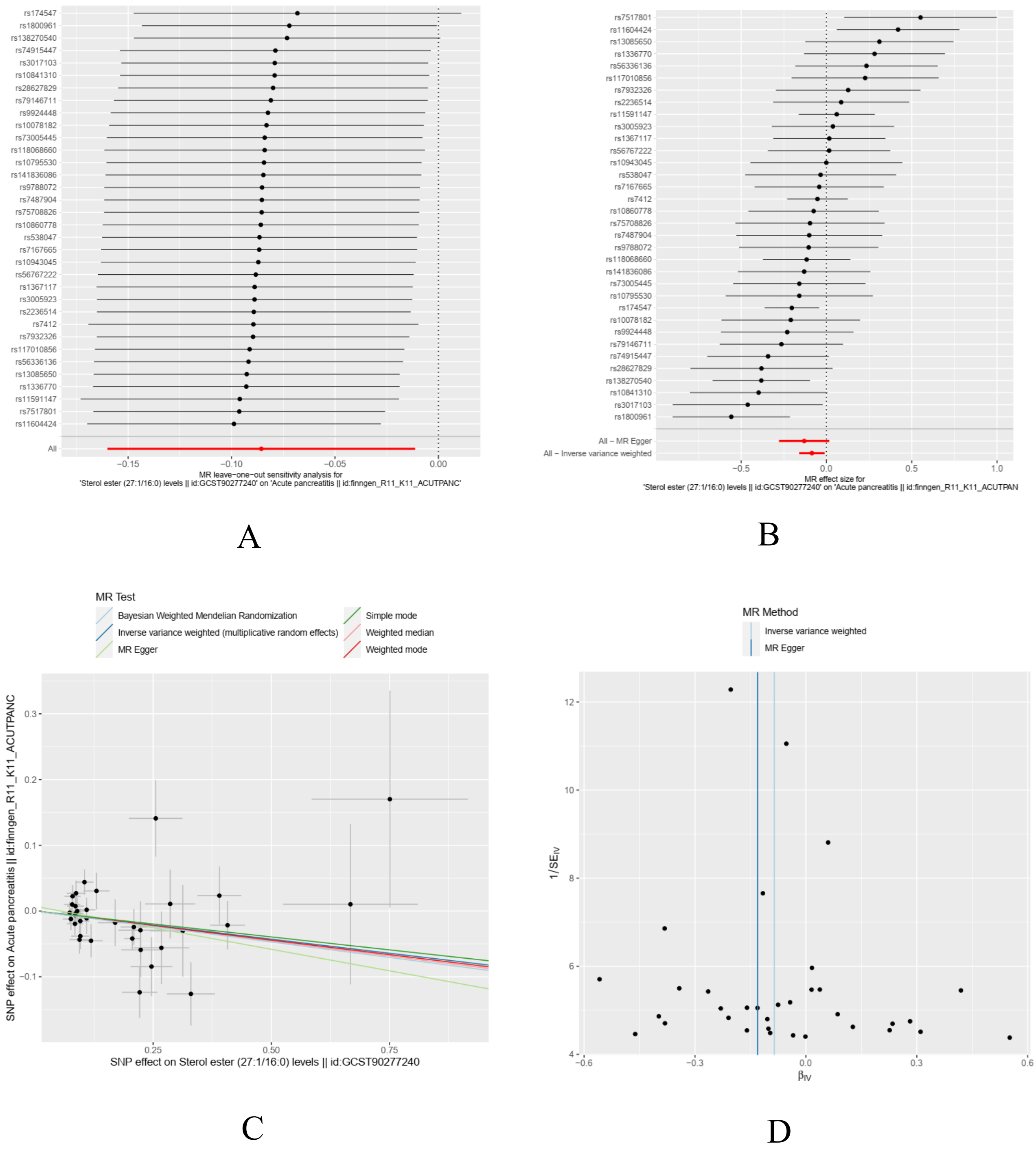

Figure S3 Leave-one-out analysis (A), MR effect size (B), scatter plot (C) and funnel plot(D) for Sterol ester (27:1/20:2) levels on acute pancreatitis

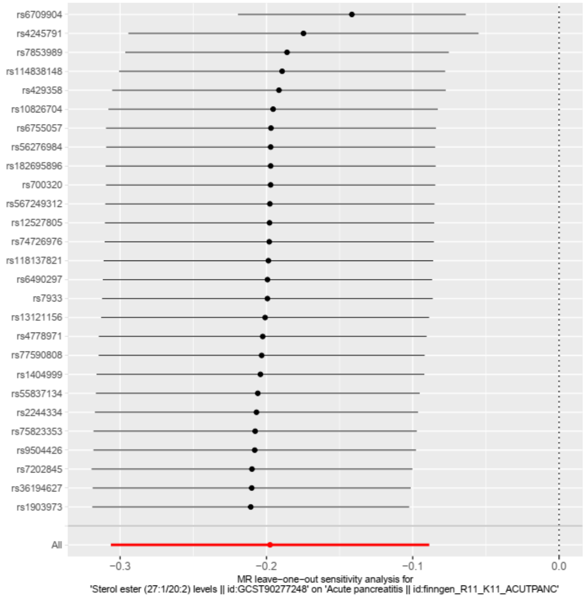

A

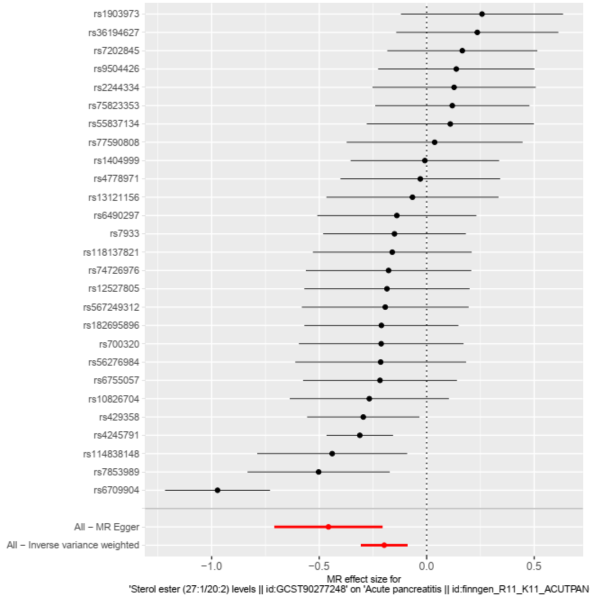

B

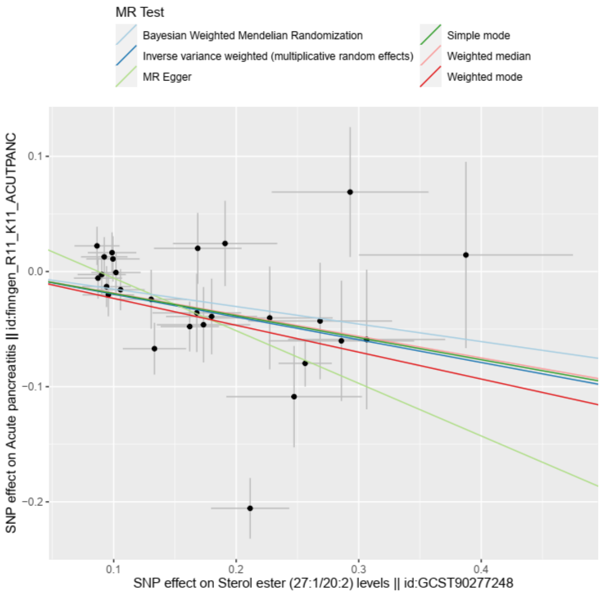

C

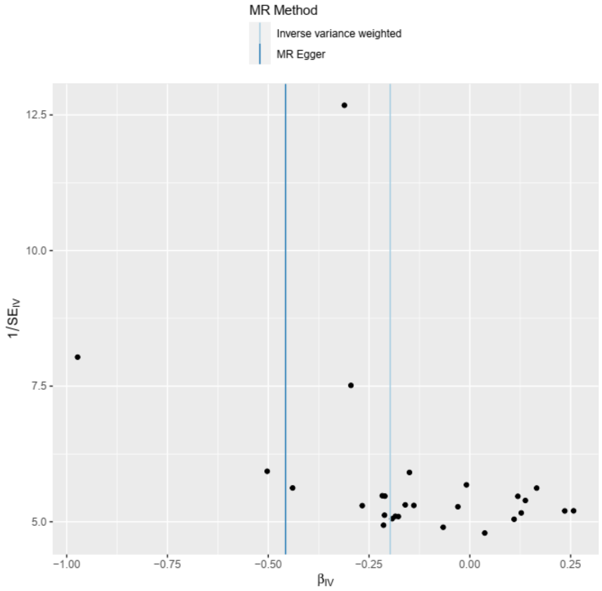

D

Figure S4 Leave-one-out analysis (A), MR effect size (B), scatter plot (C) and funnel plot(D) for Sterol ester (27:1/20:4) levels on acute pancreatitis

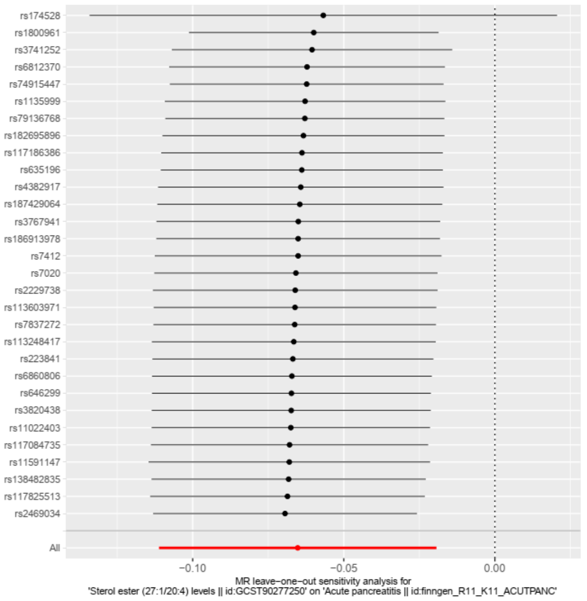

A

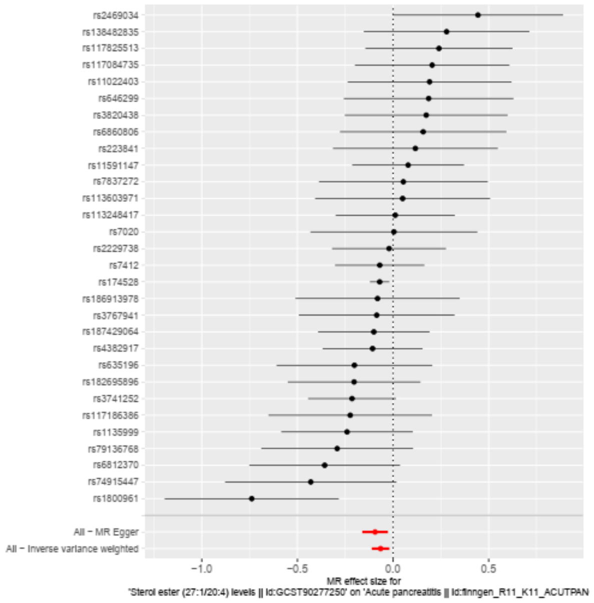

B

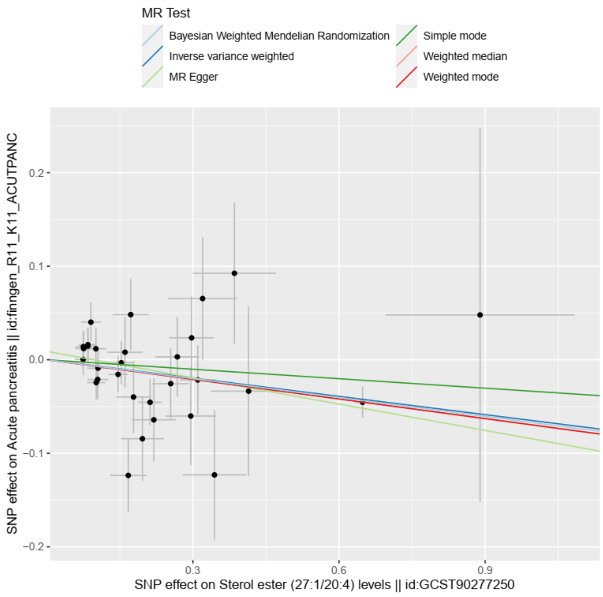

C

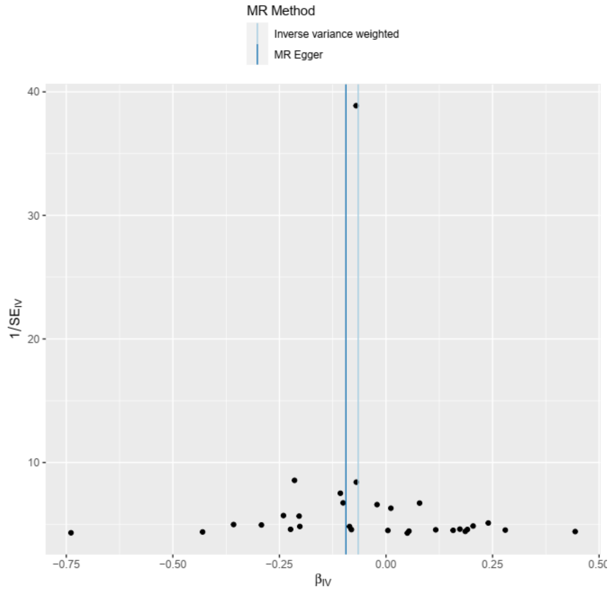

D

Figure S5 Leave-one-out analysis (A), MR effect size (B), scatter plot (C) and funnel plot(D) for Sterol ester (27:1/20:5) levels on acute pancreatitis

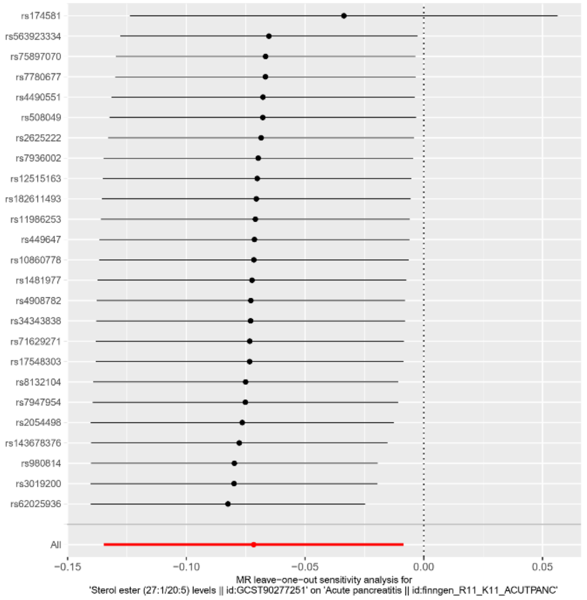

A

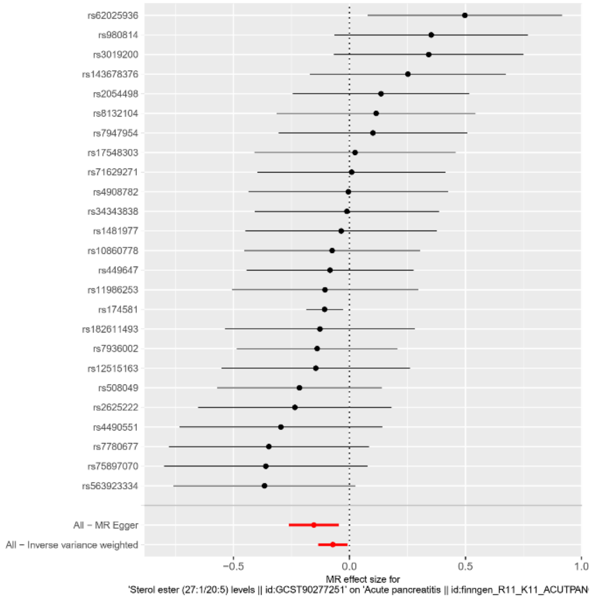

B

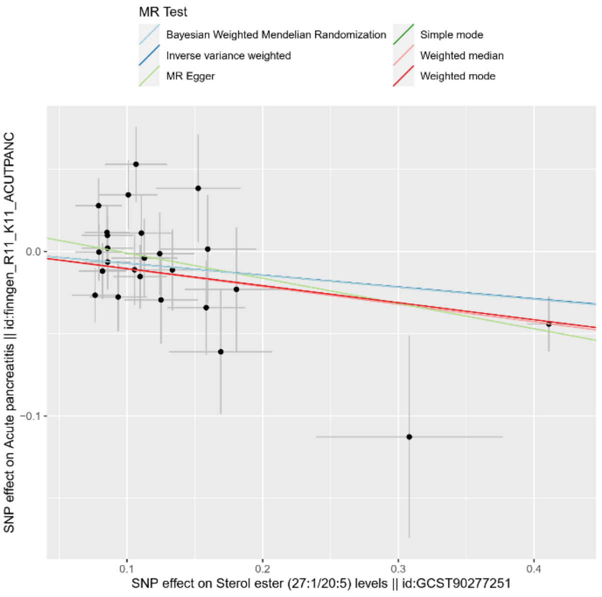

C

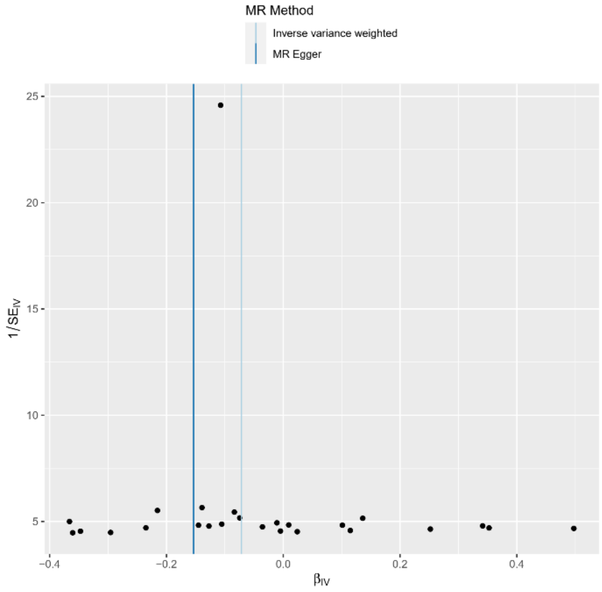

D

Figure S6 Leave-one-out analysis (A), MR effect size (B), scatter plot (C) and funnel plot(D) for Phosphatidylethanolamine (18:2_0:0) levels on acute pancreatitis

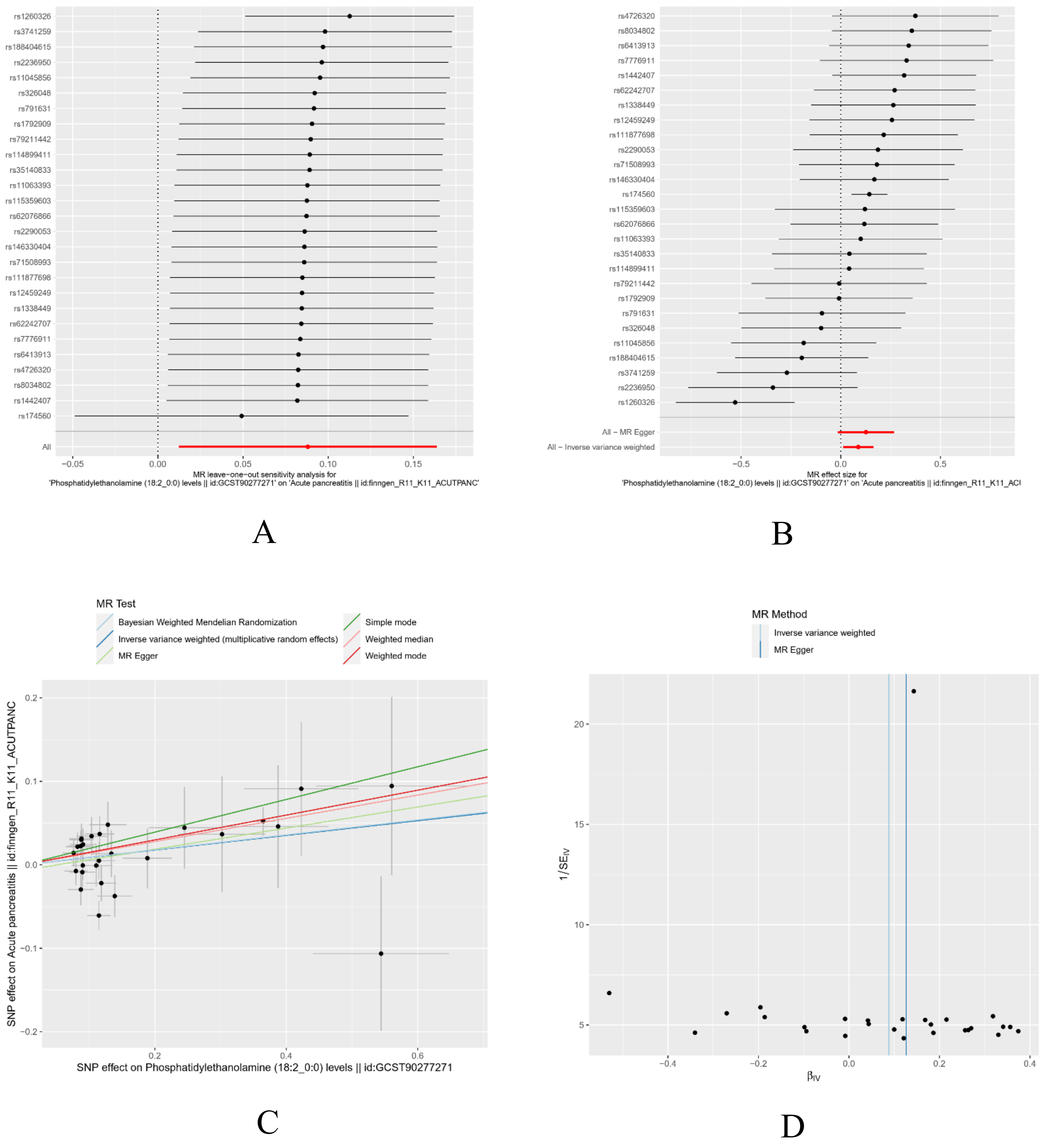

Figure S7 Leave-one-out analysis (A), MR effect size (B), scatter plot (C) and funnel plot(D) for Phosphatidylcholine (15:0_18:2) levels on acute pancreatitis

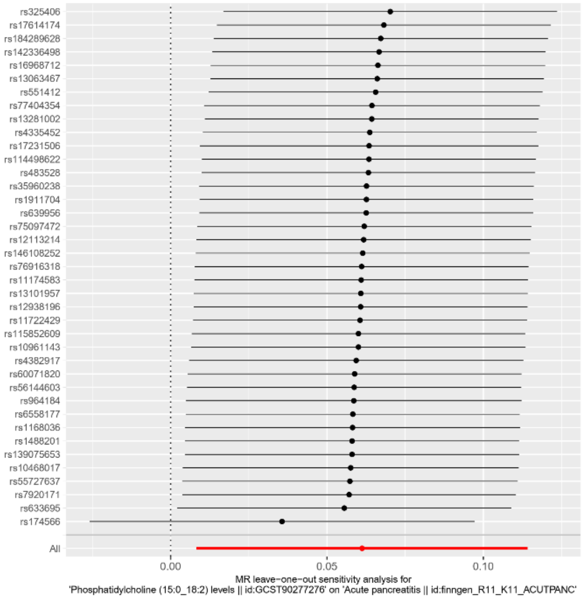

A

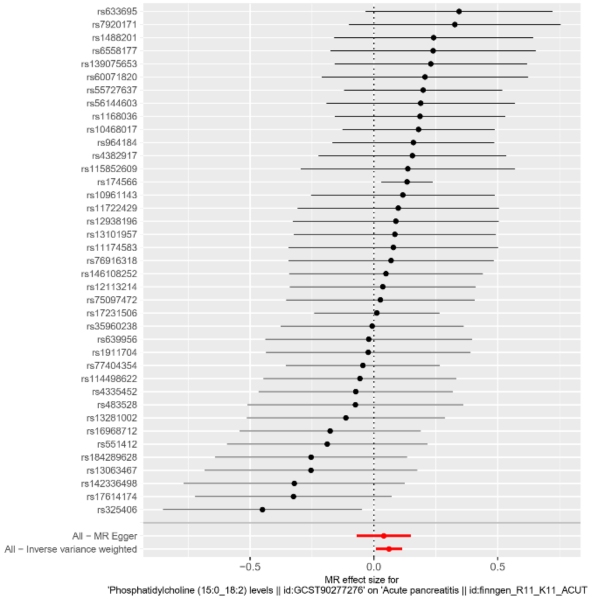

B

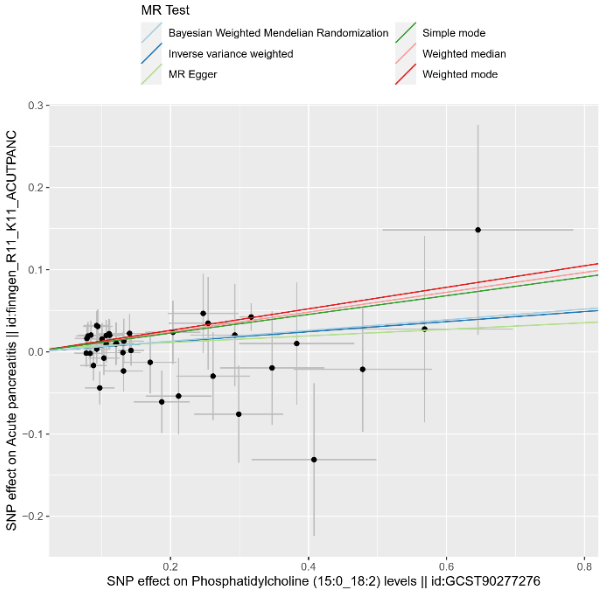

C

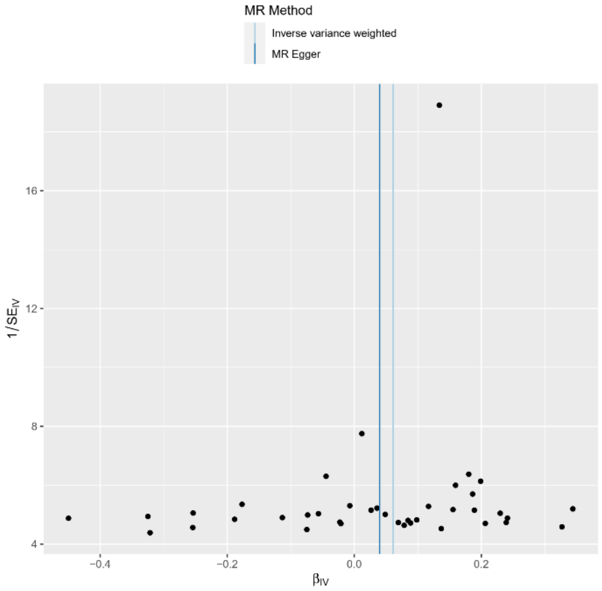

D

Figure S8 Leave-one-out analysis (A), MR effect size (B), scatter plot (C) and funnel plot(D) for Phosphatidylcholine (16:1_20:4) levels on acute pancreatitis

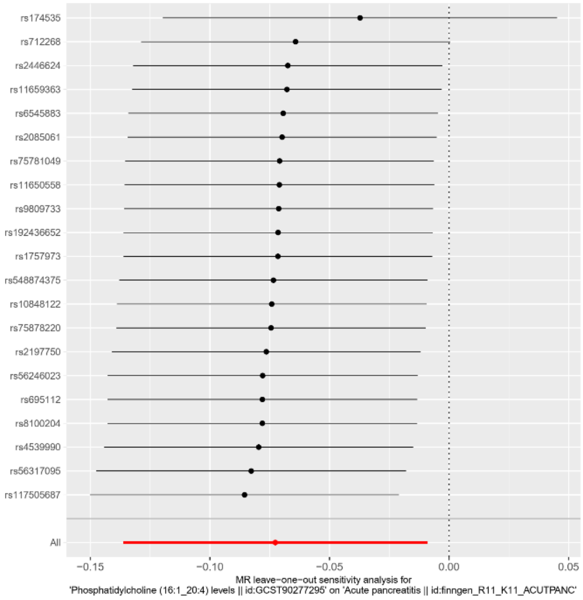

A

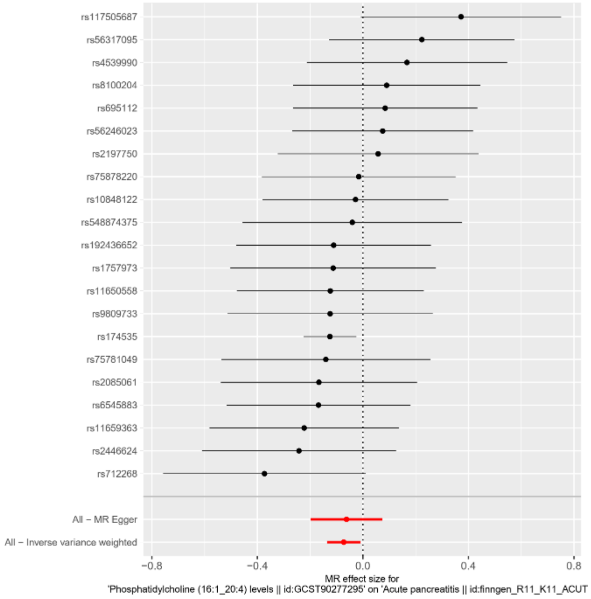

B

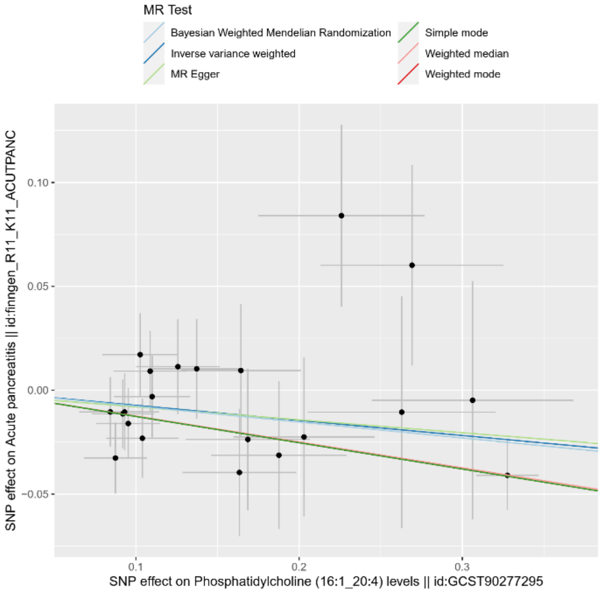

C

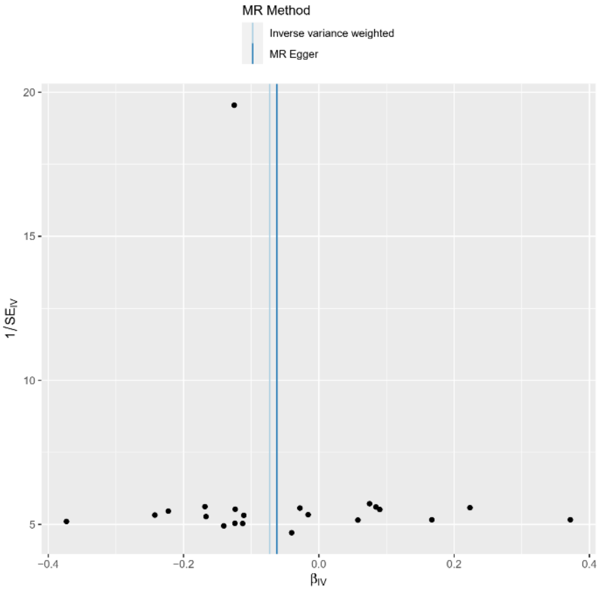

D

Figure S9 Leave-one-out analysis (A), MR effect size (B), scatter plot (C) and funnel plot(D) for Phosphatidylcholine (17:0_18:2) levels on acute pancreatitis

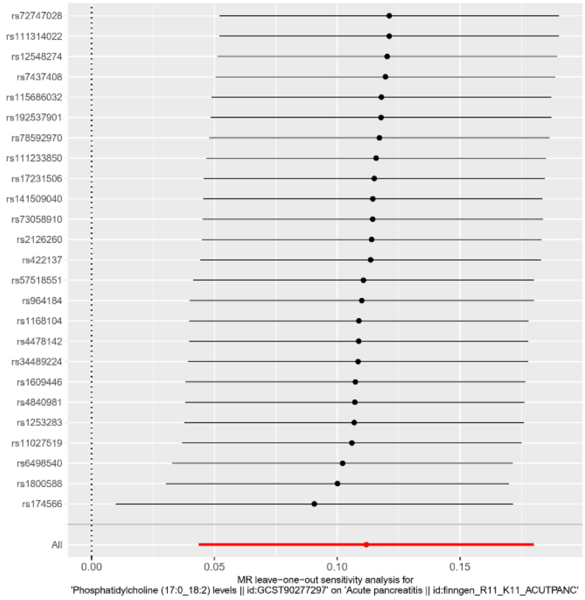

A

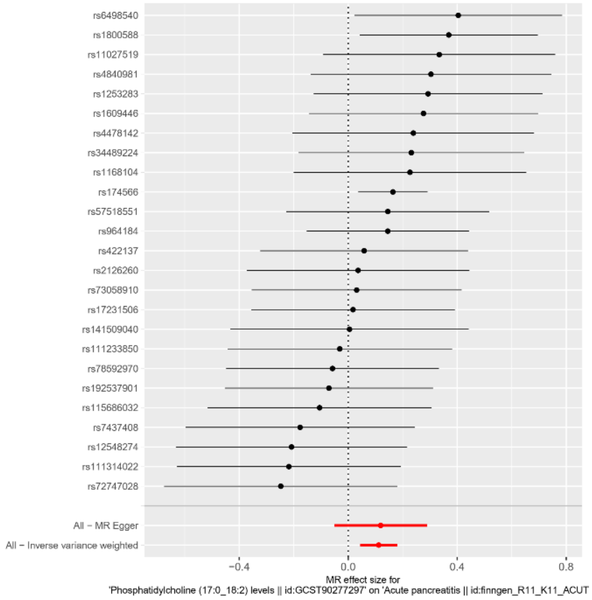

B

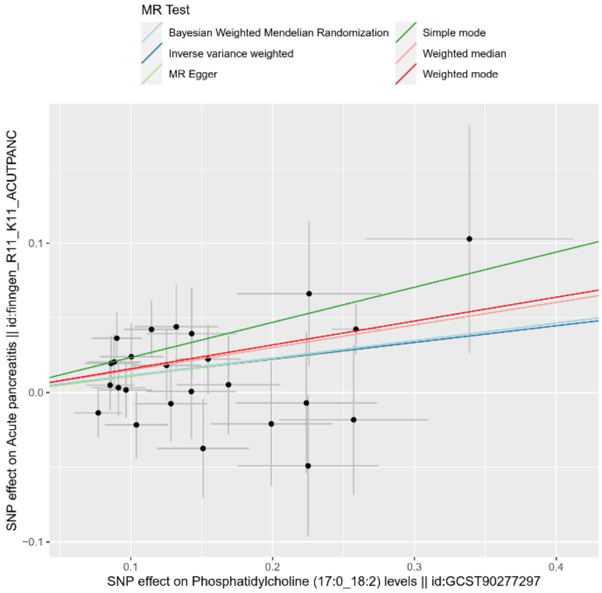

C

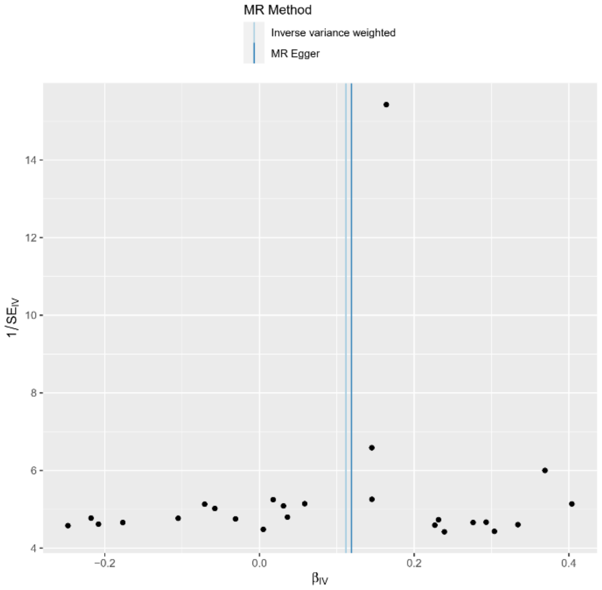

D

Figure S10 Leave-one-out analysis (A), MR effect size (B), scatter plot (C) and funnel plot(D) for Phosphatidylcholine (17:0_20:4) levels on acute pancreatitis

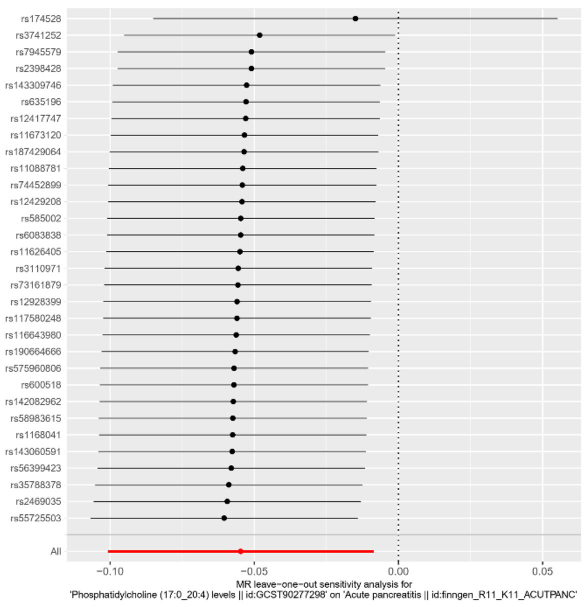

A

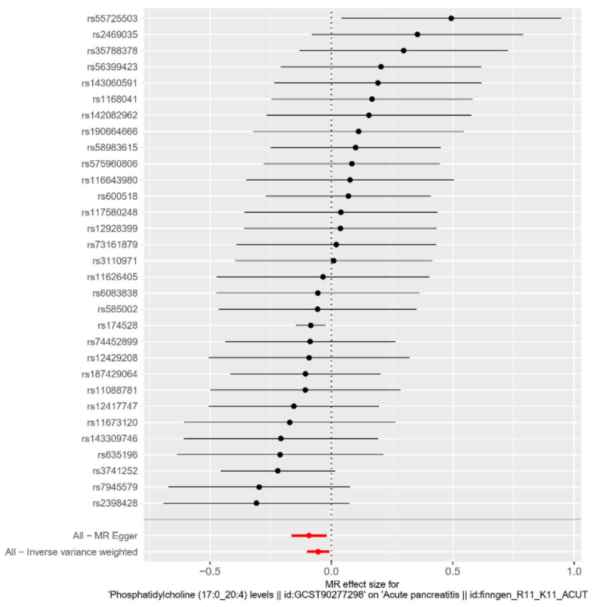

B

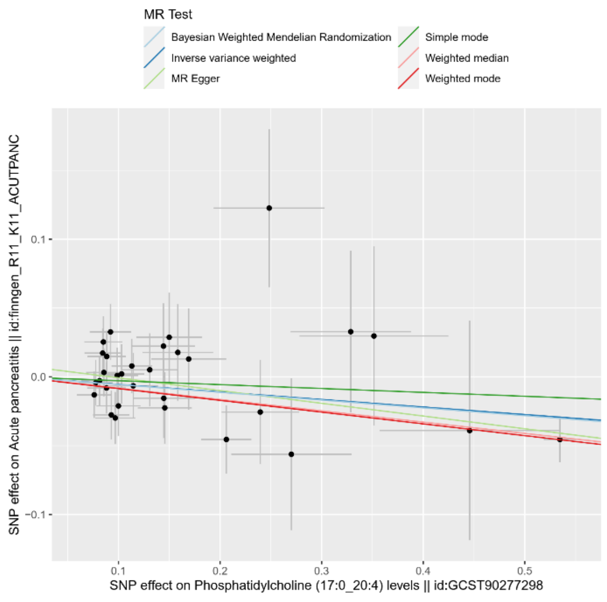

C

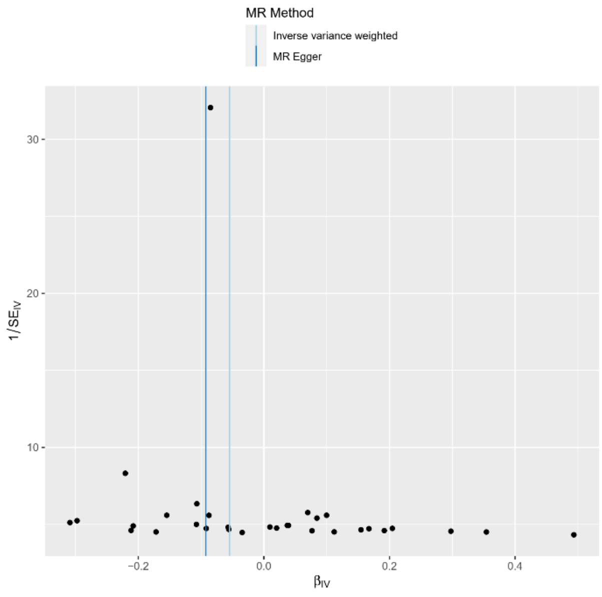

D

Figure S11 Leave-one-out analysis (A), MR effect size (B), scatter plot (C) and funnel plot(D) for Phosphatidylcholine (18:1_18:1) levels on acute pancreatitis

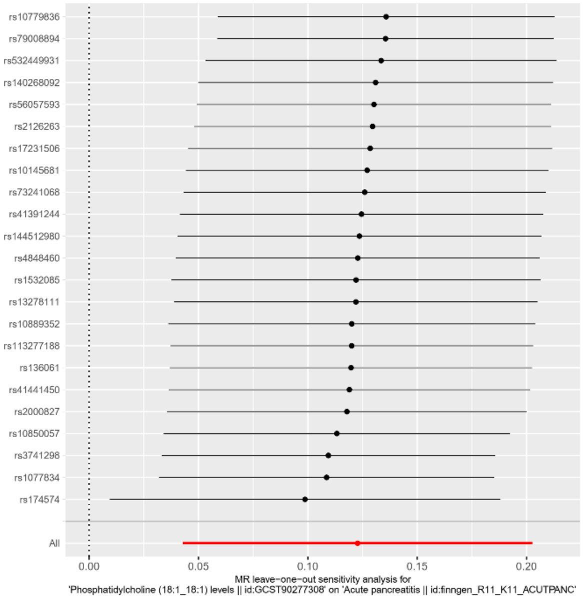

A

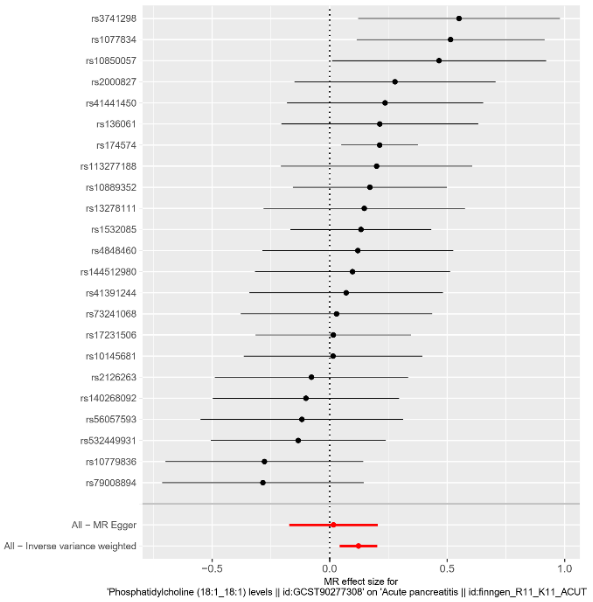

B

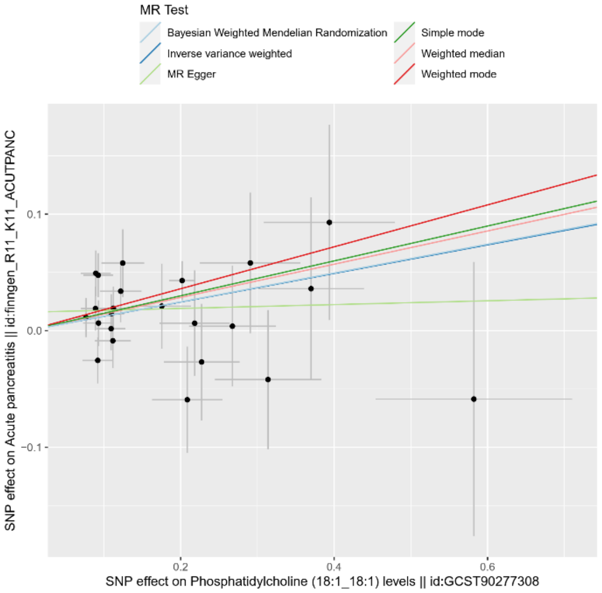

C

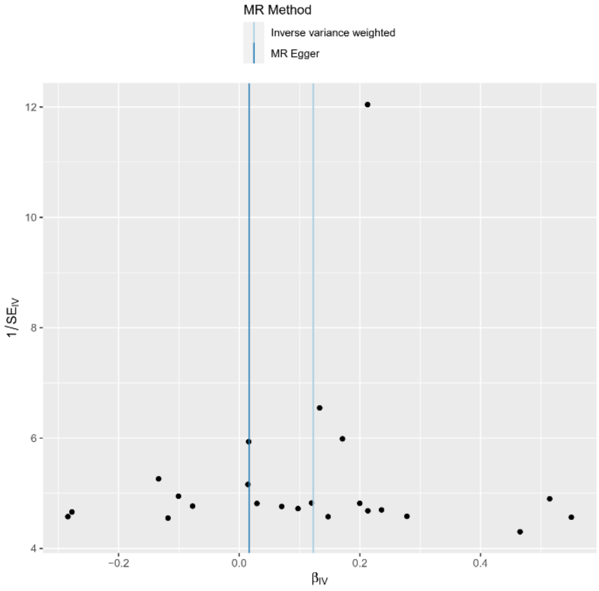

D

Figure S12 Leave-one-out analysis (A), MR effect size (B), scatter plot (C) and funnel plot(D) for Phosphatidylcholine (18:1_18:2) levels on acute pancreatitis

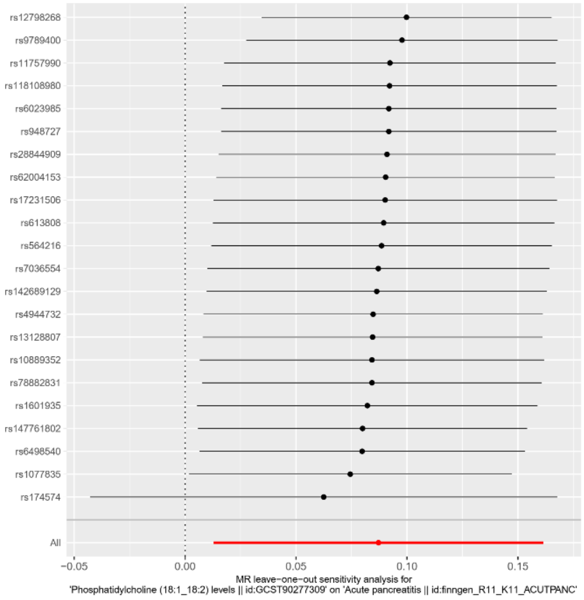

A

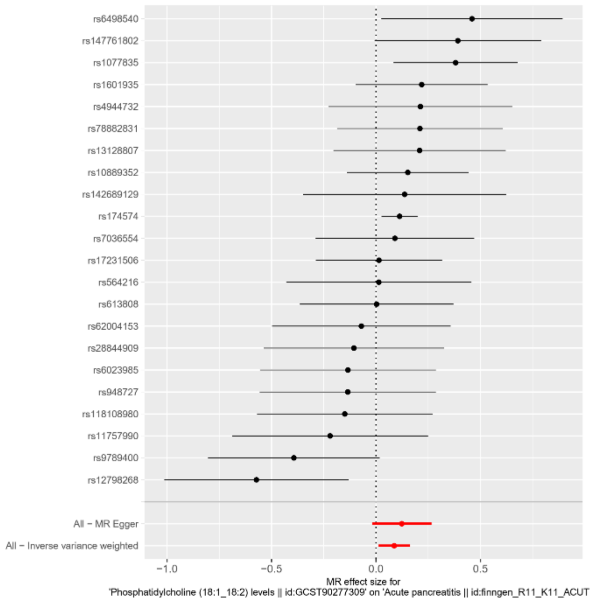

B

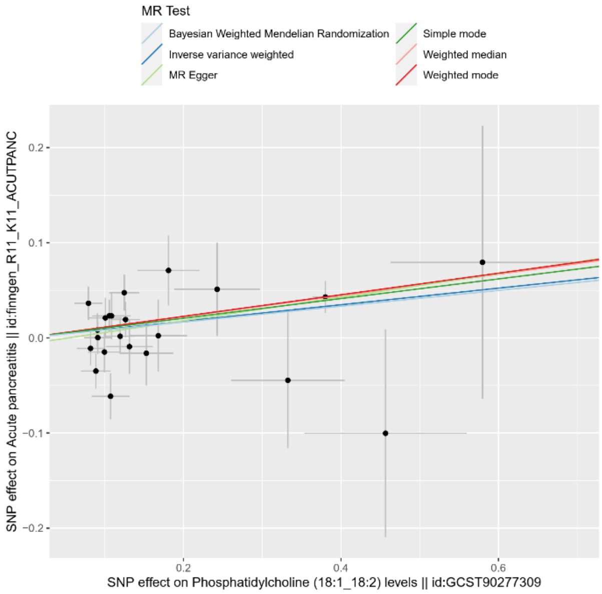

C

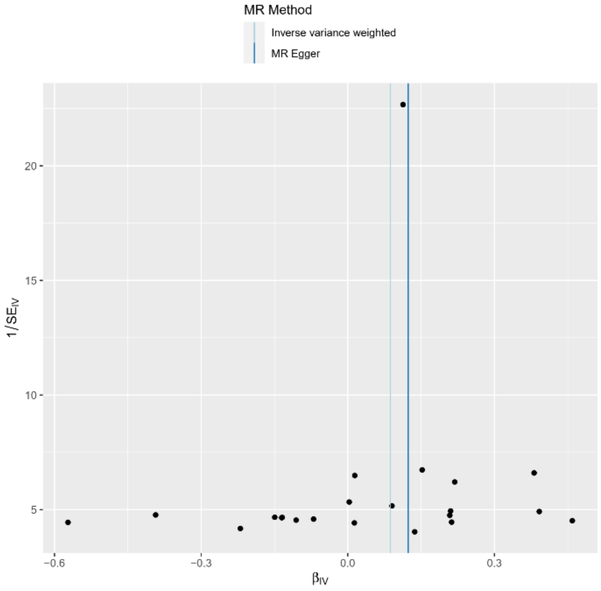

D

Figure S13 Leave-one-out analysis (A), MR effect size (B), scatter plot (C) and funnel plot(D) for Phosphatidylethanolamine (18:1_18:1) levels on acute pancreatitis

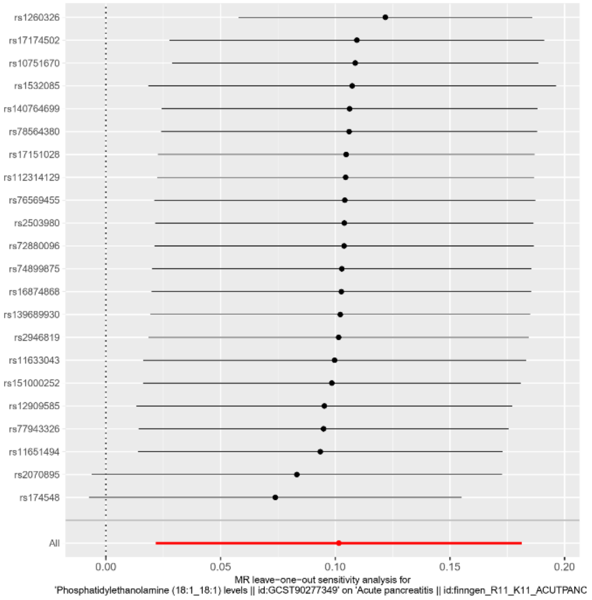

A

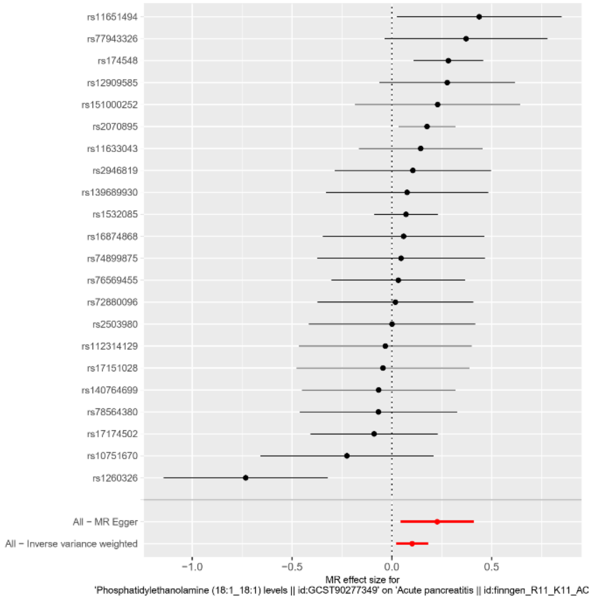

B

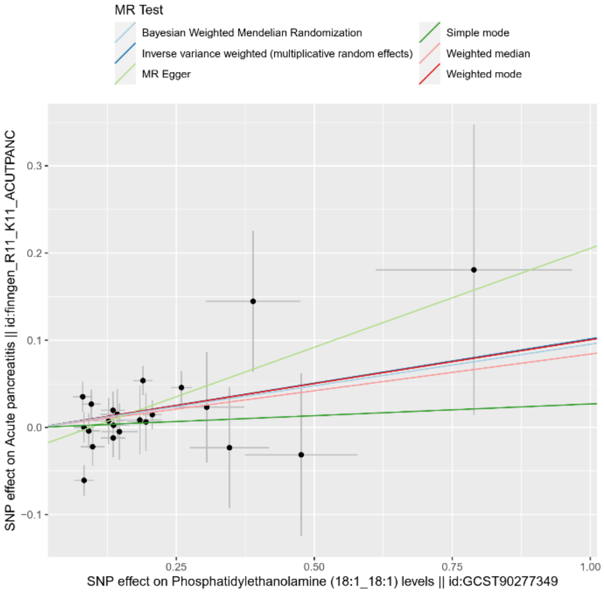

C

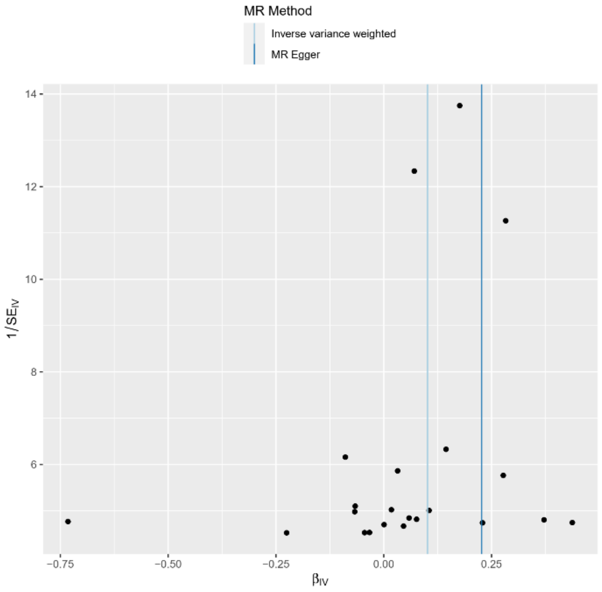

D

Figure S14 Leave-one-out analysis (A), MR effect size (B), scatter plot (C) and funnel plot(D) for Phosphatidylethanolamine (O-18:2_20:4) levels on acute pancreatitis

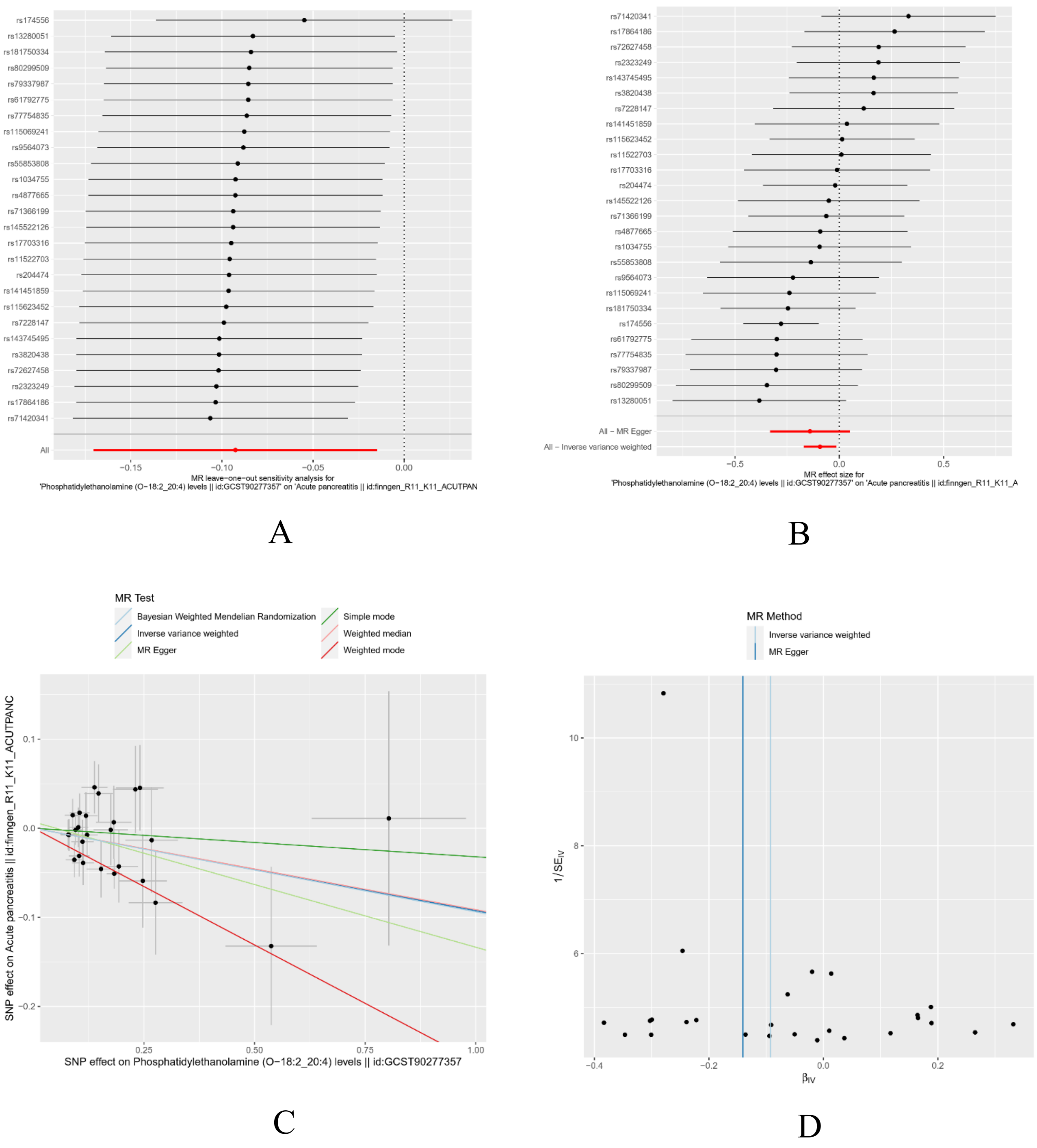

Figure S15 Leave-one-out analysis (A), MR effect size (B), scatter plot (C) and funnel plot(D) for Phosphatidylinositol (18:0_20:3) levels on acute pancreatitis

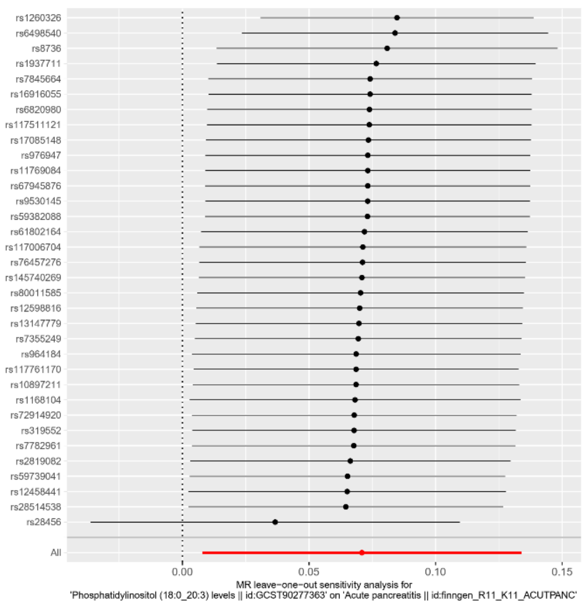

A

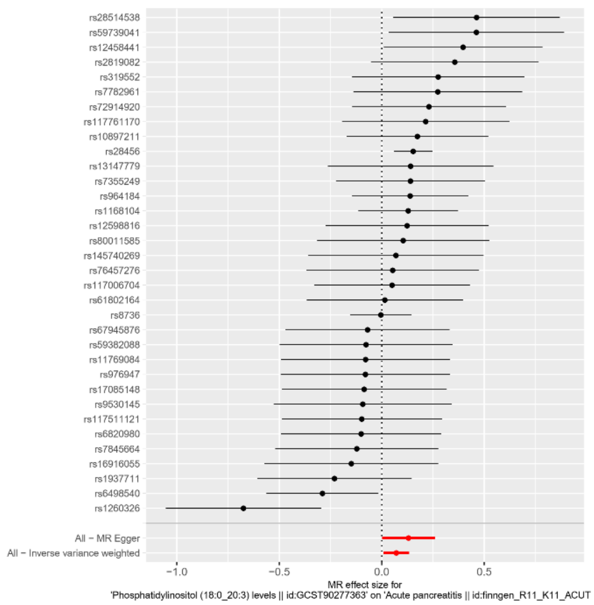

B

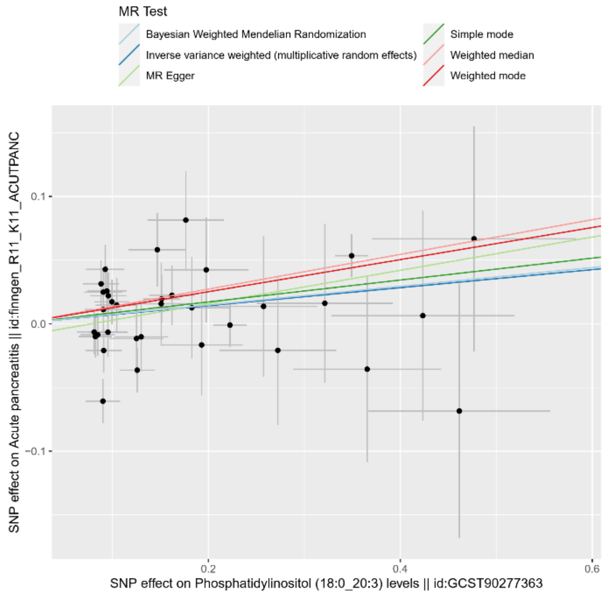

C

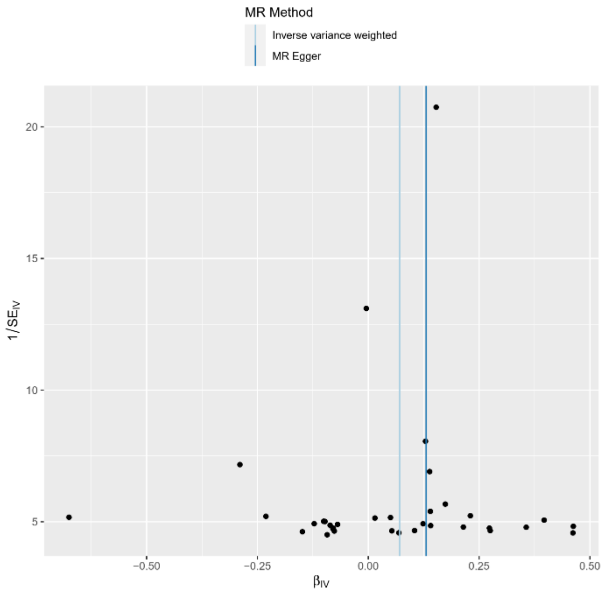

D

Figure S16 Leave-one-out analysis (A), MR effect size (B), scatter plot (C) and funnel plot(D) for Sphingomyelin (d34:1) levels on acute pancreatitis

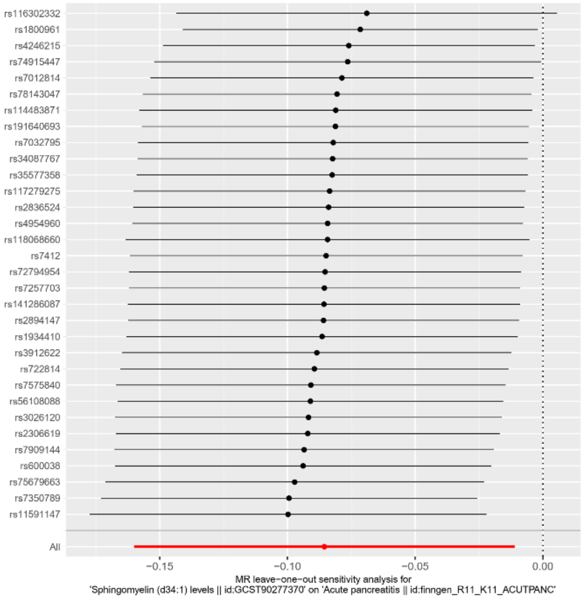

A

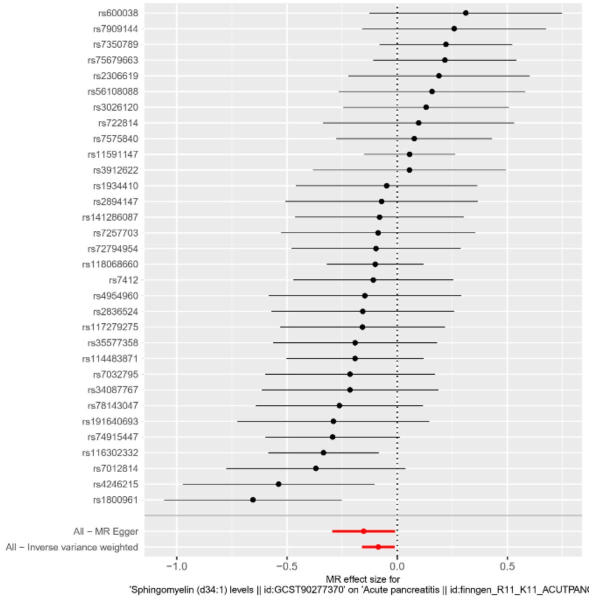

B

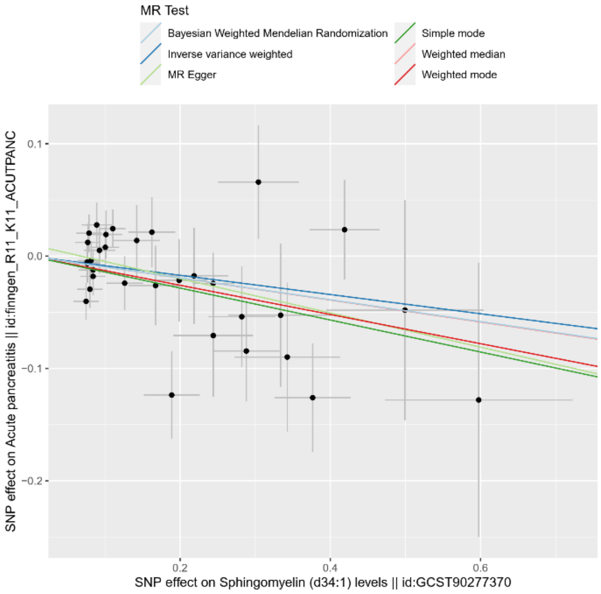

C

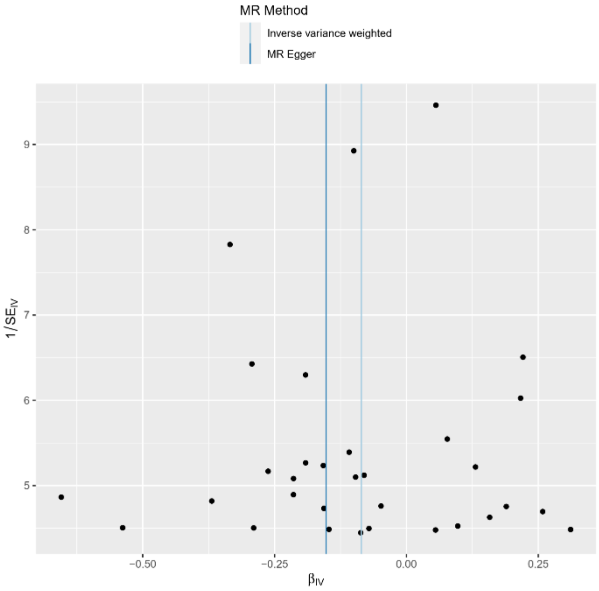

D

Figure S17 Leave-one-out analysis (A), MR effect size (B), scatter plot (C) and funnel plot(D) for Sphingomyelin (d34:2) levels on acute pancreatitis

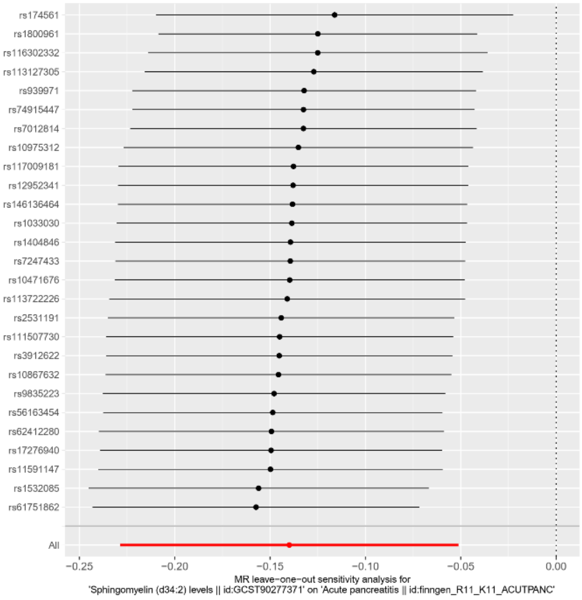

A

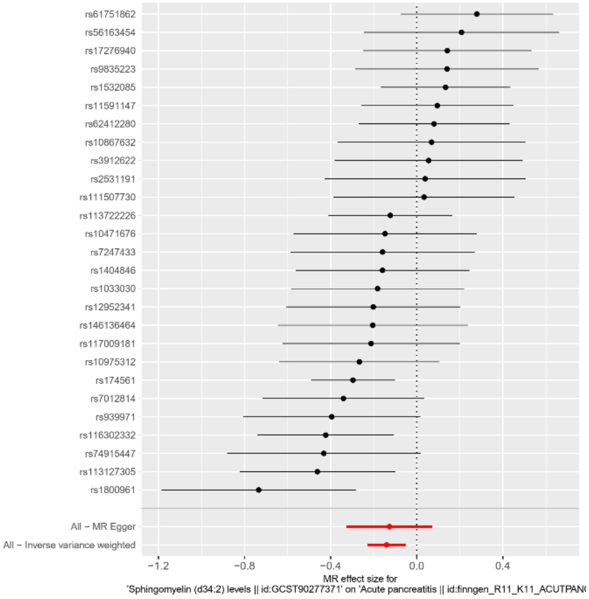

B

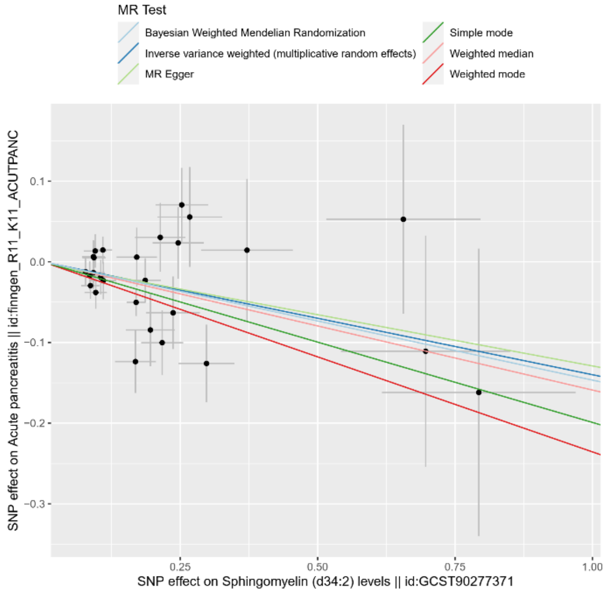

C

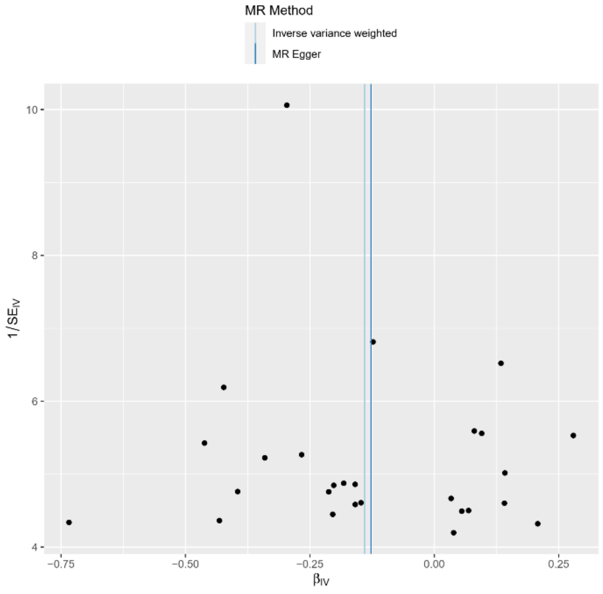

D

Figure S18 Leave-one-out analysis (A), MR effect size (B), scatter plot (C) and funnel plot(D) for Sphingomyelin (d40:2) levels on acute pancreatitis

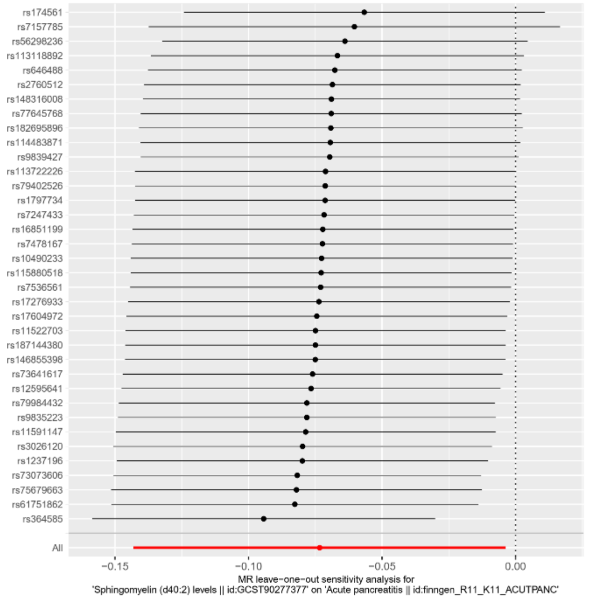

A

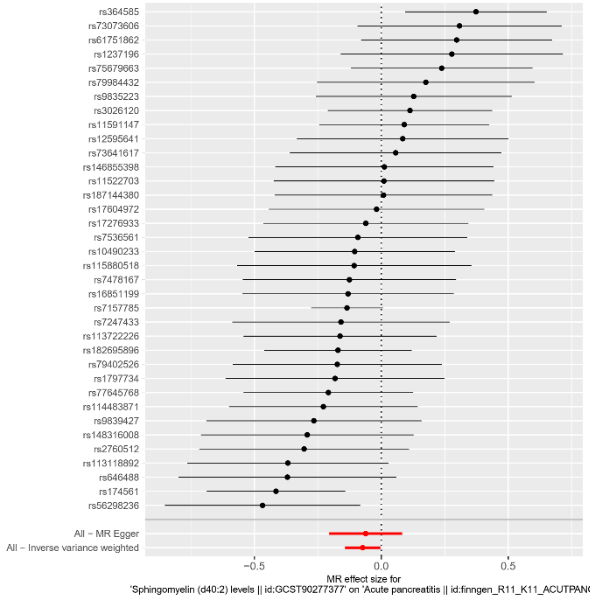

B

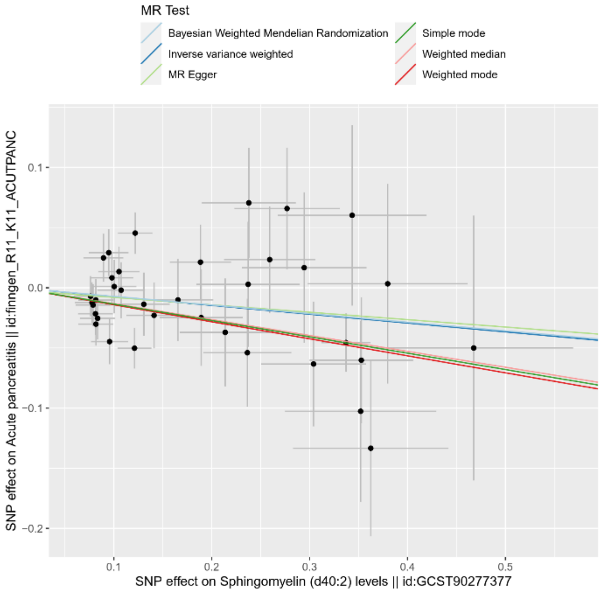

C

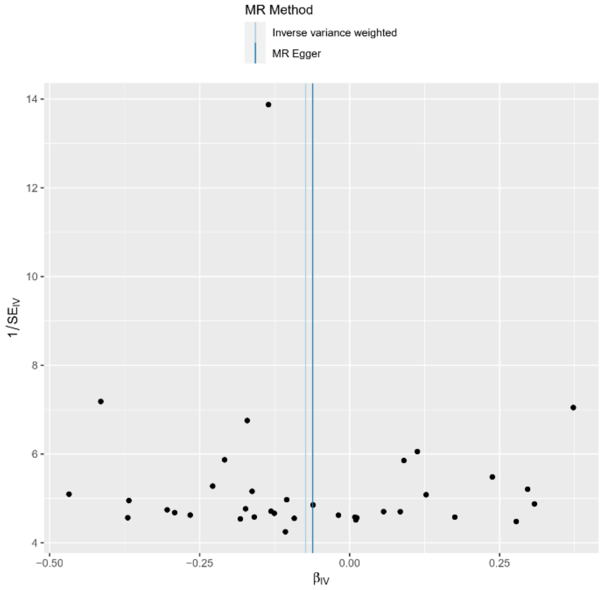

D

Figure S19 Leave-one-out analysis (A), MR effect size (B), scatter plot (C) and funnel plot(D) for Triacylglycerol (48:1) levels on acute pancreatitis

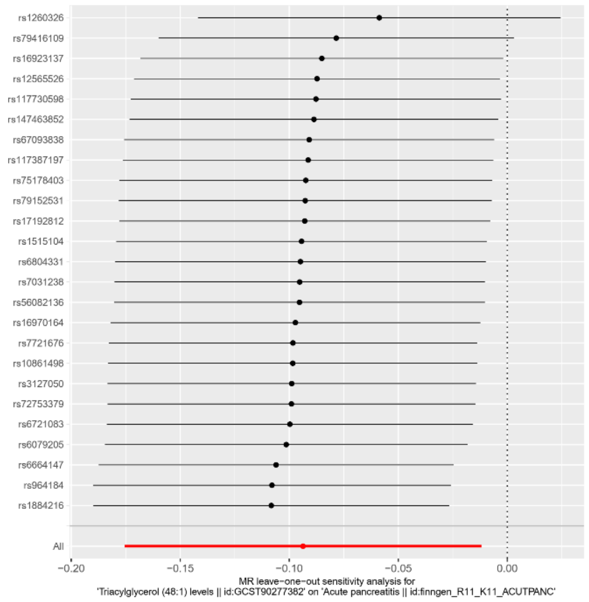

A

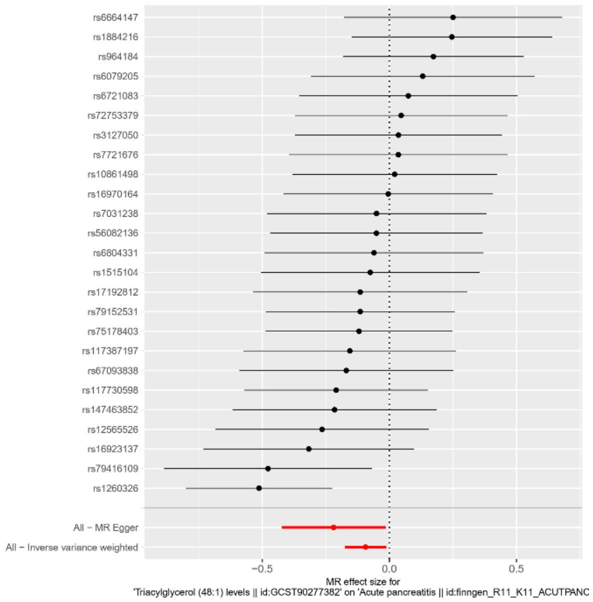

B

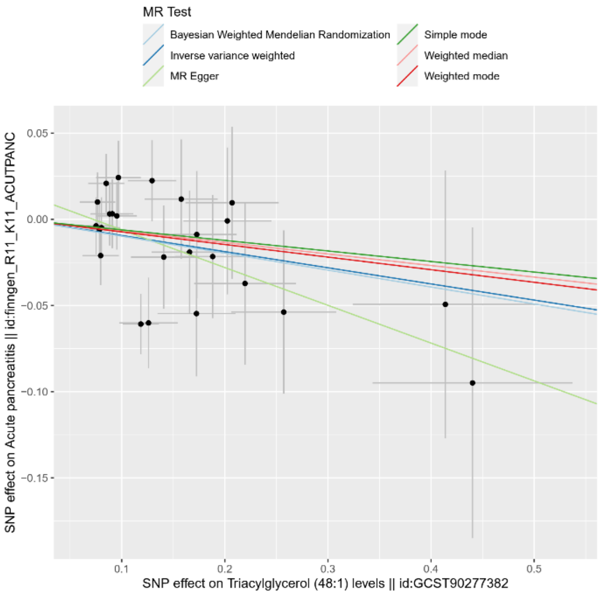

C

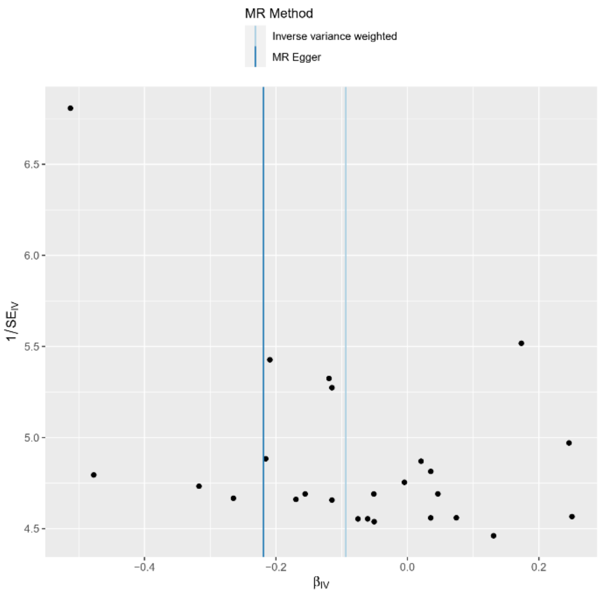

D

Figure S20 Leave-one-out analysis (A), MR effect size (B), scatter plot (C) and funnel plot(D) for Triacylglycerol (50:2) levels on acute pancreatitis

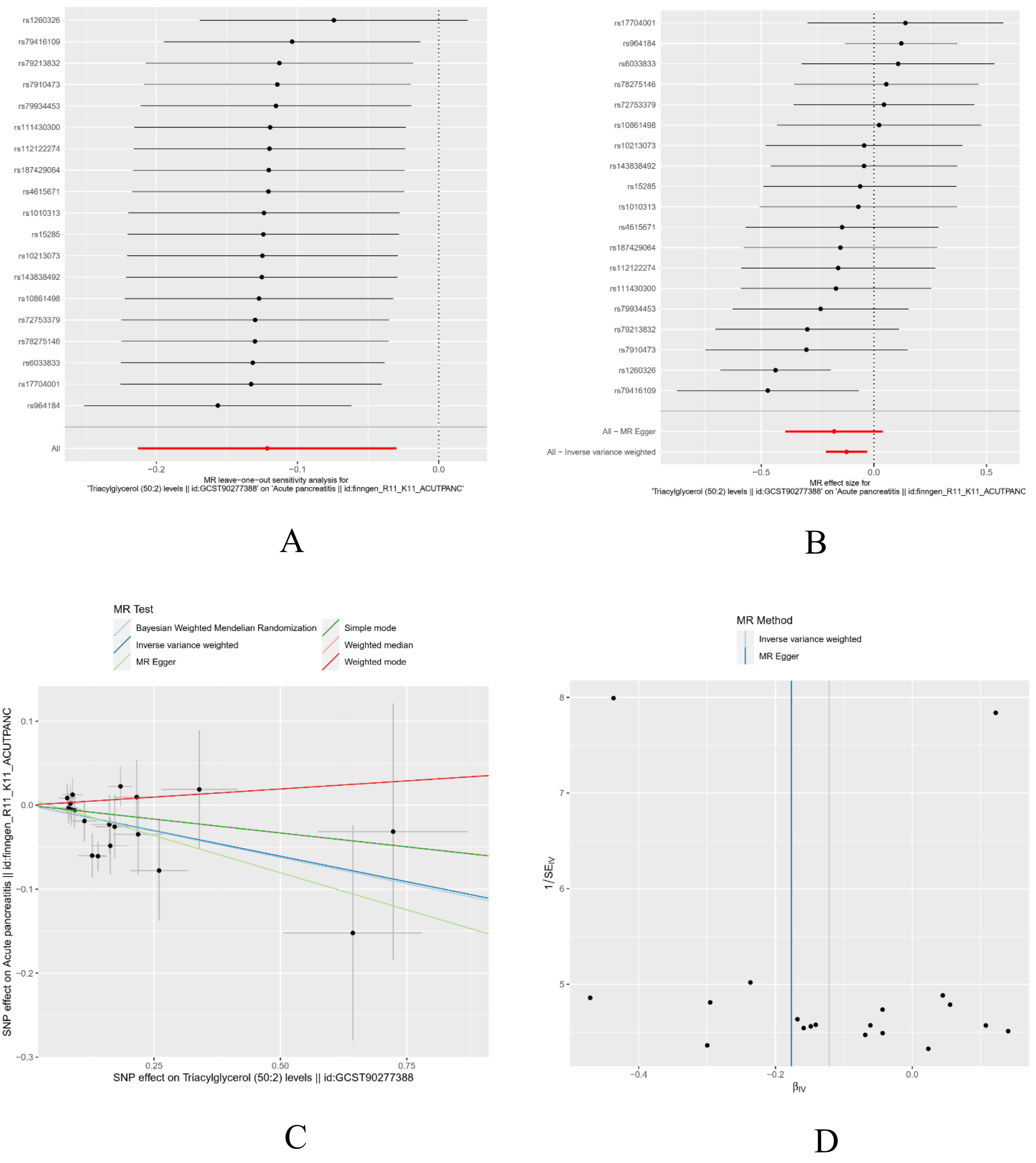

Figure S21 Leave-one-out analysis (A), MR effect size (B), scatter plot (C) and funnel plot(D) for Triacylglycerol (50:4) levels on acute pancreatitis

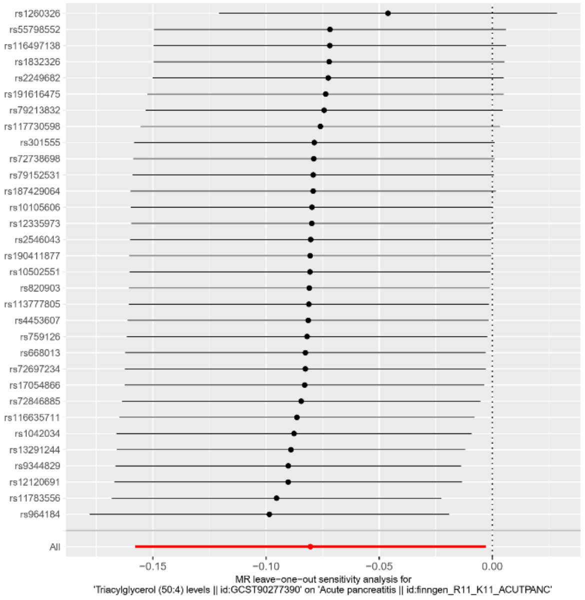

A

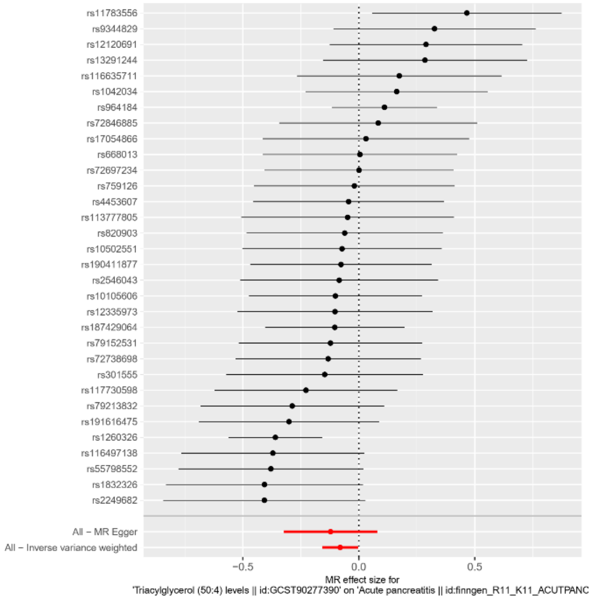

B

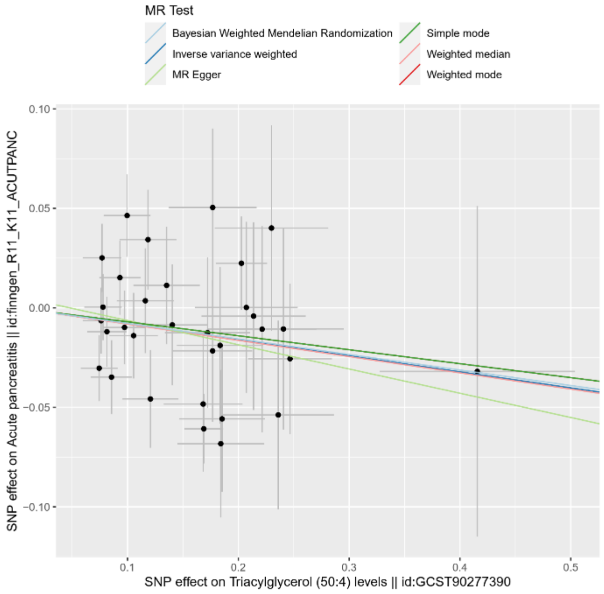

C

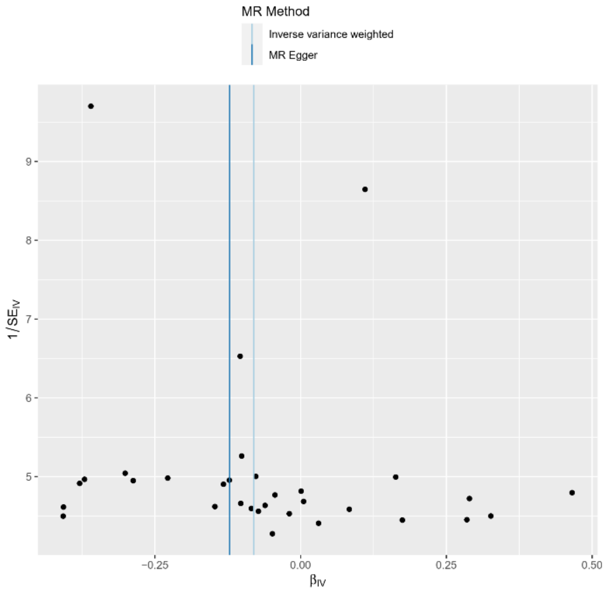

D

Figure S22 Leave-one-out analysis (A), MR effect size (B), scatter plot (C) and funnel plot(D) for Triacylglycerol (52:6) levels on acute pancreatitis

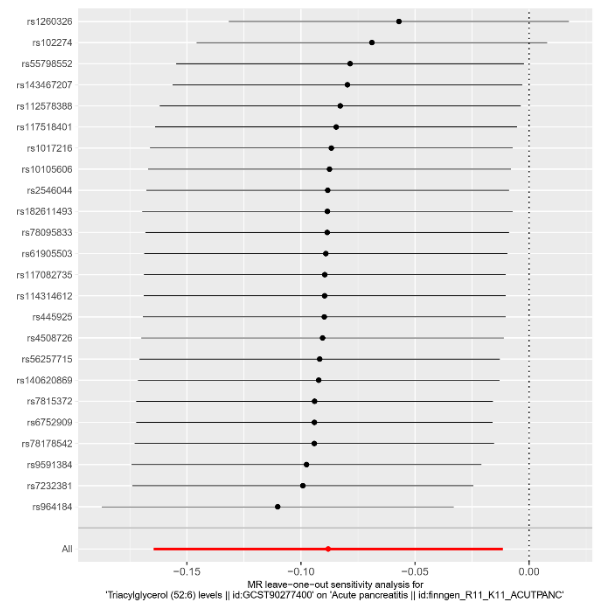

A

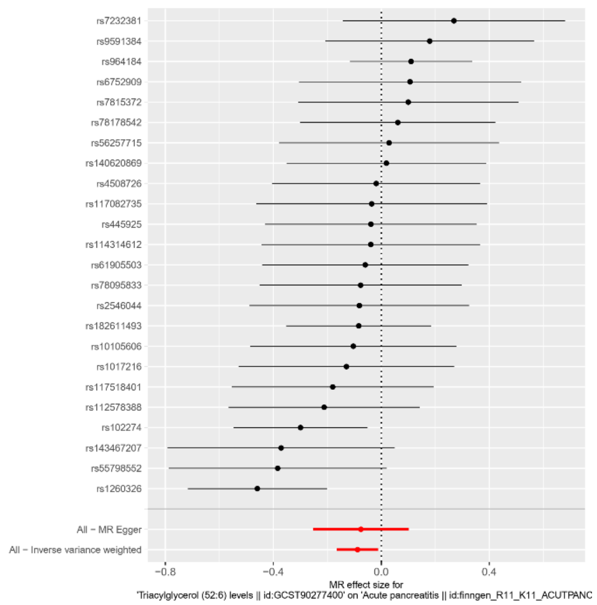

B

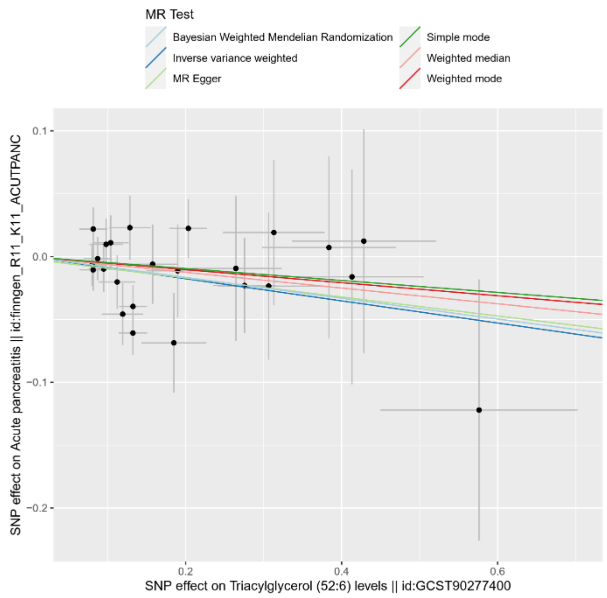

C

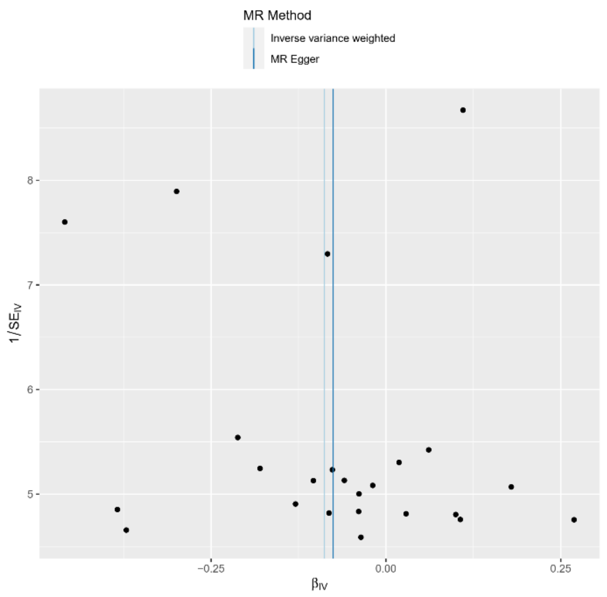

D

Figure S23 Leave-one-out analysis (A), MR effect size (B), scatter plot (C) and funnel plot(D) for Triacylglycerol (54:3) levels on acute pancreatitis

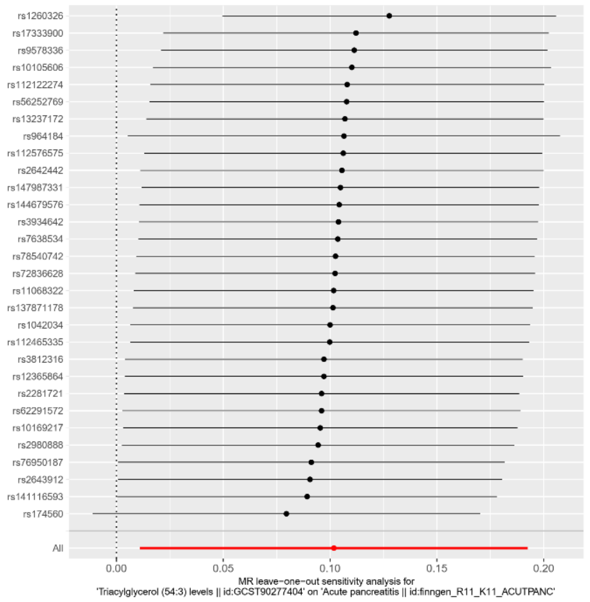

A

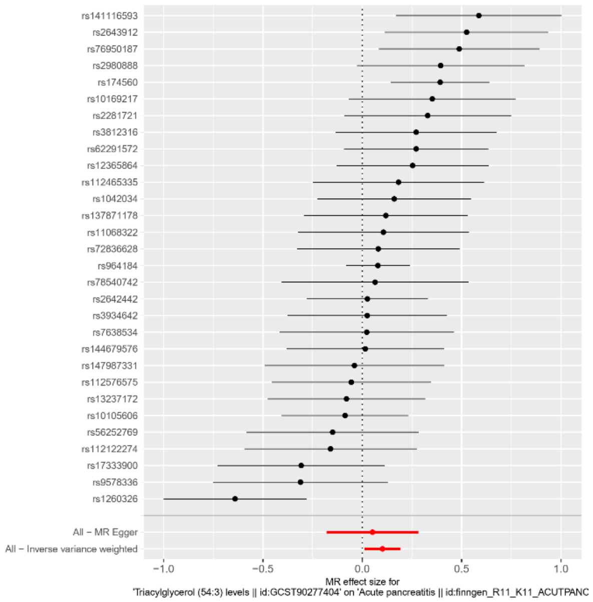

B

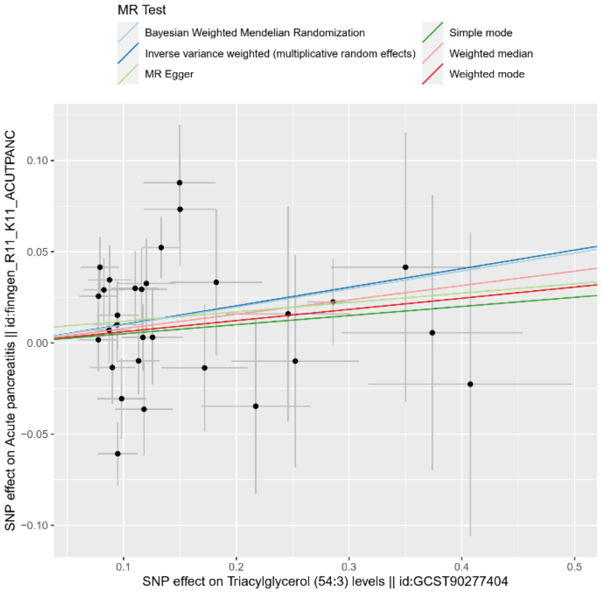

C

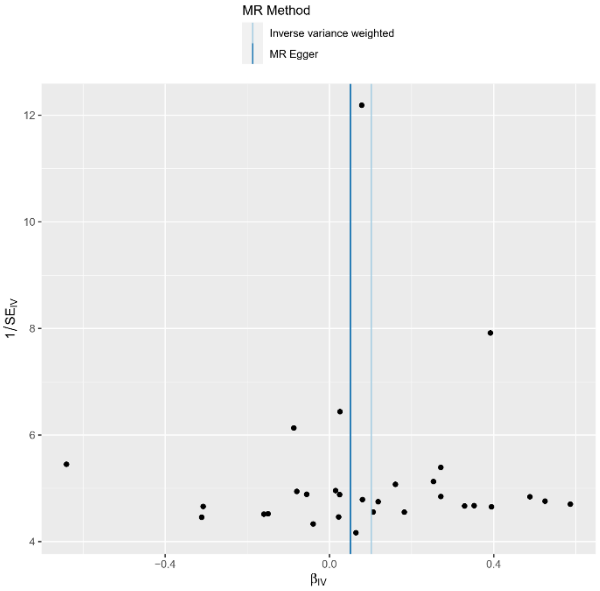

D

Figure S24 Leave-one-out analysis (A), MR effect size (B), scatter plot (C) and funnel plot(D) for Triacylglycerol (56:6) levels on acute pancreatitis

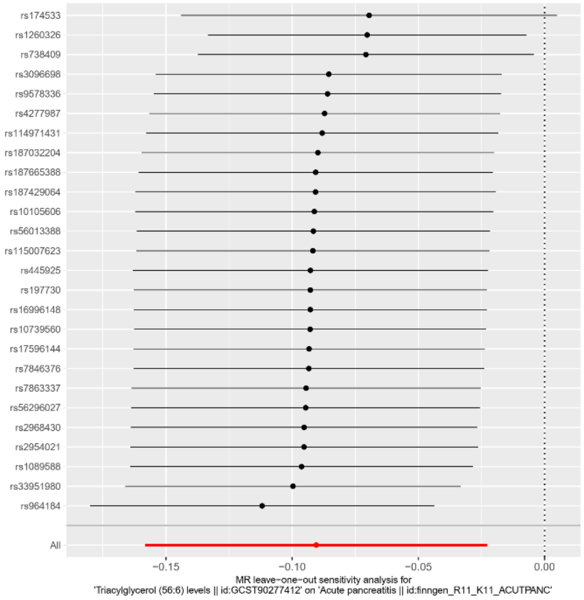

A

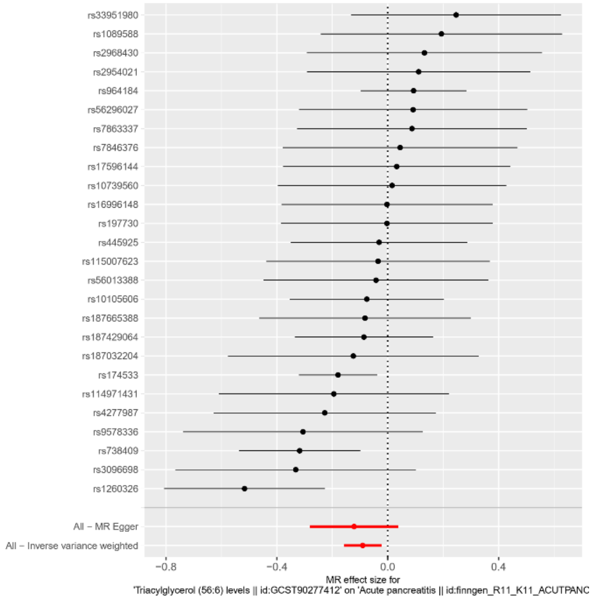

B

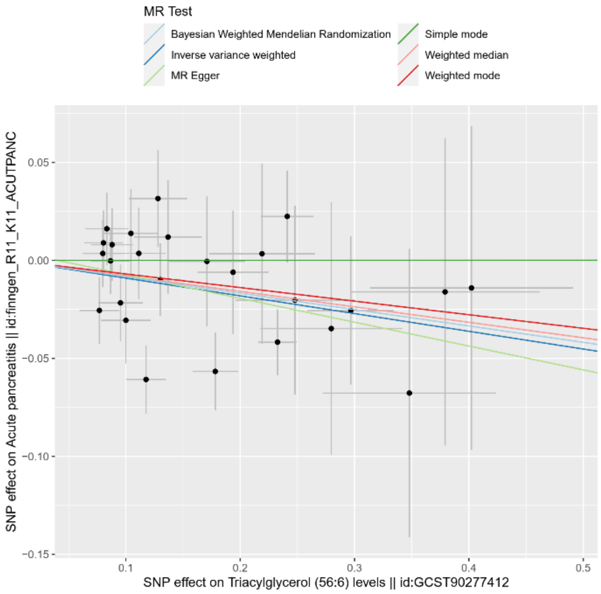

C

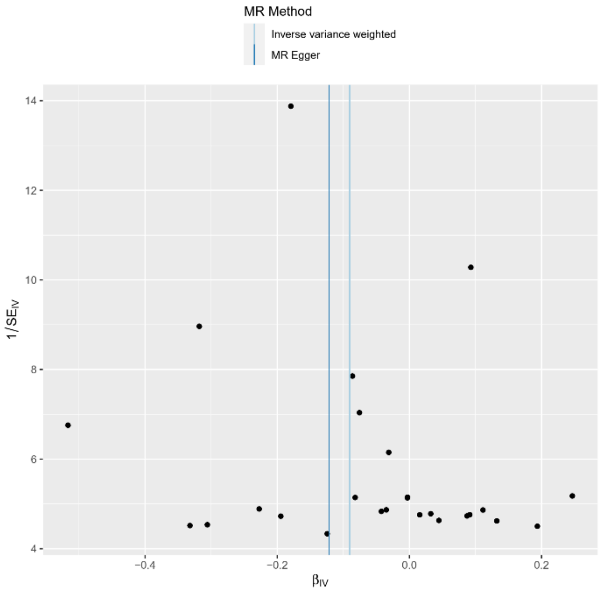

D

Figure S25 Leave-one-out analysis (A), MR effect size (B), scatter plot (C) and funnel plot(D) for Triacylglycerol (56:7) levels on acute pancreatitis

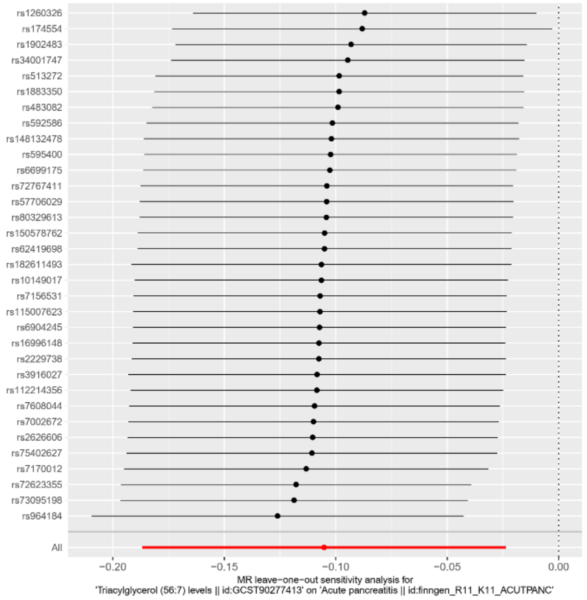

A

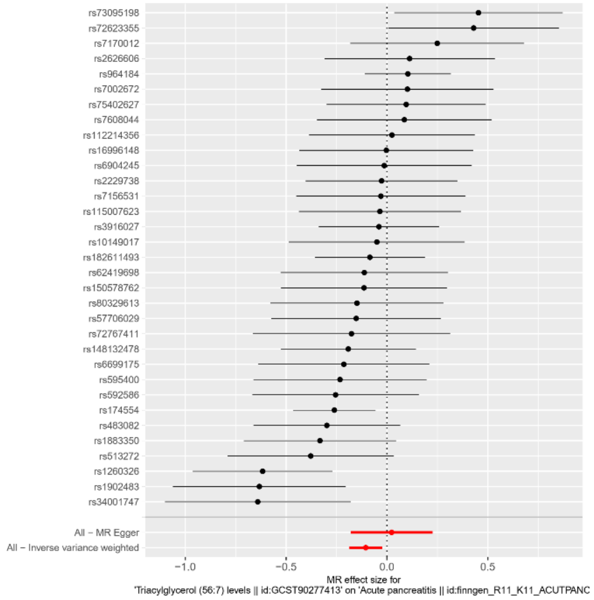

B

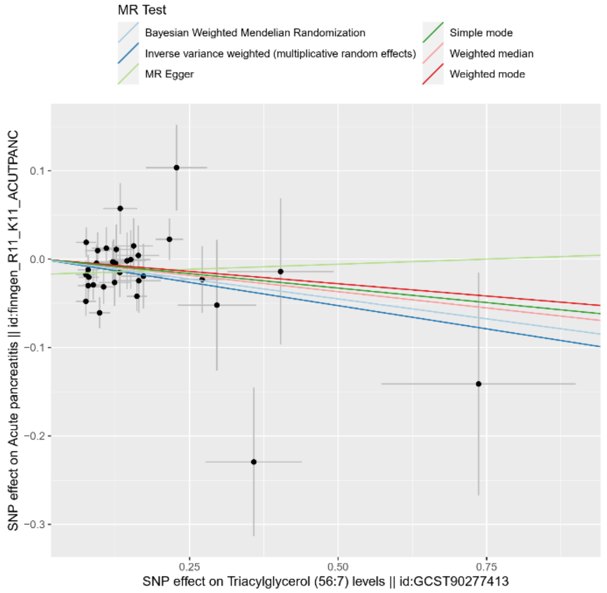

C

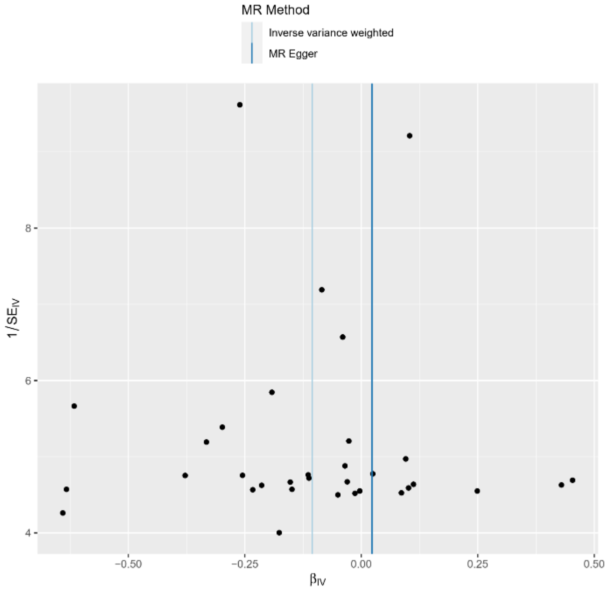

D

Figure S26 Leave-one-out analysis (A), MR effect size (B), scatter plot (C) and funnel plot(D) for Triacylglycerol (56:8) levels on acute pancreatitis

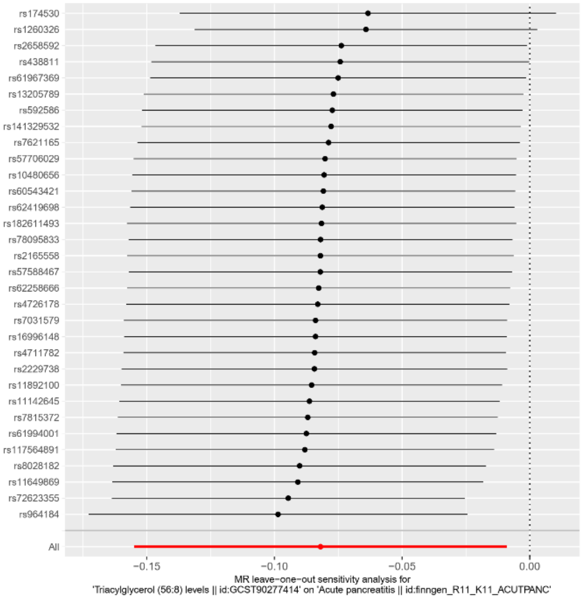

A

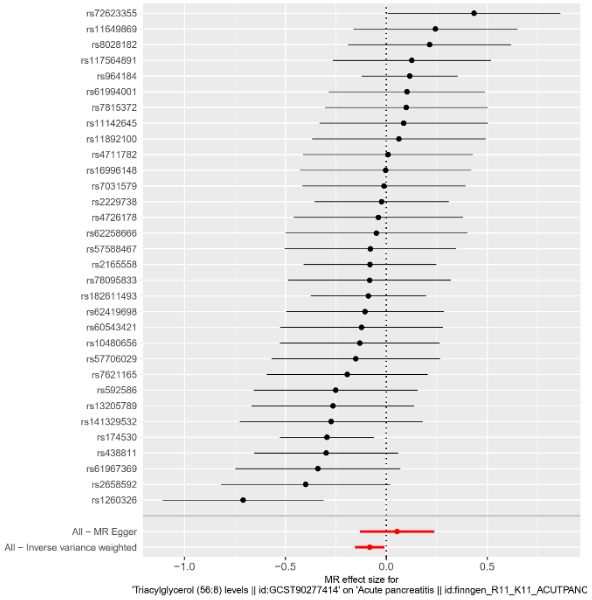

B

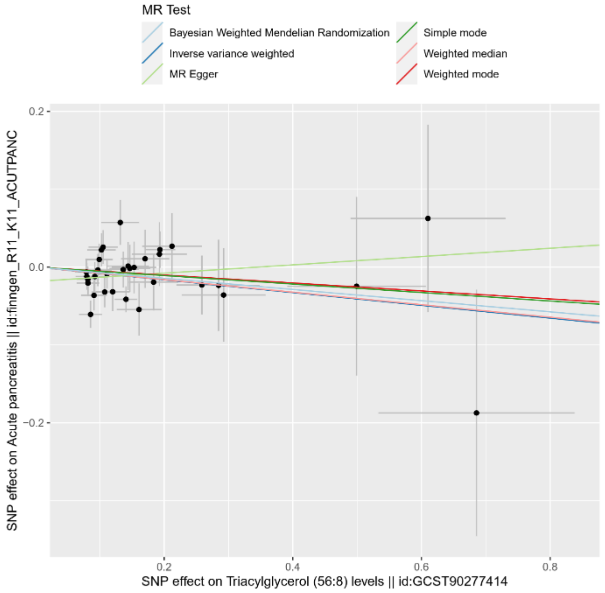

C

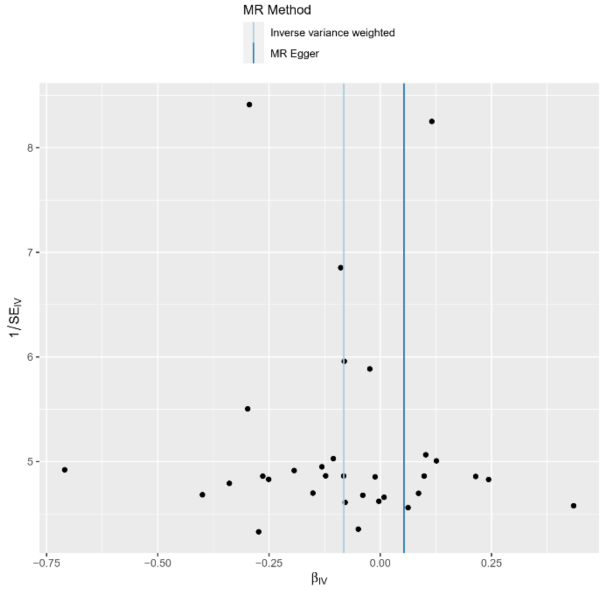

D

Figure S27 Leave-one-out analysis (A), MR effect size (B), scatter plot (C) and funnel plot (D) for Sterol ester (27:1/20:2) levels on acute pancreatitis after eliminating outliers

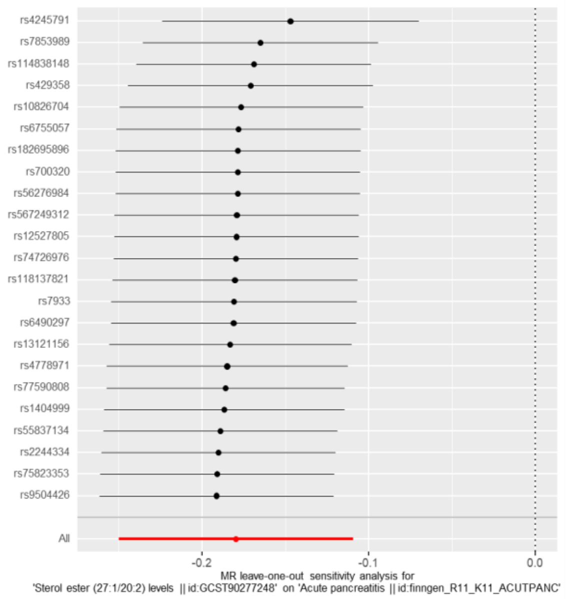

A

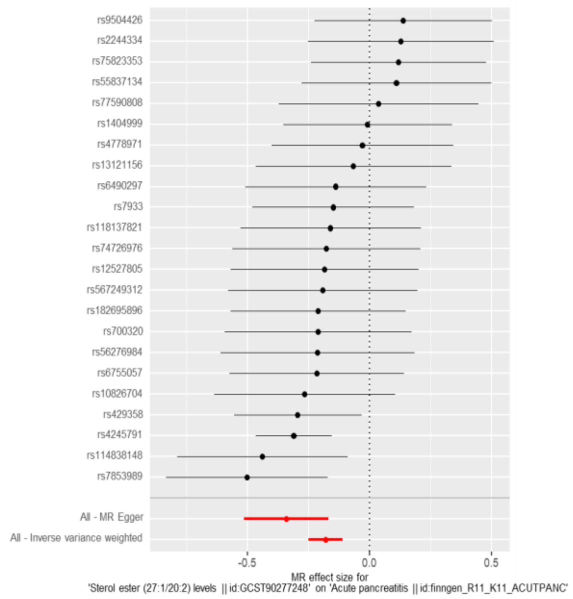

B

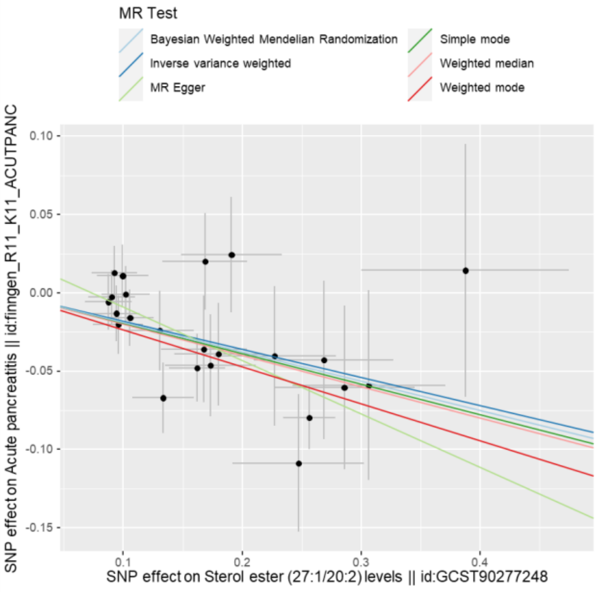

C

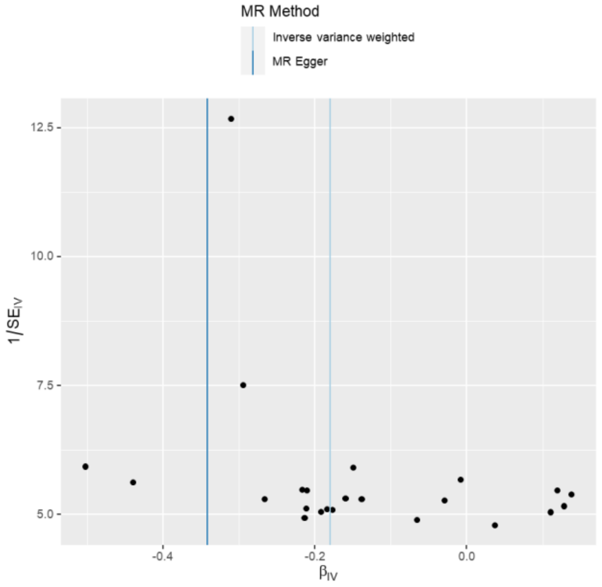

D
