# Supplementary material for: Genetically predicted causal link between the plasma lipidome and pancreatic diseases: a bidirectional Mendelian randomization study
Source: Front Nutr. 2025 Jan 15;11:1466509. doi: 10.3389/fnut.2024.1466509 (PMC11774697; doi:10.3389/fnut.2024.1466509)

Figure S28 Leave-one-out analysis (A), MR effect size (B), scatter plot (C) and funnel plot (D) for Sterol ester (27:1/20:4) levels on chronic pancreatitis

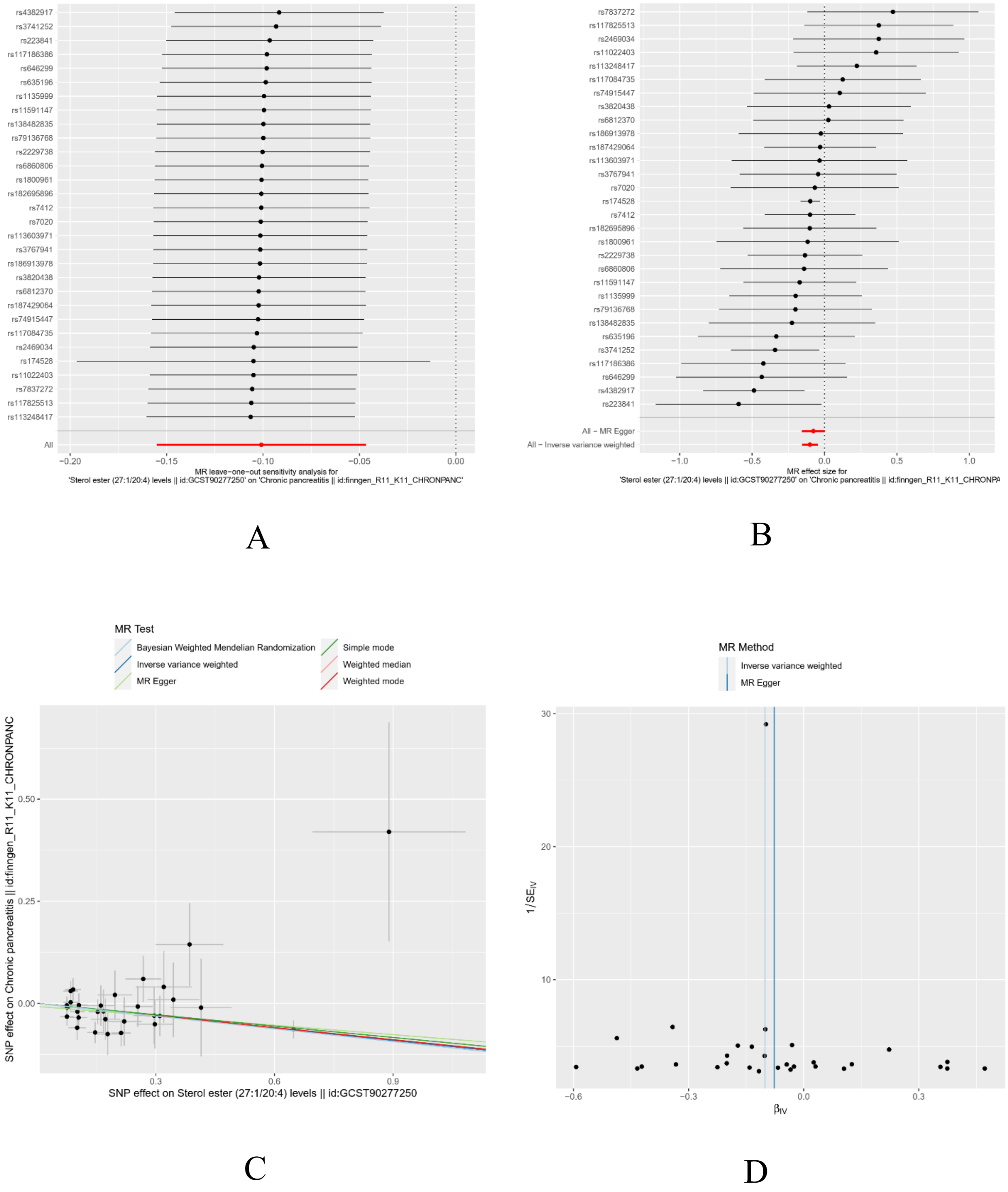

Figure S29 Leave-one-out analysis (A), MR effect size (B), scatter plot (C) and funnel plot (D) for Sterol ester (27:1/20:5) levels on chronic pancreatitis

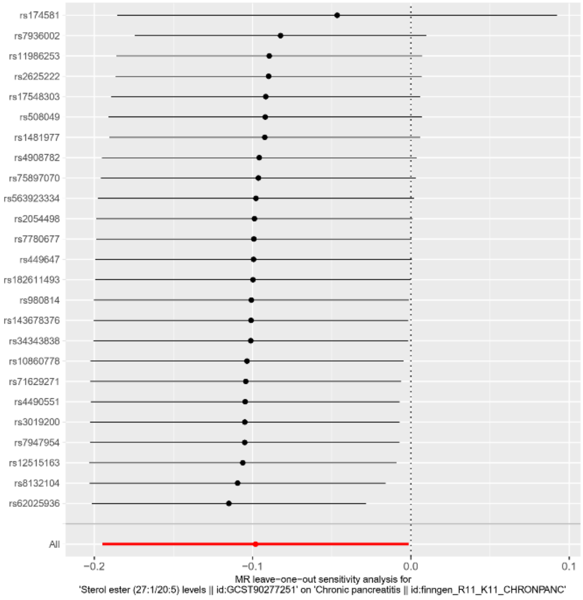

A

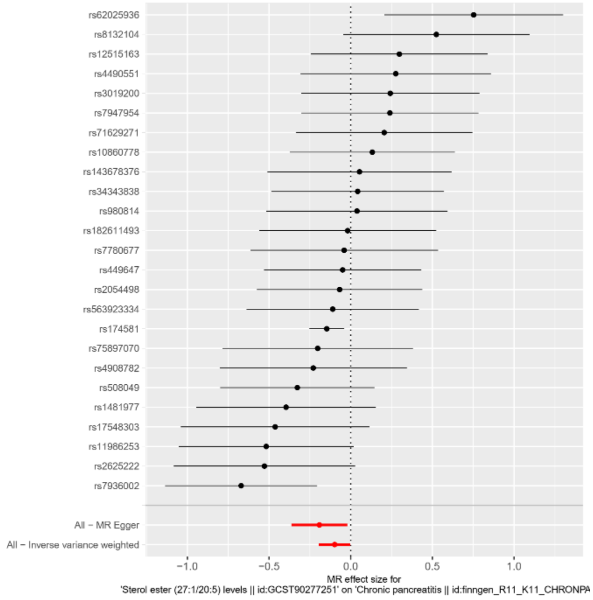

B

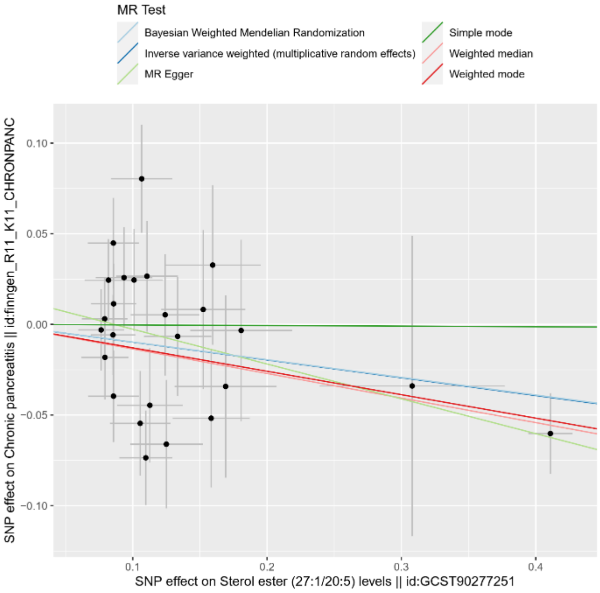

C

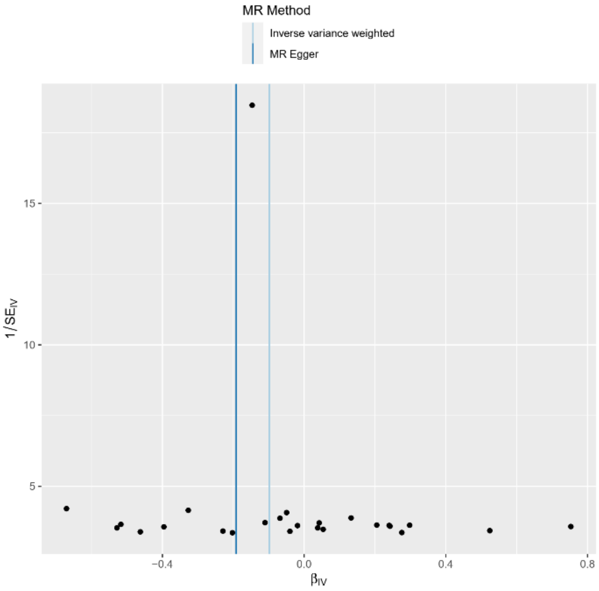

D

Figure S30 Leave-one-out analysis (A), MR effect size (B), scatter plot (C) and funnel plot (D) for Sterol ester (27:1/22:6) levels on chronic pancreatitis

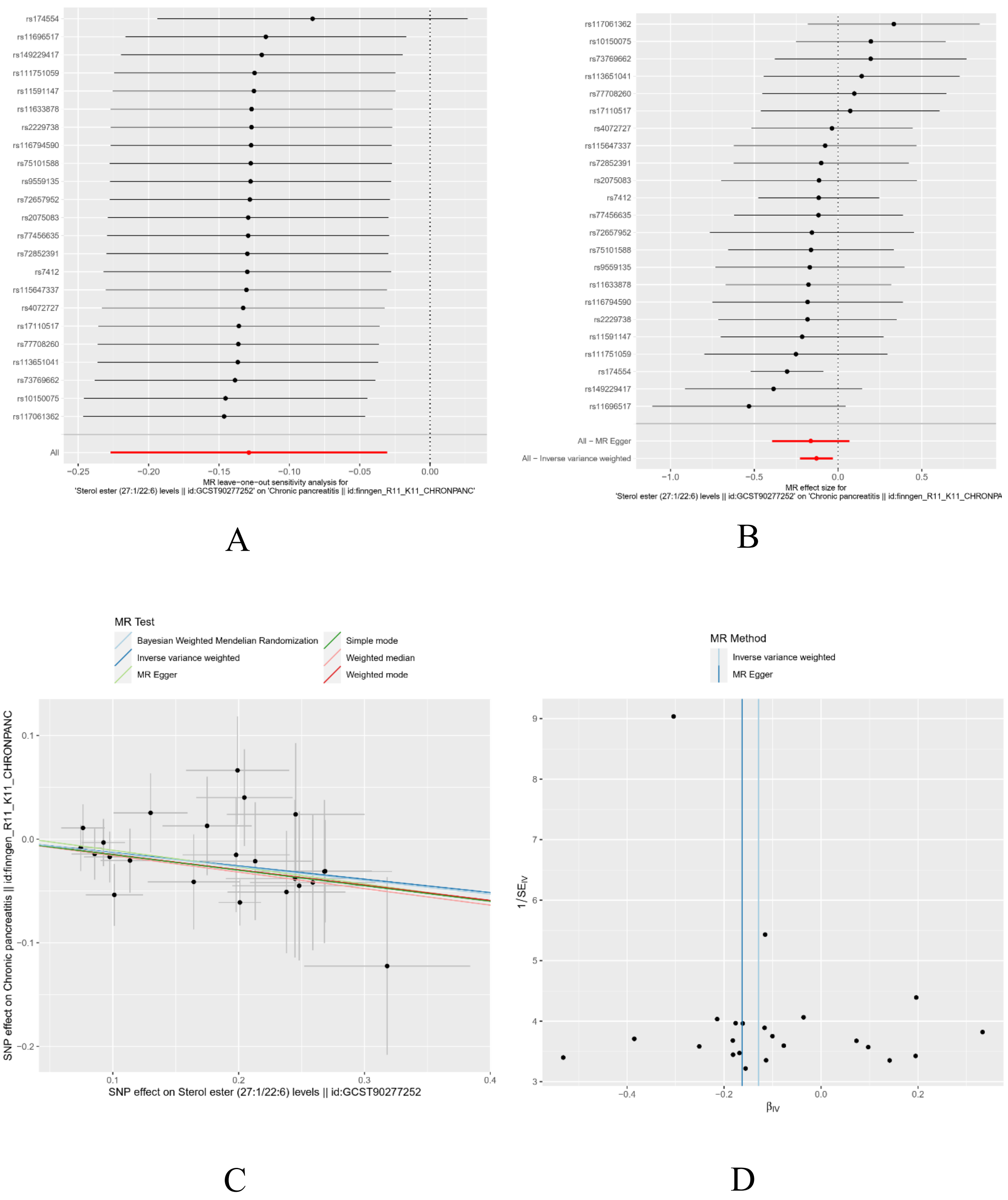

Figure S31 Leave-one-out analysis (A), MR effect size (B), scatter plot (C) and funnel plot (D) for Cholesterol levels on chronic pancreatitis

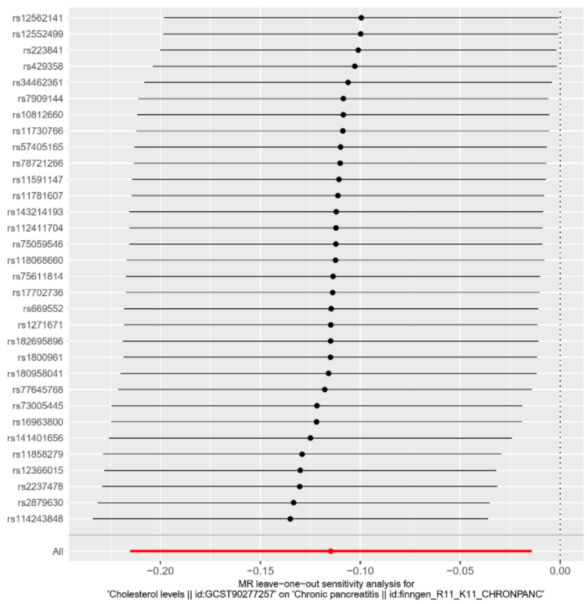

A

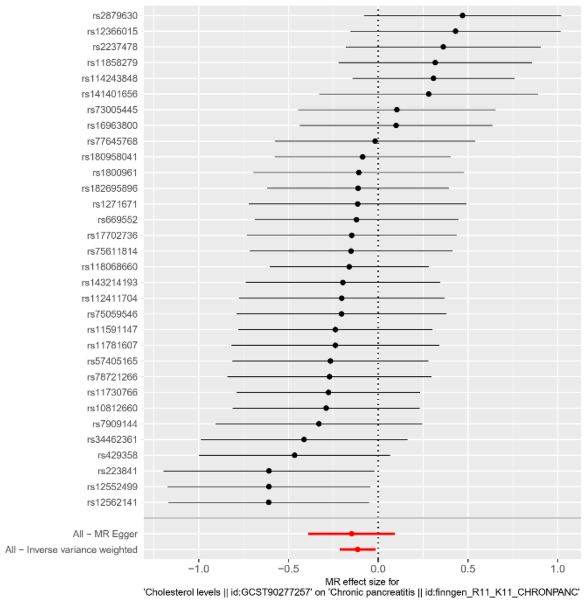

B

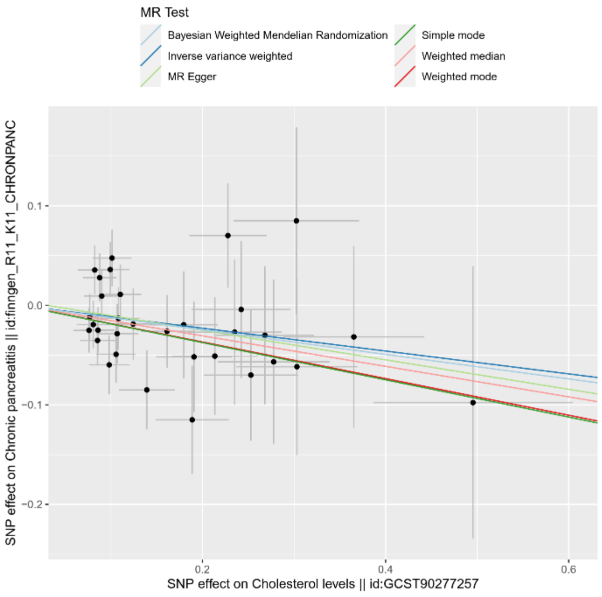

C

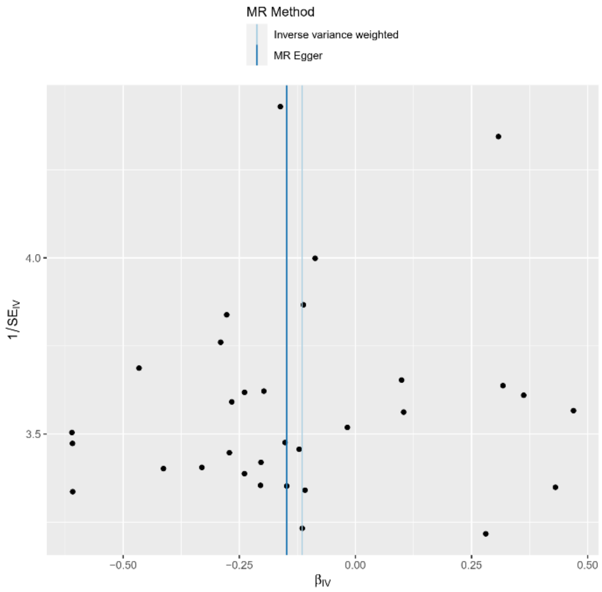

D

Figure S32 Leave-one-out analysis (A), MR effect size (B), scatter plot (C) and funnel plot (D) for Phosphatidylcholine (20:4_0:0) levels on chronic pancreatitis

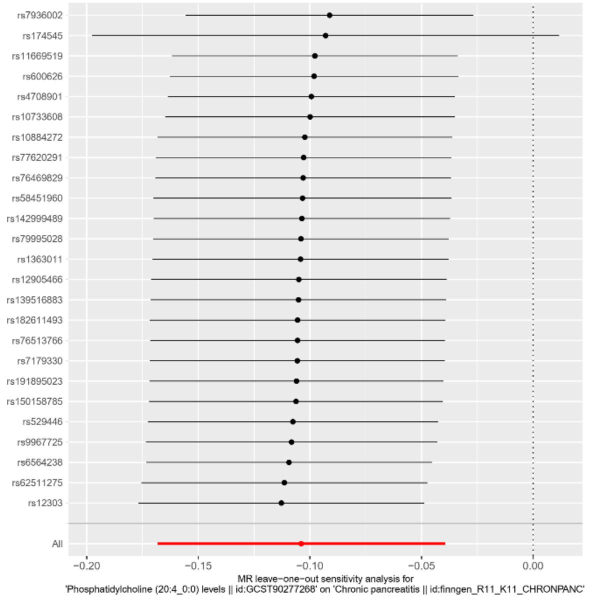

A

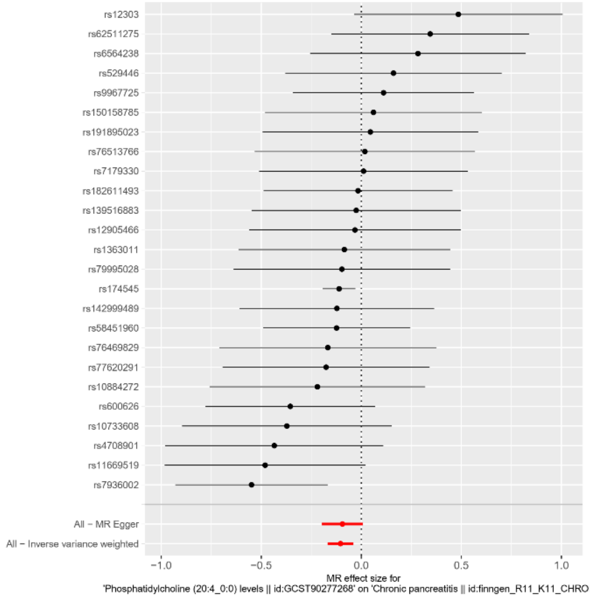

B

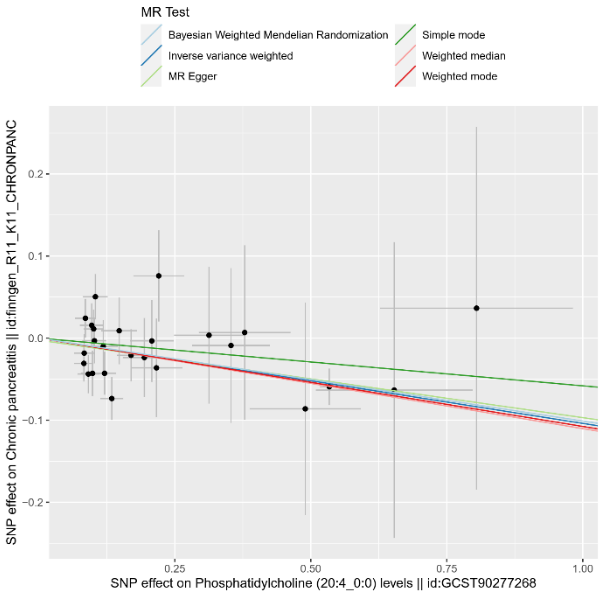

C

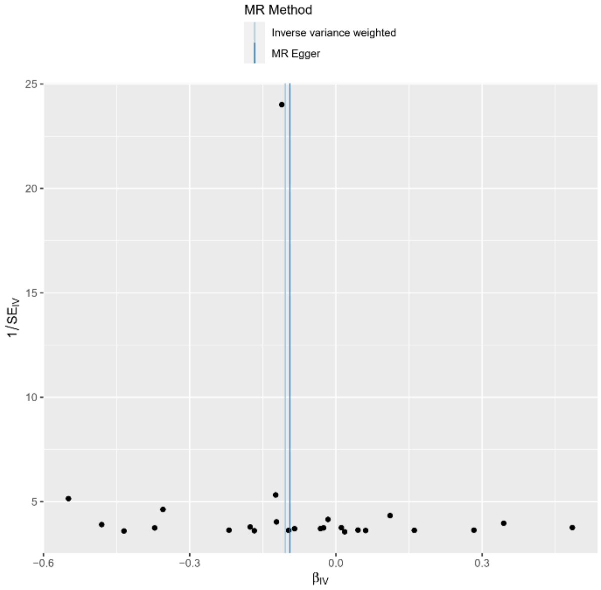

D

Figure S33 Leave-one-out analysis (A), MR effect size (B), scatter plot (C) and funnel plot (D) for Phosphatidylethanolamine (18:2_0:0) levels on chronic pancreatitis

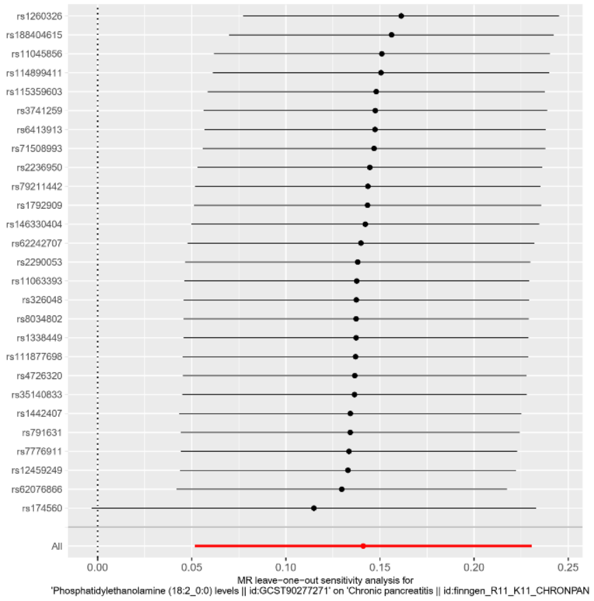

A

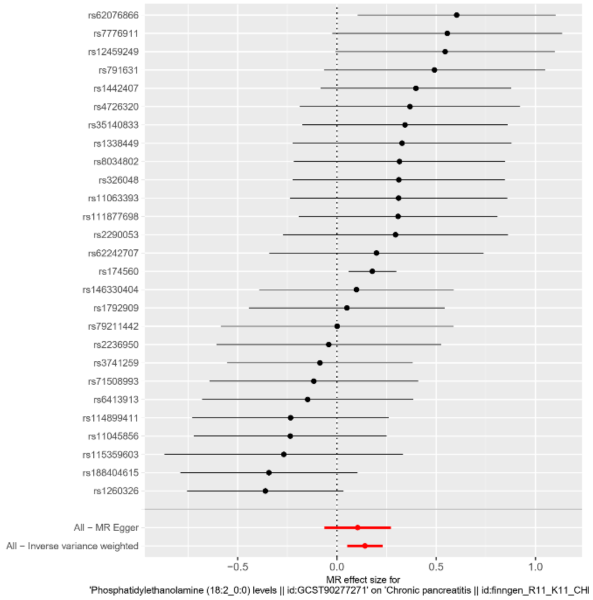

B

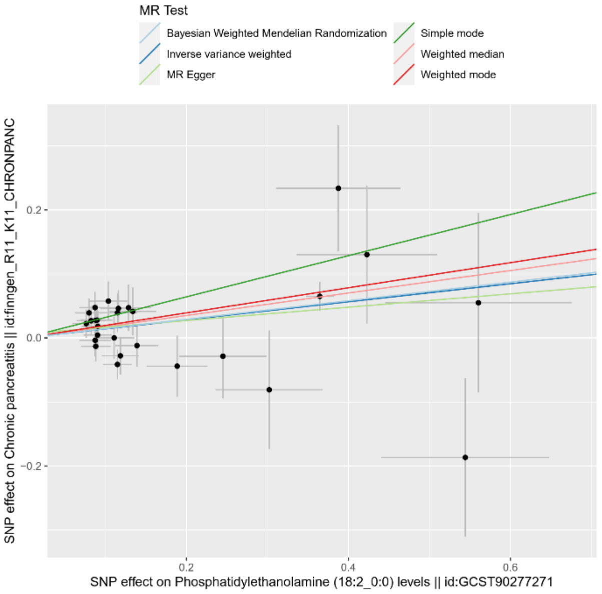

C

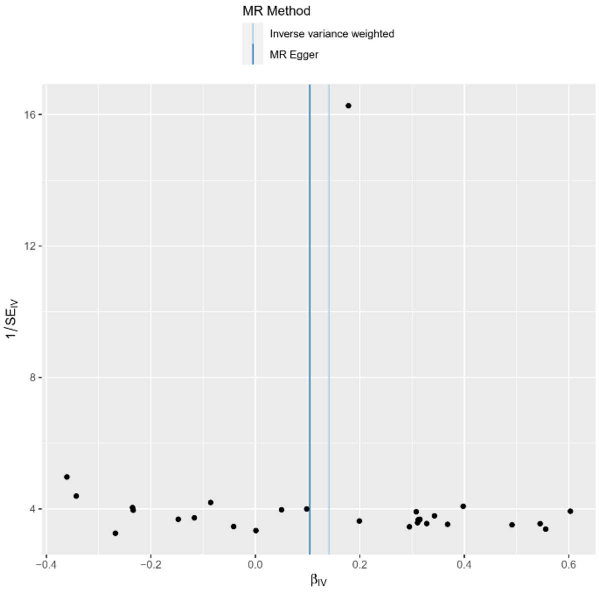

D

Figure S34 Leave-one-out analysis (A), MR effect size (B), scatter plot (C) and funnel plot (D) for Phosphatidylcholine (16:0_20:4) levels on chronic pancreatitis

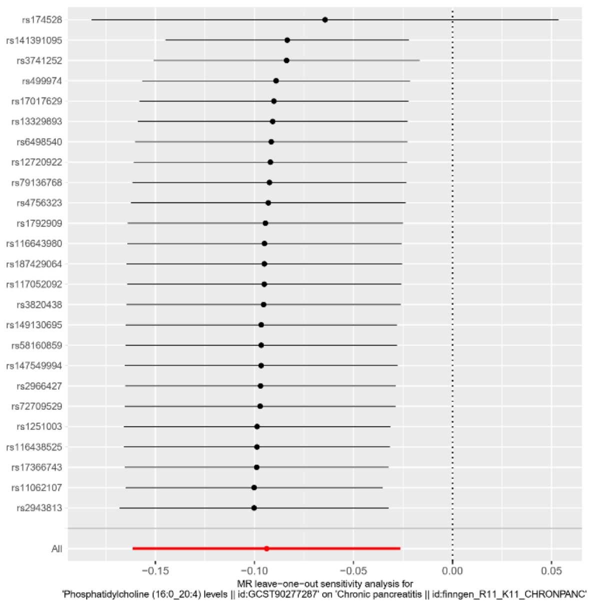

A

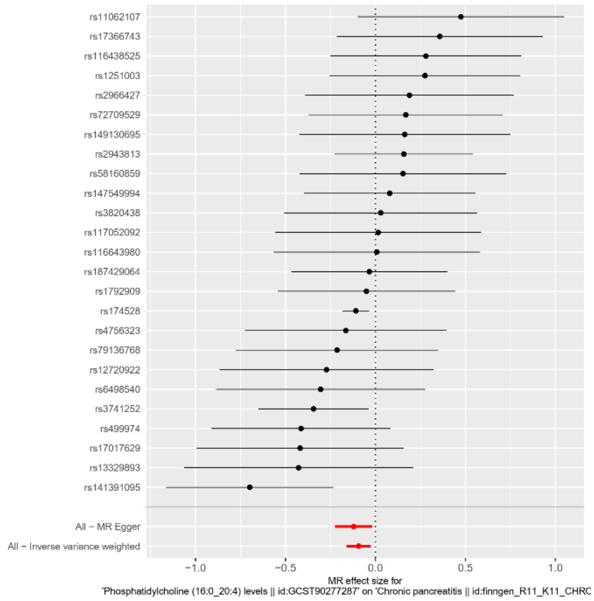

B

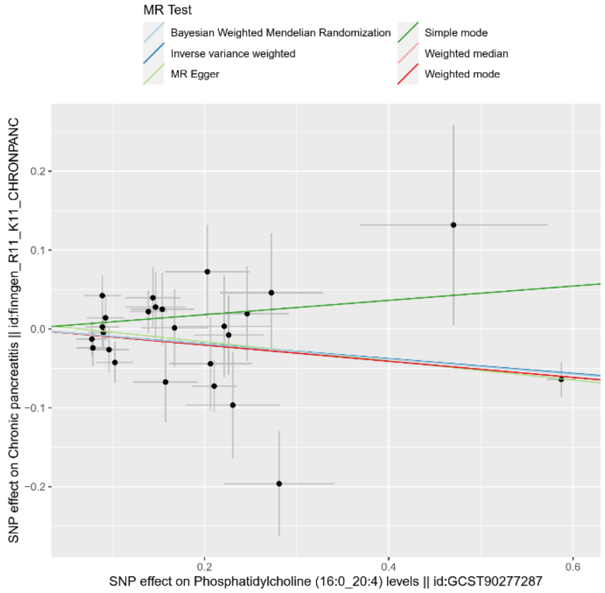

C

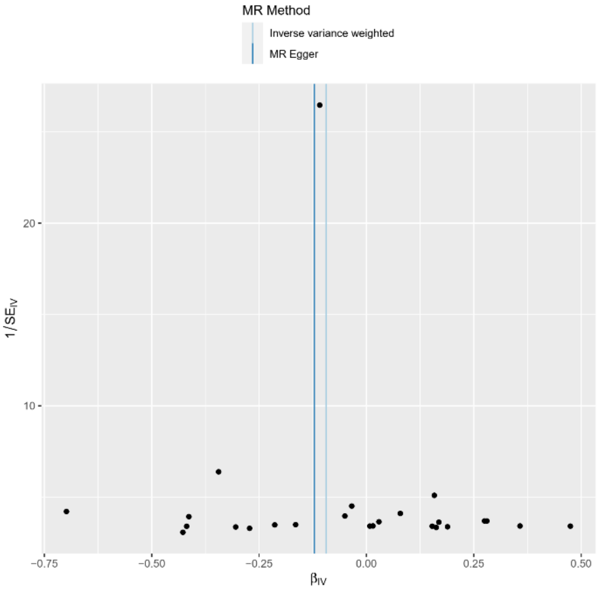

D

Figure S35 Leave-one-out analysis (A), MR effect size (B), scatter plot (C) and funnel plot (D) for Phosphatidylcholine (16:0_22:5) levels on chronic pancreatitis

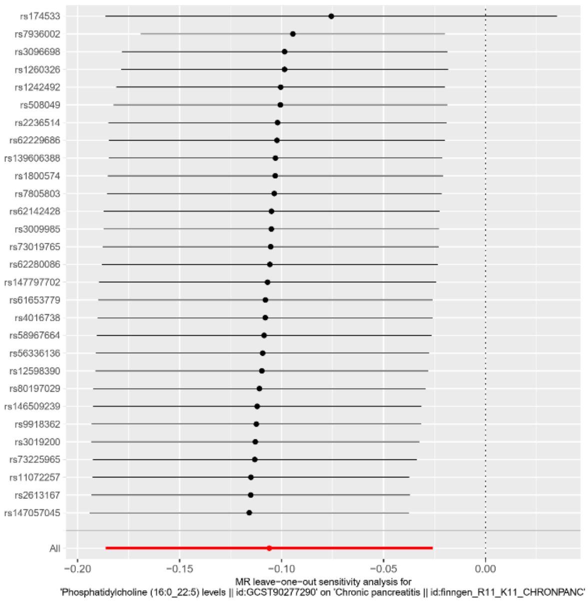

A

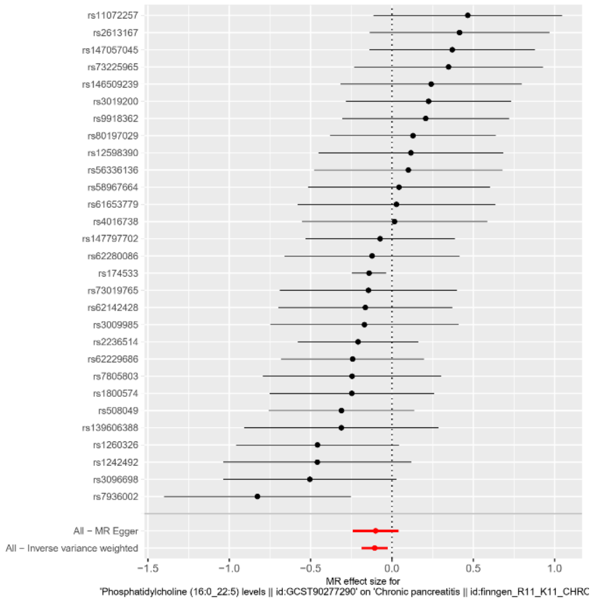

B

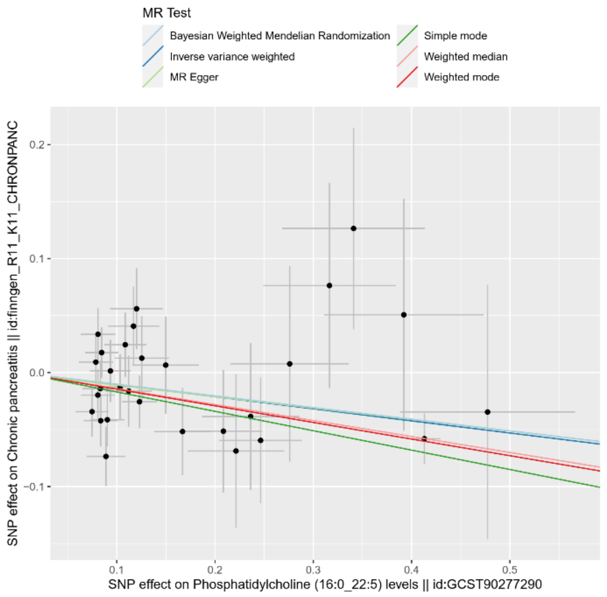

C

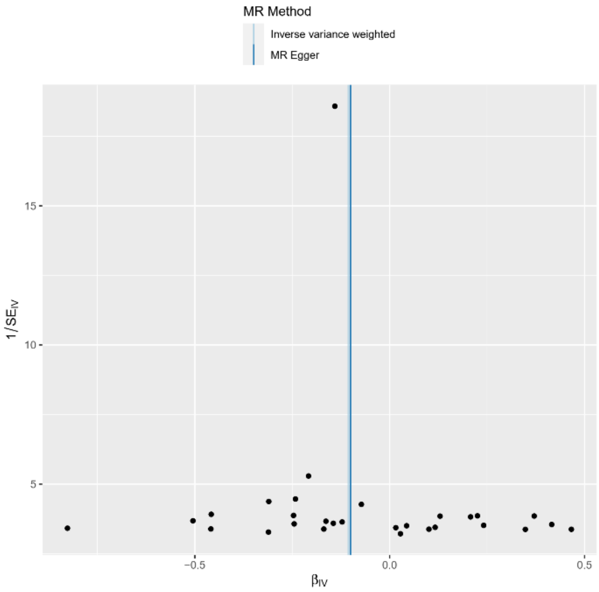

D

Figure S36 Leave-one-out analysis (A), MR effect size (B), scatter plot (C) and funnel plot (D) for Phosphatidylcholine (17:0_18:1) levels on chronic pancreatitis

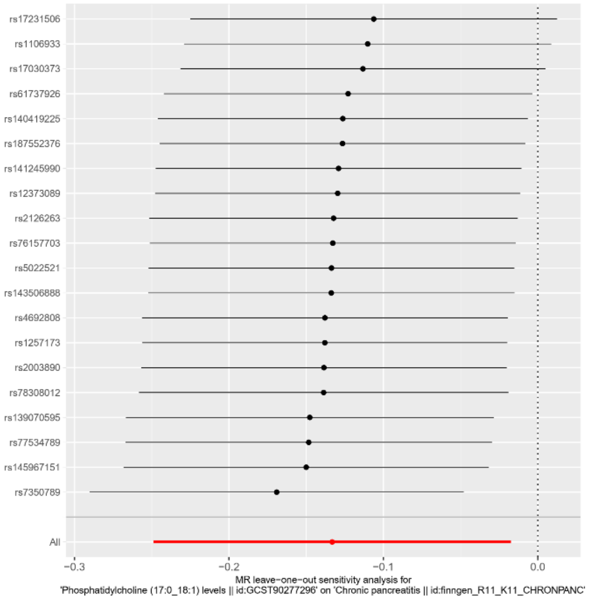

A

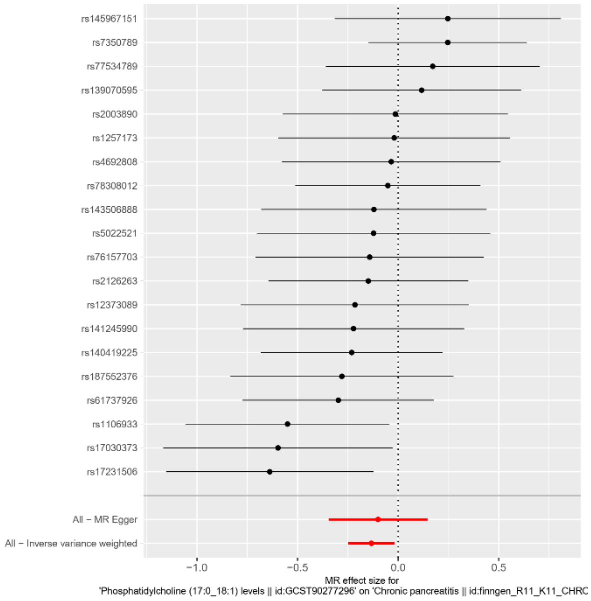

B

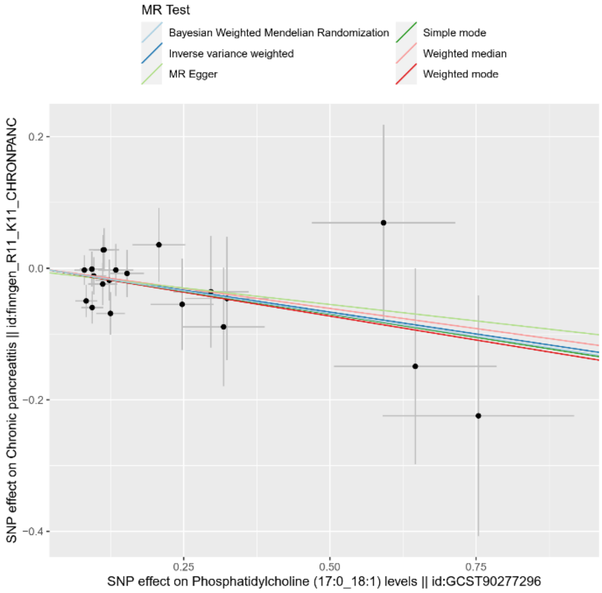

C

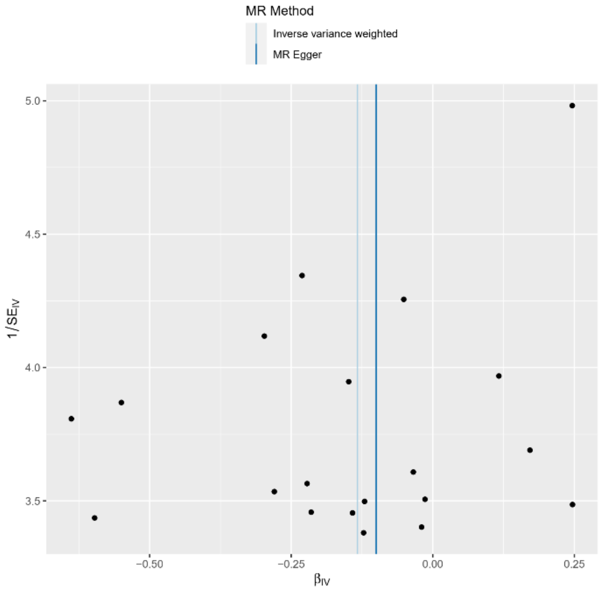

D

Figure S37 Leave-one-out analysis (A), MR effect size (B), scatter plot (C) and funnel plot (D) for Phosphatidylcholine (17:0_18:2) levels on chronic pancreatitis

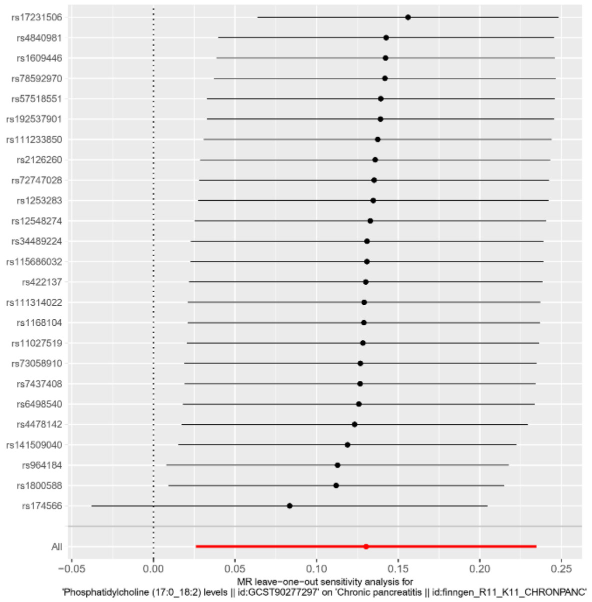

A

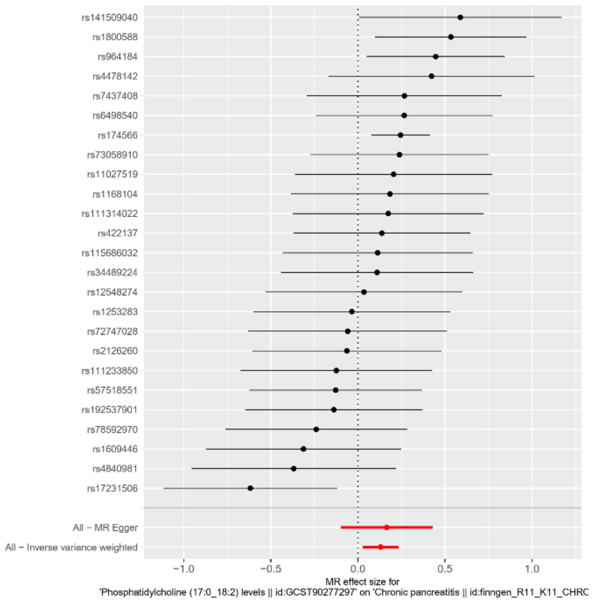

B

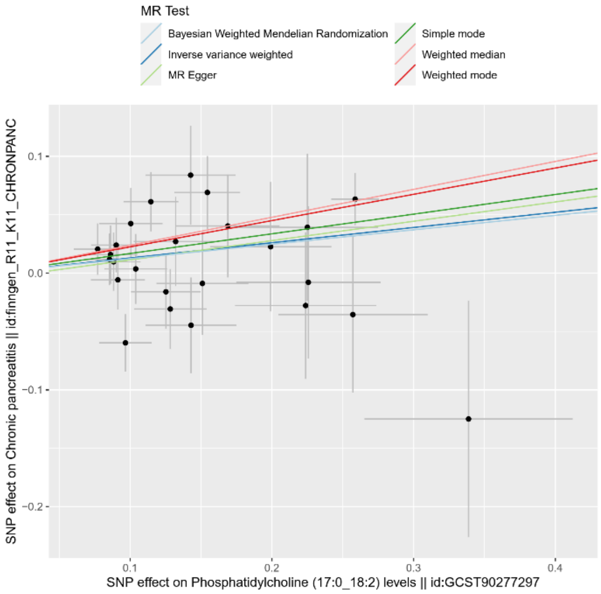

C

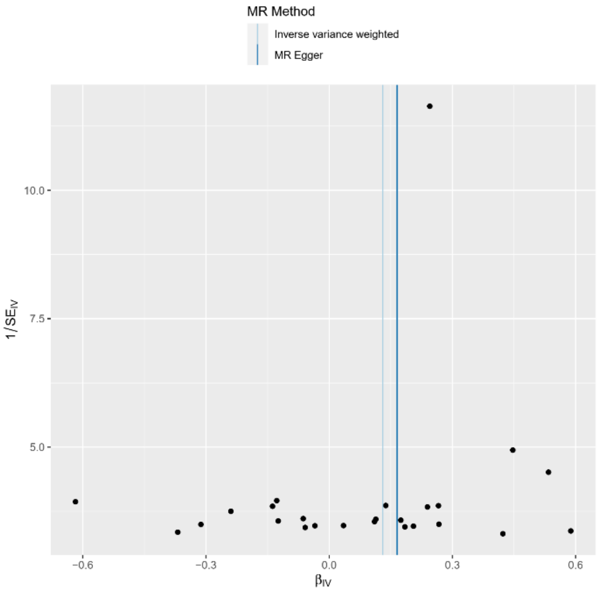

D

Figure S38 Leave-one-out analysis (A), MR effect size (B), scatter plot (C) and funnel plot (D) for Phosphatidylcholine (17:0_20:4) levels on chronic pancreatitis

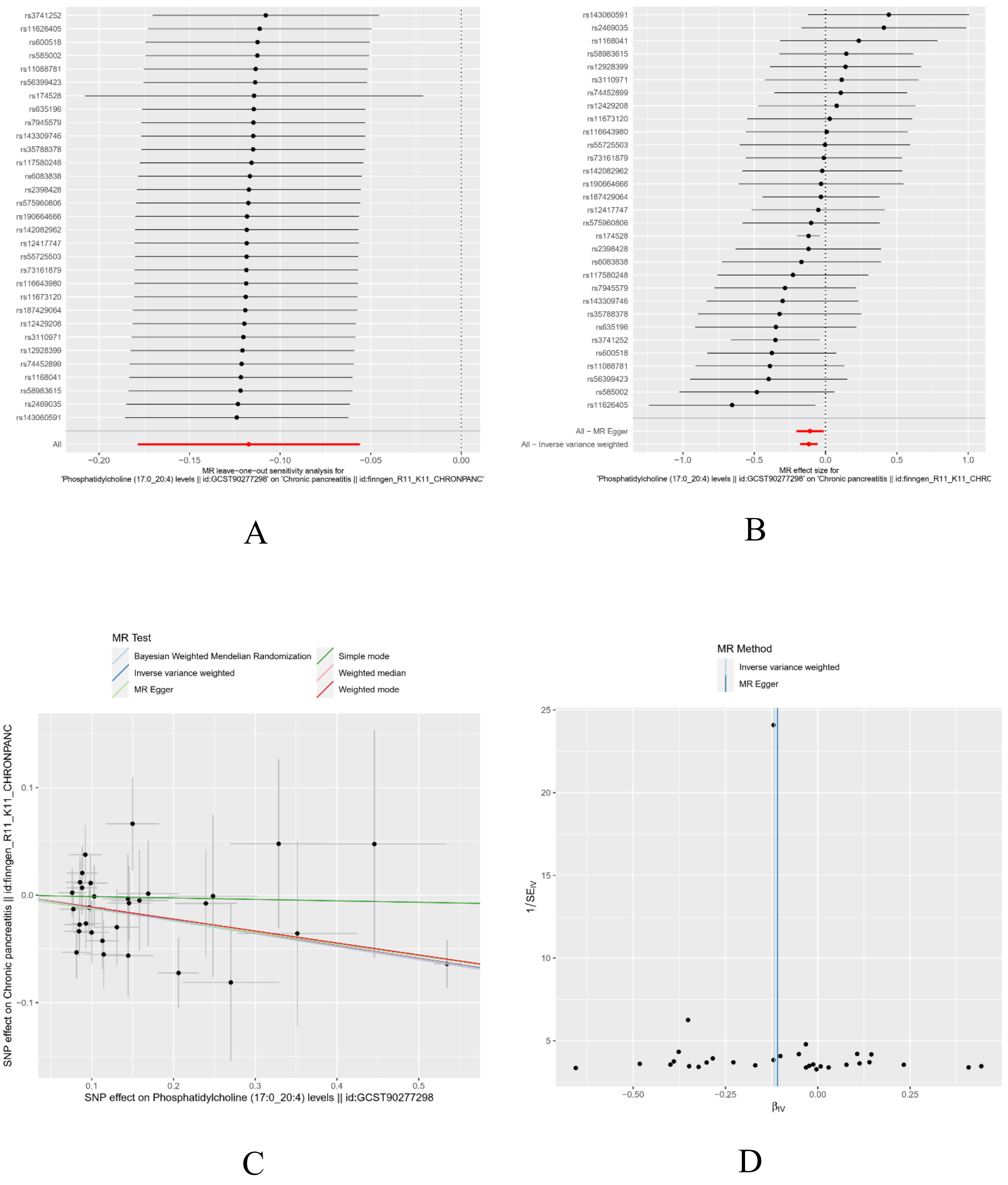

Figure S39 Leave-one-out analysis (A), MR effect size (B), scatter plot (C) and funnel plot (D) for Phosphatidylcholine (18:0_18:2) levels on chronic pancreatitis

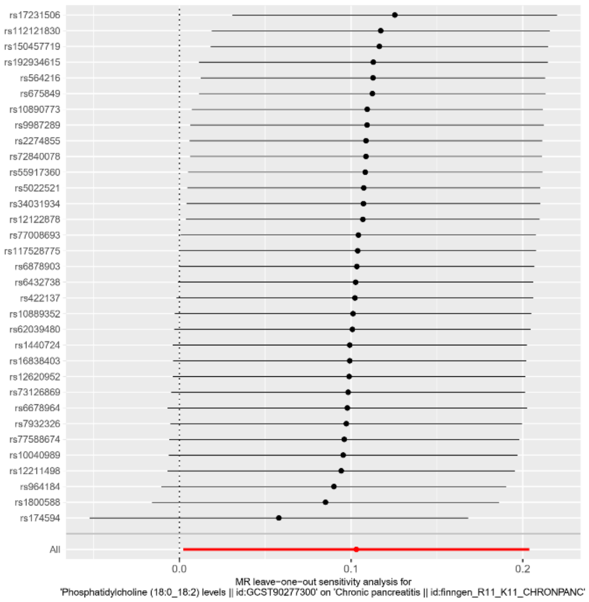

A

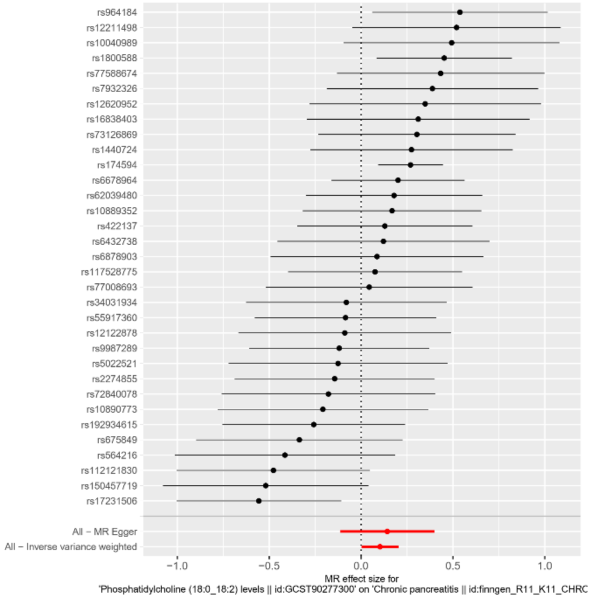

B

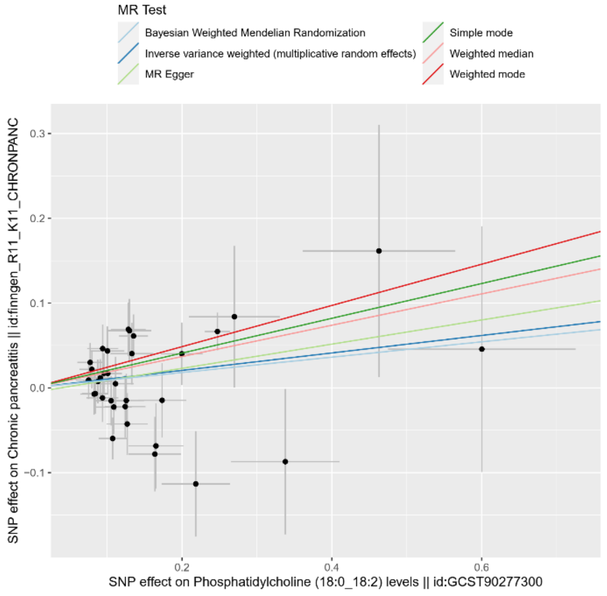

C

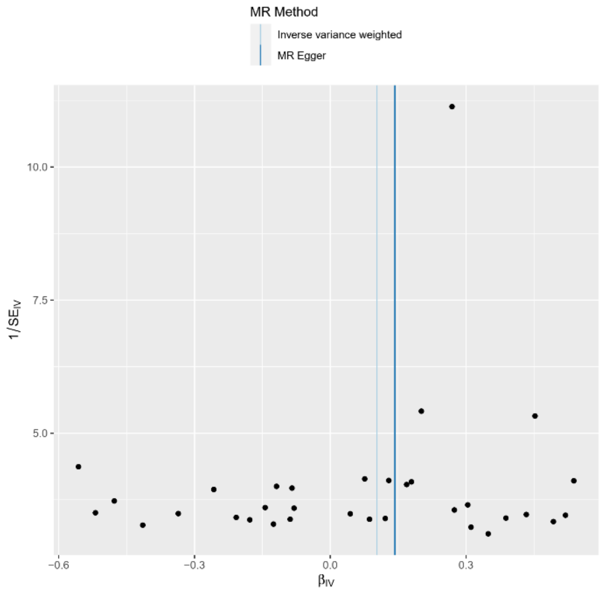

D

Figure S40 Leave-one-out analysis (A), MR effect size (B), scatter plot (C) and funnel plot (D) for Phosphatidylcholine (18:0_20:4) levels on chronic pancreatitis

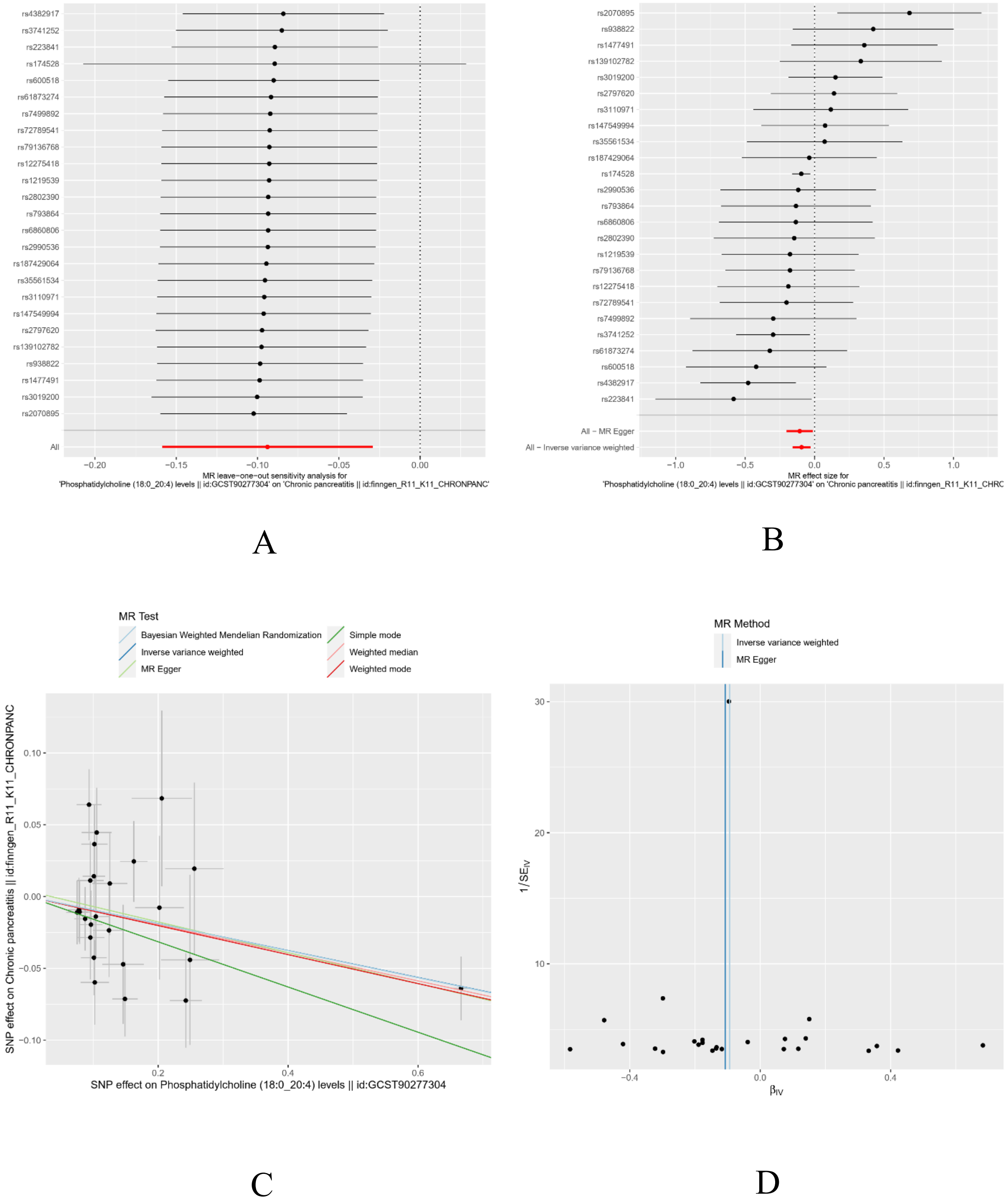

Figure S41 Leave-one-out analysis (A), MR effect size (B), scatter plot (C) and funnel plot (D) for Phosphatidylcholine (18:1_18:2) levels on chronic pancreatitis

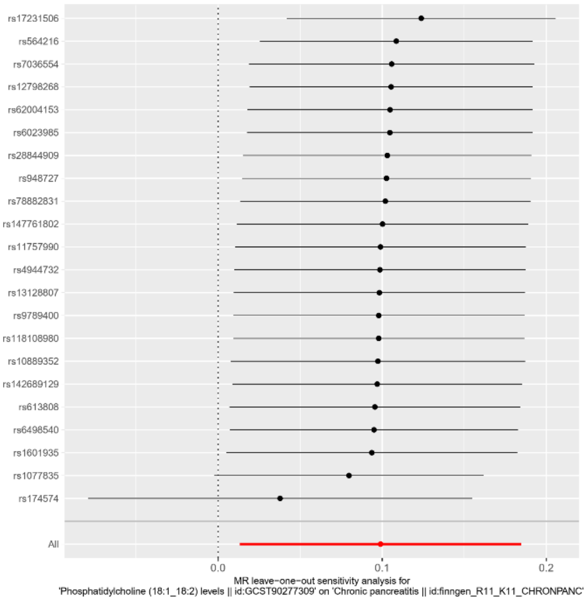

A

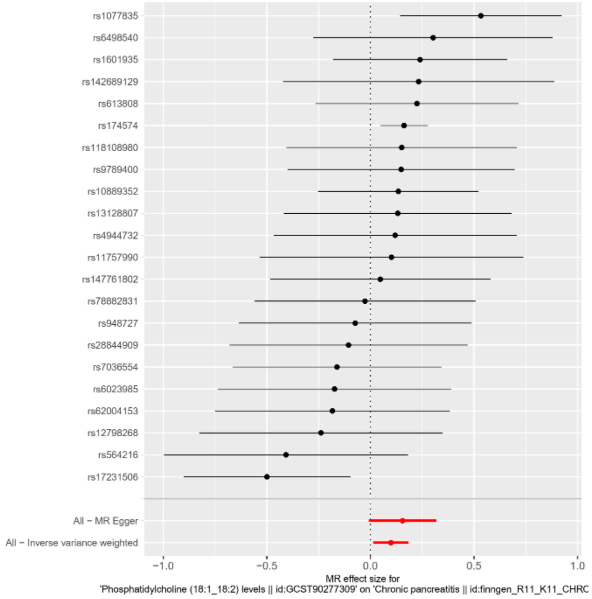

B

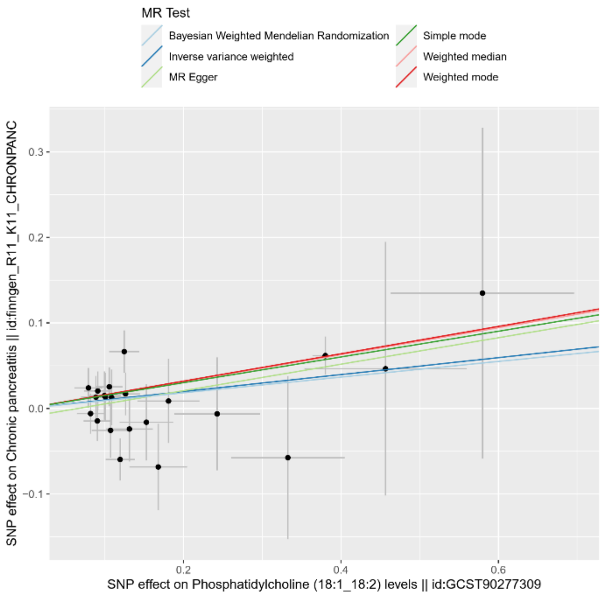

C

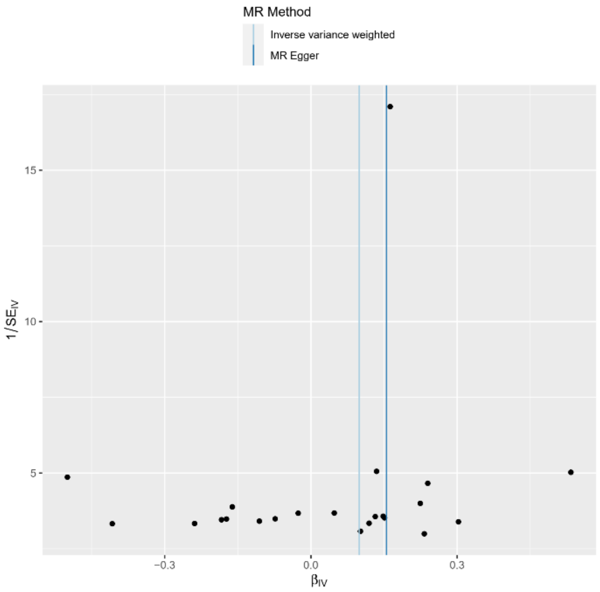

D

Figure S42 Leave-one-out analysis (A), MR effect size (B), scatter plot (C) and funnel plot (D) for Phosphatidylcholine (18:1_20:4) levels on chronic pancreatitis

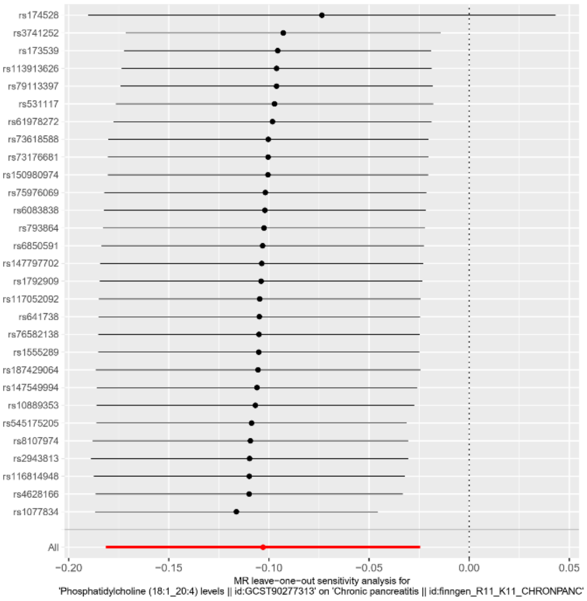

A

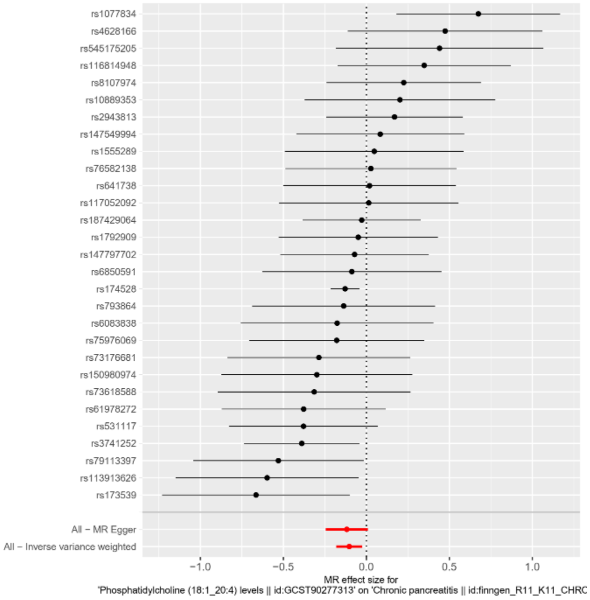

B

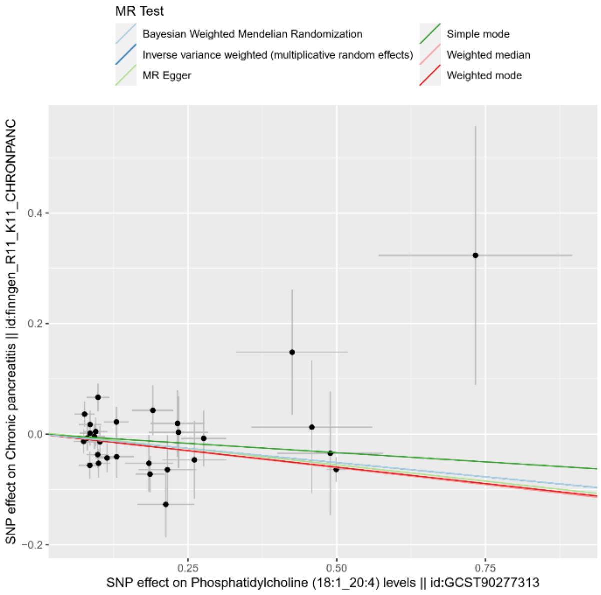

C

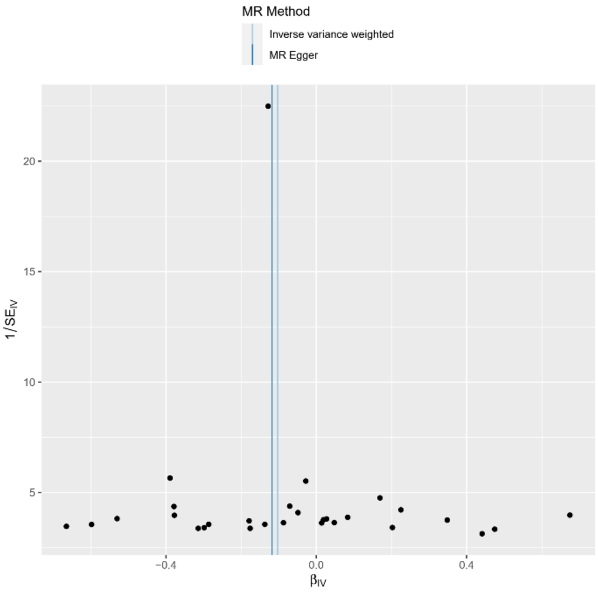

D

Figure S43 Leave-one-out analysis (A), MR effect size (B), scatter plot (C) and funnel plot (D) for Phosphatidylcholine (O-16:0_20:4) levels on chronic pancreatitis

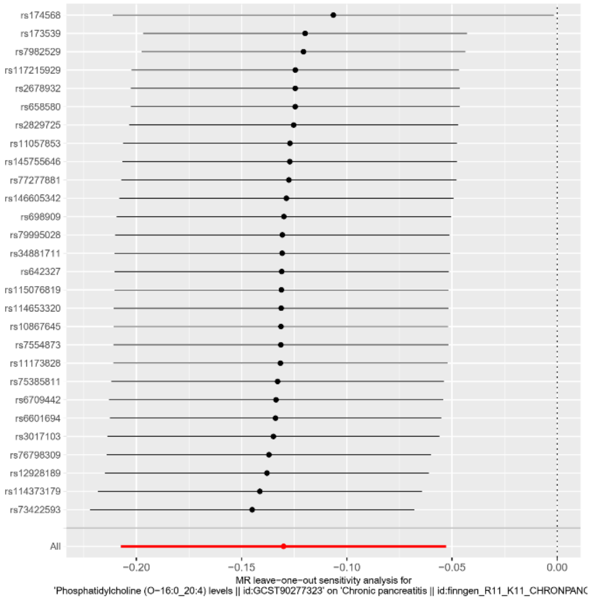

A

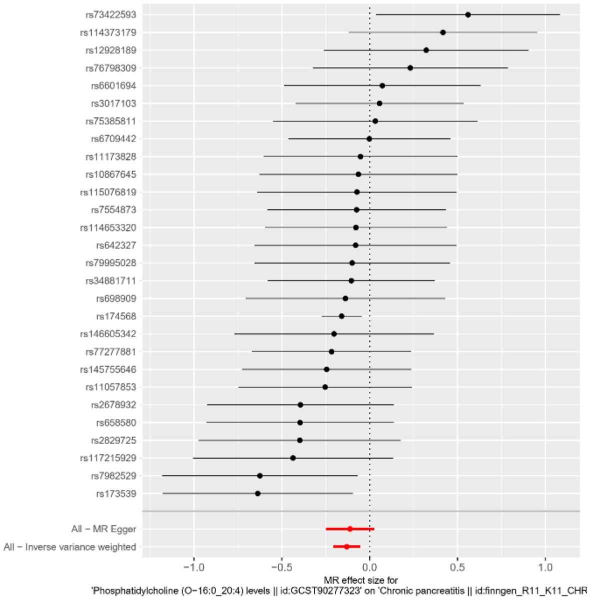

B

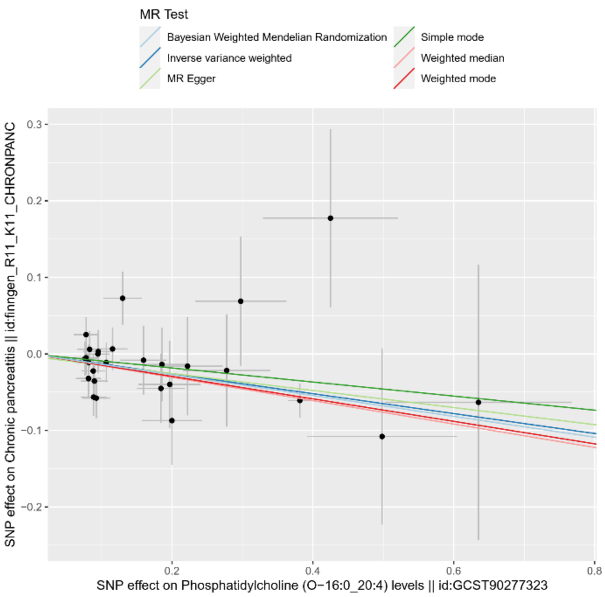

C

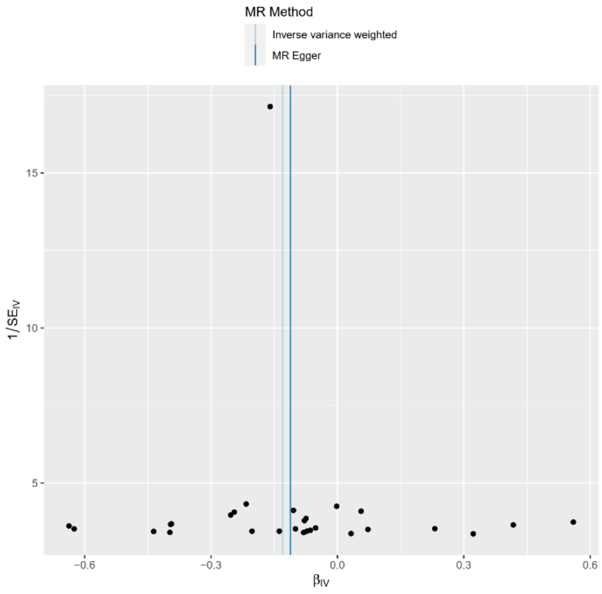

D

Figure S44 Leave-one-out analysis (A), MR effect size (B), scatter plot (C) and funnel plot (D) for Phosphatidylcholine (O-16:1_20:4) levels on chronic pancreatitis

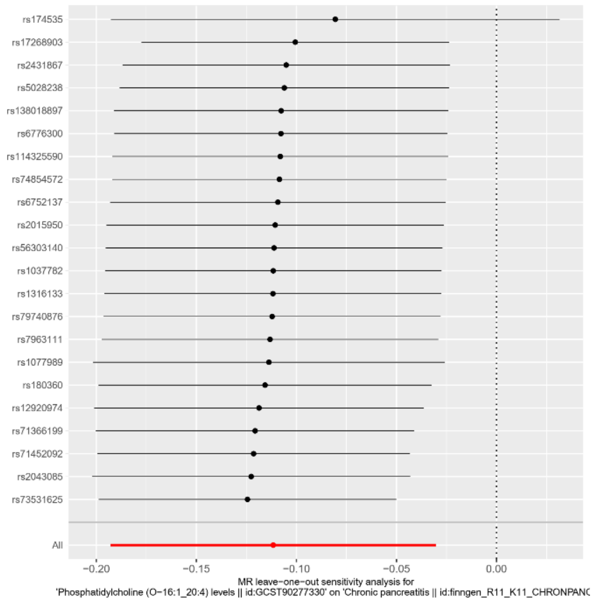

A

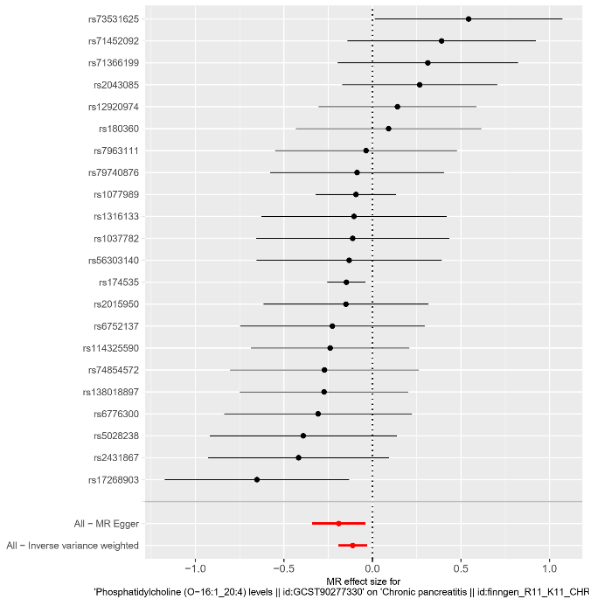

B

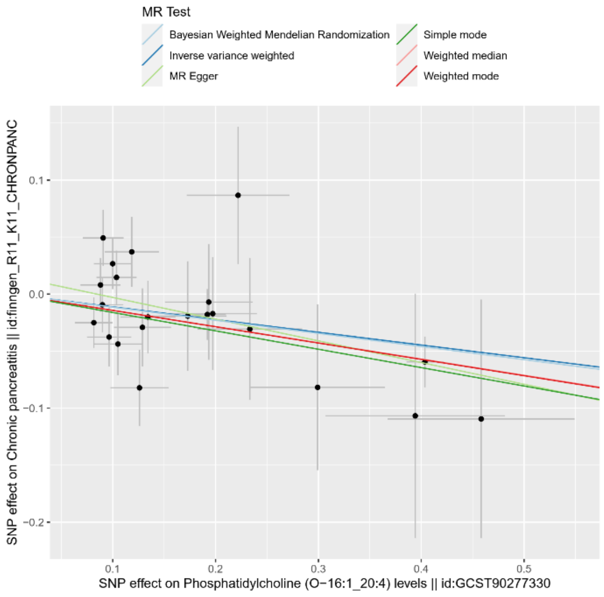

C

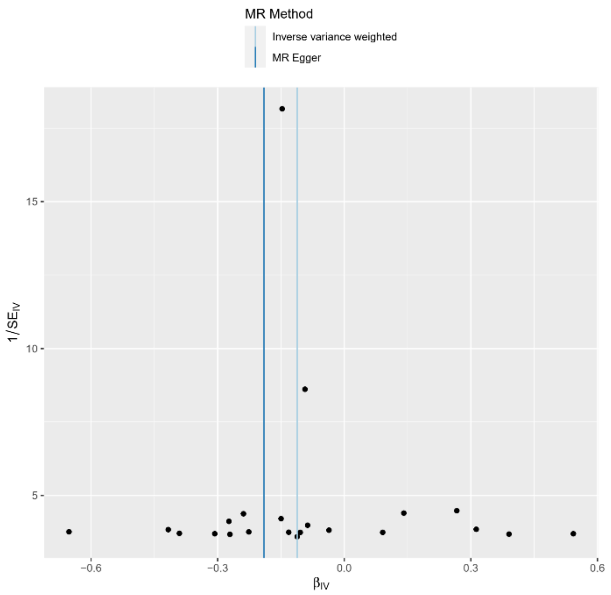

D

Figure S45 Leave-one-out analysis (A), MR effect size (B), scatter plot (C) and funnel plot (D) for Phosphatidylcholine (O-18:0_20:4) levels on chronic pancreatitis

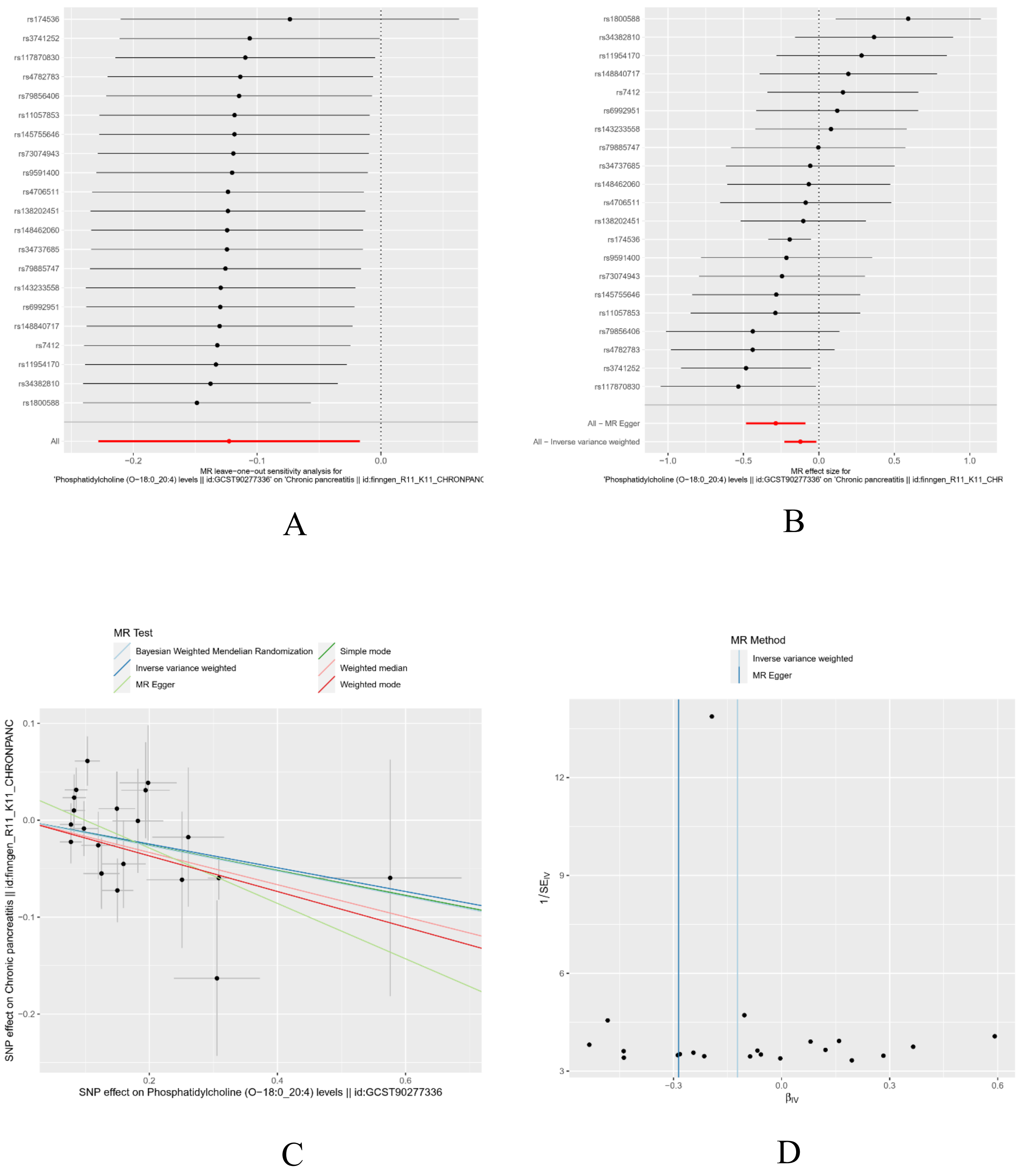

Figure S46 Leave-one-out analysis (A), MR effect size (B), scatter plot (C) and funnel plot (D) for Phosphatidylcholine (O-18:2_18:1) levels on chronic pancreatitis

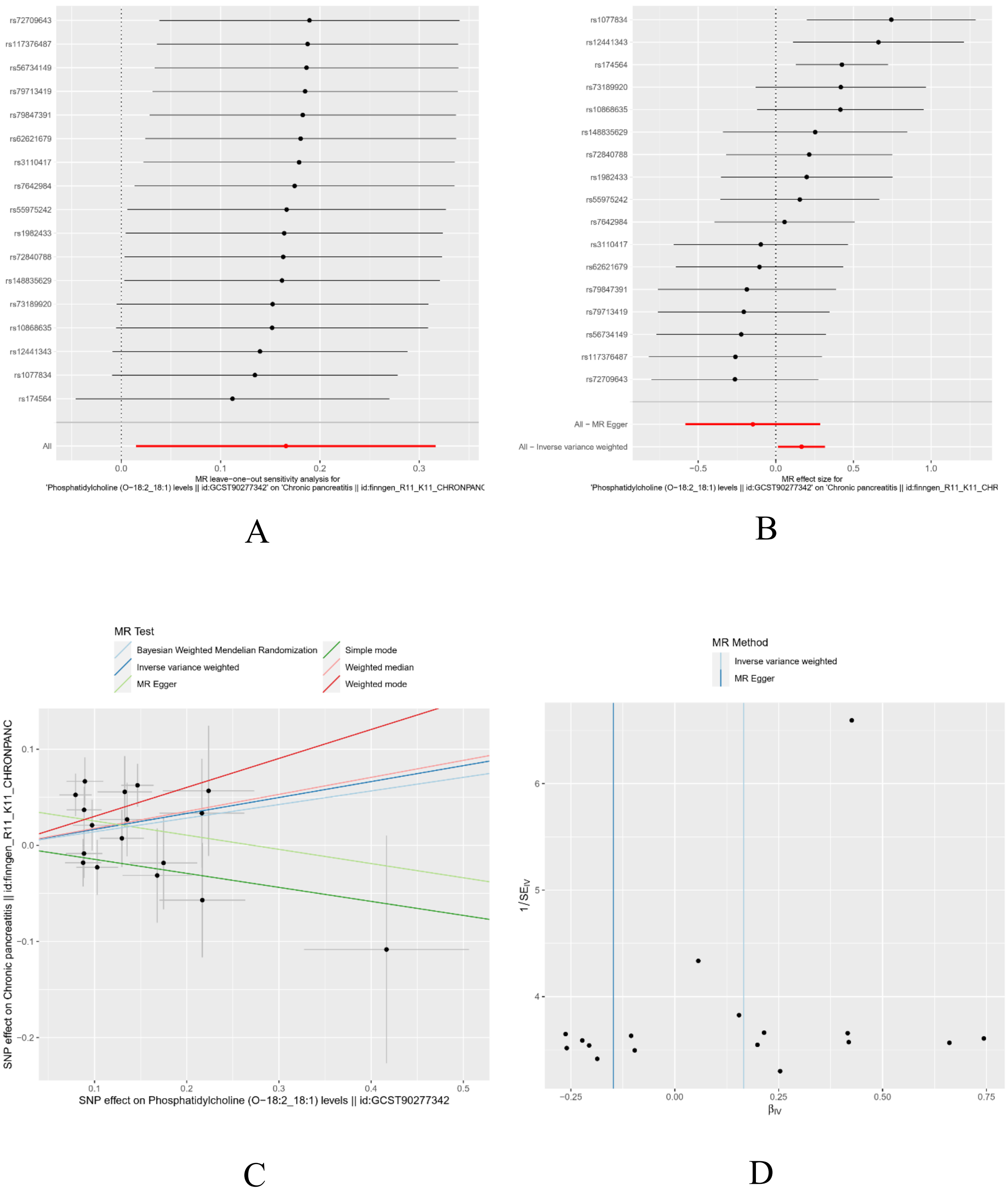

Figure S47 Leave-one-out analysis (A), MR effect size (B), scatter plot (C) and funnel plot (D) for Phosphatidylcholine (O-18:2_20:4) levels on chronic pancreatitis

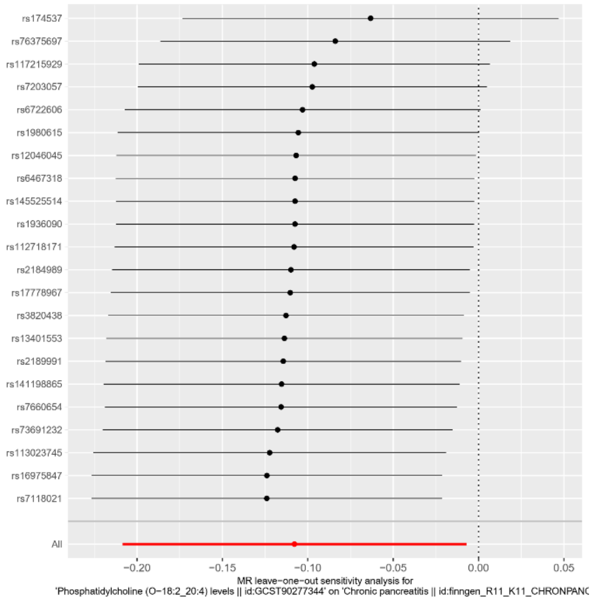

A

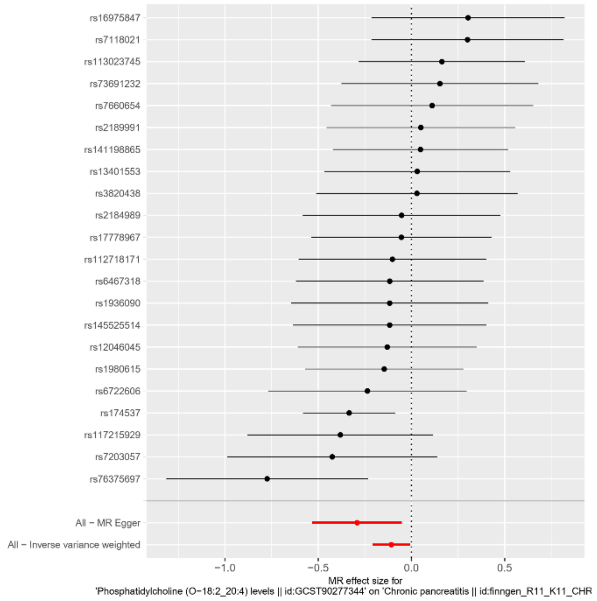

B

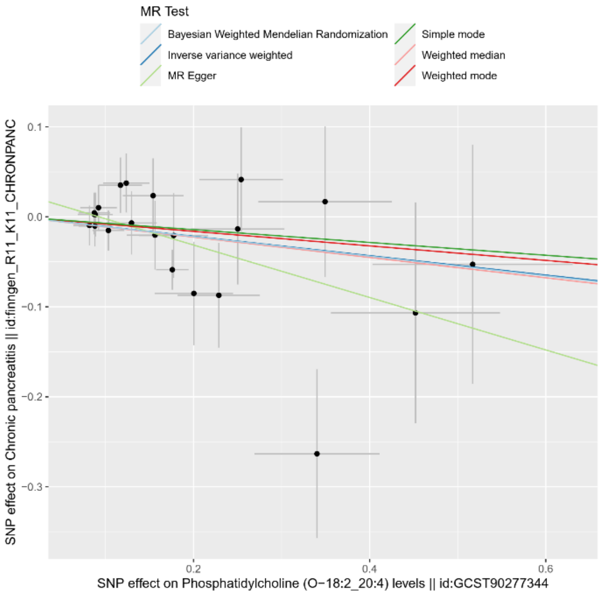

C

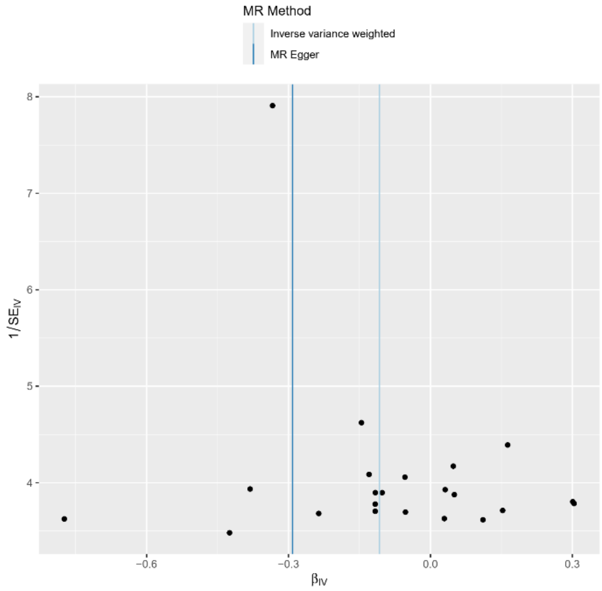

D

Figure S48 Leave-one-out analysis (A), MR effect size (B), scatter plot (C) and funnel plot (D) for Phosphatidylethanolamine (16:0_18:2) levels on chronic pancreatitis

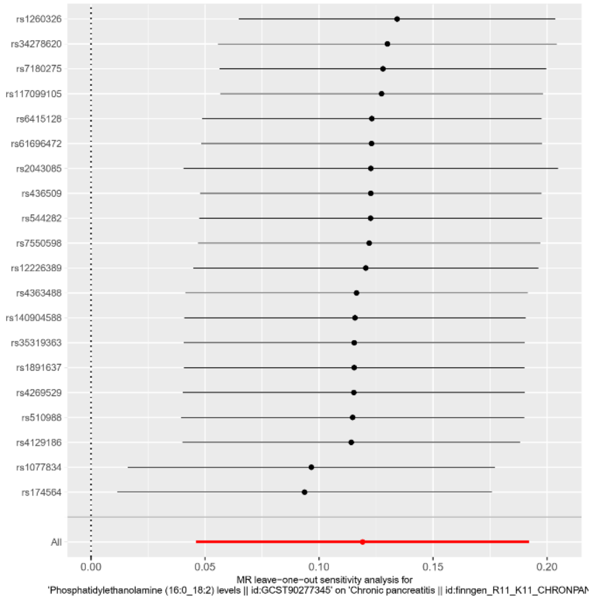

A

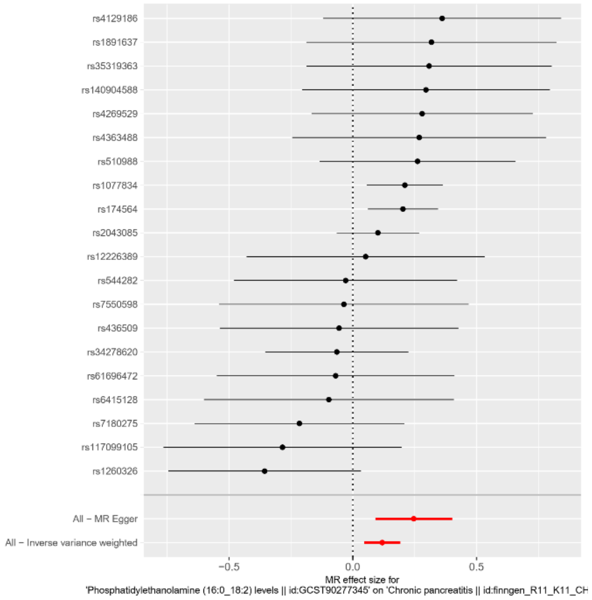

B

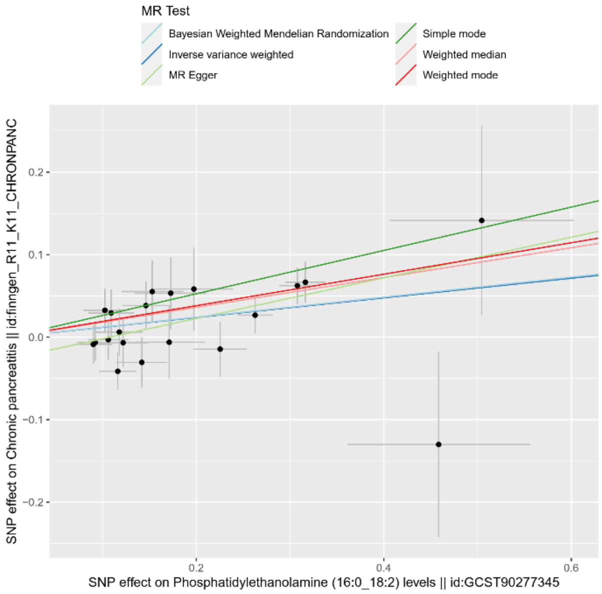

C

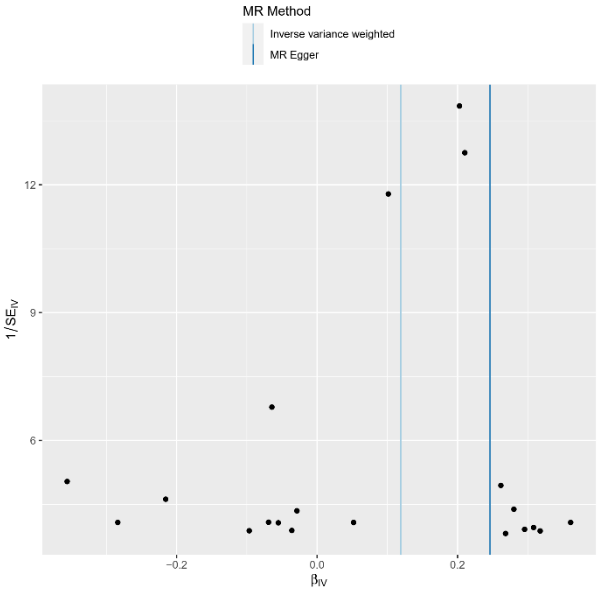

D

Figure S49 Leave-one-out analysis (A), MR effect size (B), scatter plot (C) and funnel plot (D) for Phosphatidylethanolamine (18:1_18:1) levels on chronic pancreatitis

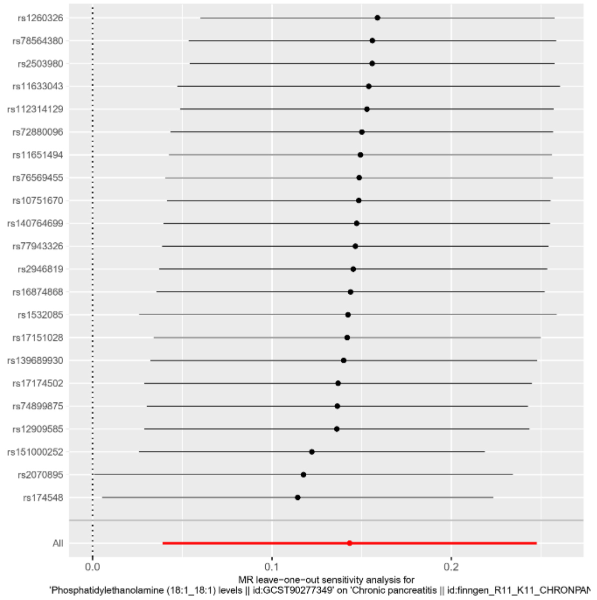

A

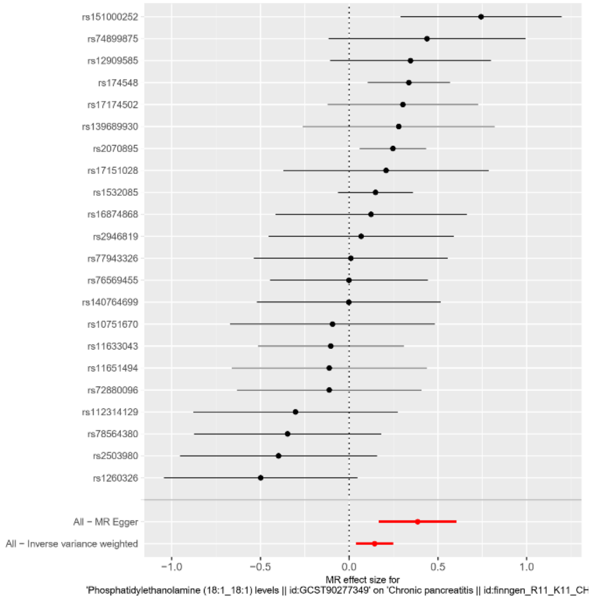

B

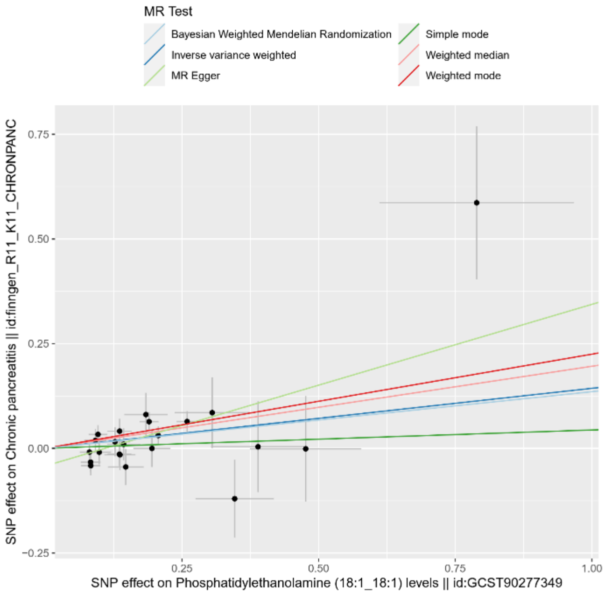

C

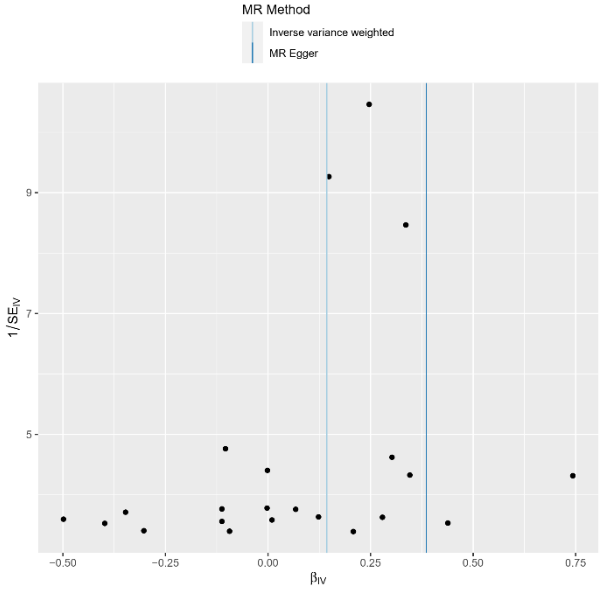

D

Figure S50 Leave-one-out analysis (A), MR effect size (B), scatter plot (C) and funnel plot (D) for Phosphatidylethanolamine (O-18:1_20:4) levels on chronic pancreatitis

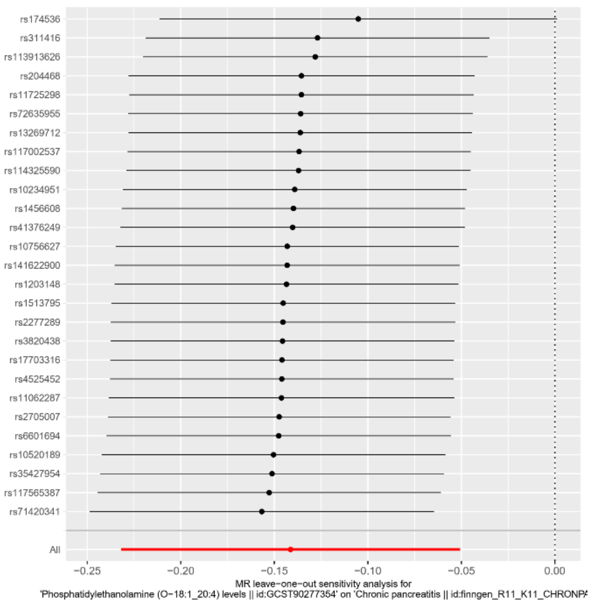

A

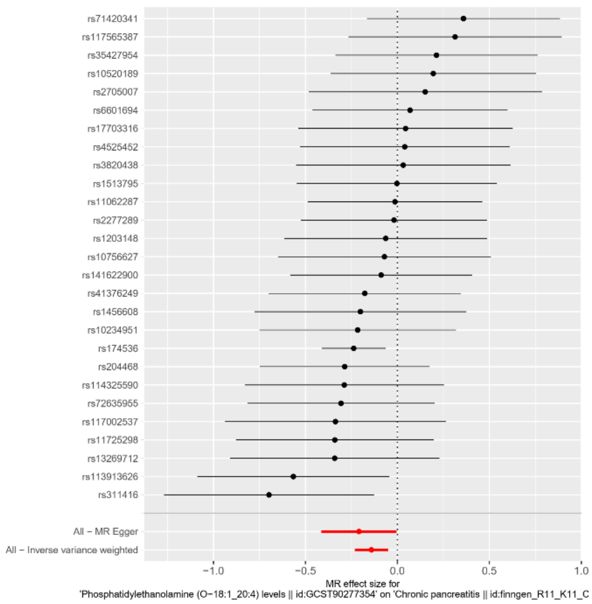

B

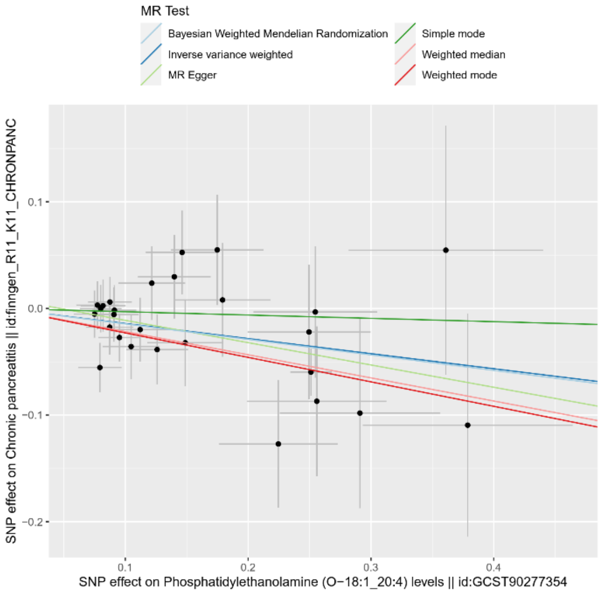

C

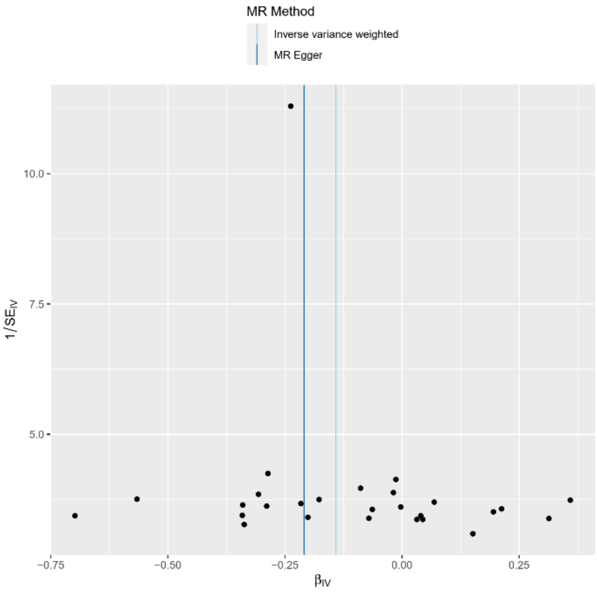

D

Figure S51 Leave-one-out analysis (A), MR effect size (B), scatter plot (C) and funnel plot (D) for Phosphatidylinositol (16:0_18:1) levels on chronic pancreatitis

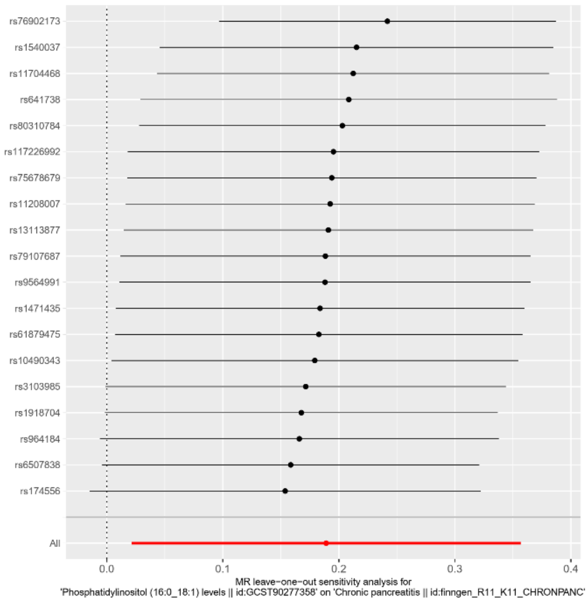

A

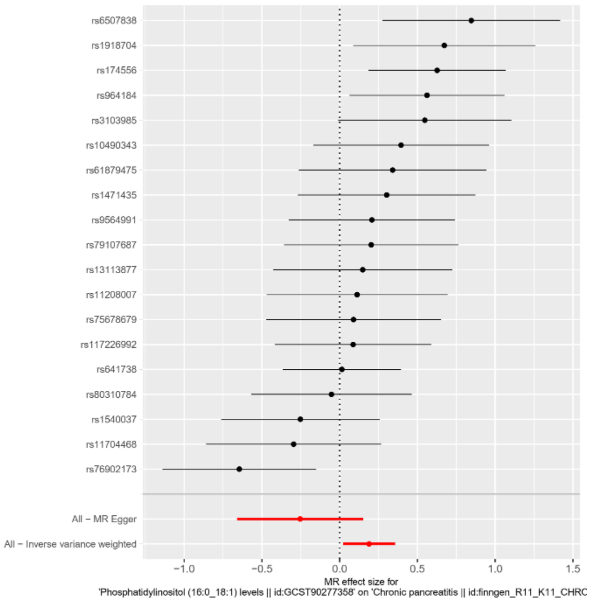

B

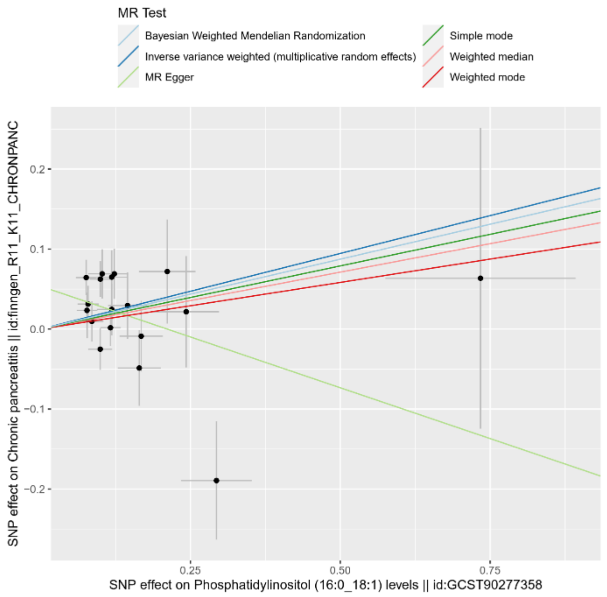

C

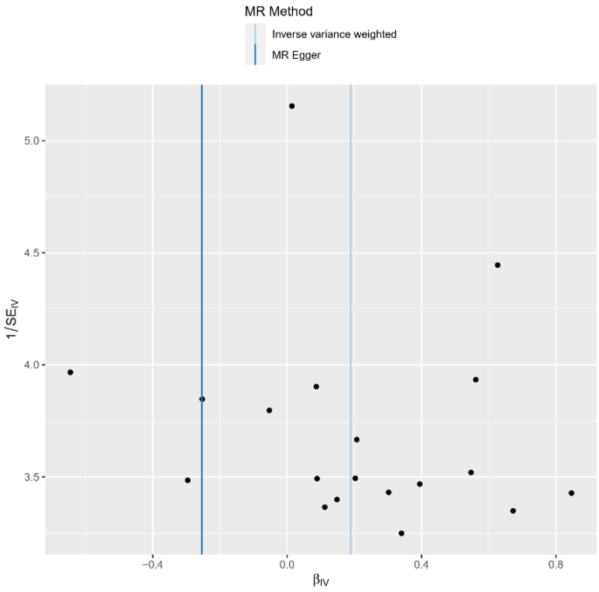

D

Figure S52 Leave-one-out analysis (A), MR effect size (B), scatter plot (C) and funnel plot (D) for Sphingomyelin (d34:2) levels on chronic pancreatitis

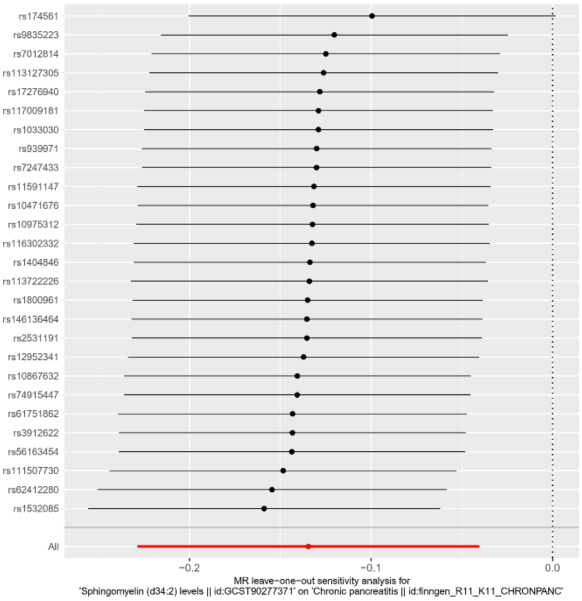

A

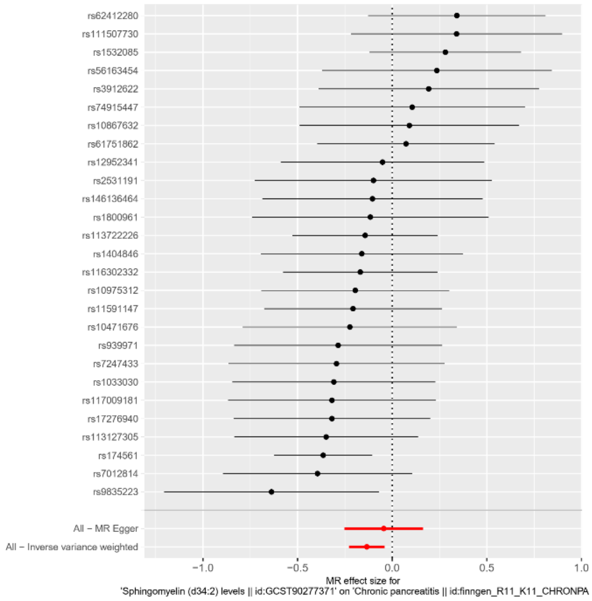

B

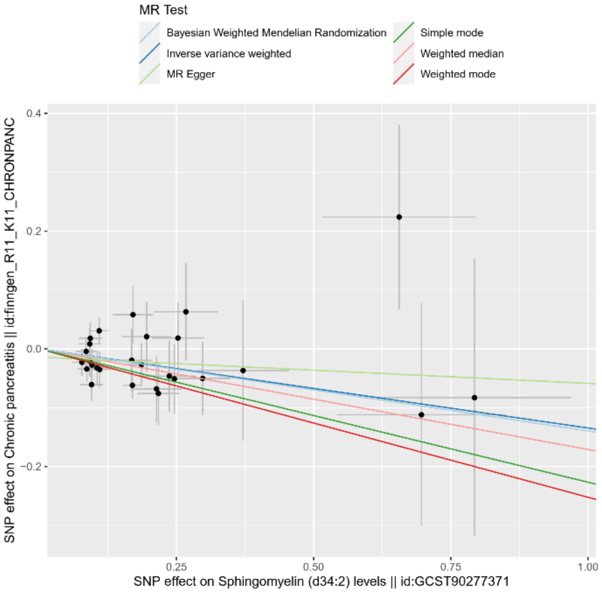

C

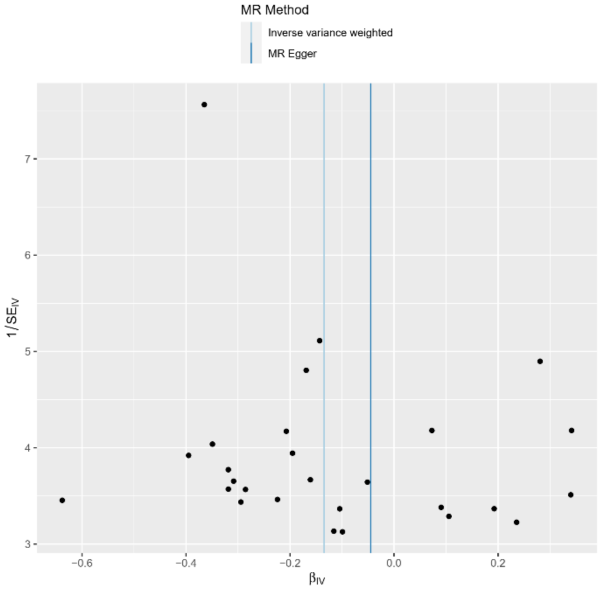

D

Figure S53 Leave-one-out analysis (A), MR effect size (B), scatter plot (C) and funnel plot (D) for Phosphatidylethanolamine (18:1_18:1) levels on chronic pancreatitis after eliminating outliers

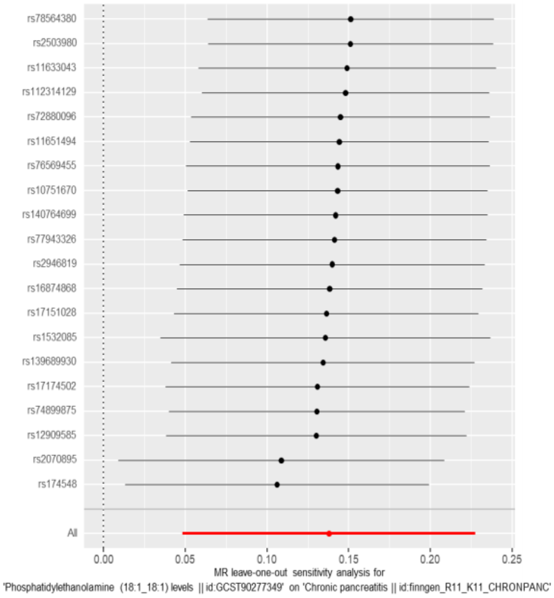

A

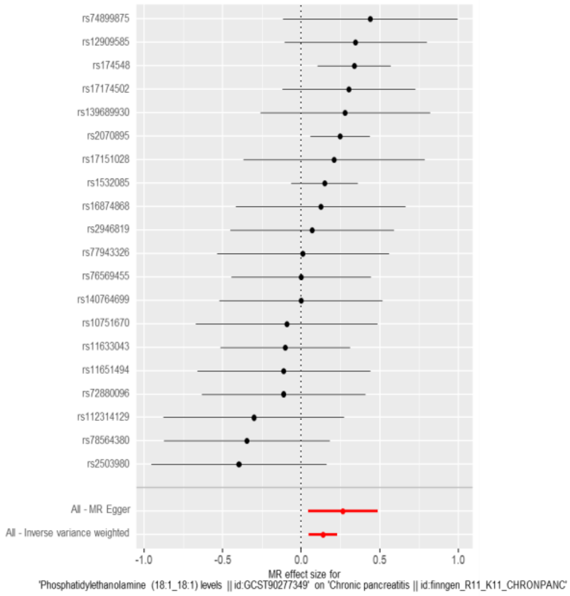

B

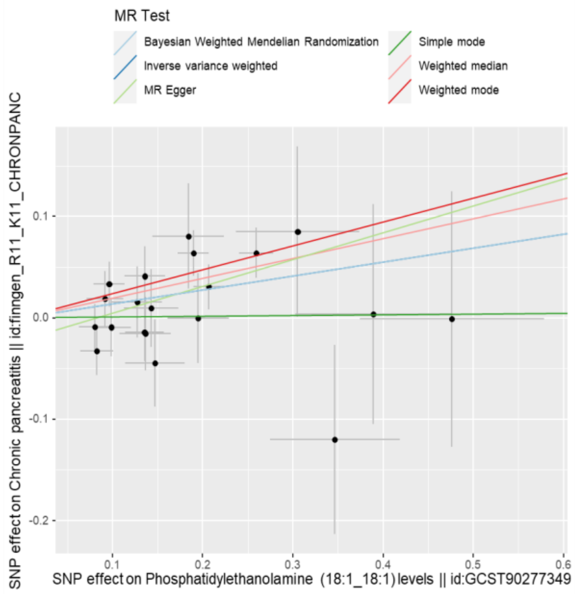

C

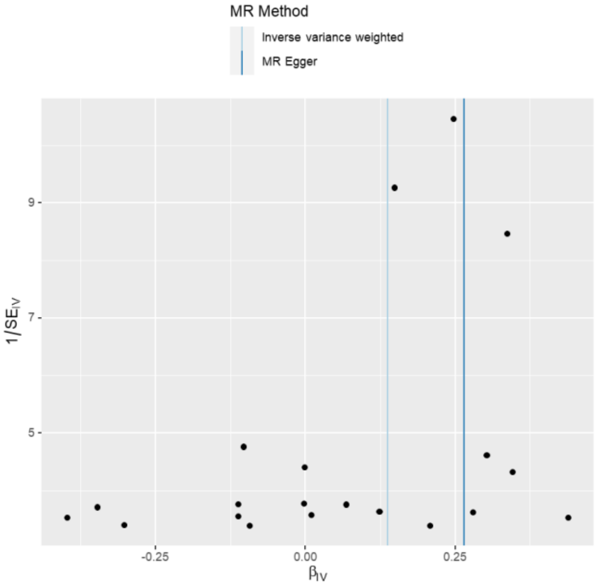

D

Figure S54 Leave-one-out analysis (A), MR effect size (B), scatter plot (C) and funnel plot (D) for Phosphatidylinositol (16:0_18:1) levels on chronic pancreatitis after eliminating outliers

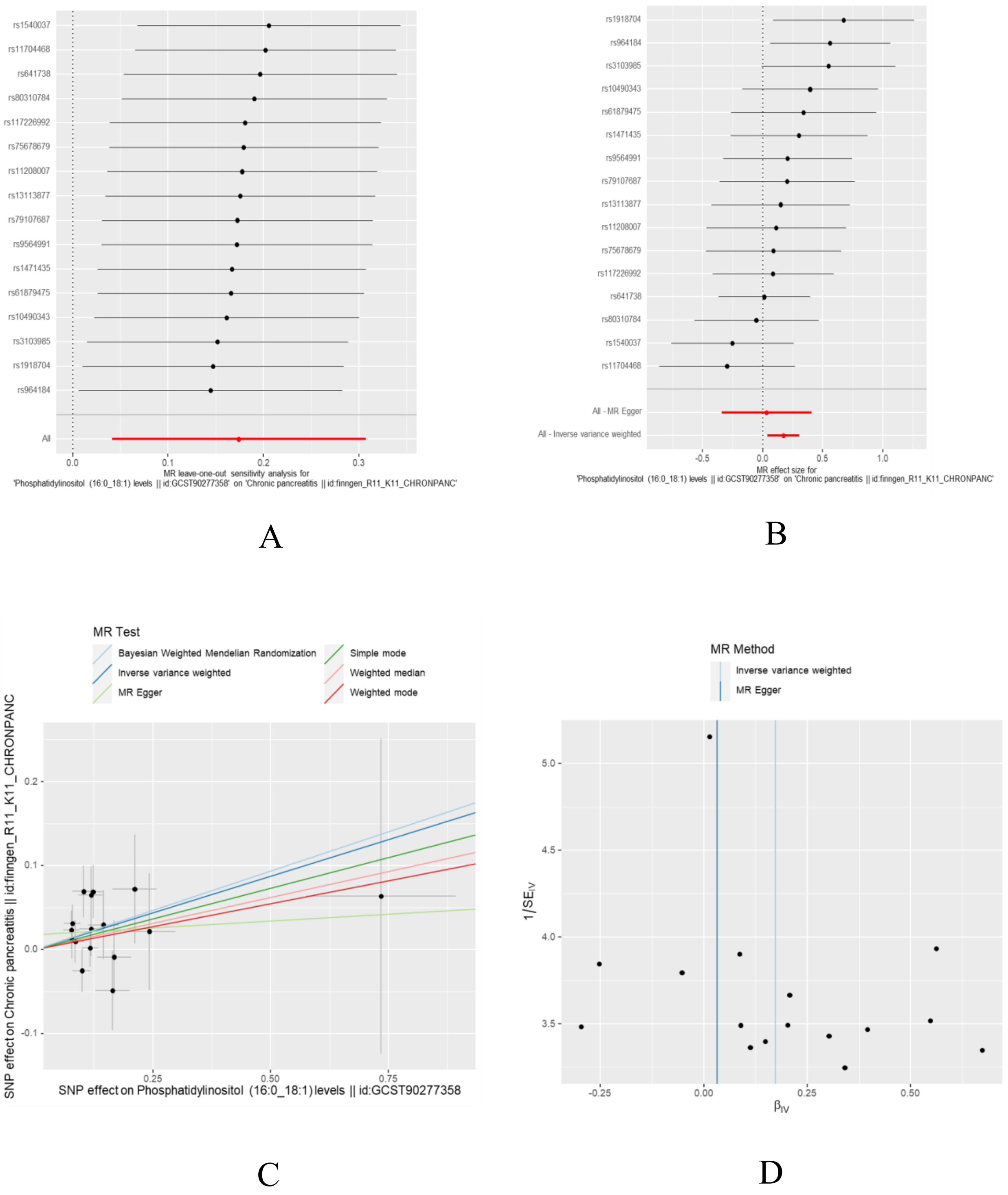

Supplement: Supplementary file 13 [file Image_2.pdf]
