# Supplementary material for: Genetically predicted causal link between the plasma lipidome and pancreatic diseases: a bidirectional Mendelian randomization study
Source: Front Nutr. 2025 Jan 15;11:1466509. doi: 10.3389/fnut.2024.1466509 (PMC11774697; doi:10.3389/fnut.2024.1466509)
Supplement: Supplementary file 14 [file Image_3.pdf]

Figure S55 Leave-one-out analysis (A), MR effect size (B), scatter plot (C) and funnel plot (D) for Phosphatidylethanolamine (18:1_0:0) levels on alcohol-induced acute pancreatitis

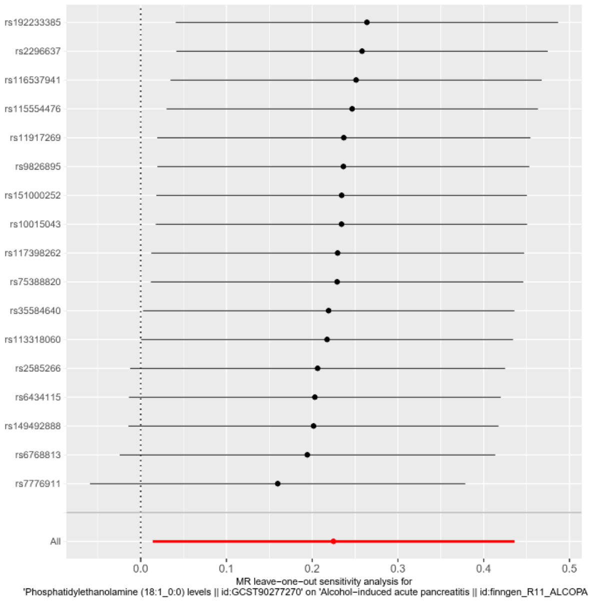

A

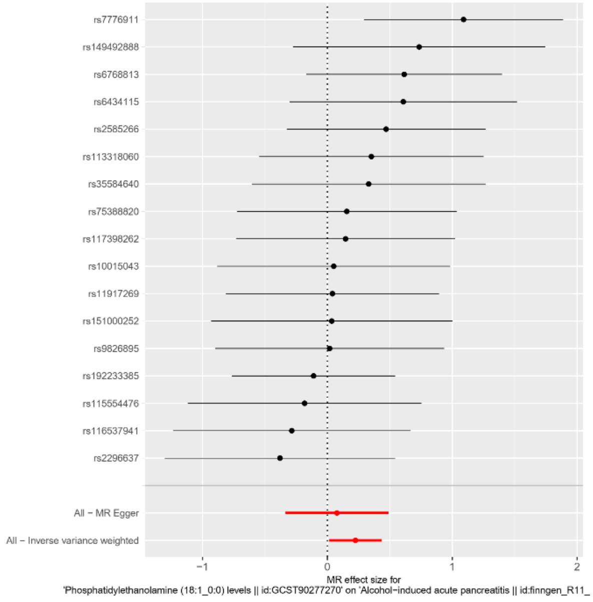

B

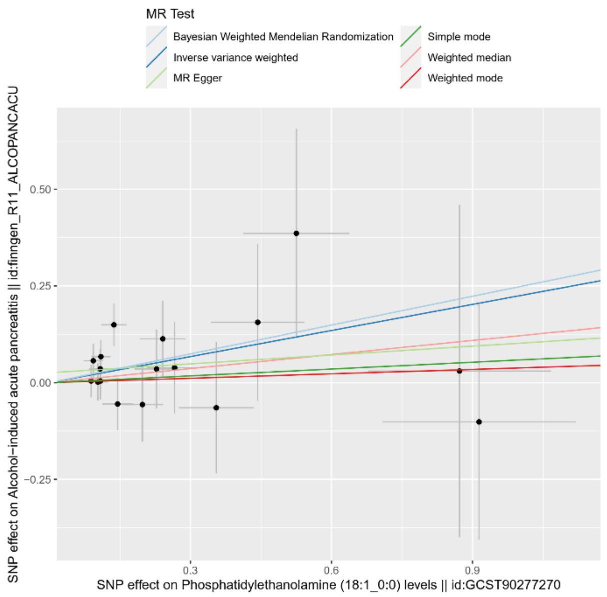

C

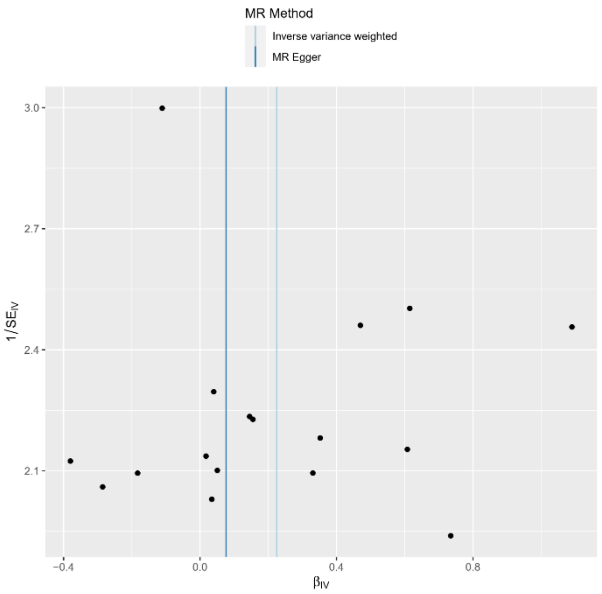

D

Figure S56 Leave-one-out analysis (A), MR effect size (B), scatter plot (C) and funnel plot (D) for Phosphatidylethanolamine (18:1_18:1) levels on alcohol-induced acute pancreatitis

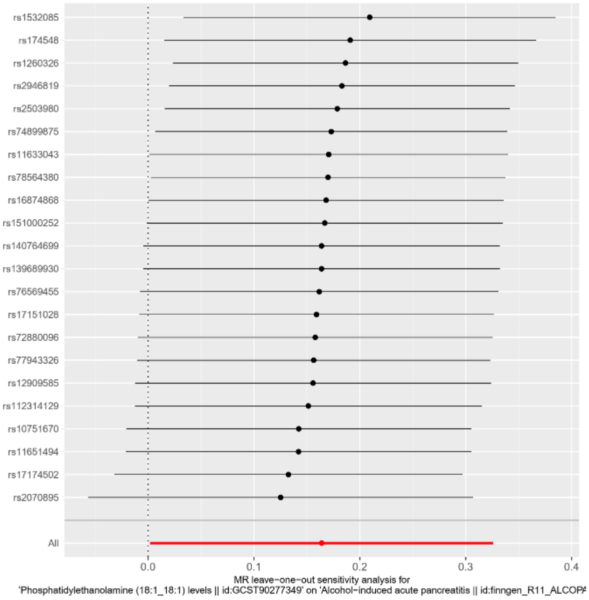

A

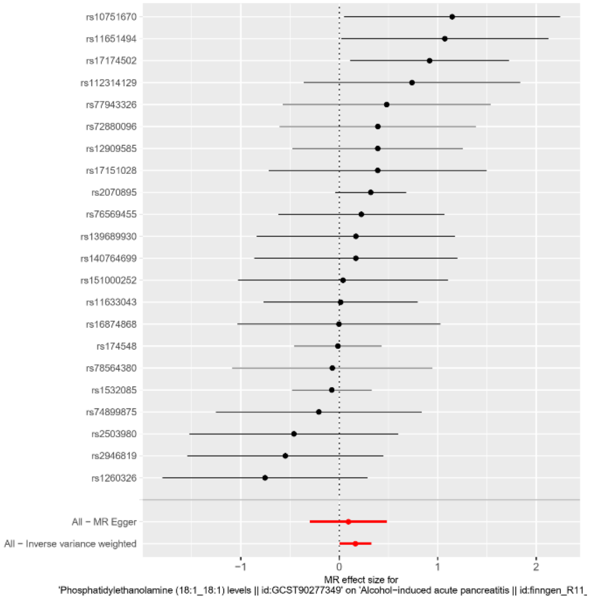

B

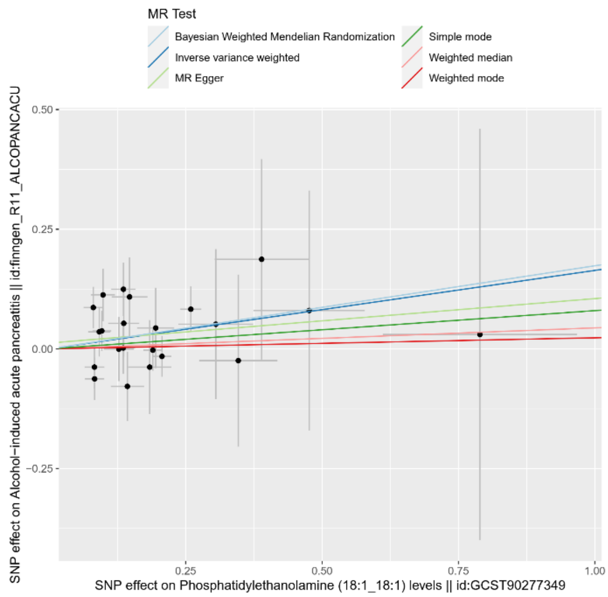

C

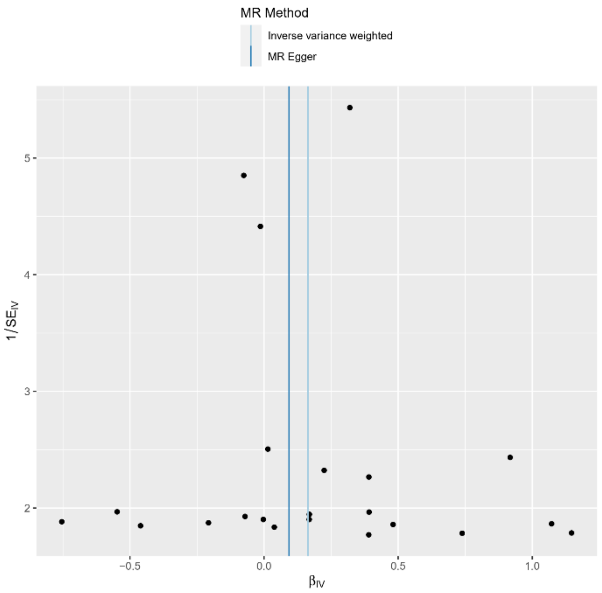

D
