# Supplementary material for: Genetically predicted causal link between the plasma lipidome and pancreatic diseases: a bidirectional Mendelian randomization study
Source: Front Nutr. 2025 Jan 15;11:1466509. doi: 10.3389/fnut.2024.1466509 (PMC11774697; doi:10.3389/fnut.2024.1466509)
Supplement: Supplementary file 15 [file Image_4.pdf]

Figure S57 Leave-one-out analysis (A), MR effect size (B), scatter plot (C) and funnel plot (D) for Sterol ester (27:1/16:0) levels on alcohol-induced chronic pancreatitis

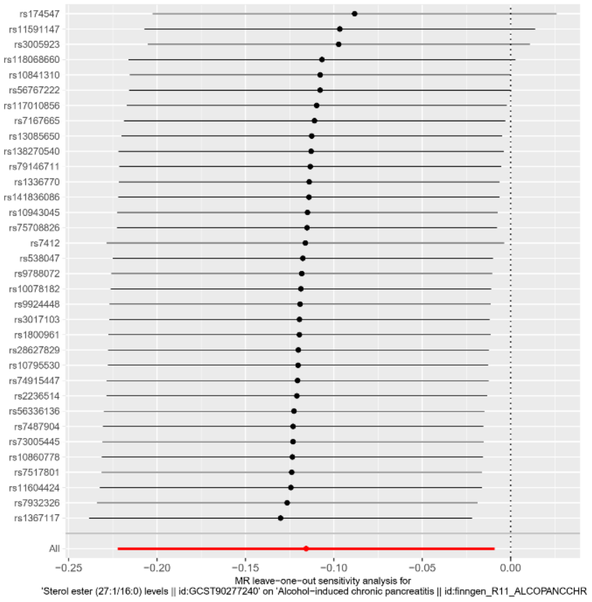


A

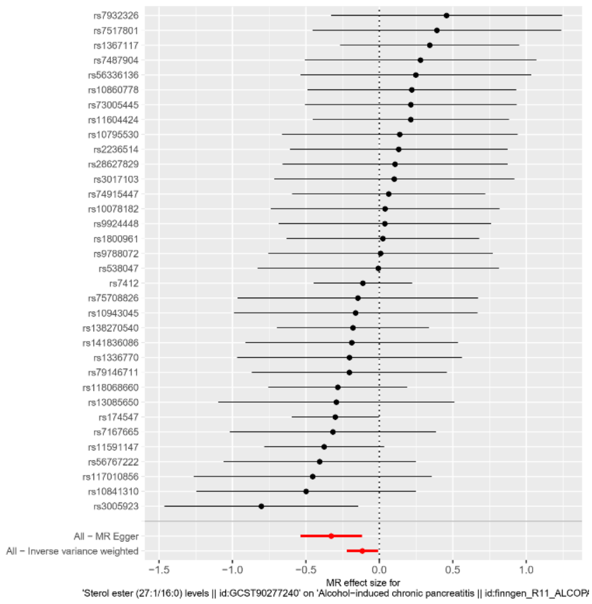


B

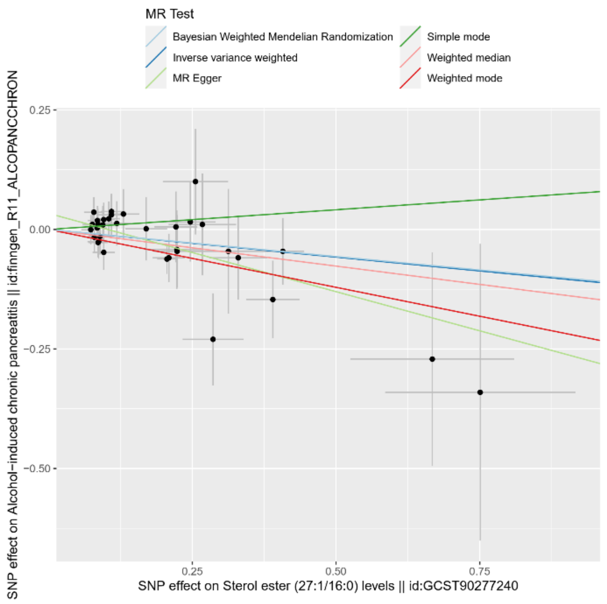


C

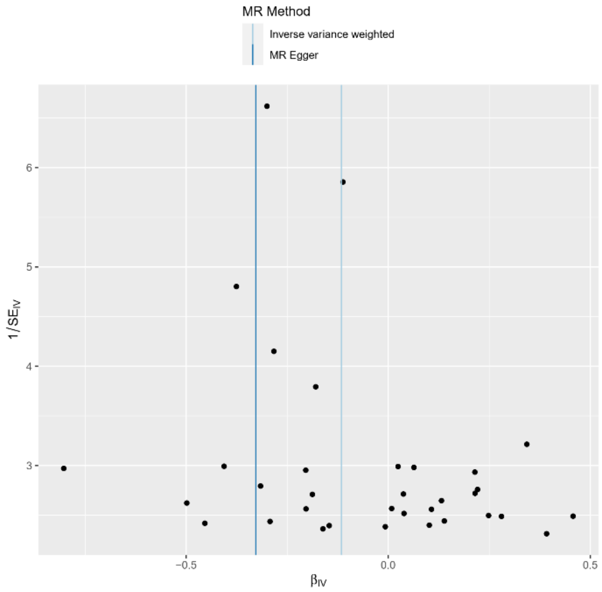


D

Figure S58 Leave-one-out analysis (A), MR effect size (B), scatter plot (C) and funnel plot (D) for Sterol ester (27:1/20:4) levels on alcohol-induced chronic pancreatitis

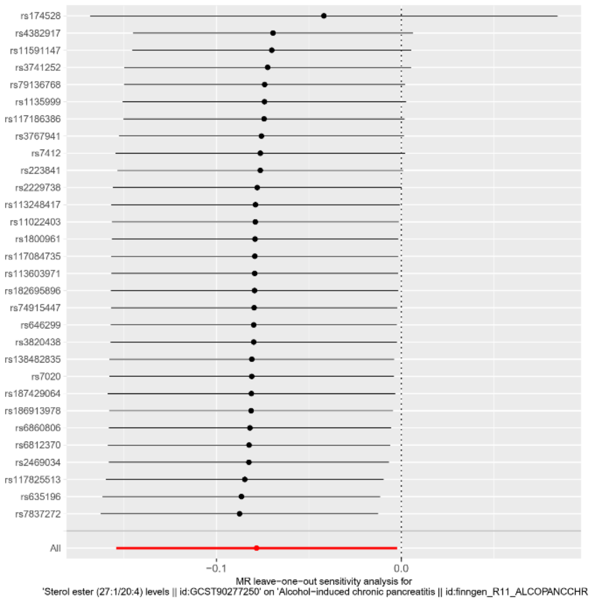


A

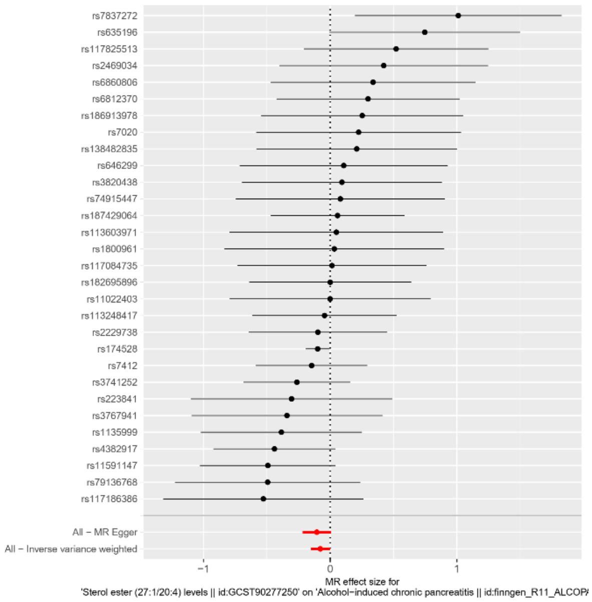


B

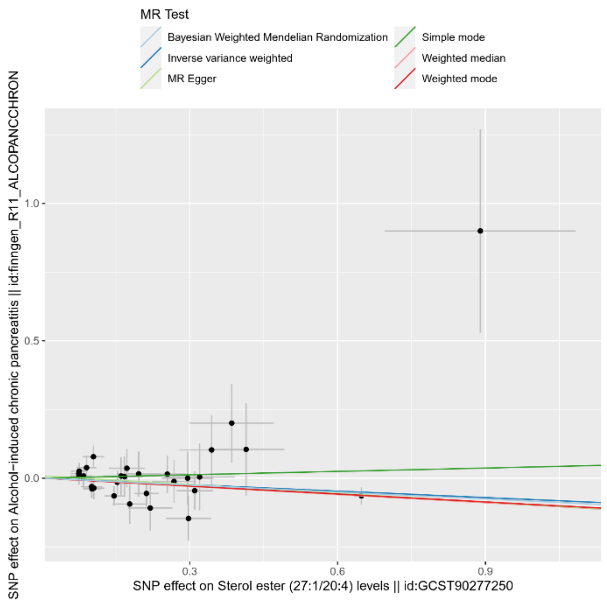


C

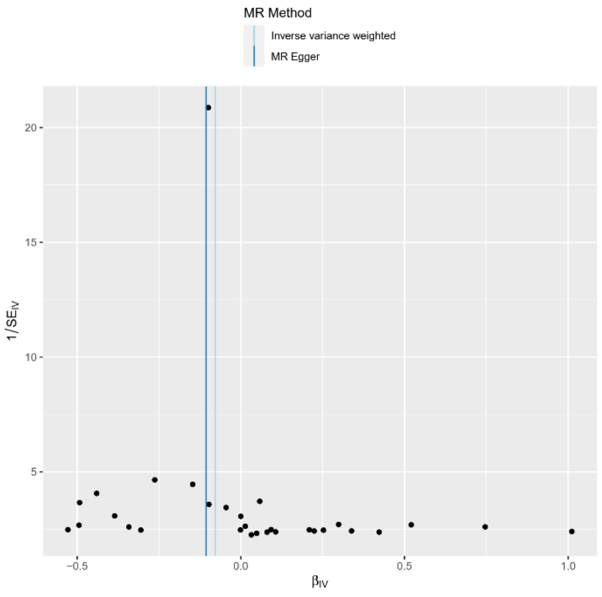


D

Figure S59 Leave-one-out analysis (A), MR effect size (B), scatter plot (C) and funnel plot (D) for Sterol ester (27:1/22:6) levels on alcohol-induced chronic pancreatitis

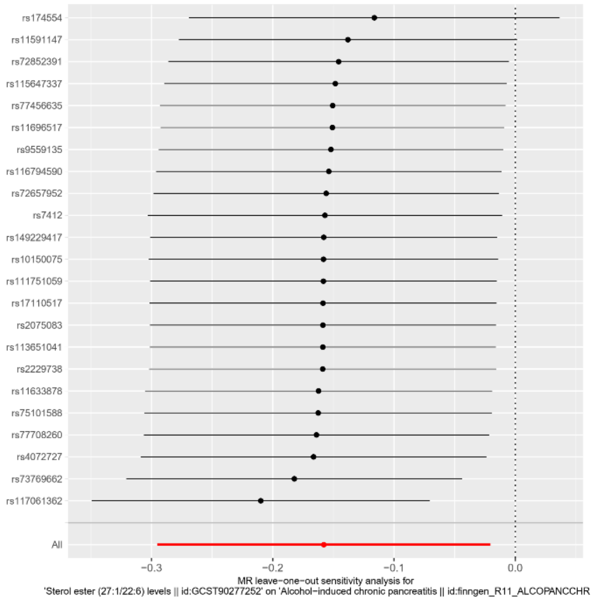


A

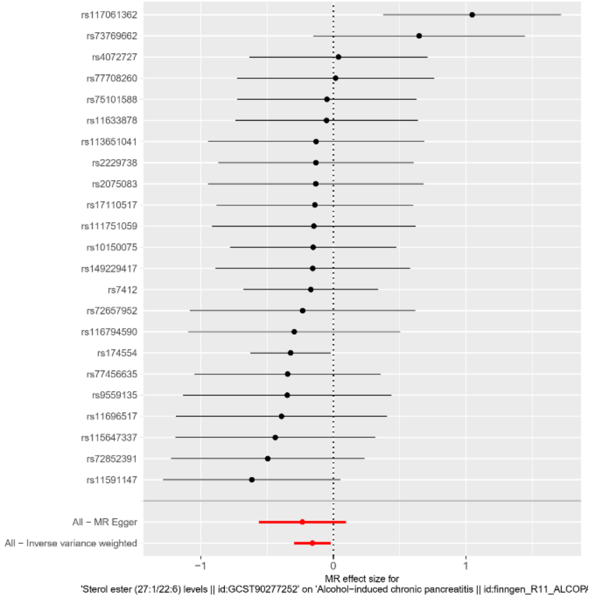


B

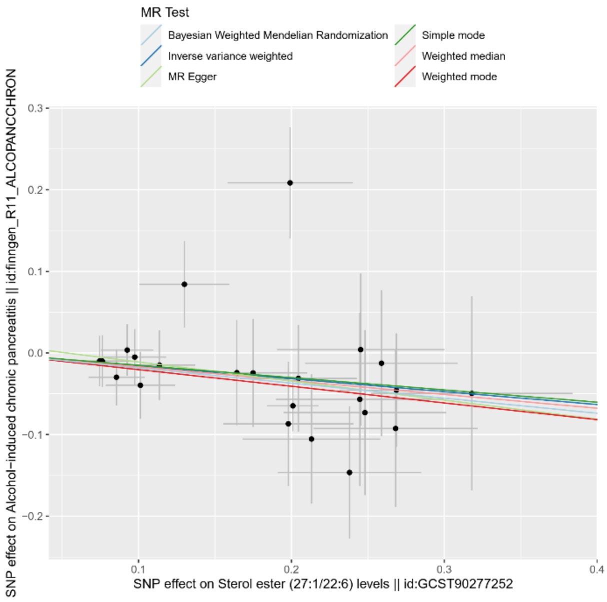


C

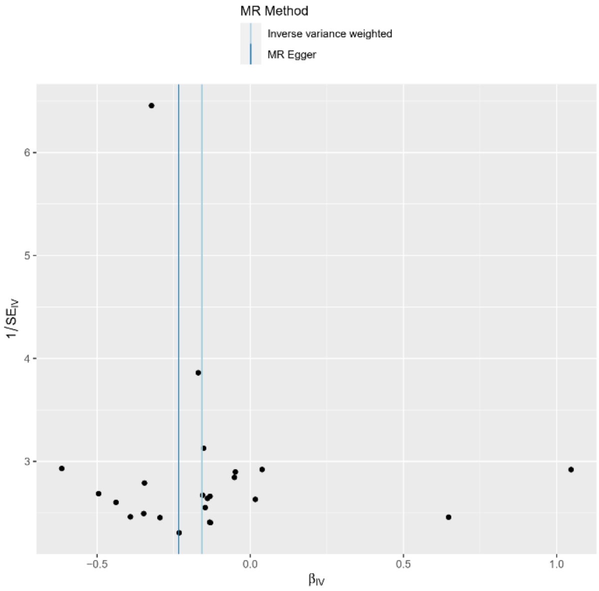


D

Figure S60 Leave-one-out analysis (A), MR effect size (B), scatter plot (C) and funnel plot (D) for Phosphatidylcholine (20:4_0:0) levels on alcohol-induced chronic pancreatitis

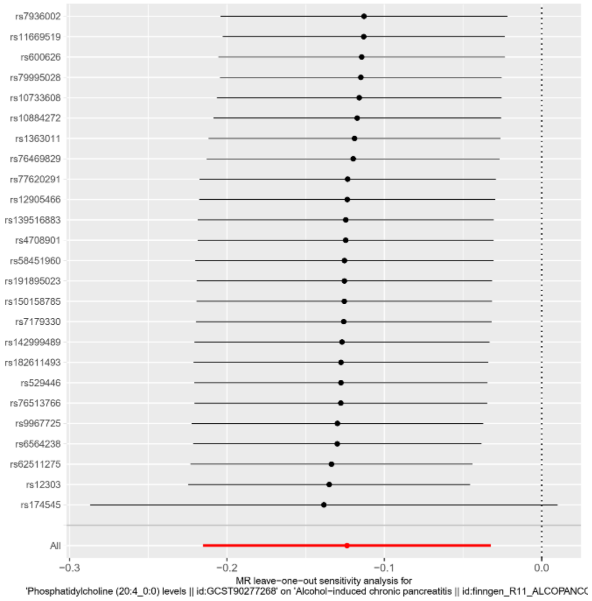


A

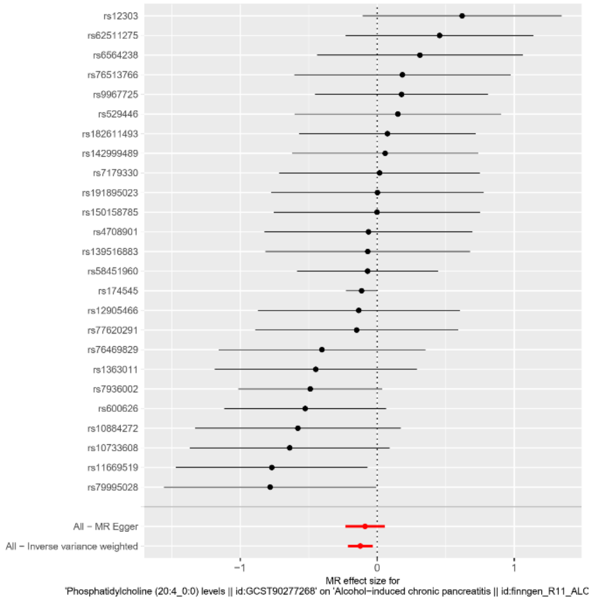


B

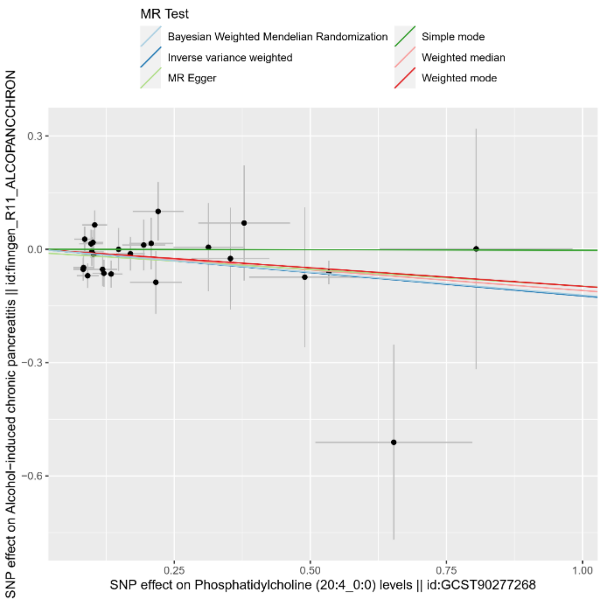


C

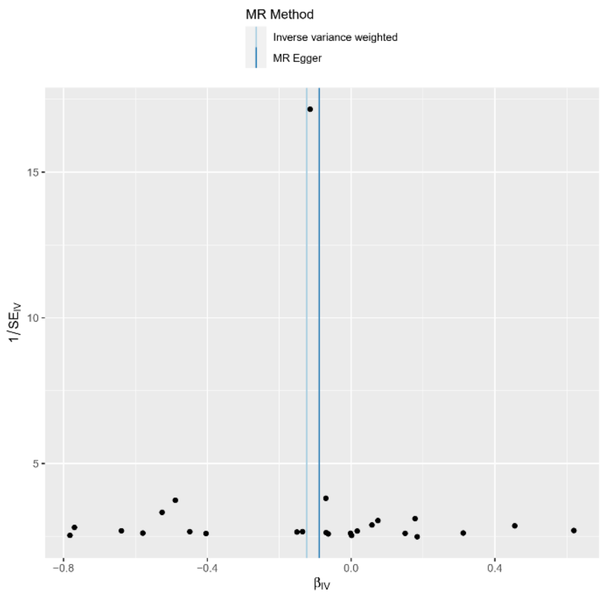


D

Figure S61 Leave-one-out analysis (A), MR effect size (B), scatter plot (C) and funnel plot (D) for Phosphatidylethanolamine (18:2_0:0) levels on alcohol-induced chronic pancreatitis

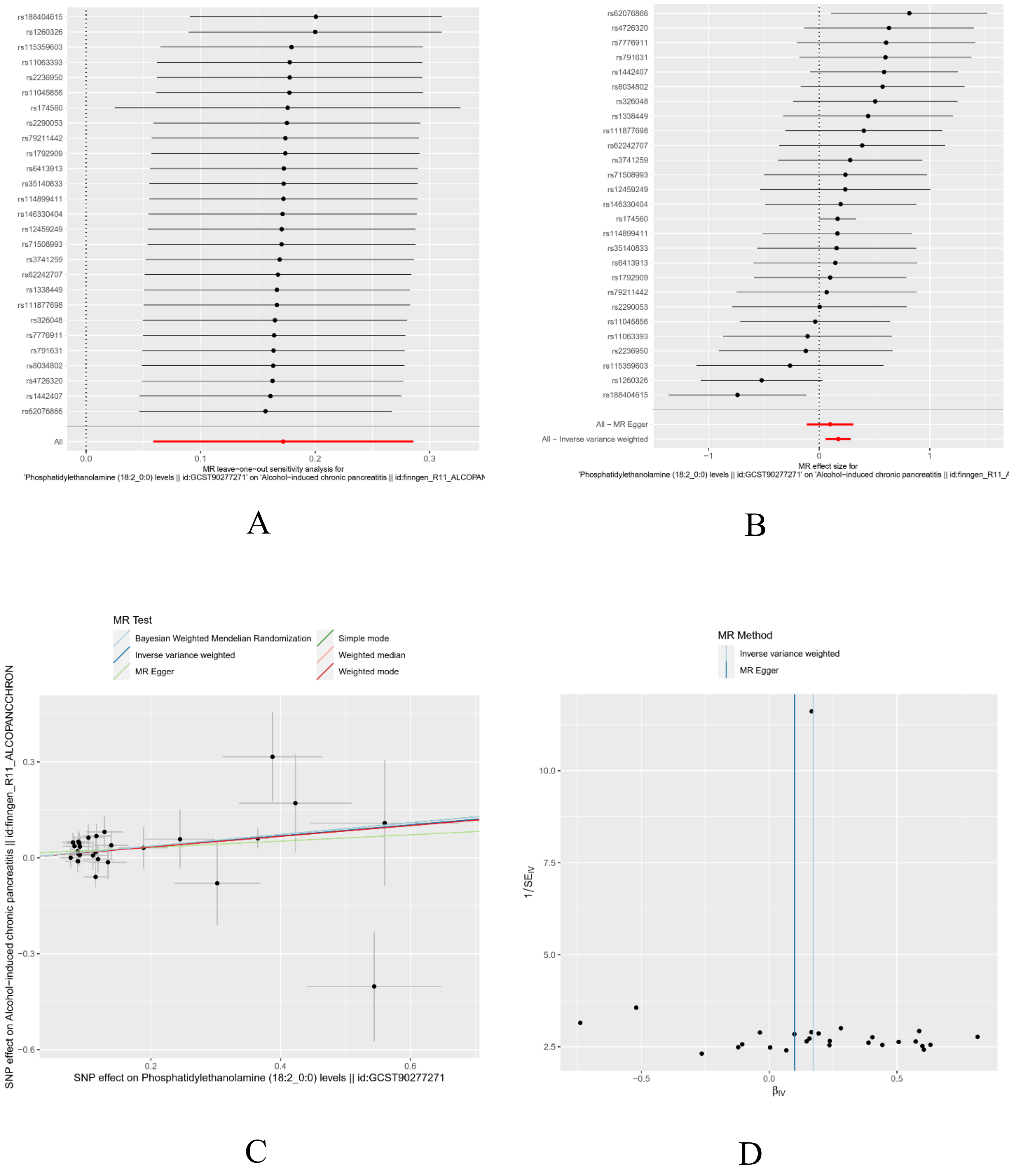

Figure S62 Leave-one-out analysis (A), MR effect size (B), scatter plot (C) and funnel plot (D) for Phosphatidylcholine (16:0_18:0) on alcohol-induced chronic pancreatitis

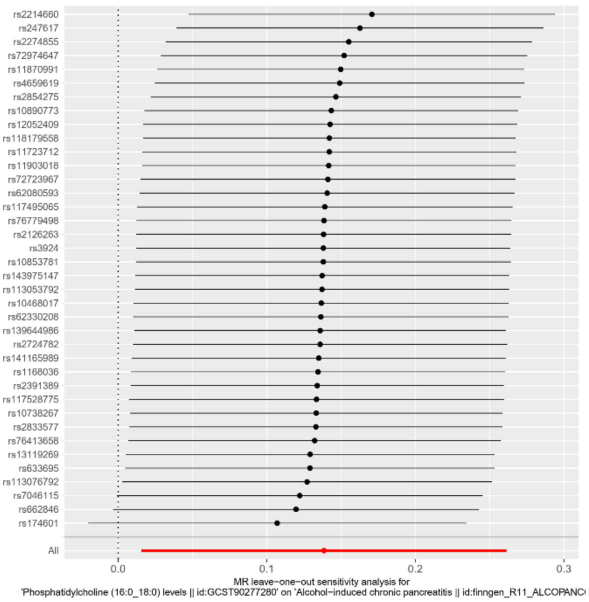


A

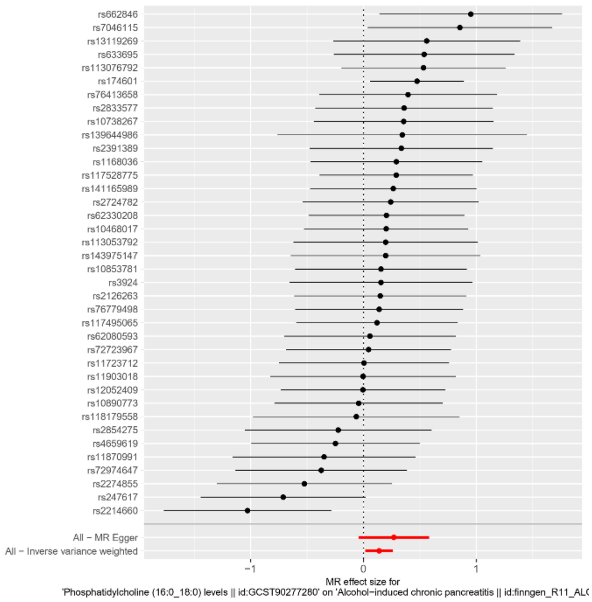


B

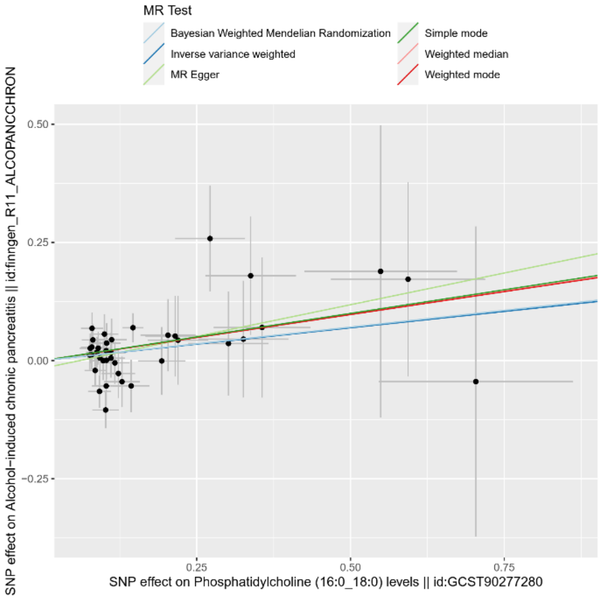


C

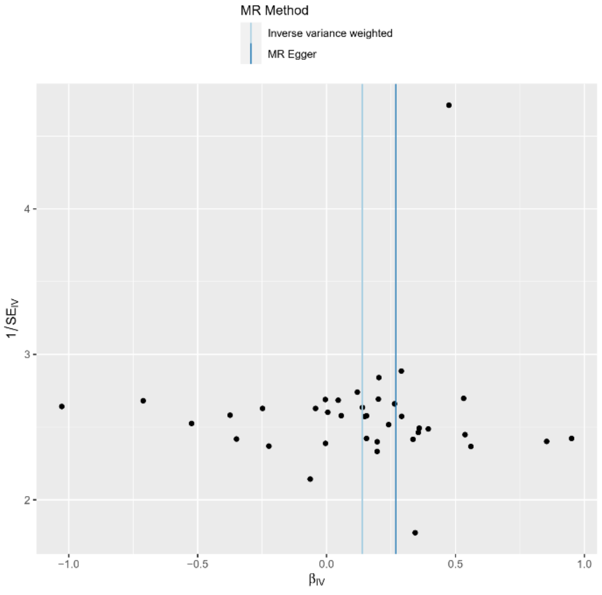


D

Figure S63 Leave-one-out analysis (A), MR effect size (B), scatter plot (C) and funnel plot (D) for Phosphatidylcholine (16:0_20:4) on alcohol-induced chronic pancreatitis

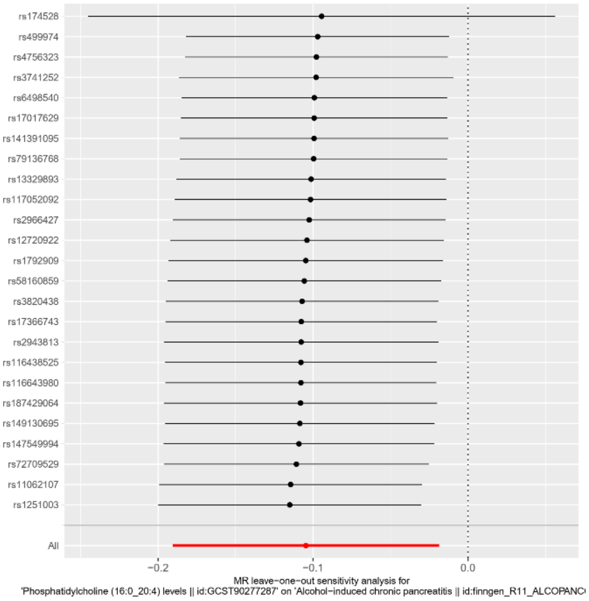


A

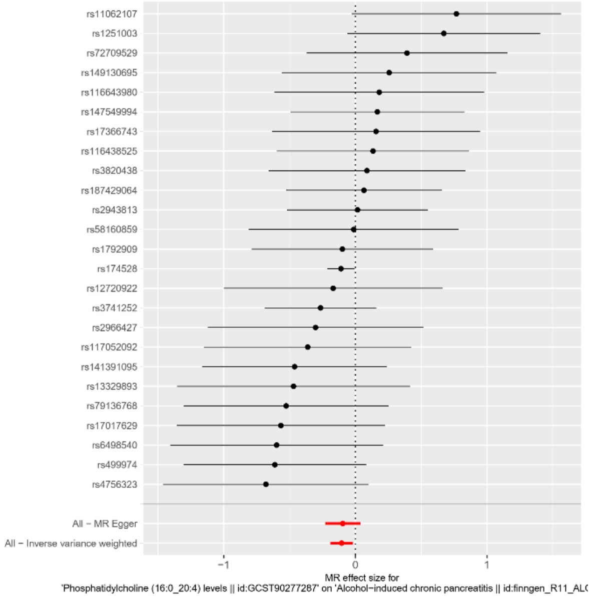


B

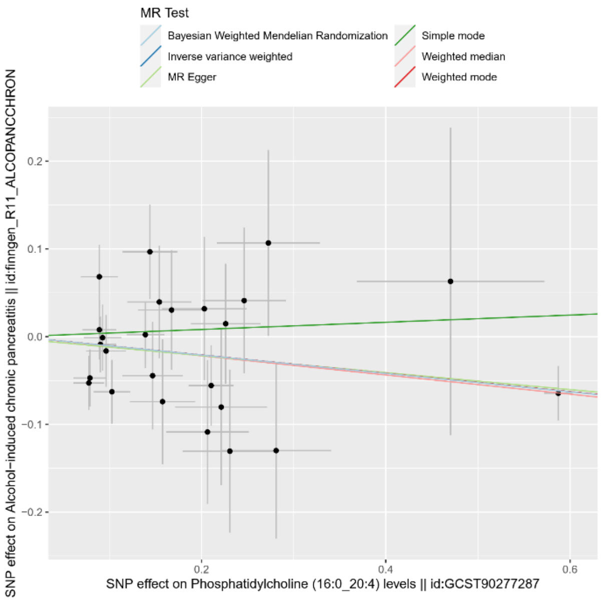


C

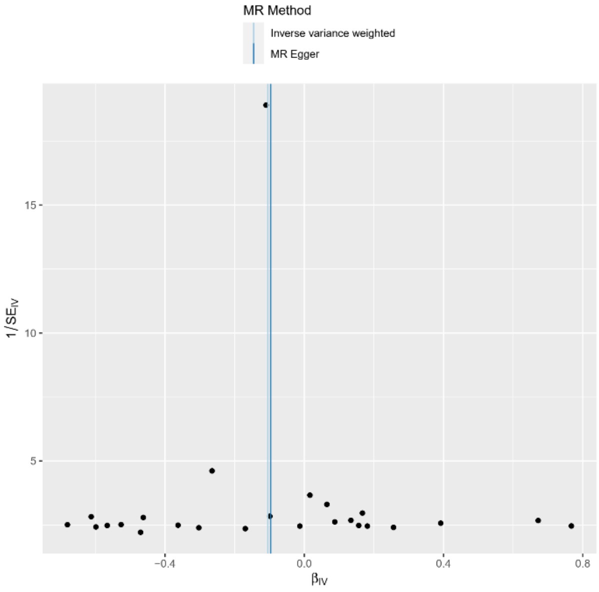


D

Figure S64 Leave-one-out analysis (A), MR effect size (B), scatter plot (C) and funnel plot (D) for Phosphatidylcholine (17:0_20:4) on alcohol-induced chronic pancreatitis

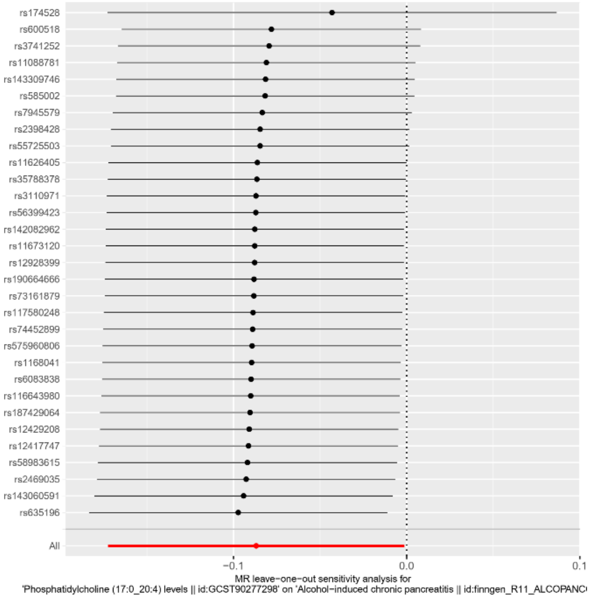


A

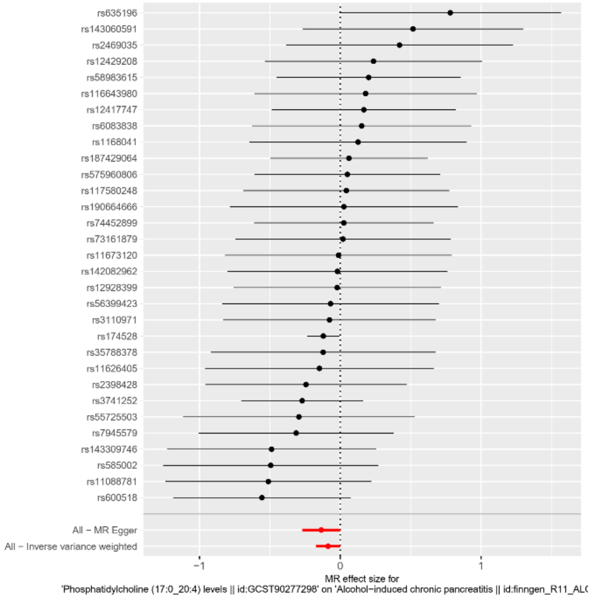


B

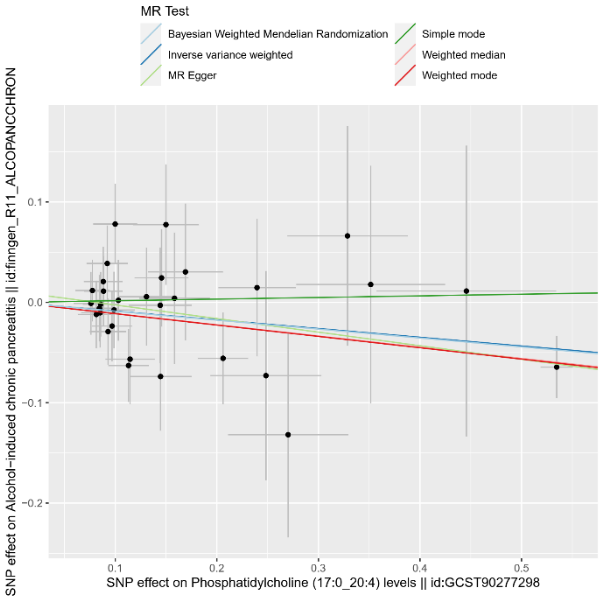


C

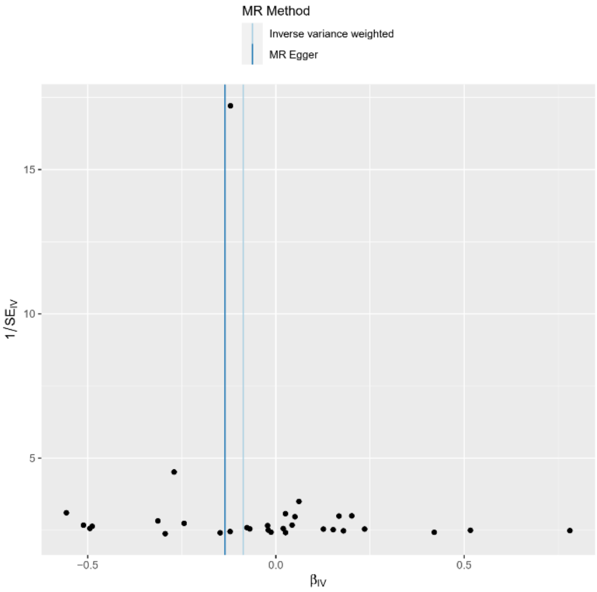


D

Figure S65 Leave-one-out analysis (A), MR effect size (B), scatter plot (C) and funnel plot (D) for Phosphatidylcholine (18:0_18:2) on alcohol-induced chronic pancreatitis

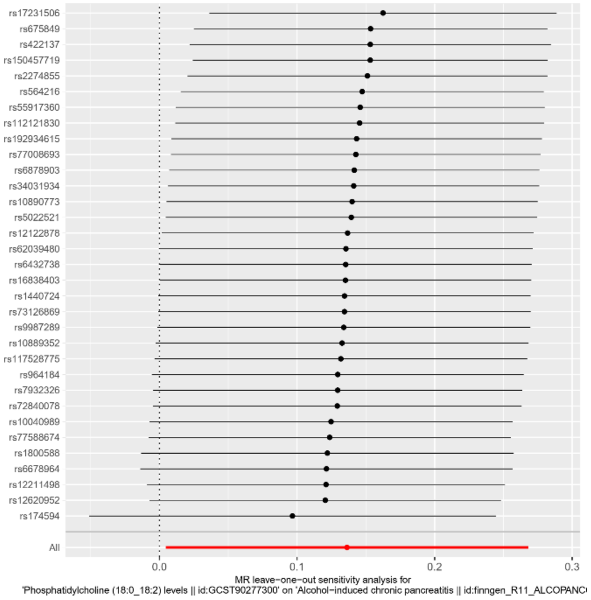


A

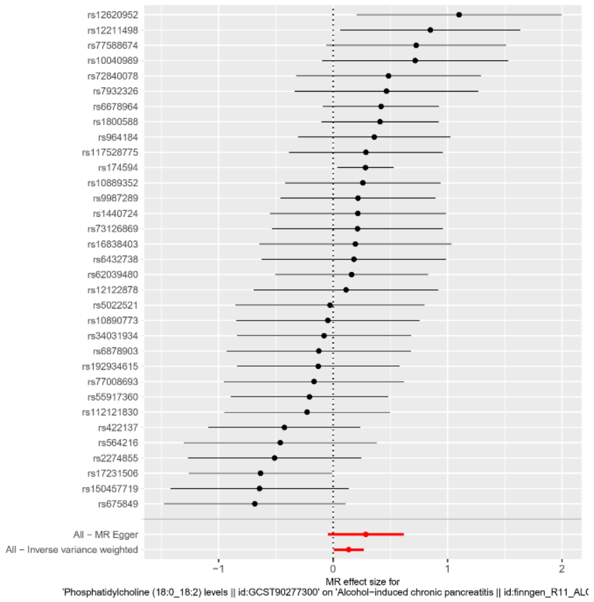


B

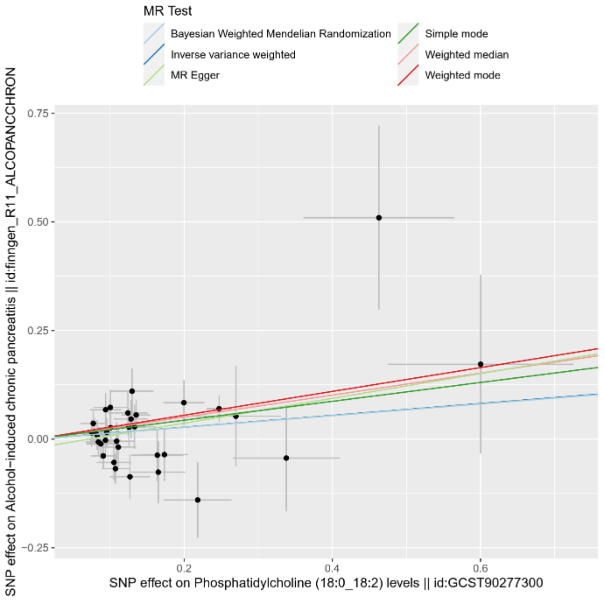


C

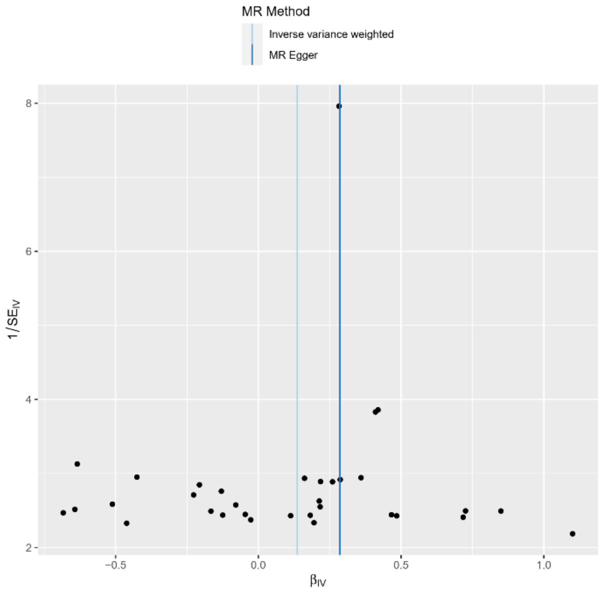


D

Figure S66 Leave-one-out analysis (A), MR effect size (B), scatter plot (C) and funnel plot (D) for Phosphatidylcholine (18:0_20:4) on alcohol-induced chronic pancreatitis

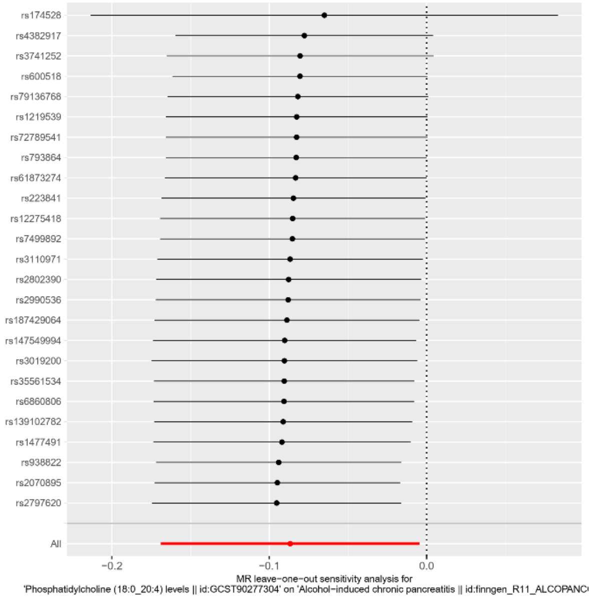


A

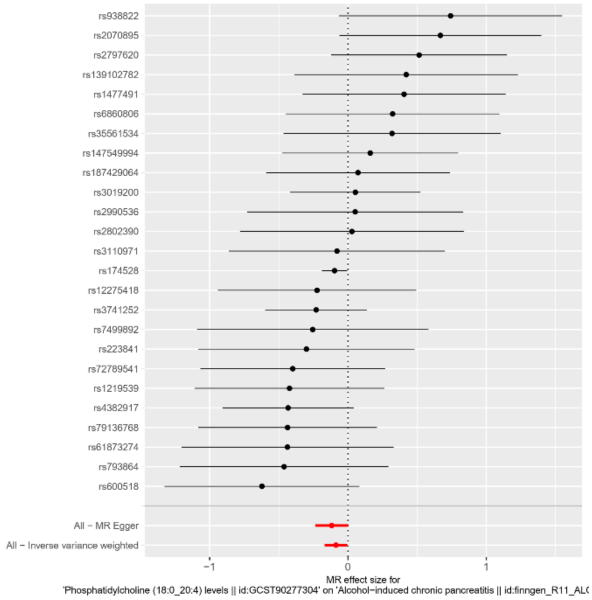


B

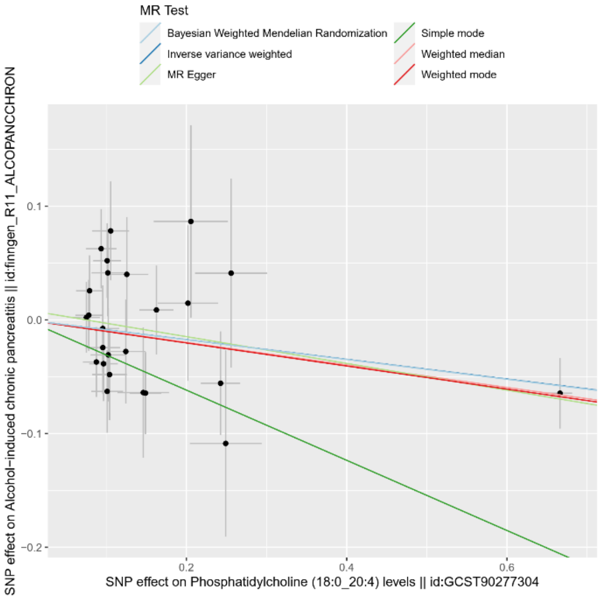


C

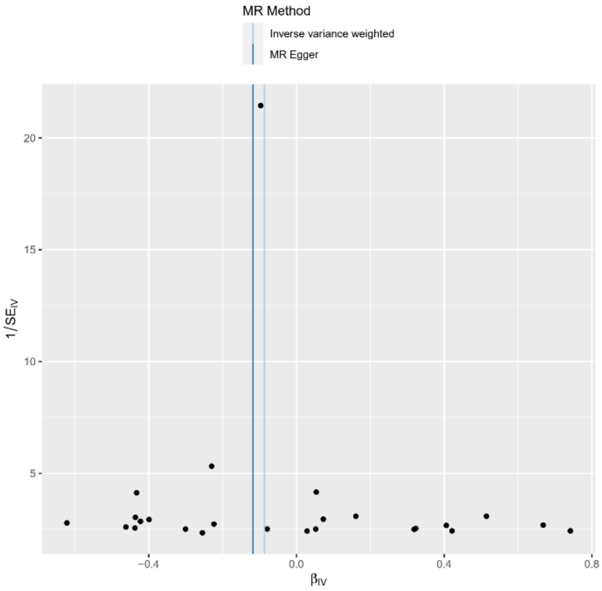


D

Figure S67 Leave-one-out analysis (A), MR effect size (B), scatter plot (C) and funnel plot (D) for Phosphatidylcholine (18:1_20:4) on alcohol-induced chronic pancreatitis

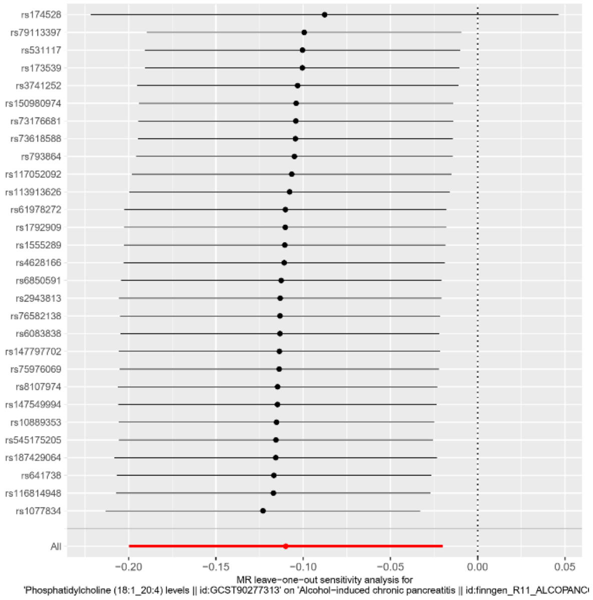


A

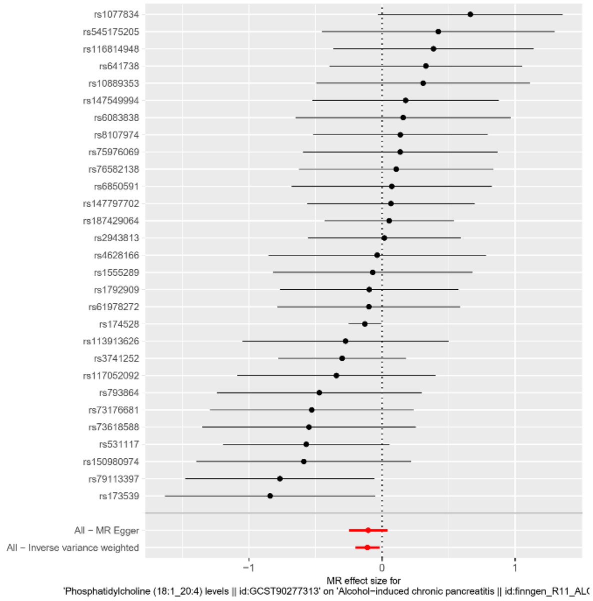


B

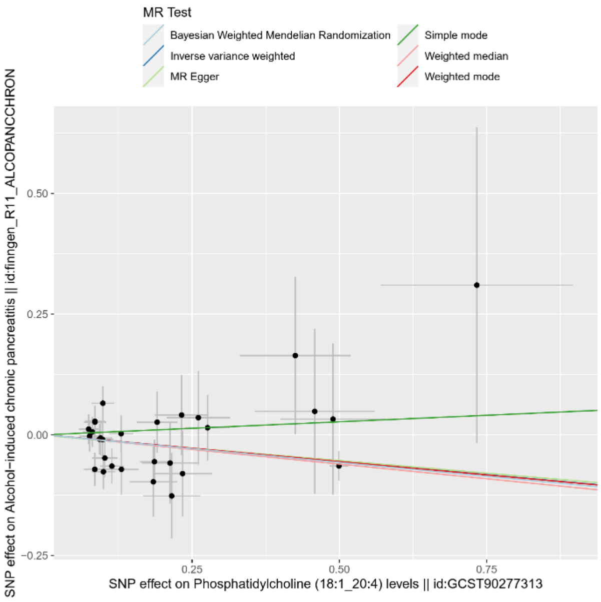


C

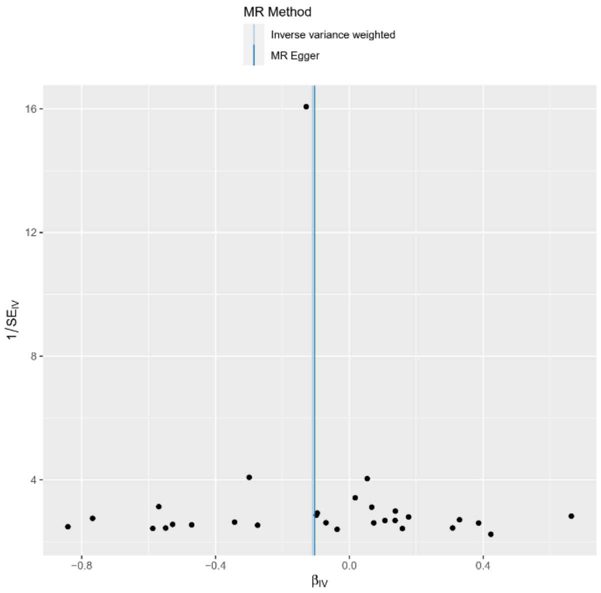


D

Figure S68 Leave-one-out analysis (A), MR effect size (B), scatter plot (C) and funnel plot (D) for Phosphatidylcholine (O-16:0_20:4) on alcohol-induced chronic pancreatitis

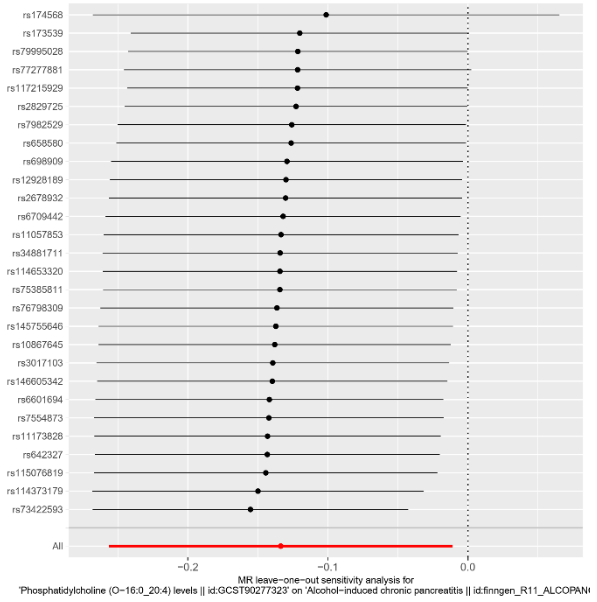


A

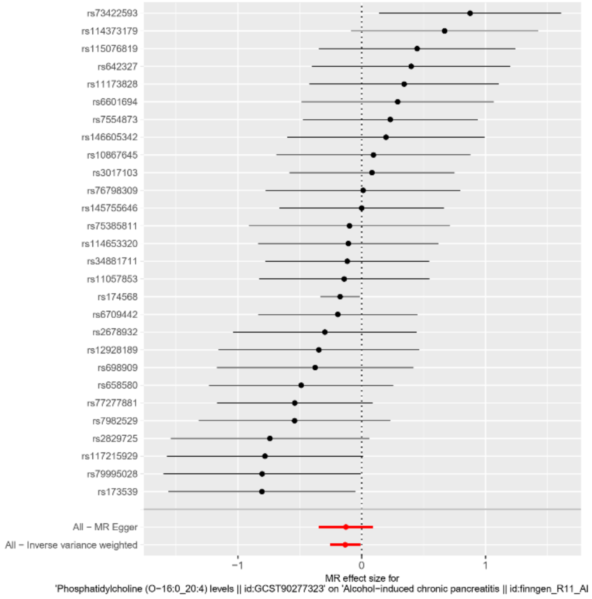


B

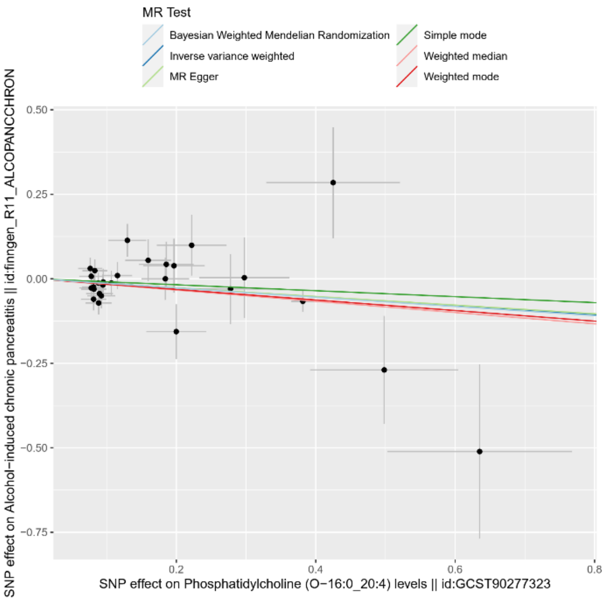


C

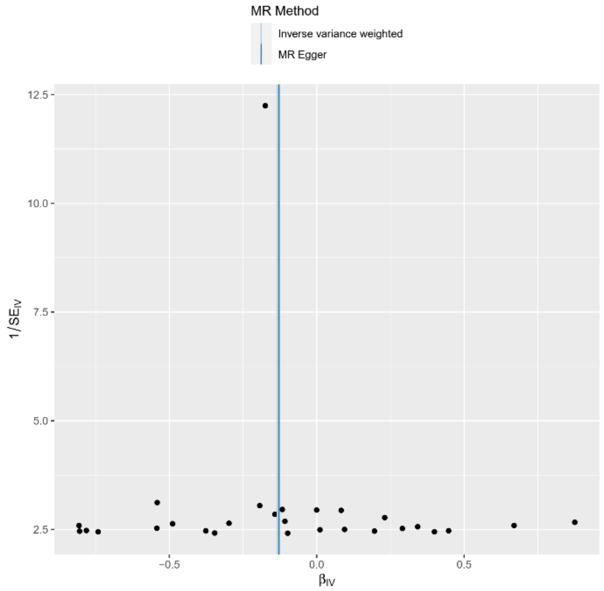


D

Figure S69 Leave-one-out analysis (A), MR effect size (B), scatter plot (C) and funnel plot (D) for Phosphatidylcholine (O-16:1_20:4) on alcohol-induced chronic pancreatitis

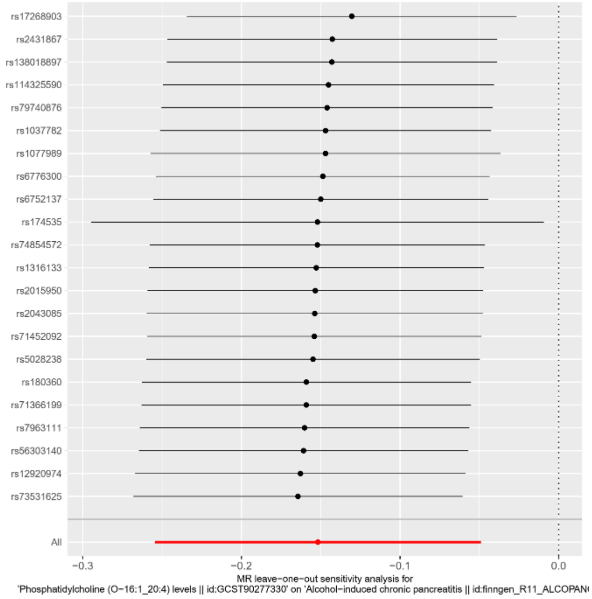


A

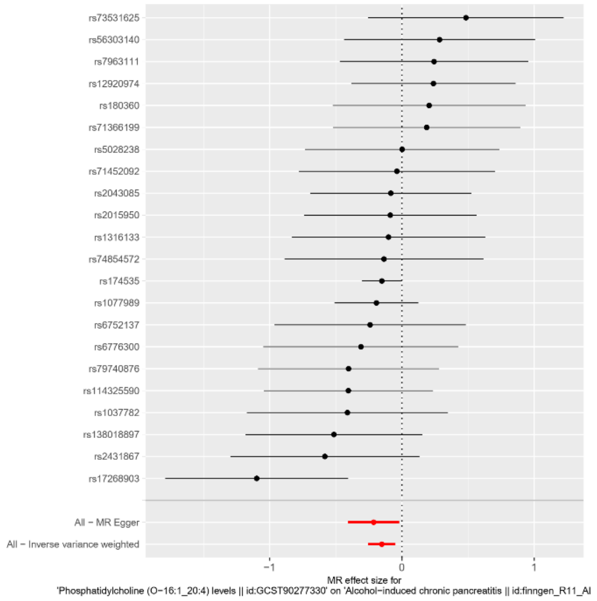


B

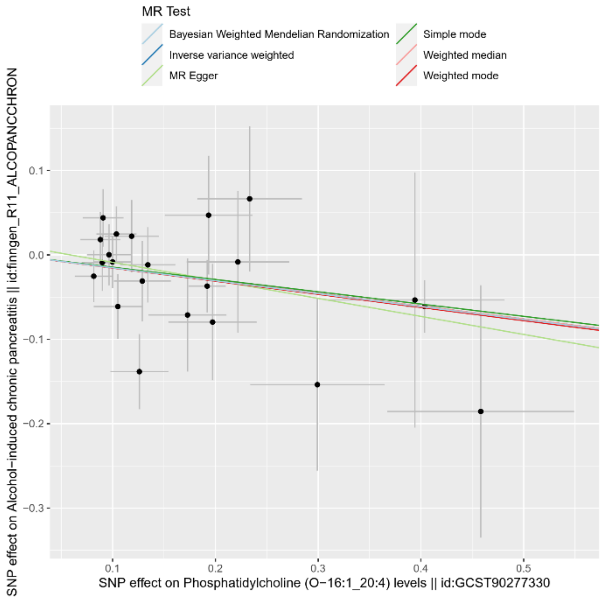


C

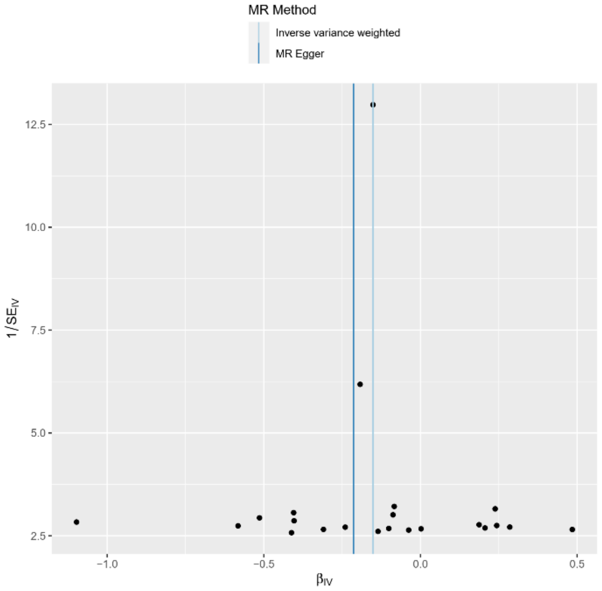


D

Figure S70 Leave-one-out analysis (A), MR effect size (B), scatter plot (C) and funnel plot (D) for Phosphatidylcholine (O-18:2_18:1) on alcohol-induced chronic pancreatitis

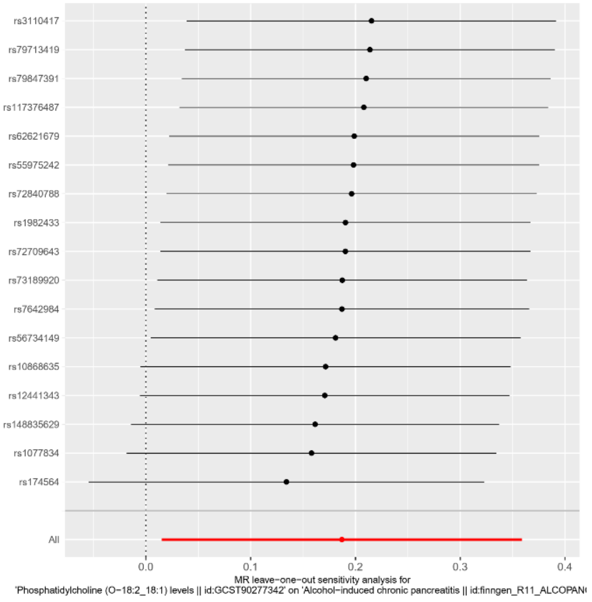


A

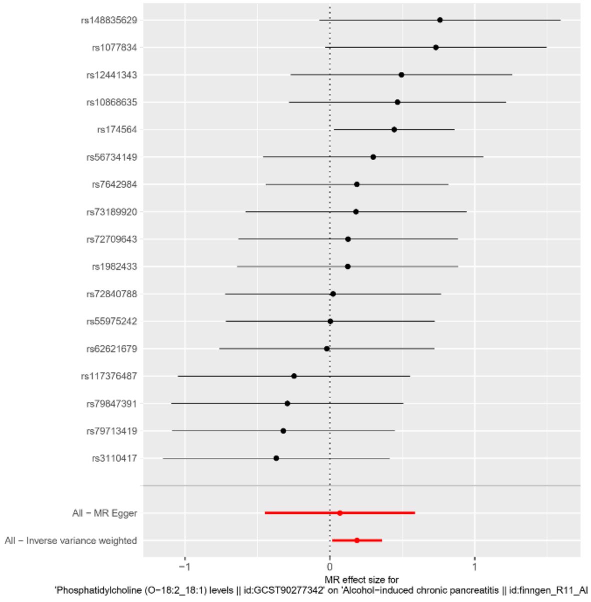


B

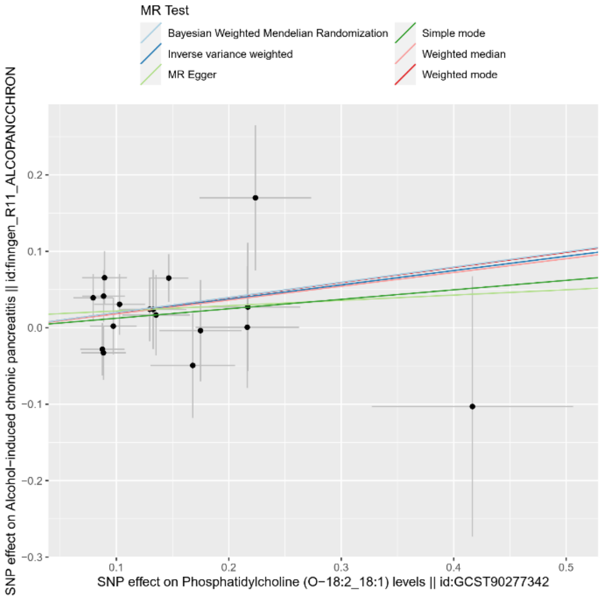


C

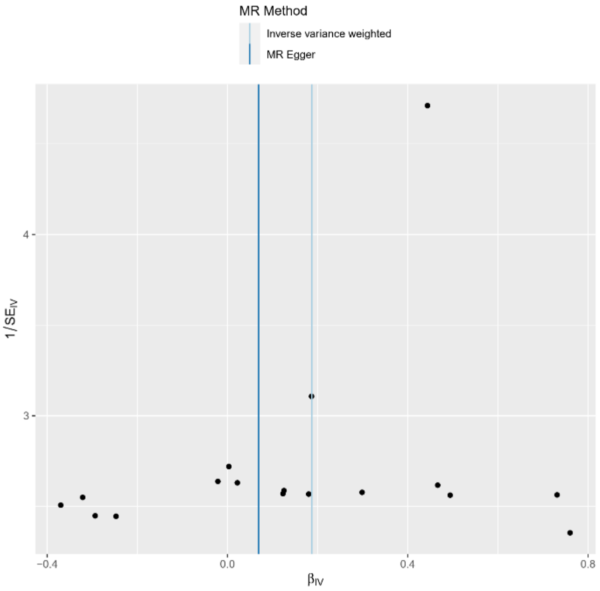


D

Figure S71 Leave-one-out analysis (A), MR effect size (B), scatter plot (C) and funnel plot (D) for Phosphatidylcholine (O-18:2_20:4) on alcohol-induced chronic pancreatitis

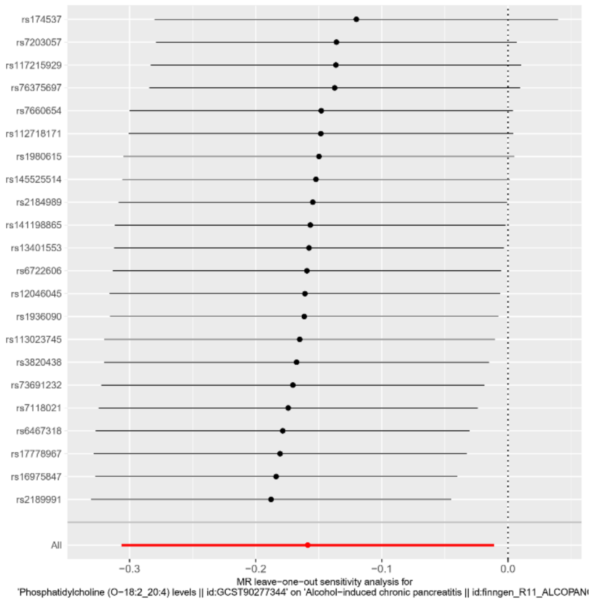


A

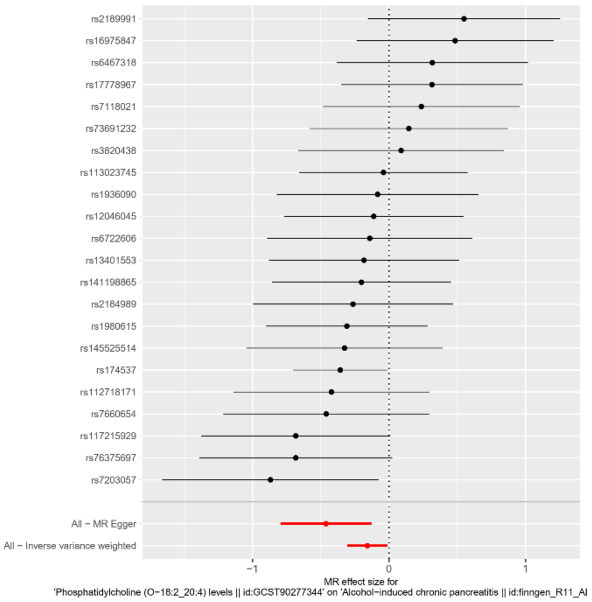


B

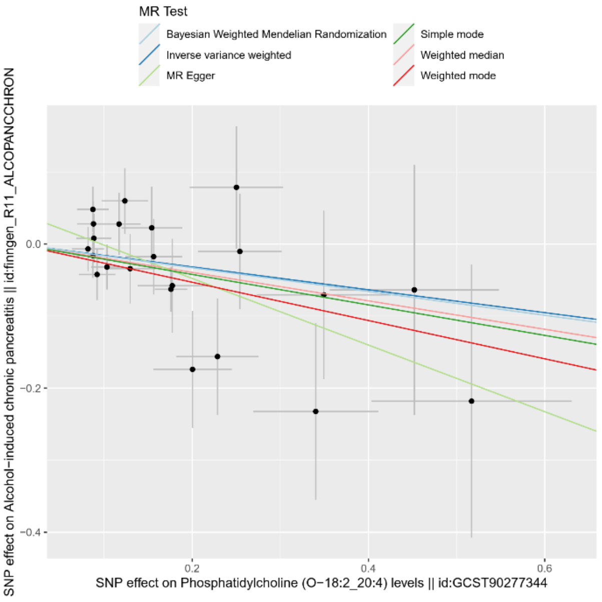


C

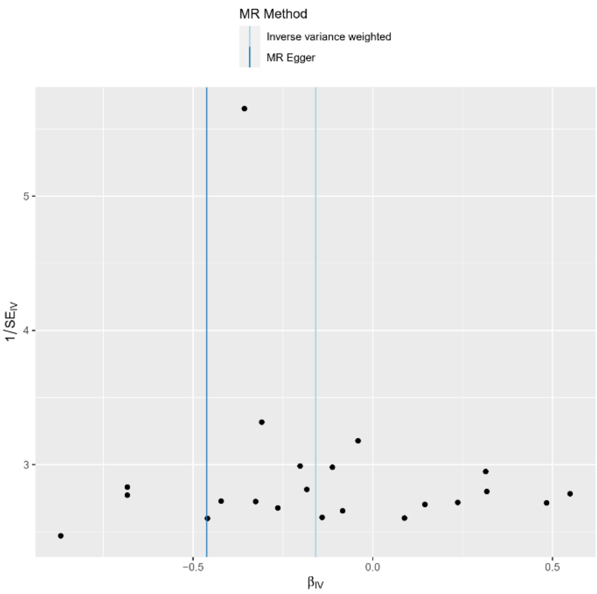


D

Figure S72 Leave-one-out analysis (A), MR effect size (B), scatter plot (C) and funnel plot (D) for Phosphatidylethanolamine (O-16:1_18:2) on alcohol-induced chronic pancreatitis

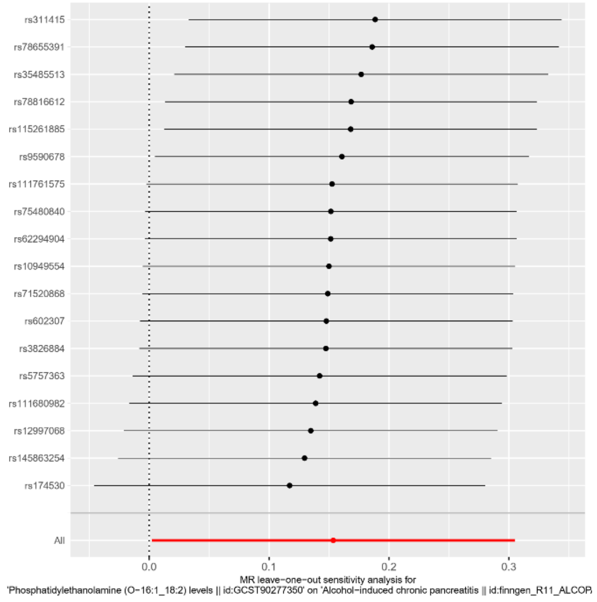


A

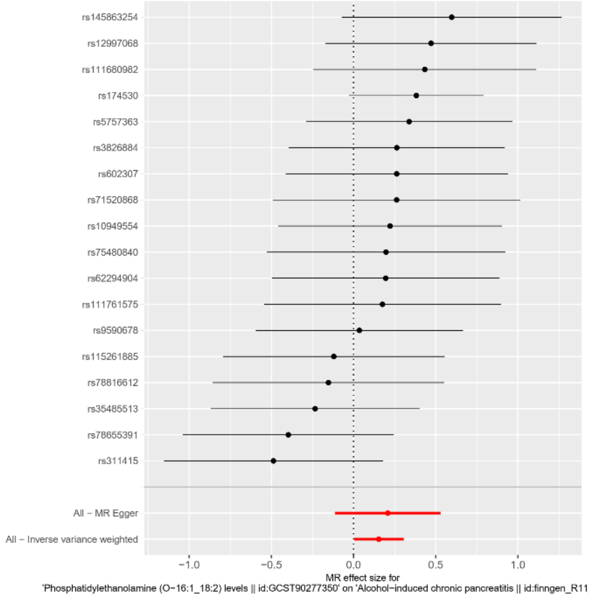


B

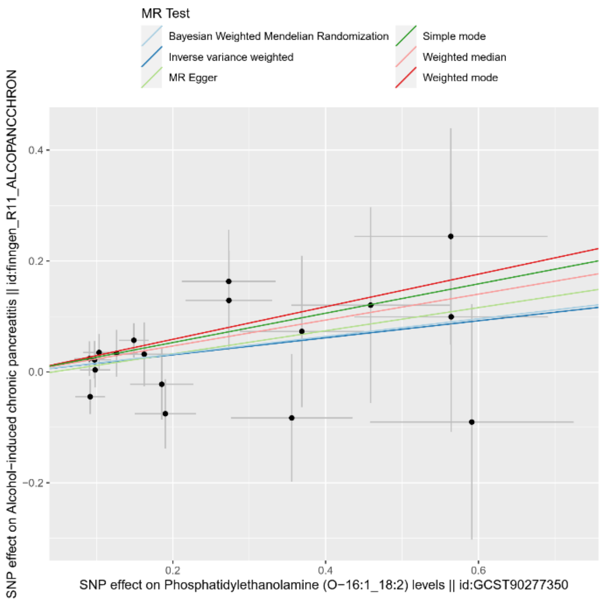


C

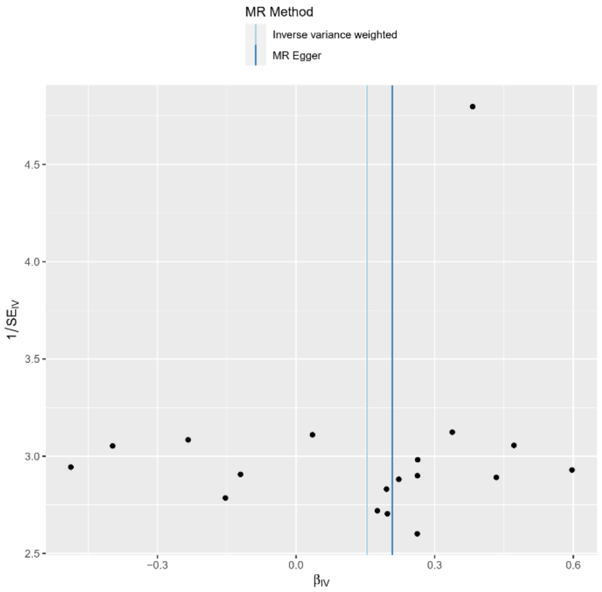


D

Figure S73 Leave-one-out analysis (A), MR effect size (B), scatter plot (C) and funnel plot (D) for Phosphatidylinositol (18:0_18:1) on alcohol-induced chronic pancreatitis

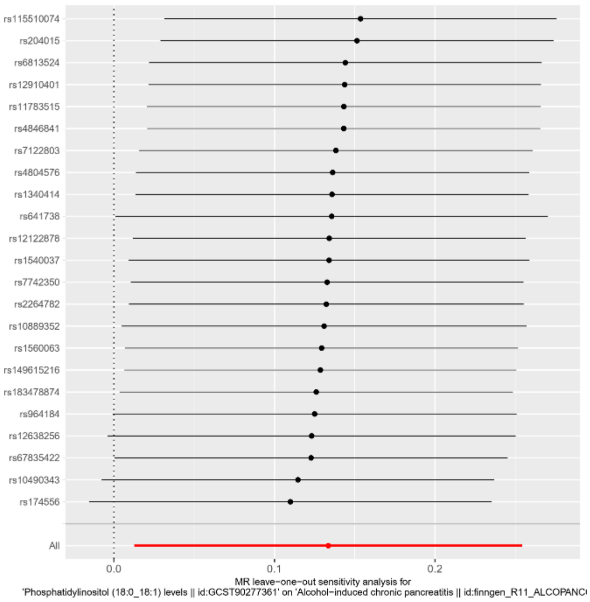


A

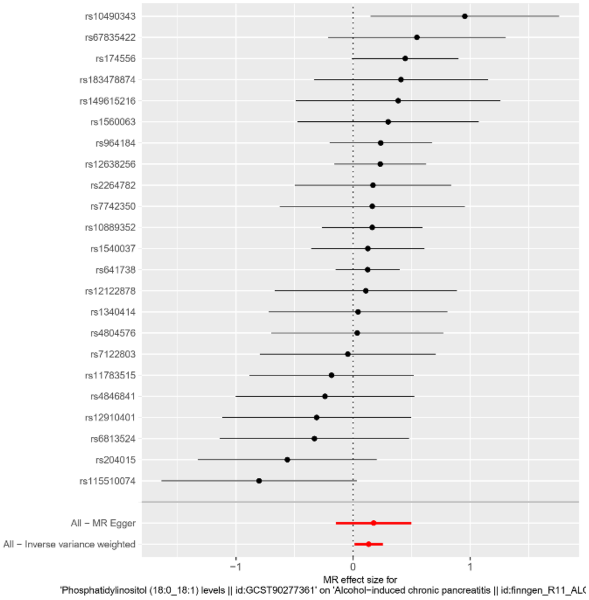


B

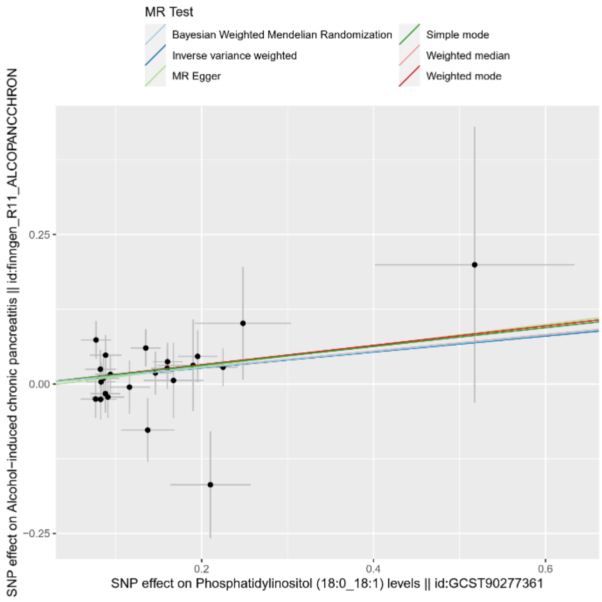


C

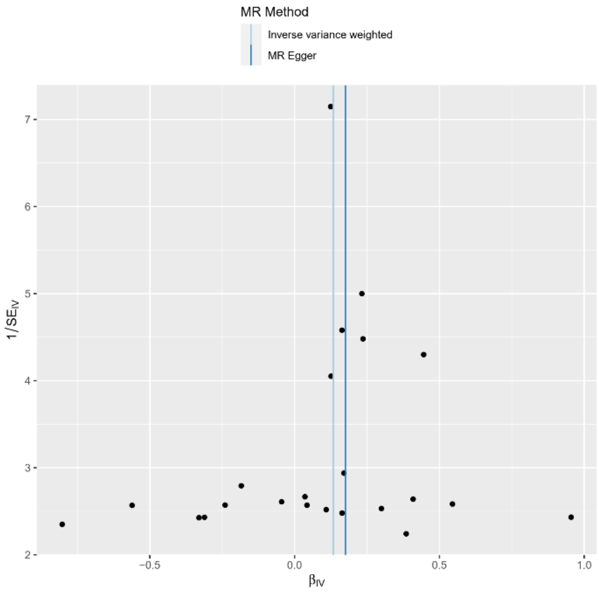


D

Figure S74 Leave-one-out analysis (A), MR effect size (B), scatter plot (C) and funnel plot (D) for Sphingomyelin (d34:2) on alcohol-induced chronic pancreatitis

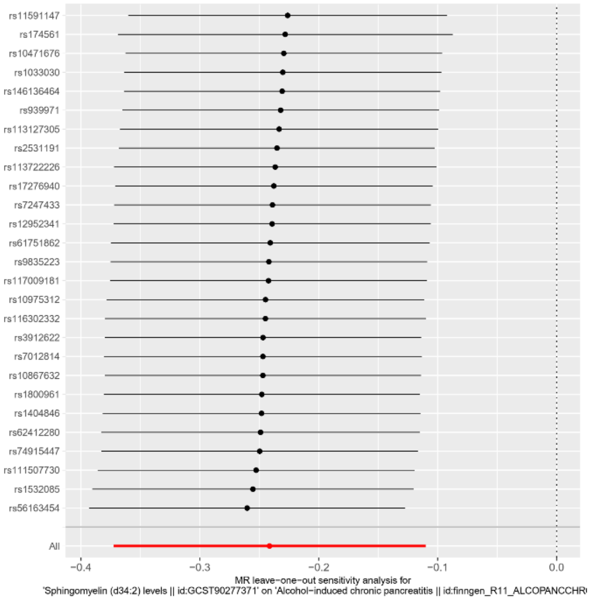


A

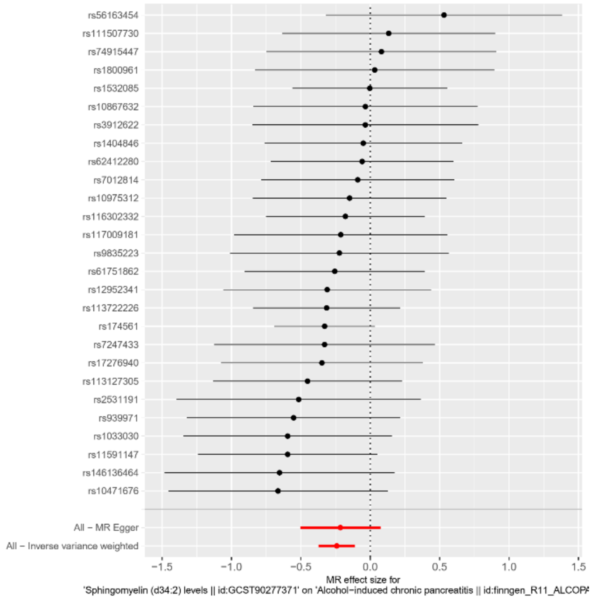


B

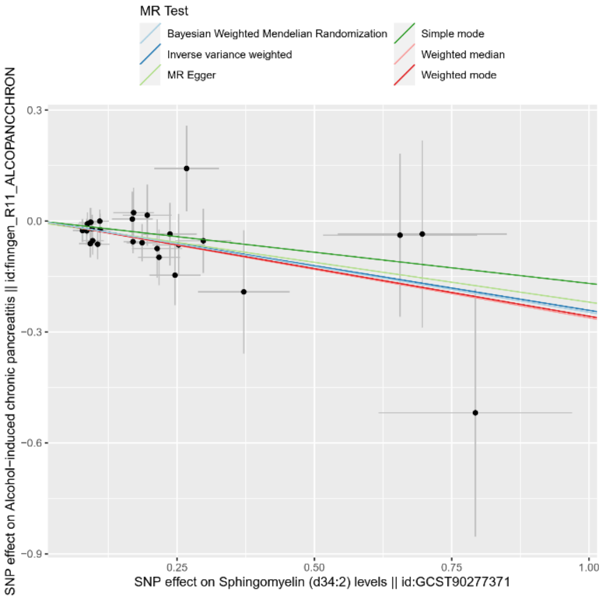


C

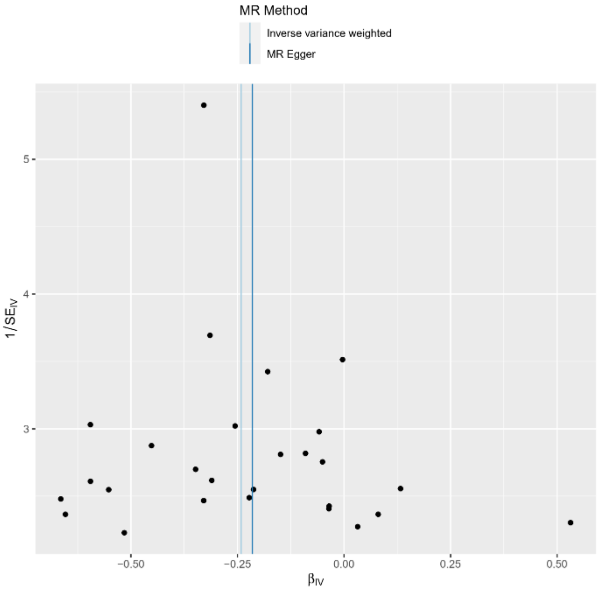


D

Figure S75 Leave-one-out analysis (A), MR effect size (B), scatter plot (C) and funnel plot (D) for Triacylglycerol (46:2) on alcohol-induced chronic pancreatitis

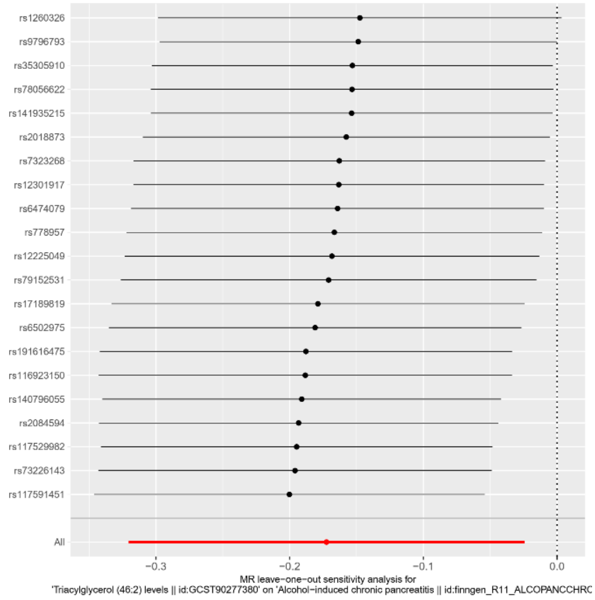


A

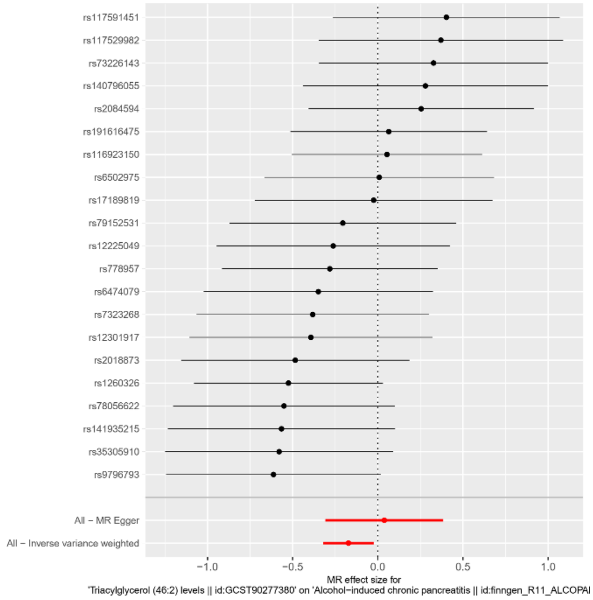


B

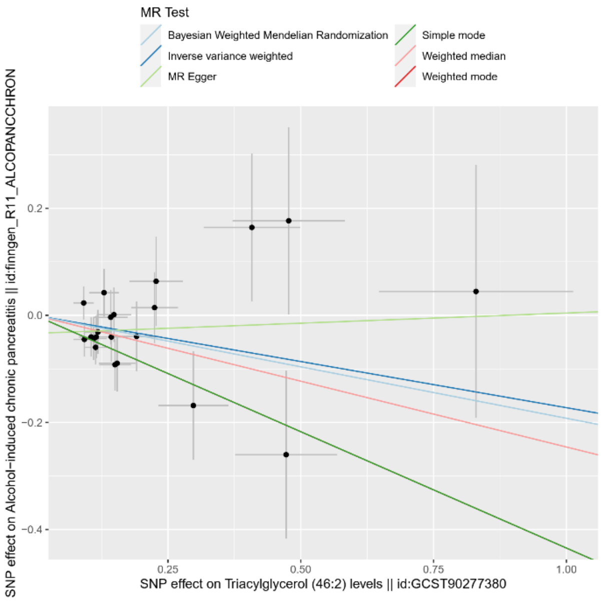


C

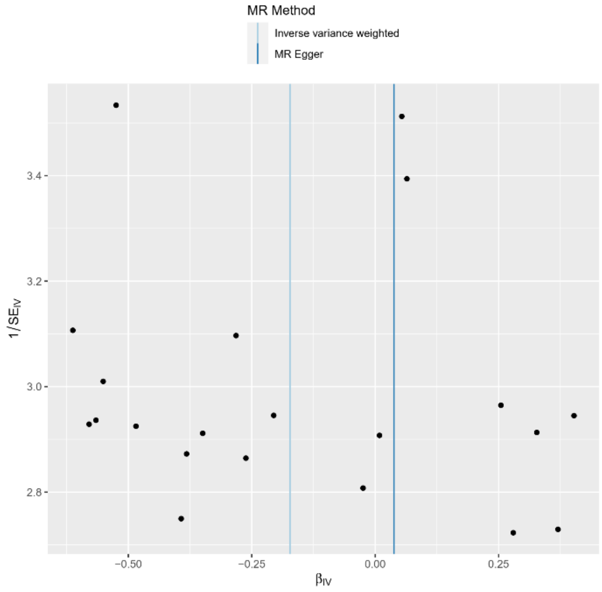


D

Figure S76 Leave-one-out analysis (A), MR effect size (B), scatter plot (C) and funnel plot (D) for Triacylglycerol (50:2) on alcohol-induced chronic pancreatitis

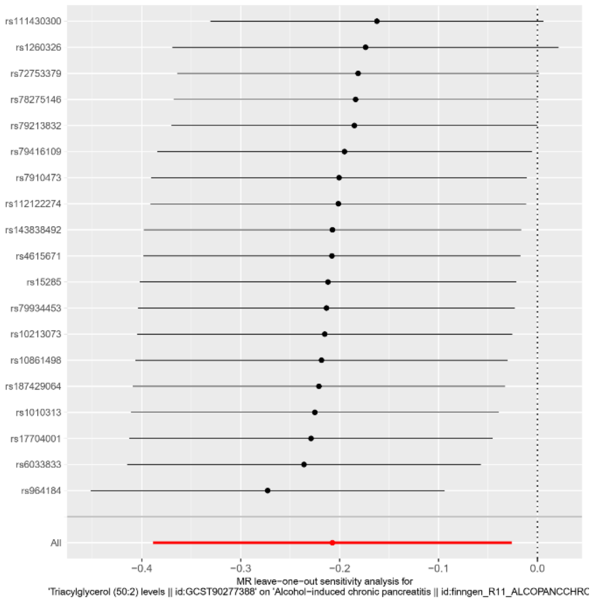


A

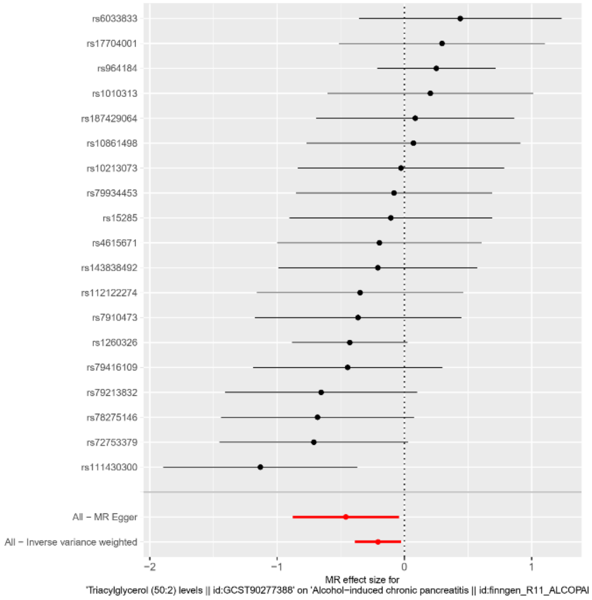


B

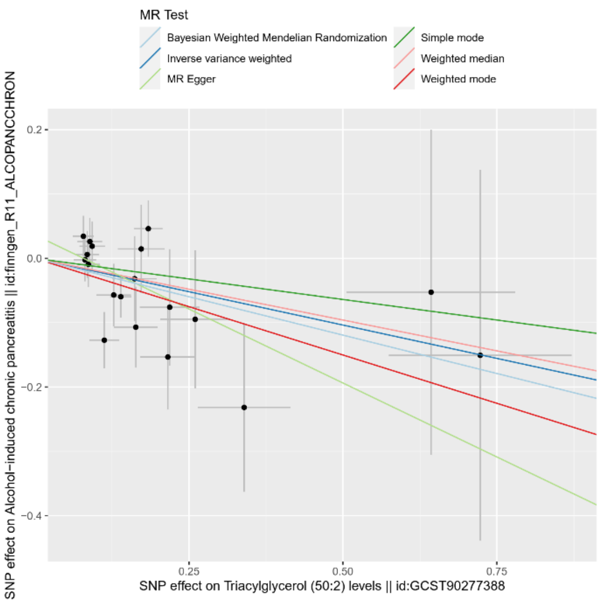


C

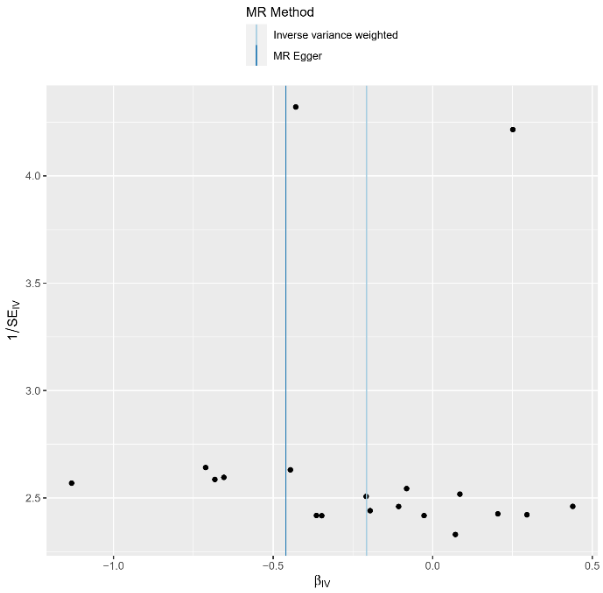


D

Figure S77 Leave-one-out analysis (A), MR effect size (B), scatter plot (C) and funnel plot (D) for Sterol ester (27:1/16:0) levels on alcohol-induced chronic pancreatitis after eliminating outliers

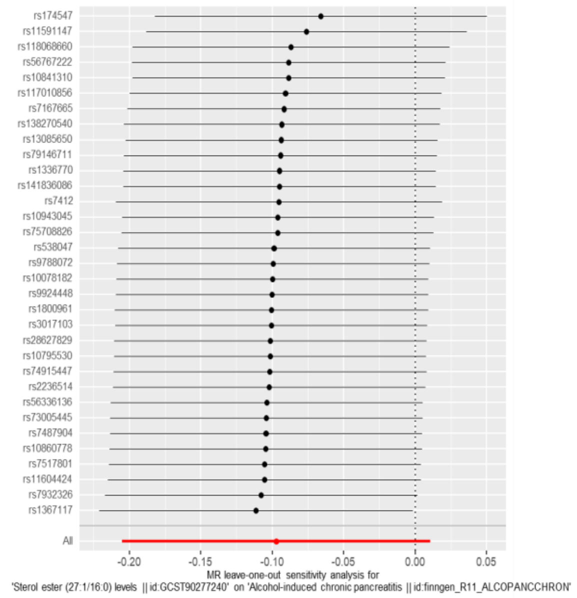


A

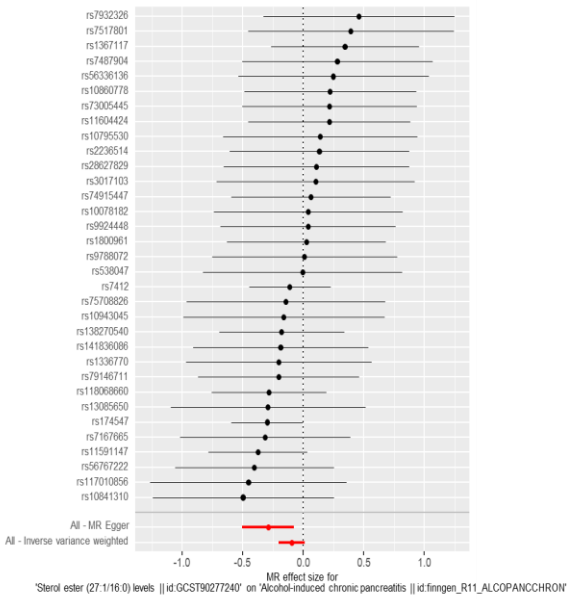


B

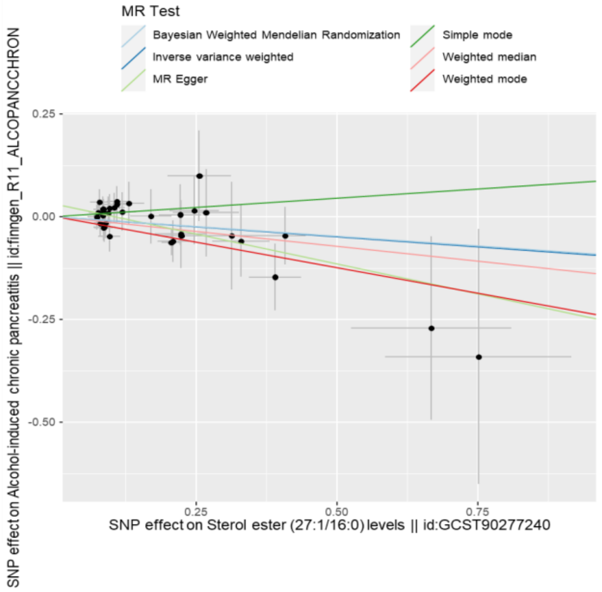


C

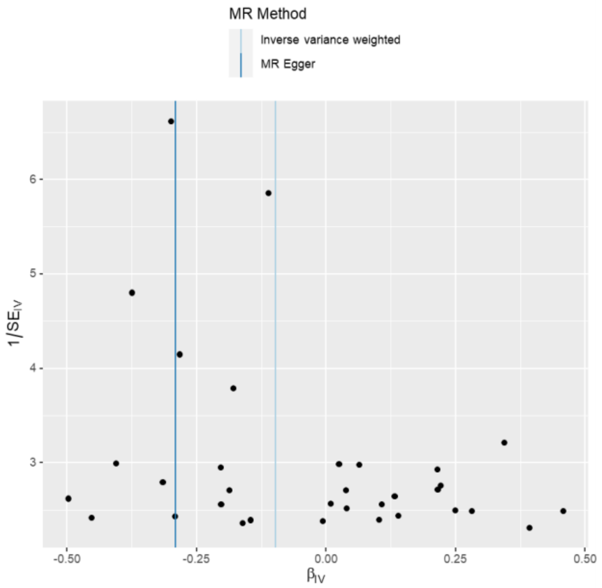


D
