# Supplementary material for: Genetically predicted causal link between the plasma lipidome and pancreatic diseases: a bidirectional Mendelian randomization study
Source: Front Nutr. 2025 Jan 15;11:1466509. doi: 10.3389/fnut.2024.1466509 (PMC11774697; doi:10.3389/fnut.2024.1466509)
Supplement: Supplementary file 16 [file Image_5.pdf]

Figure S78 Leave-one-out analysis (A), MR effect size (B), scatter plot (C) and funnel plot (D) for Diacylglycerol (16:0_18:2) levels on pancreatic cancer

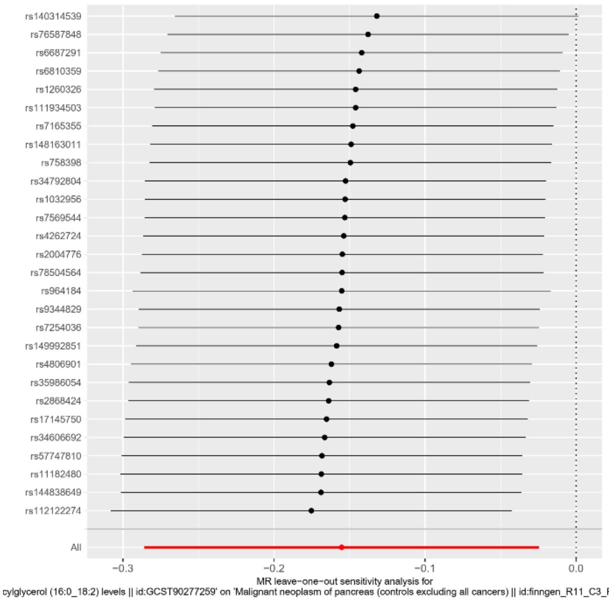

A

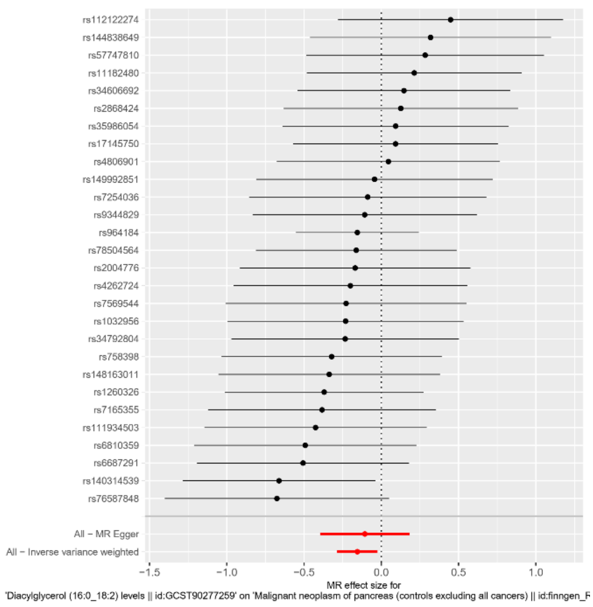

B

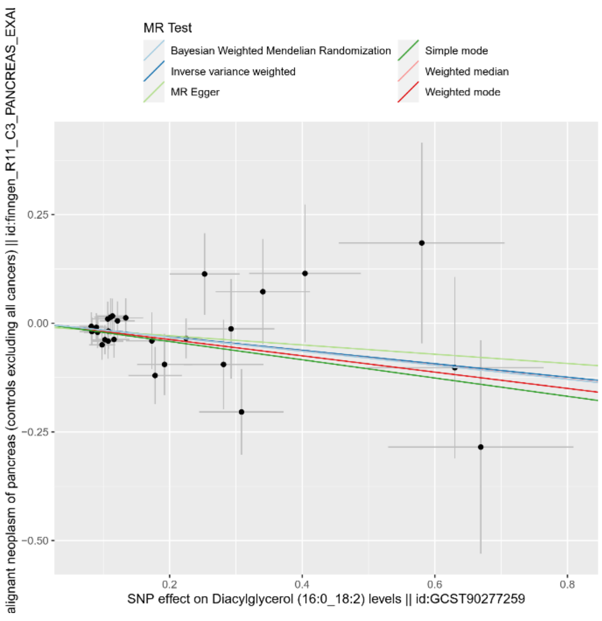

C

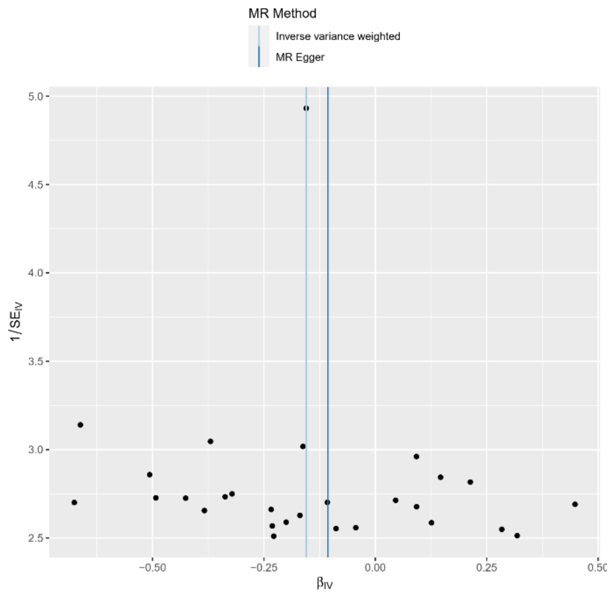

D

Figure S79 Leave-one-out analysis (A), MR effect size (B), scatter plot (C) and funnel plot (D) for Diacylglycerol (18:1_18:1) levels on pancreatic cancer

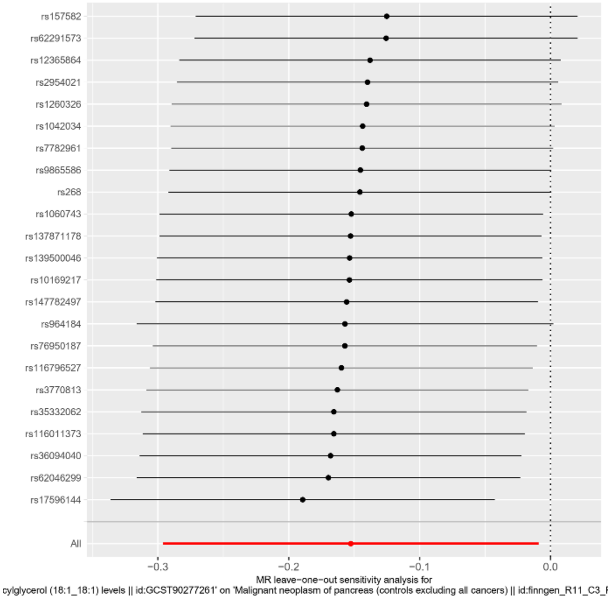

A

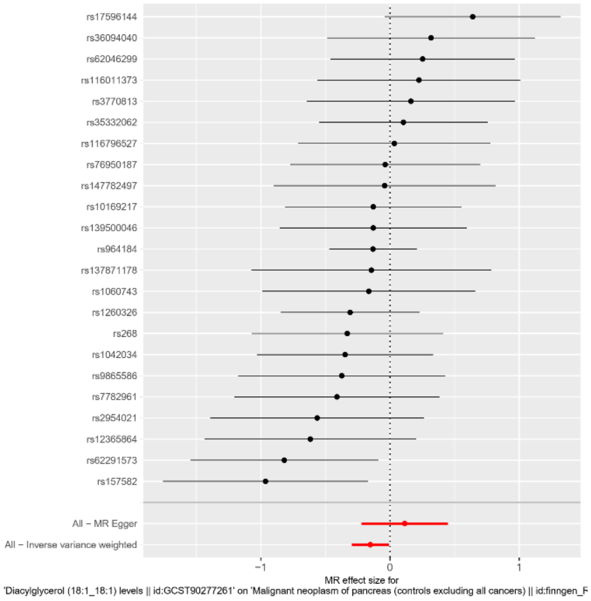

B

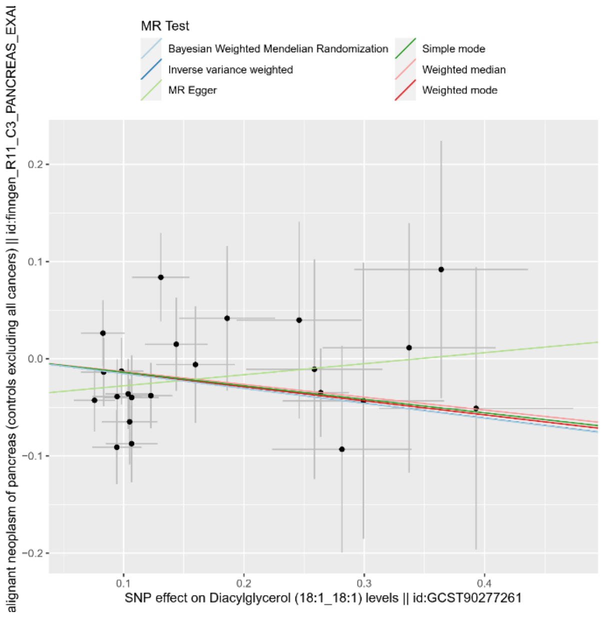

C

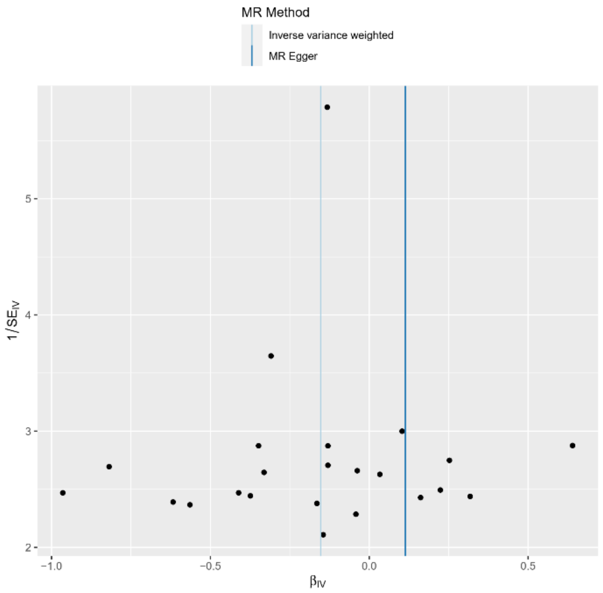

D

Figure S80 Leave-one-out analysis (A), MR effect size (B), scatter plot (C) and funnel plot (D) for Phosphatidylcholine (18:0_18:3) levels on pancreatic cancer

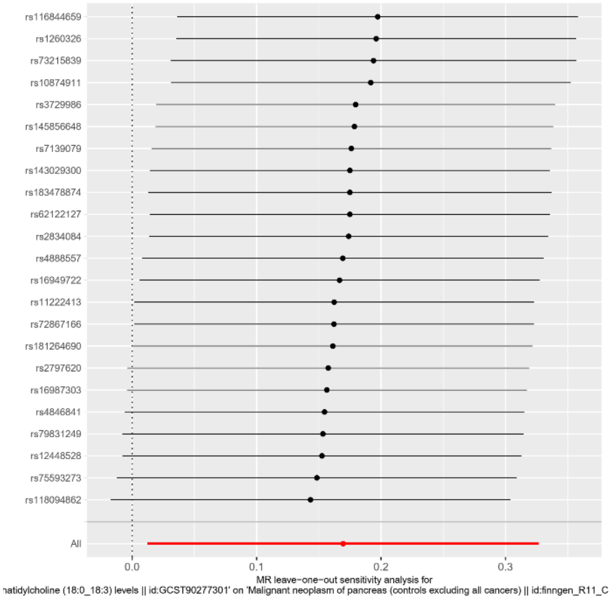

A

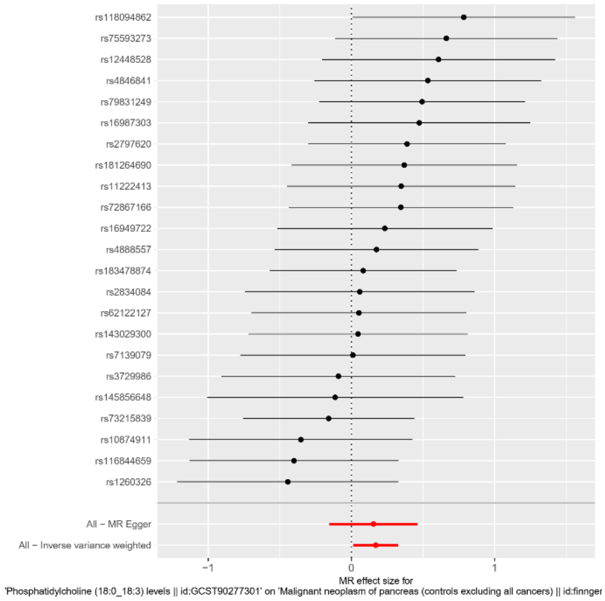

B

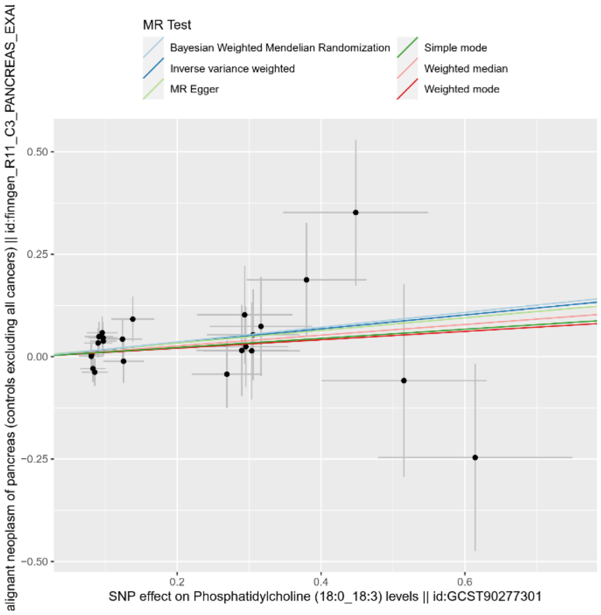

C

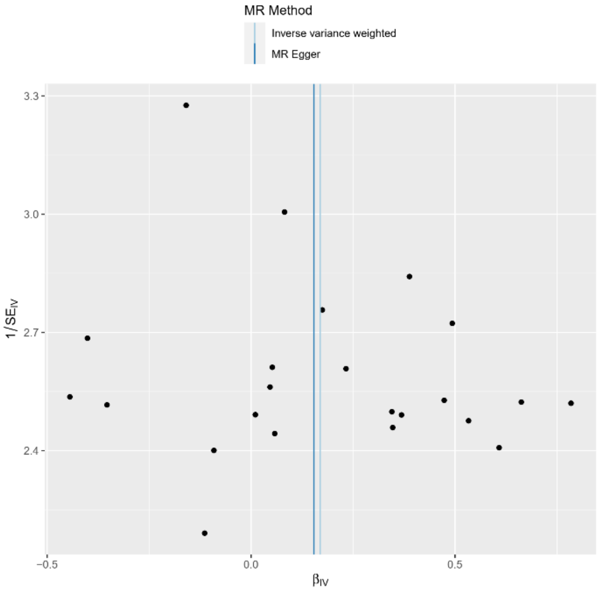

D

Figure S81 Leave-one-out analysis (A), MR effect size (B), scatter plot (C) and funnel plot (D) for Phosphatidylinositol (16:0_20:4) levels on pancreatic cancer

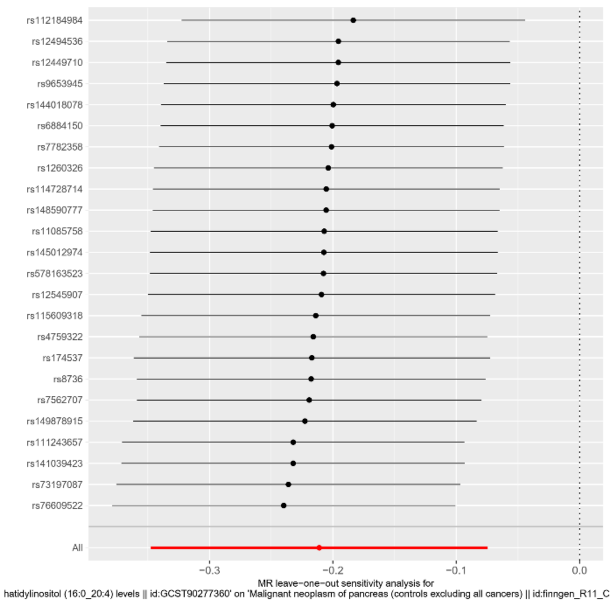

A

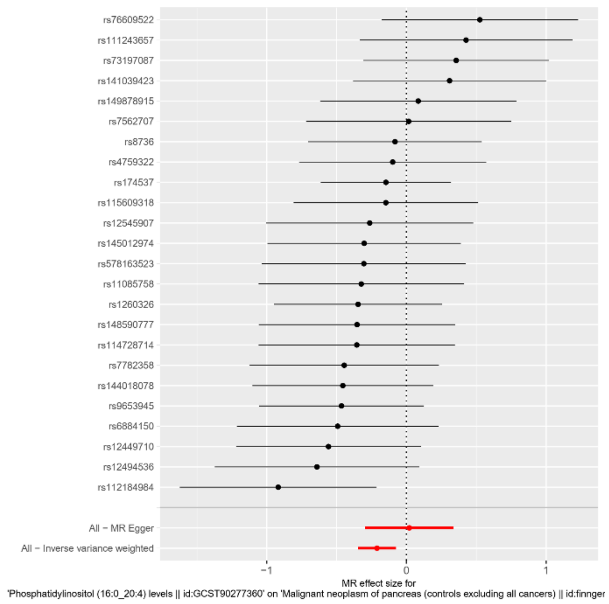

B

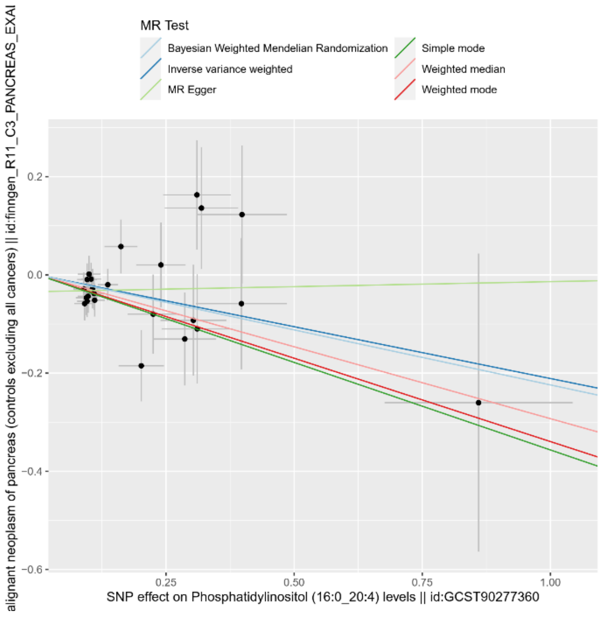

C

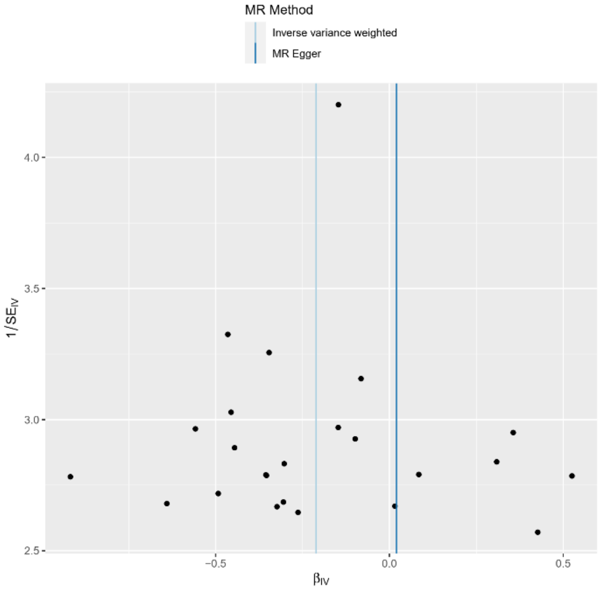

D

Figure S82 Leave-one-out analysis (A), MR effect size (B), scatter plot (C) and funnel plot (D) for Sphingomyelin (d40:1) levels on pancreatic cancer

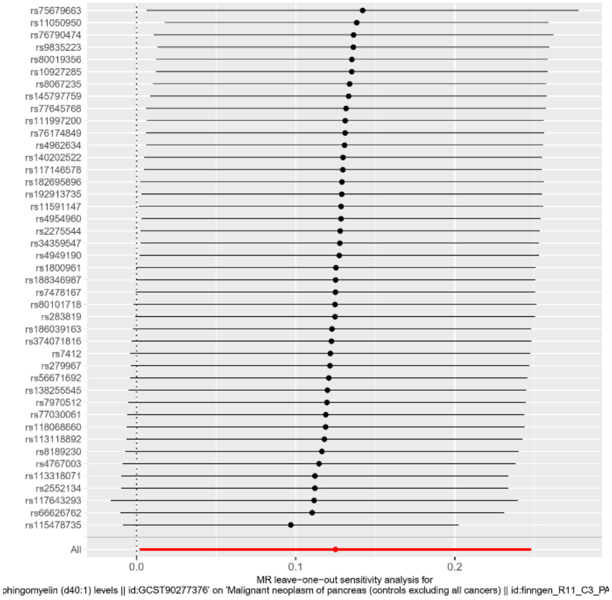

A

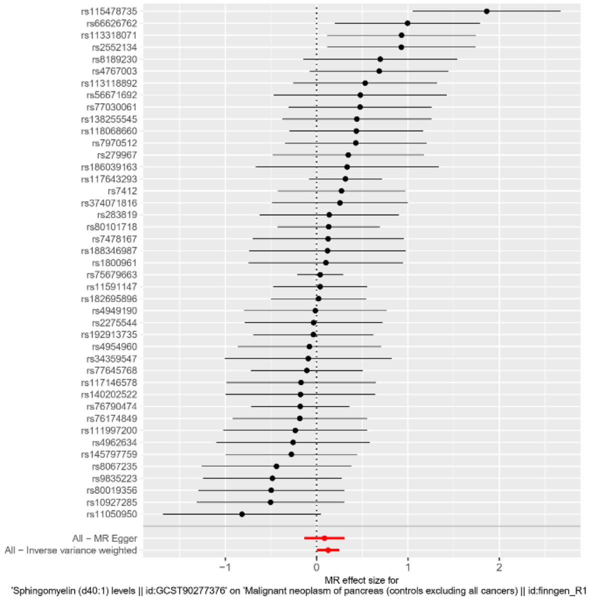

B

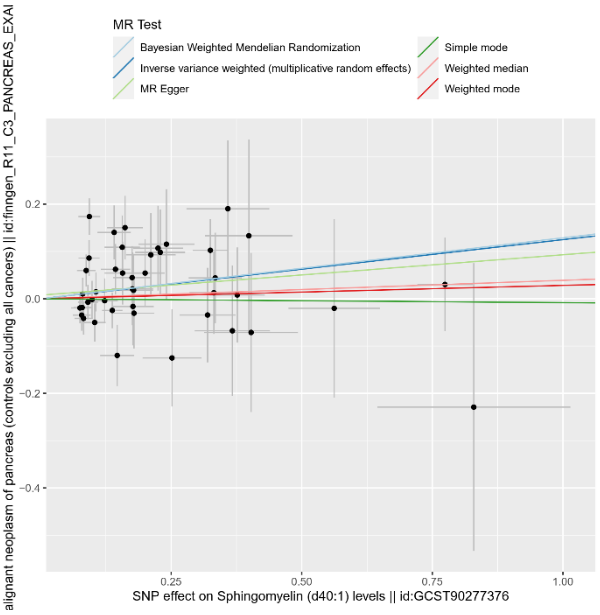

C

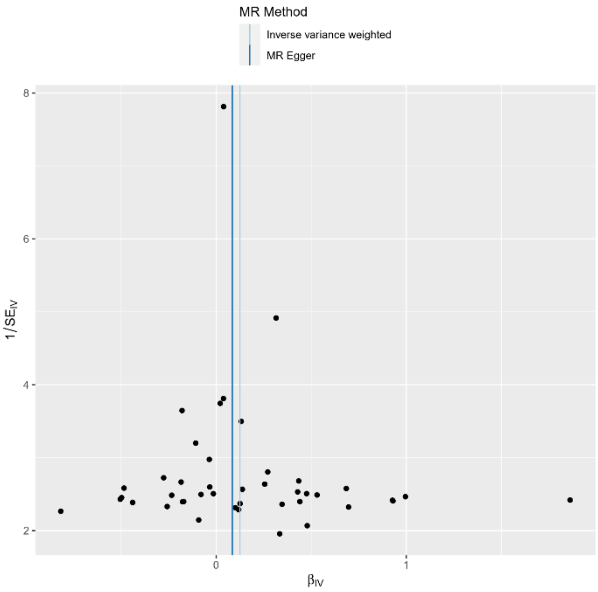

D

Figure S83 Leave-one-out analysis (A), MR effect size (B), scatter plot (C) and funnel plot (D) for Triacylglycerol (50:1) levels on pancreatic cancer

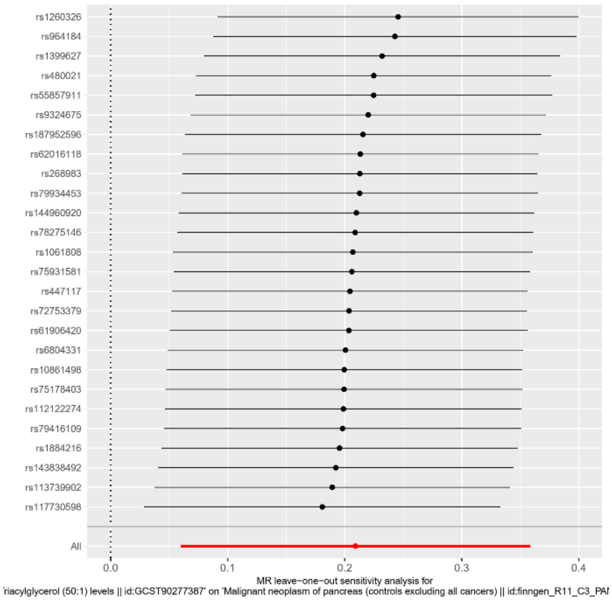

A

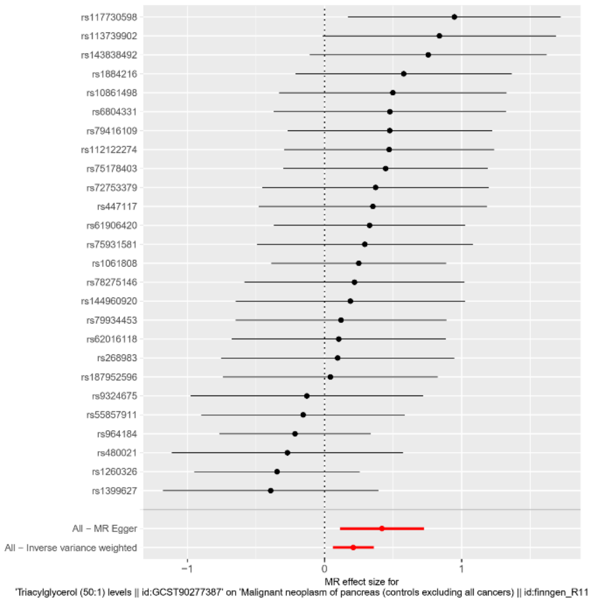

B

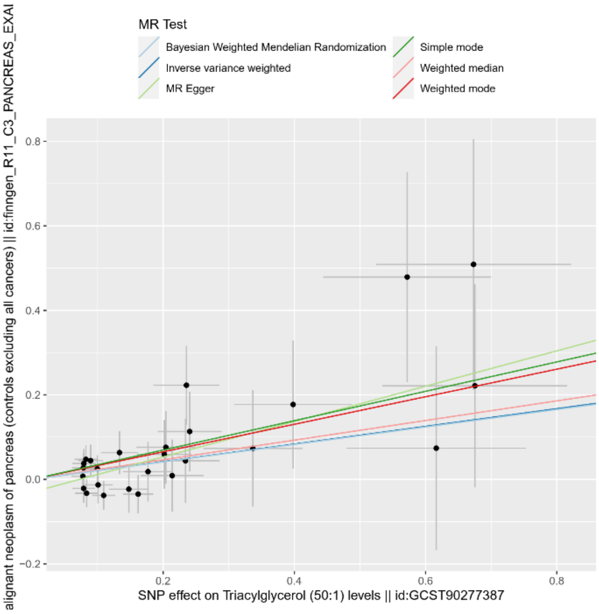

C

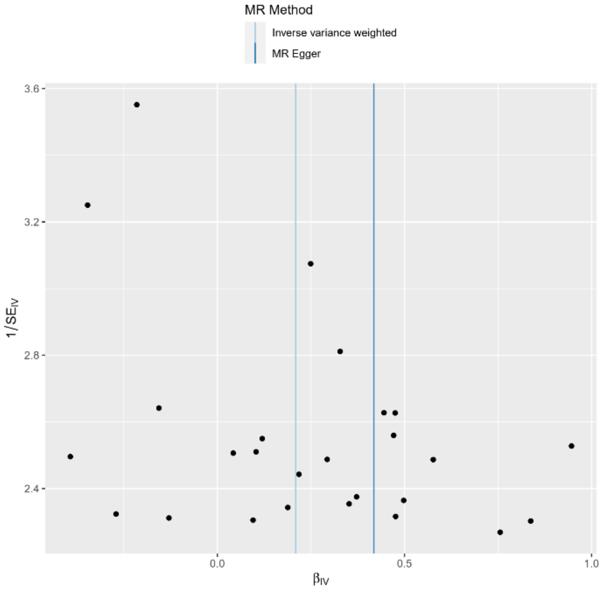

D

Figure S84 Leave-one-out analysis (A), MR effect size (B), scatter plot (C) and funnel plot (D) for Triacylglycerol (52:4) levels on pancreatic cancer

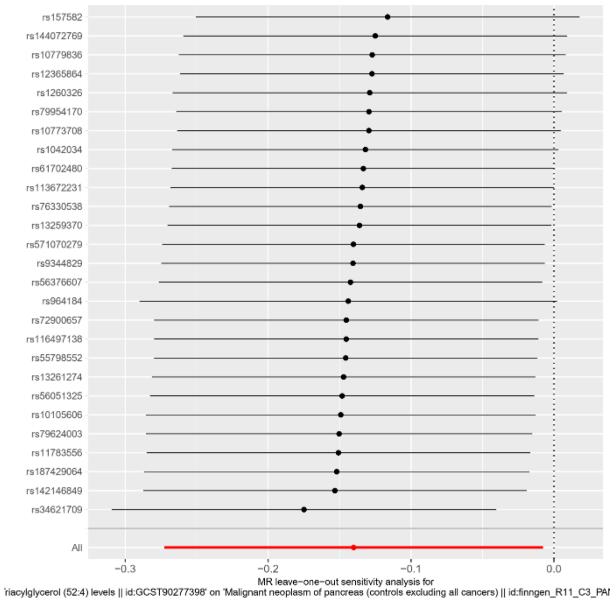

A

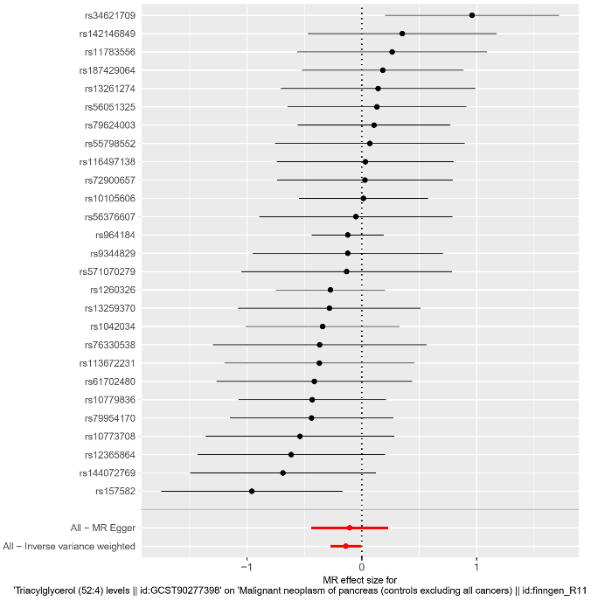

B

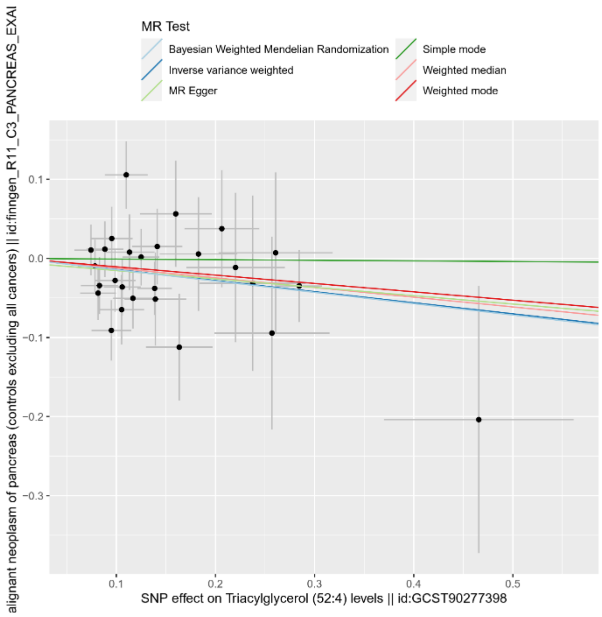

C

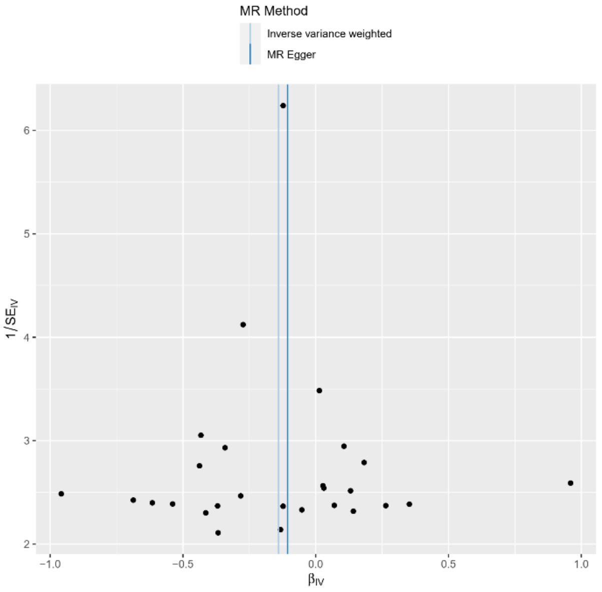

D

Figure S85 Leave-one-out analysis (A), MR effect size (B), scatter plot (C) and funnel plot (D) for Triacylglycerol (54:4) levels on pancreatic cancer

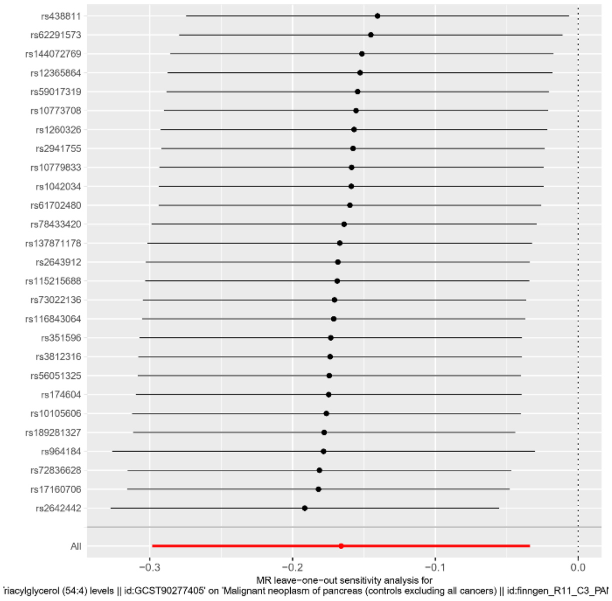

A

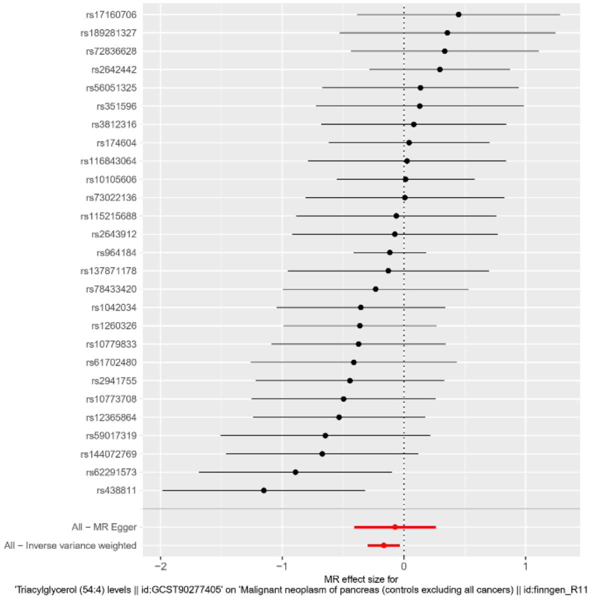

B

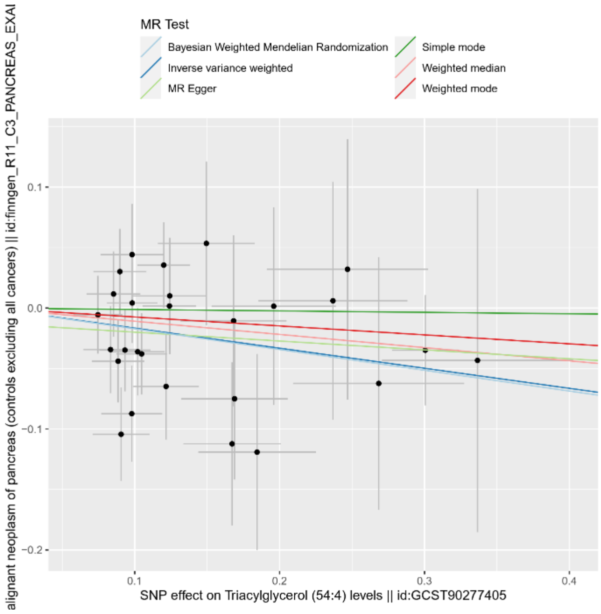

C

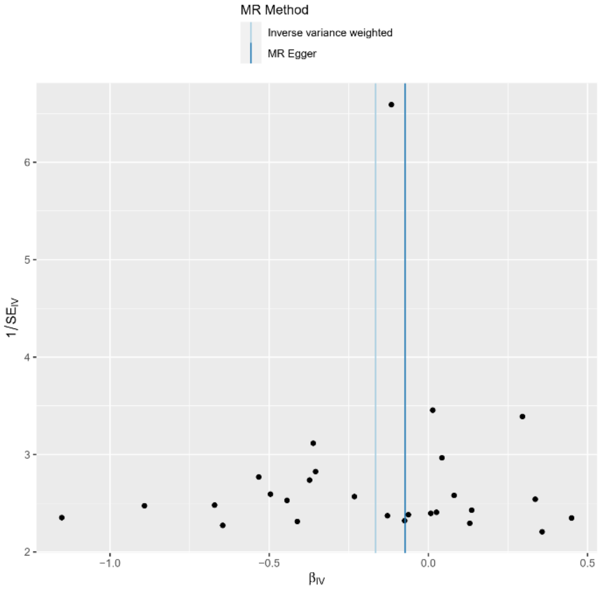

D
