# Supplementary material for: Genetically predicted causal link between the plasma lipidome and pancreatic diseases: a bidirectional Mendelian randomization study
Source: Front Nutr. 2025 Jan 15;11:1466509. doi: 10.3389/fnut.2024.1466509 (PMC11774697; doi:10.3389/fnut.2024.1466509)
Supplement: Supplementary file 17 [file Image_6.pdf]

Exposure	Outcome	nSNP	OR(95% CI)	P	OR_BWMR	P_BWMR
Sterol ester (27:1/17:0) levels	AP	27	0.9991 (0.9984 to 0.9998)	0.01227	0.9991	0.01593
Sterol ester (27:1/18:1) levels	AP	29	0.9993 (0.9987 to 0.9999)	0.01704	0.9993	0.01964
Diacylglycerol (16:0_18:2) levels	AP	28	0.9994 (0.9989 to 1.0000)	0.03369	0.9993	0.02238
Phosphatidylcholine (17:0_20:4) levels	AP	29	0.9995 (0.9991 to 1.0000)	0.03776	0.9995	0.04143
Phosphatidylcholine (18:1_18:3) levels	AP	16	0.9991 (0.9983 to 1.0000)	0.04130	0.9991	0.04602
Phosphatidylcholine (18:1_20:2) levels	AP	22	1.0005 (1.0001 to 1.0010)	0.02349	1.0005	0.02845
Phosphatidylinositol (18:1_18:1) levels	AP	23	1.0006 (1.0001 to 1.0012)	0.03058	1.0006	0.04812
Phosphatidylinositol (18:1_20:4) levels	AP	25	0.9993 (0.9986 to 1.0000)	0.04281	0.9992	0.02676
Sphingomyelin (d34:2) levels	AP	27	0.9993 (0.9987 to 0.9998)	0.01102	0.9992	0.01567
Triacylglycerol (48:0) levels	AP	18	0.9993 (0.9987 to 1.0000)	0.04835	0.9993	0.05358
Triacylglycerol (50:4) levels	AP	31	0.9993 (0.9986 to 0.9999)	0.01863	0.9992	0.02018
Triacylglycerol (51:4) levels	AP	20	0.9994 (0.9988 to 0.9999)	0.03012	0.9993	0.02250
Triacylglycerol (52:4) levels	AP	27	0.9992 (0.9985 to 0.9999)	0.01699	0.9991	0.01350
Triacylglycerol (52:5) levels	AP	25	0.9987 (0.9980 to 0.9993)	0.00009	0.9987	0.00015

0.998 1 1.0012

Figure S86 Forest plot to visualize the causal effect of plasma lipidome on AP of UK Biobank using the inverse variance weighted method. The accompanying BWMR tests are shown together. AP, acute pancreatitis; SNP, single nucleotide polymorphisms; OR, odds ratio; CI, confidence interval; BWMR, Bayesian weighted Mendelian randomization**.**

Exposure	Outcome	nSNP	OR(95% CI)	P	OR_BWMR	P_BWMR
Sterol ester (27:1/18:1) levels	CP	29	0.9997 (0.9995 to 1.0000)	0.03838	0.9997	0.03723
Sterol ester (27:1/20:2) levels	CP	27	0.9997 (0.9995 to 0.9999)	0.01104	0.9997	0.01251
Ceramide (d40:2) levels	CP	20	1.0003 (1.0000 to 1.0006)	0.04365	1.0003	0.06177
Phosphatidylcholine (16:0_20:5) levels	CP	21	0.9997 (0.9994 to 1.0000)	0.02506	0.9997	0.02839
Phosphatidylcholine (18:0_18:1) levels	CP	29	0.9997 (0.9994 to 0.9999)	0.01841	0.9996	0.01611
Phosphatidylcholine (O−18:1_20:4) levels	CP	22	0.9997 (0.9994 to 0.9999)	0.01139	0.9997	0.01497
Phosphatidylcholine (O−18:2_18:1) levels	CP	17	1.0004 (1.0001 to 1.0008)	0.01315	1.0004	0.01475
Phosphatidylinositol (18:0_18:2) levels	CP	33	1.0002 (1.0001 to 1.0004)	0.00771	1.0002	0.00671
Sphingomyelin (d38:1) levels	CP	36	1.0003 (1.0001 to 1.0005)	0.00968	1.0003	0.01299

0.9993 1 1.00076

Figure S87 Forest plot to visualize the causal effect of plasma lipidome on CP of UK Biobank using the inverse variance weighted method. The accompanying BWMR tests are shown together. CP, chronic pancreatitis; SNP, single nucleotide polymorphisms OR, odds ratio; CI, confidence interval; BWMR, Bayesian weighted Mendelian randomization**.**
