# Supplementary material for: Genetically predicted causal link between the plasma lipidome and pancreatic diseases: a bidirectional Mendelian randomization study
Source: Front Nutr. 2025 Jan 15;11:1466509. doi: 10.3389/fnut.2024.1466509 (PMC11774697; doi:10.3389/fnut.2024.1466509)
Supplement: Supplementary file 18 [file Image_7.pdf]

Figure S88 Leave-one-out analysis (A), MR effect size (B), scatter plot (C) and funnel plot (D) for Sterol ester (27:1/17:0) levels on acute pancreatitis for UK Biobank

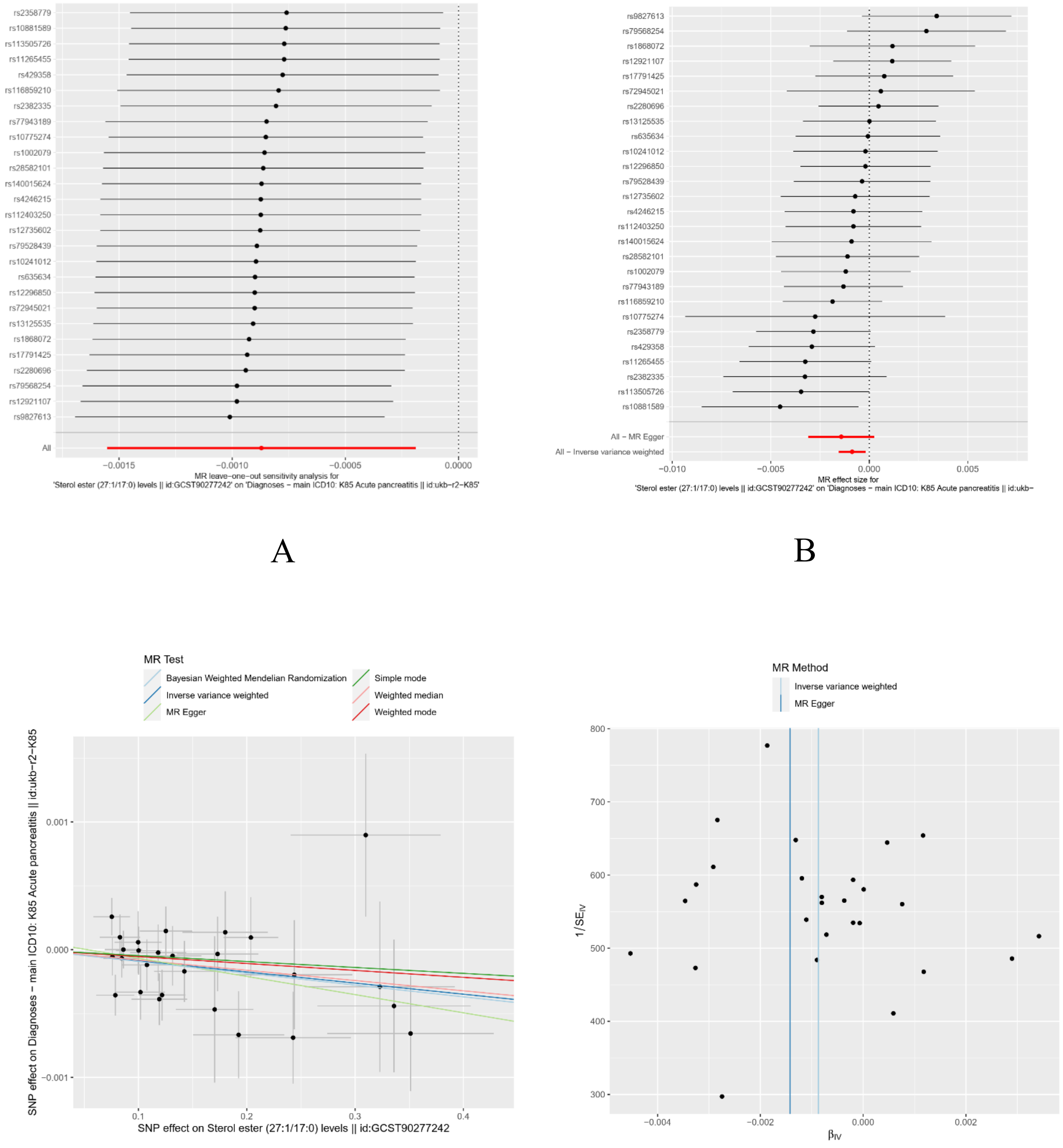

C

D

Figure S89 Leave-one-out analysis (A), MR effect size (B), scatter plot (C) and funnel plot (D) for Sterol ester (27:1/18:1) levels on acute pancreatitis for UK Biobank

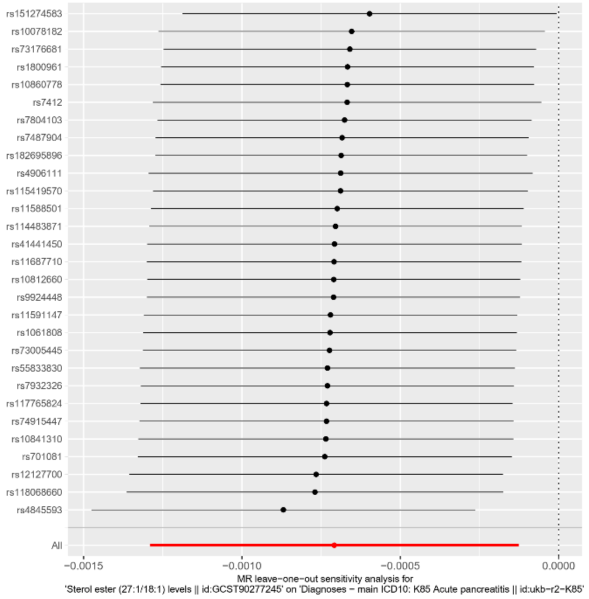

A

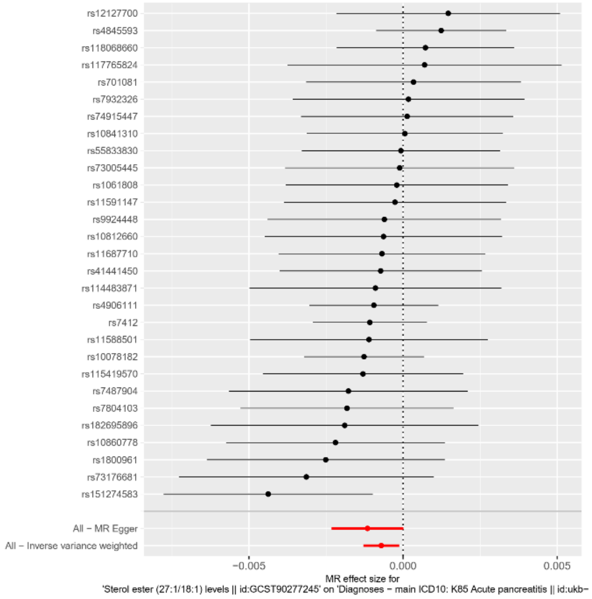

B

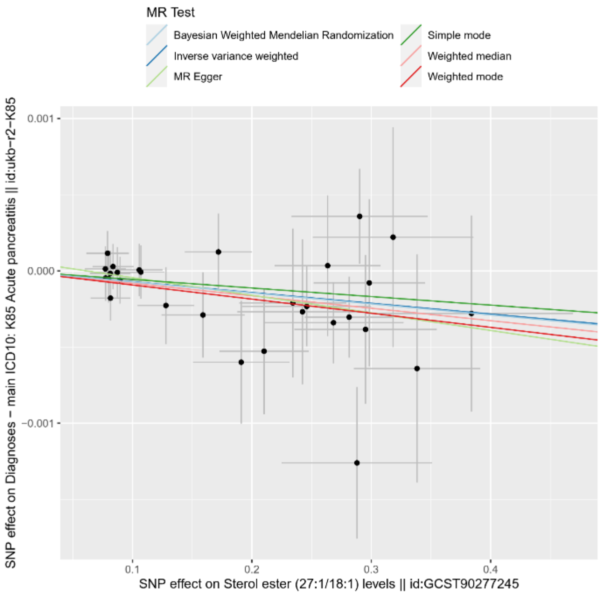

C

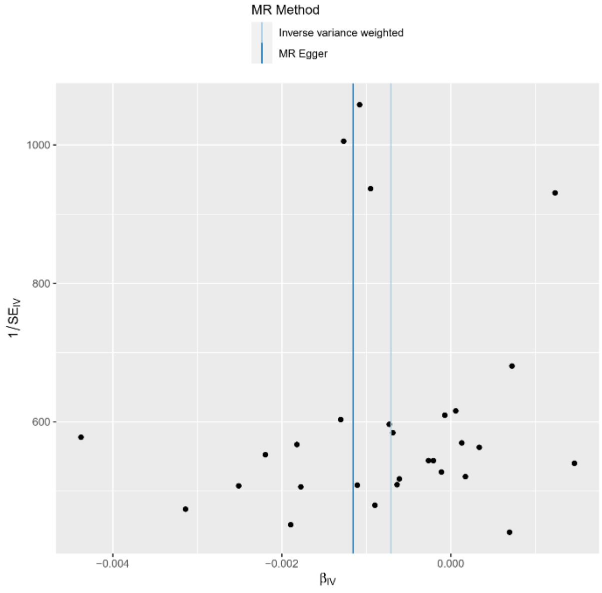

D

Figure S90 Leave-one-out analysis (A), MR effect size (B), scatter plot (C) and funnel plot (D) for Diacylglycerol (16:0_18:2) levels on acute pancreatitis for UK Biobank

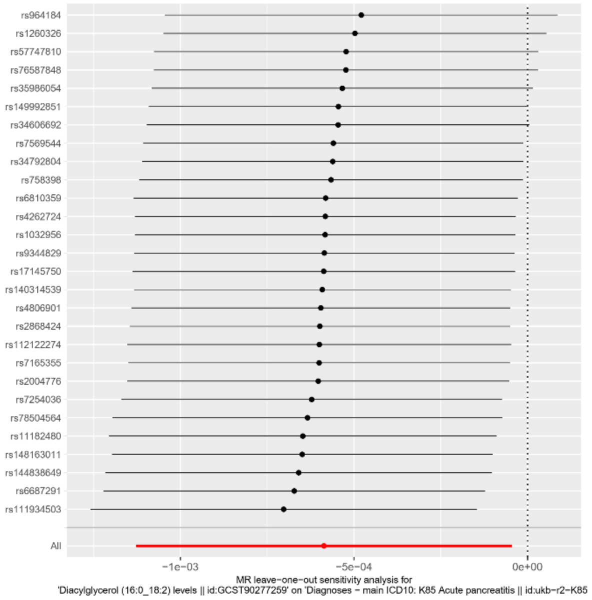

A

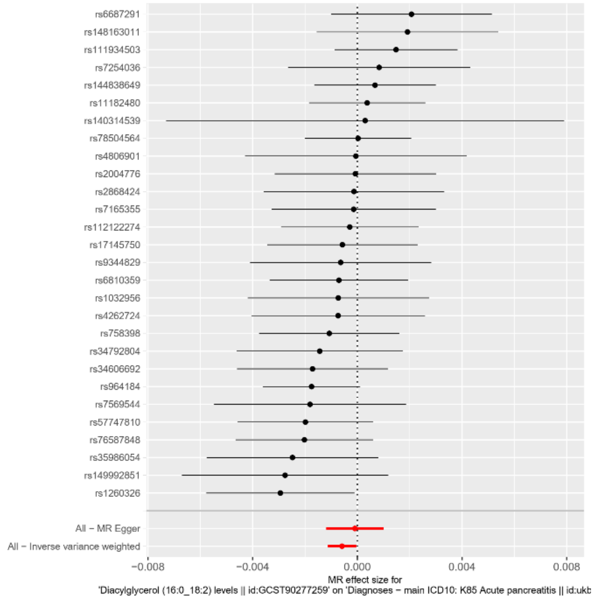

B

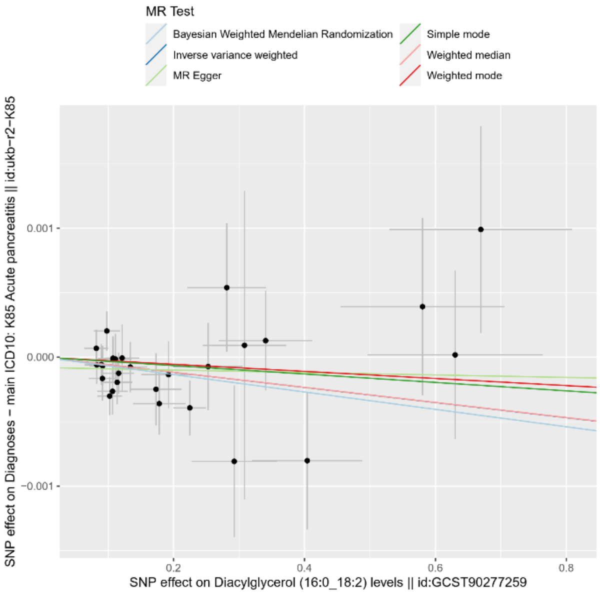

C

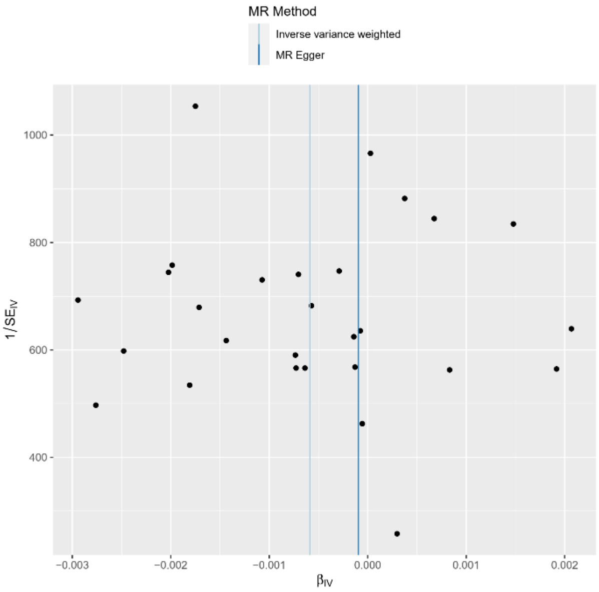

D

Figure S91 Leave-one-out analysis (A), MR effect size (B), scatter plot (C) and funnel plot (D) for Phosphatidylcholine (17:0_20:4) levels on acute pancreatitis for UK Biobank

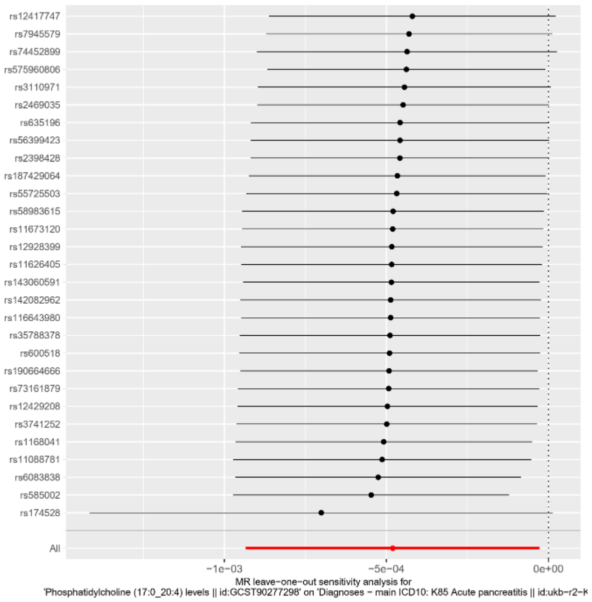

A

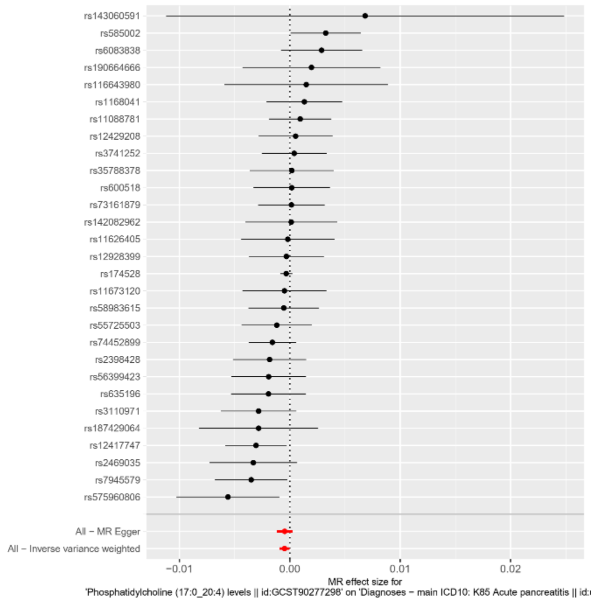

B

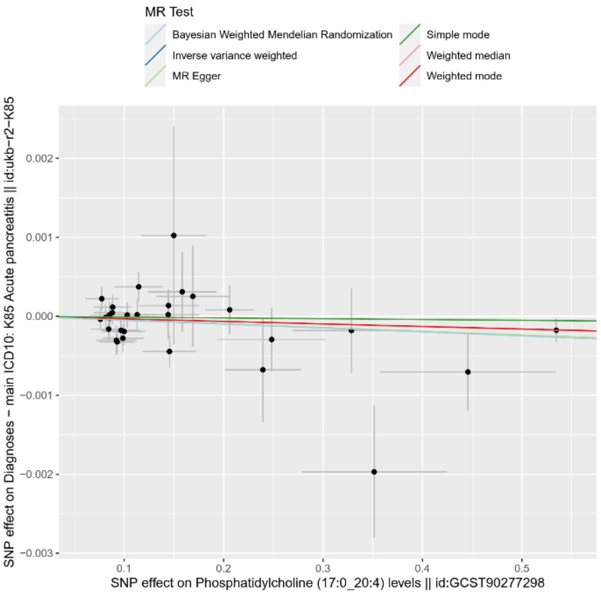

C

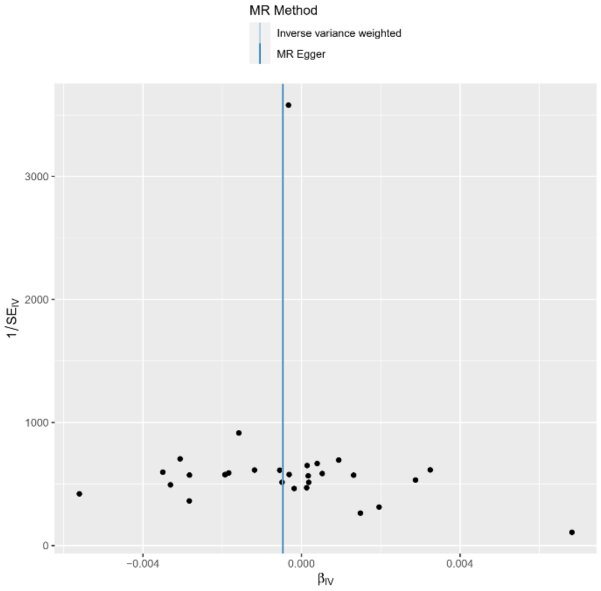

D

Figure S92 Leave-one-out analysis (A), MR effect size (B), scatter plot (C) and funnel plot (D) for Phosphatidylcholine (18:1_18:3) levels on acute pancreatitis for UK Biobank

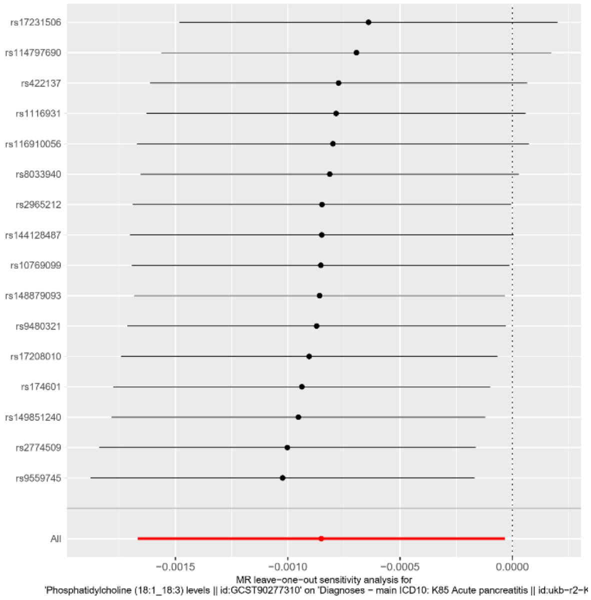

A

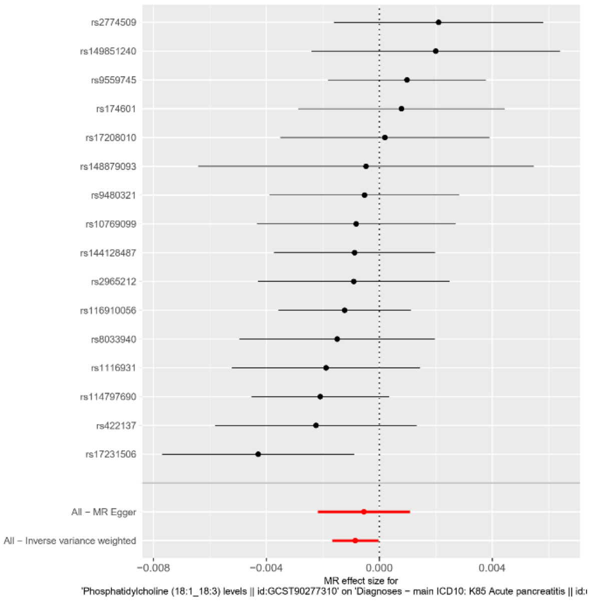

B

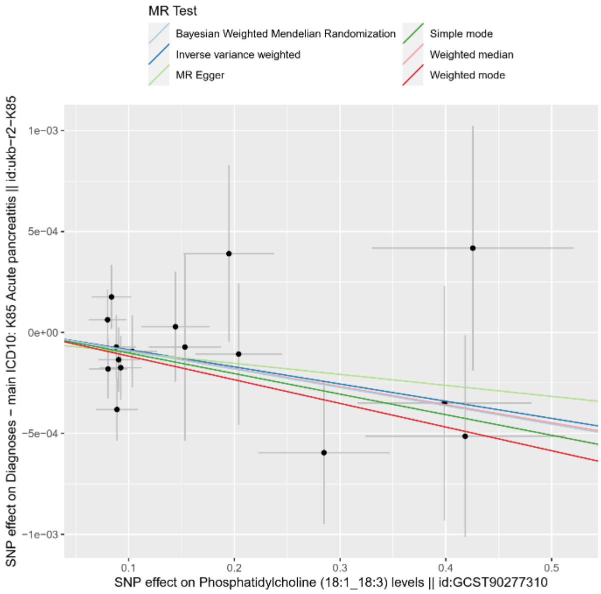

C

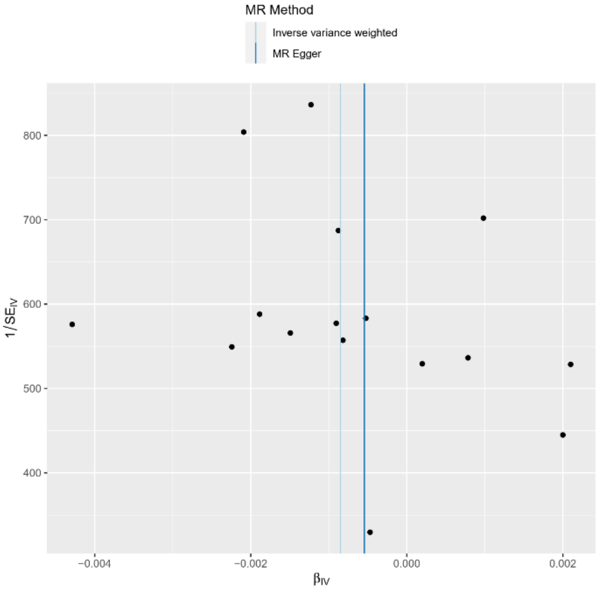

D

Figure S93 Leave-one-out analysis (A), MR effect size (B), scatter plot (C) and funnel plot (D) for Phosphatidylcholine (18:1_20:2) levels on acute pancreatitis for UK Biobank

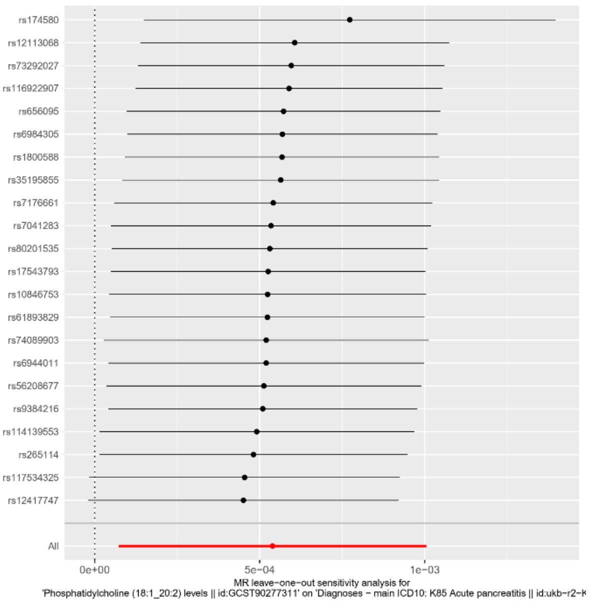

A

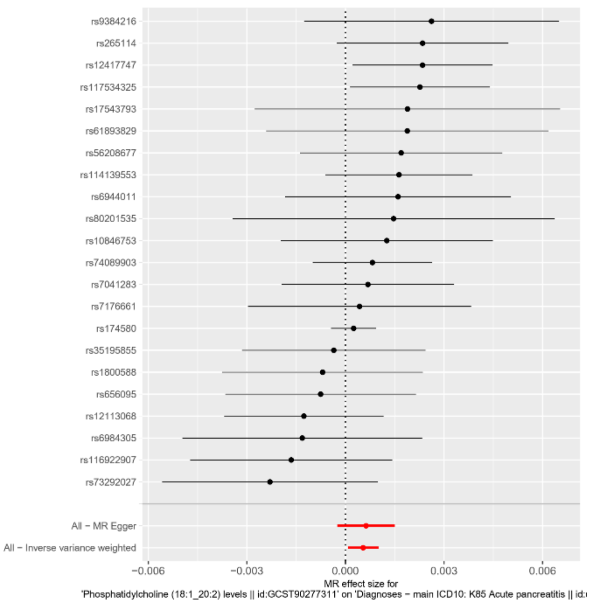

B

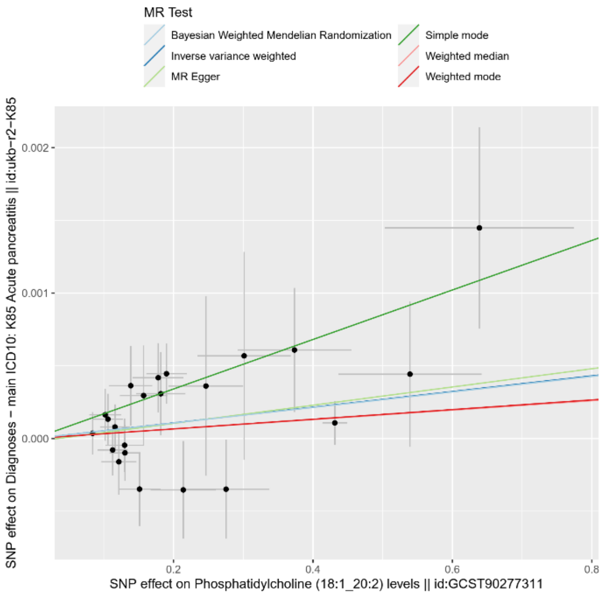

C

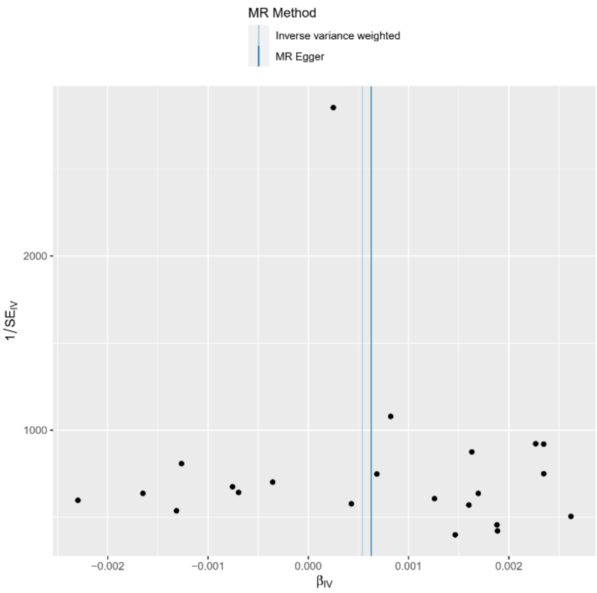

D

Figure S94 Leave-one-out analysis (A), MR effect size (B), scatter plot (C) and funnel plot (D) for Phosphatidylinositol (18:1_18:1) levels on acute pancreatitis for UK Biobank

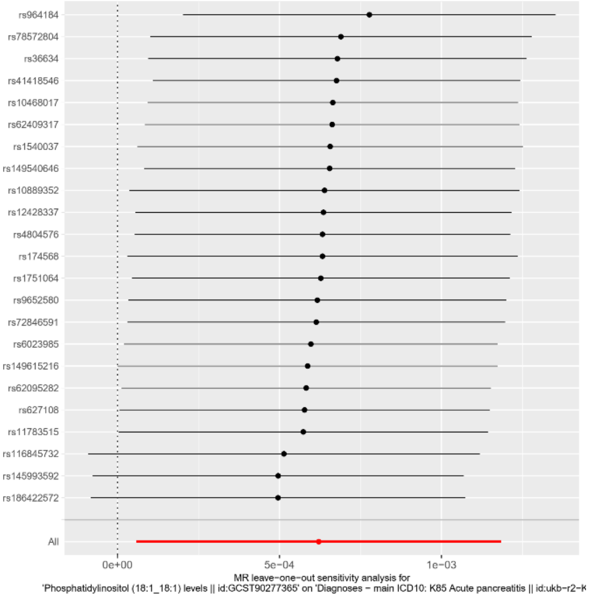

A

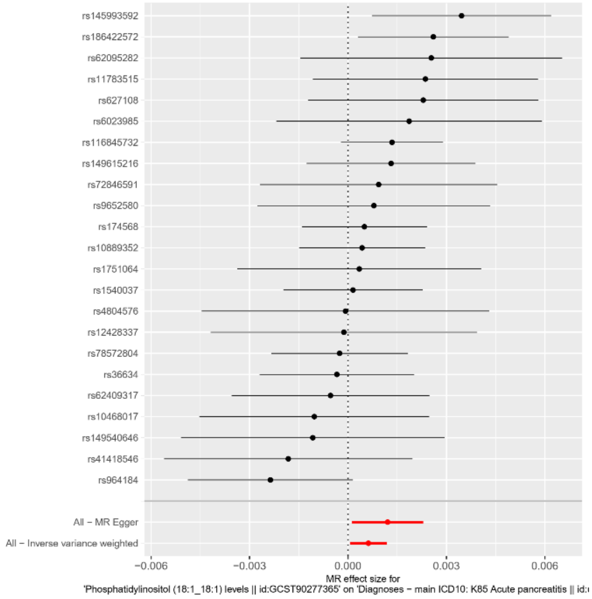

B

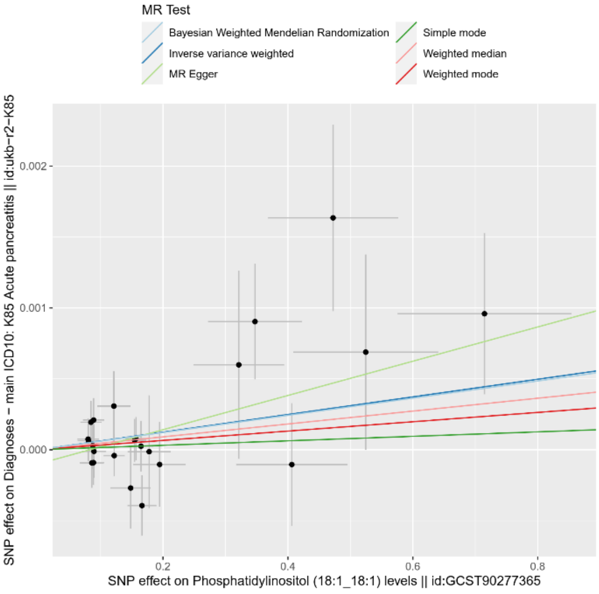

C

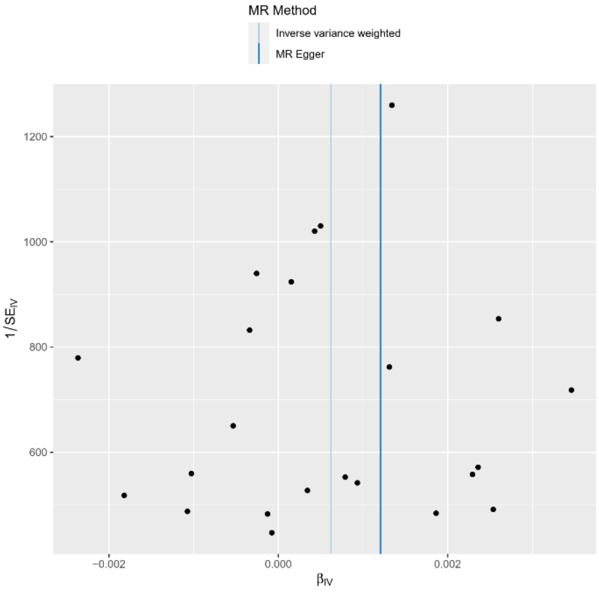

D

Figure S95 Leave-one-out analysis (A), MR effect size (B), scatter plot (C) and funnel plot (D) for Phosphatidylinositol (18:1_20:4) levels on acute pancreatitis for UK Biobank

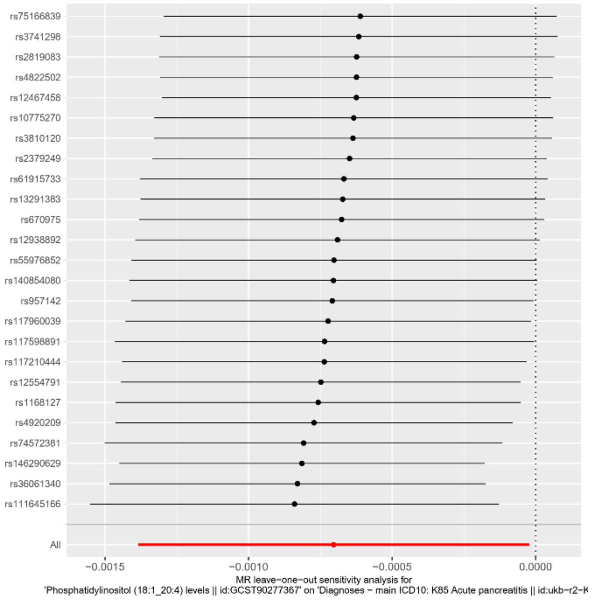

A

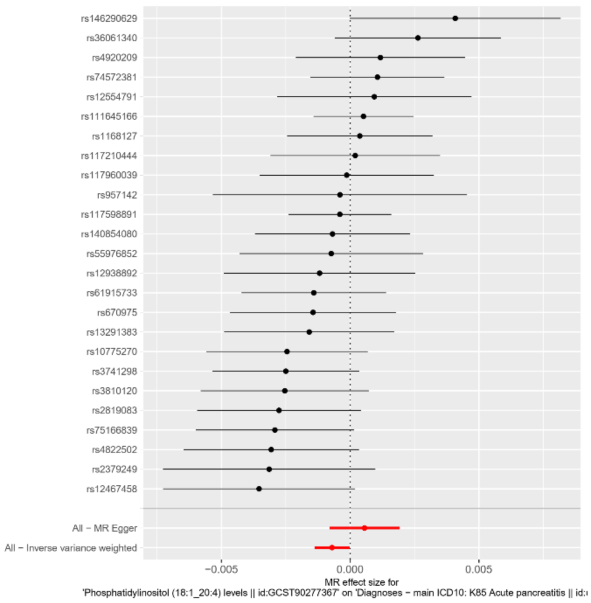

B

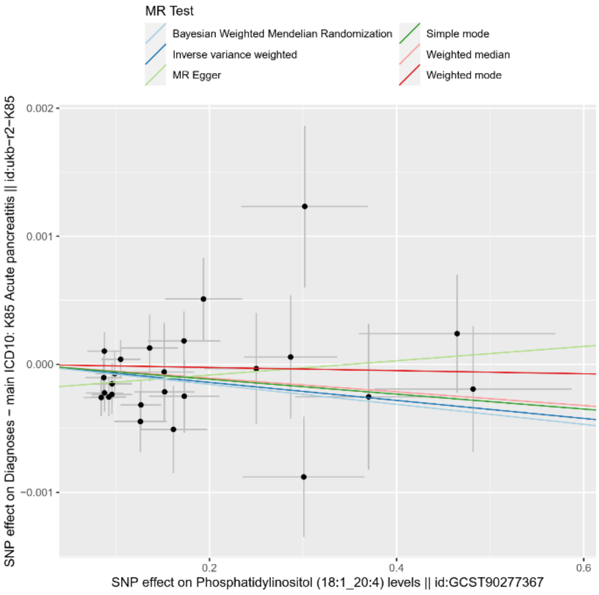

C

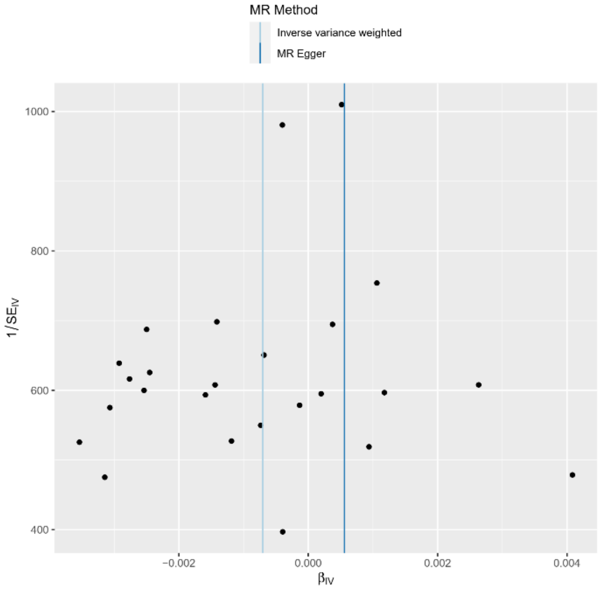

D

Figure S96 Leave-one-out analysis (A), MR effect size (B), scatter plot (C) and funnel plot (D) for Sphingomyelin (d34:2) levels on acute pancreatitis for UK Biobank

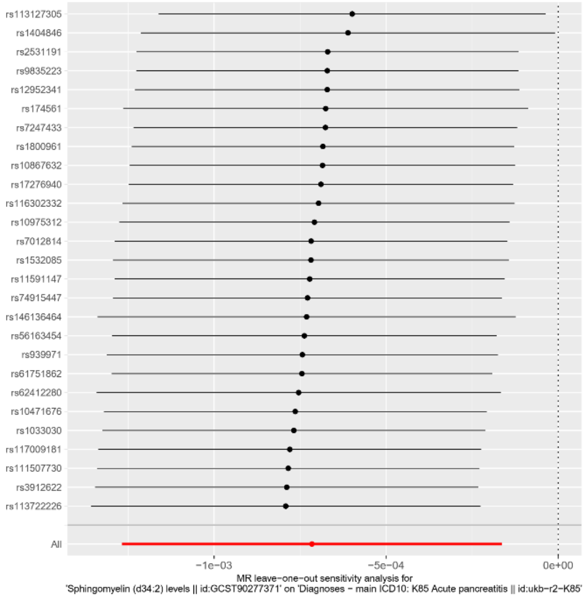

A

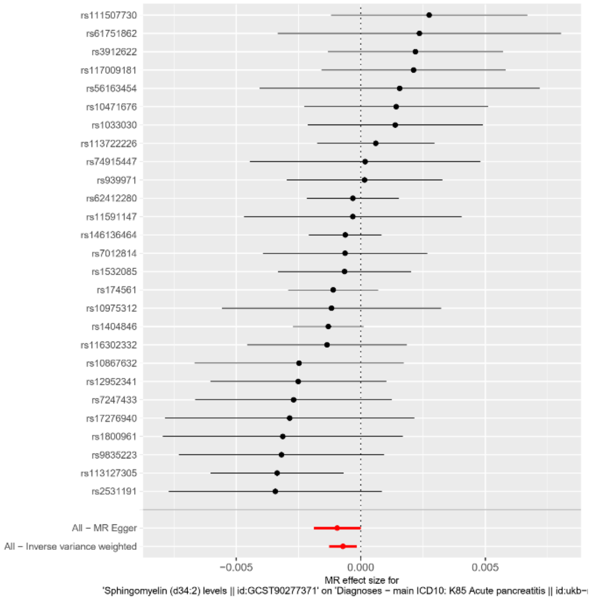

B

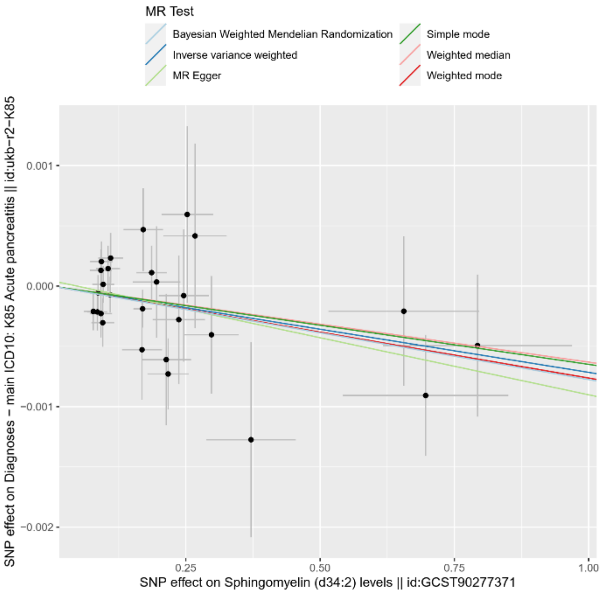

C

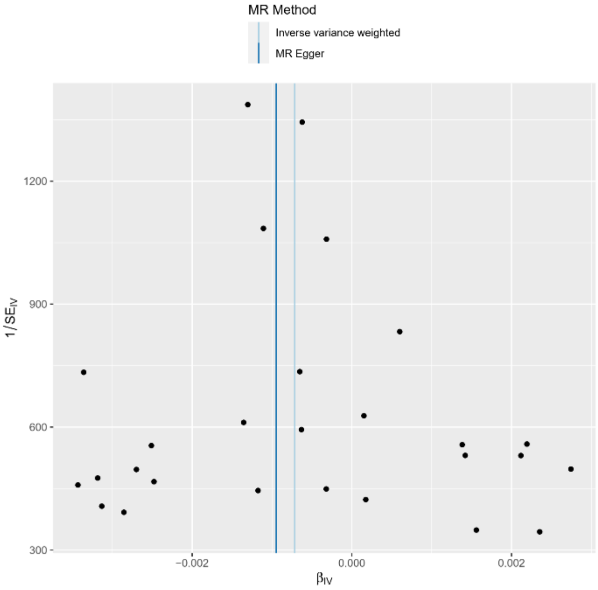

D

Figure S97 Leave-one-out analysis (A), MR effect size (B), scatter plot (C) and funnel plot (D) for Triacylglycerol (48:0) levels on acute pancreatitis for UK Biobank

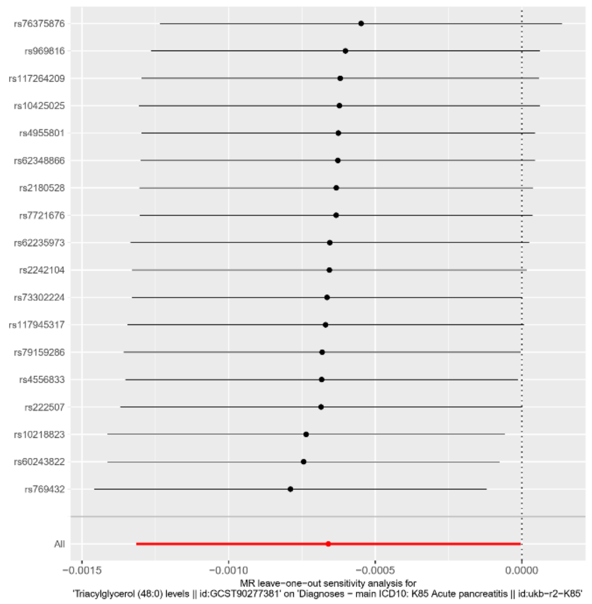

A

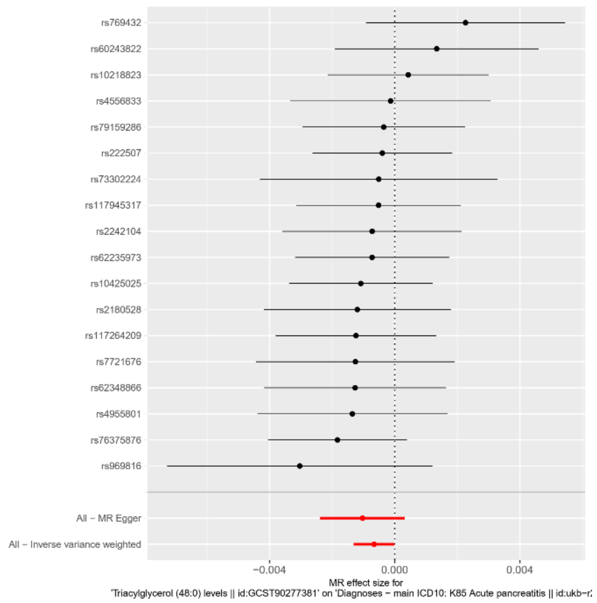

B

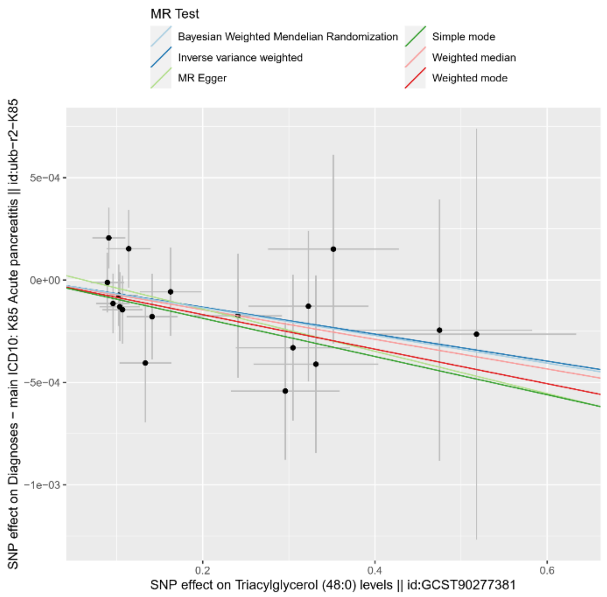

C

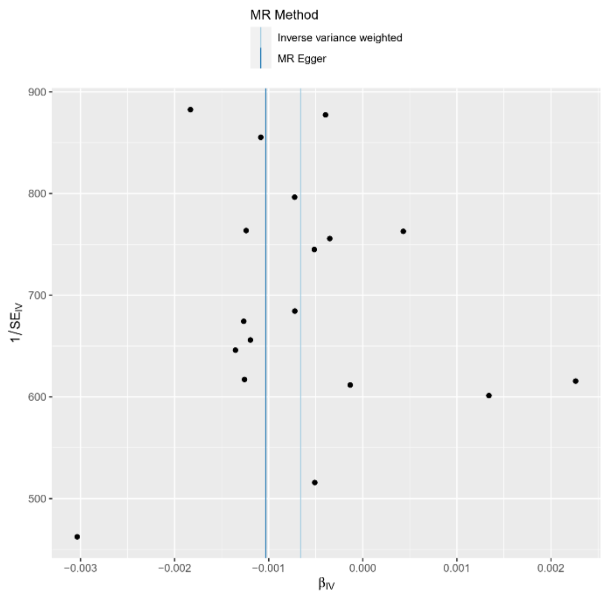

D

Figure S98 Leave-one-out analysis (A), MR effect size (B), scatter plot (C) and funnel plot (D) for Triacylglycerol (50:4) levels on acute pancreatitis for UK Biobank

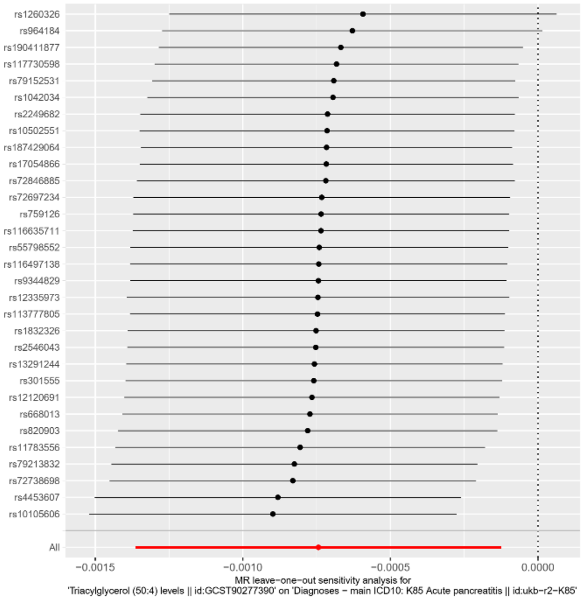

A

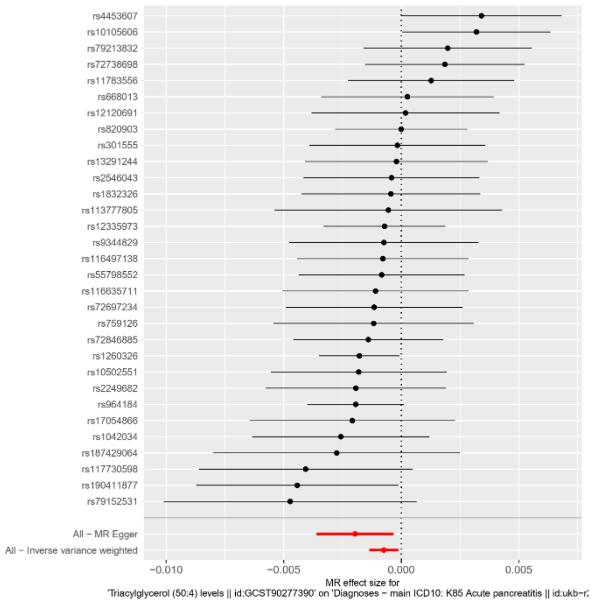

B

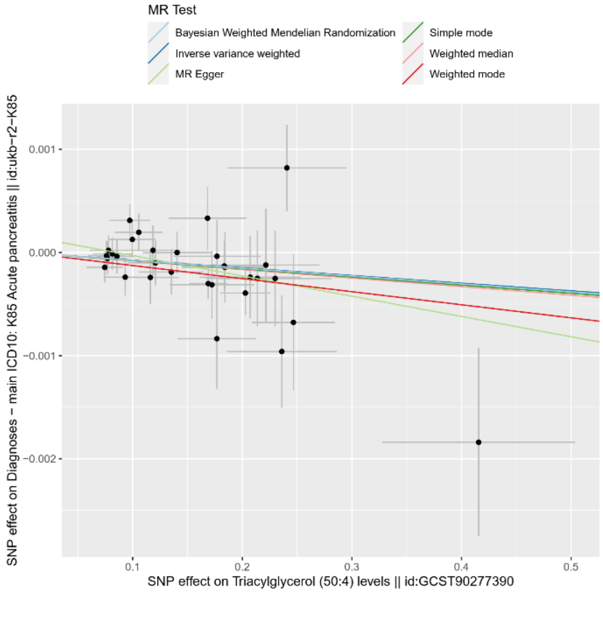

C

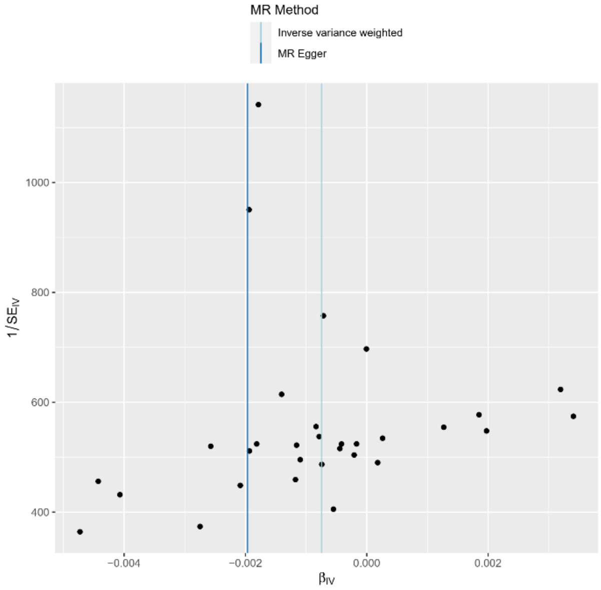

D

Figure S99 Leave-one-out analysis (A), MR effect size (B), scatter plot (C) and funnel plot (D) for Triacylglycerol (51:4) levels on acute pancreatitis for UK Biobank

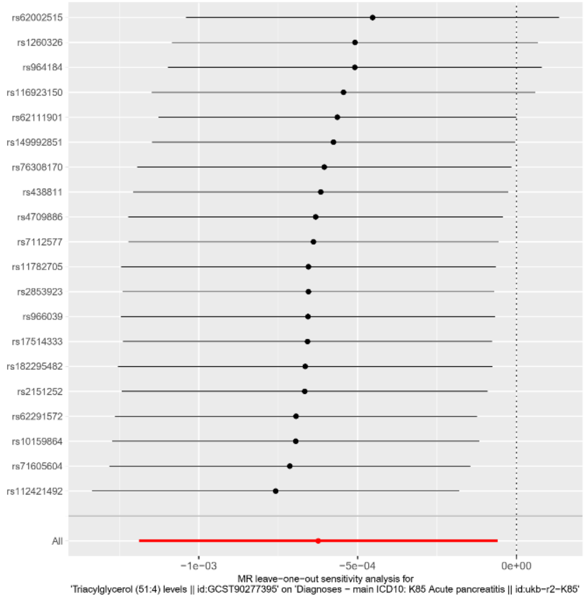

A

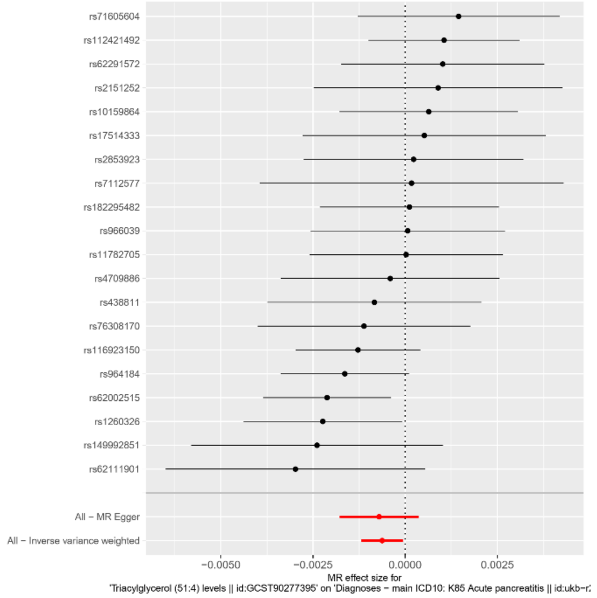

B

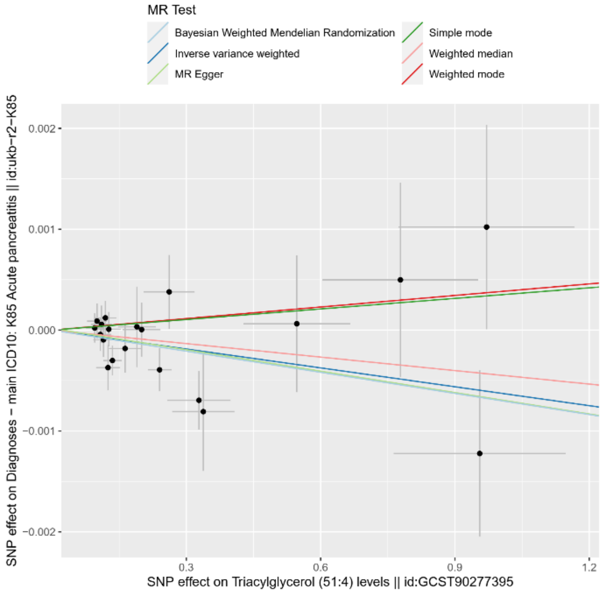

C

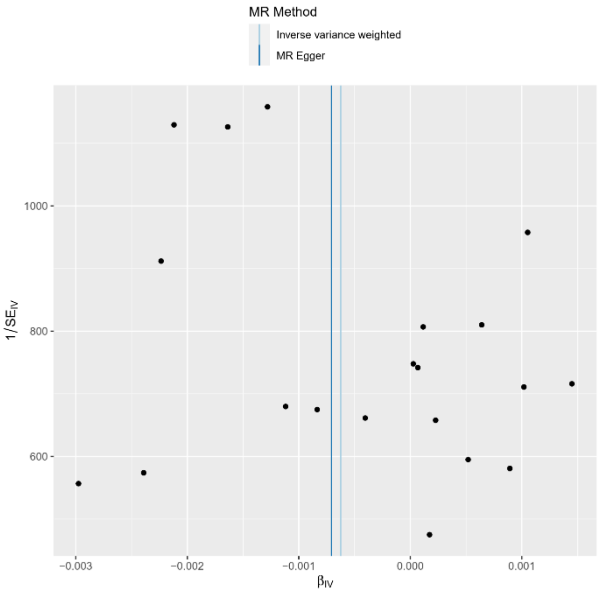

D

Figure S100 Leave-one-out analysis (A), MR effect size (B), scatter plot (C) and funnel plot (D) for Triacylglycerol (52:4) levels on acute pancreatitis for UK Biobank

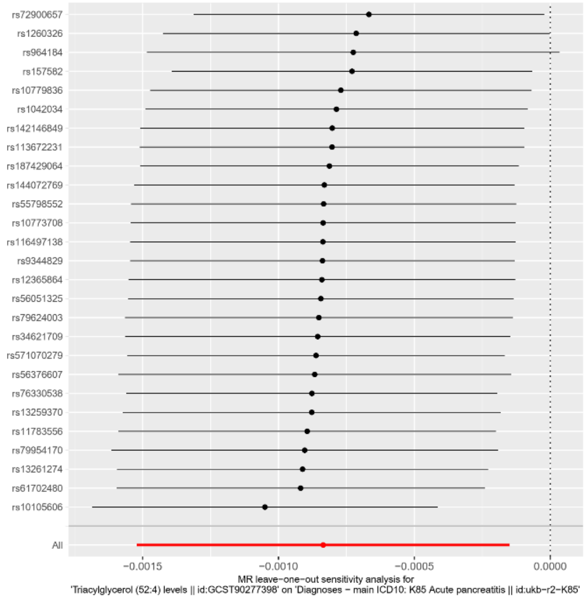

A

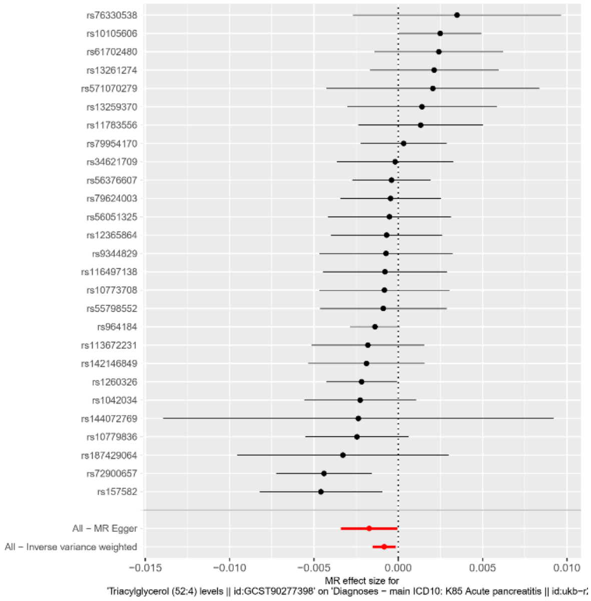

B

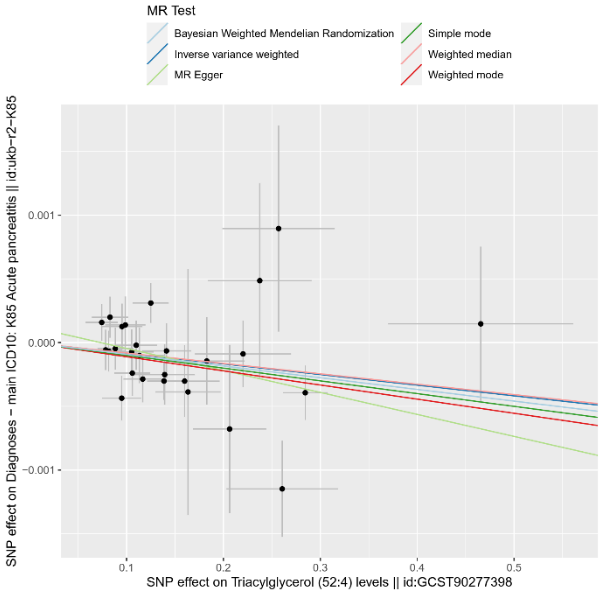

C

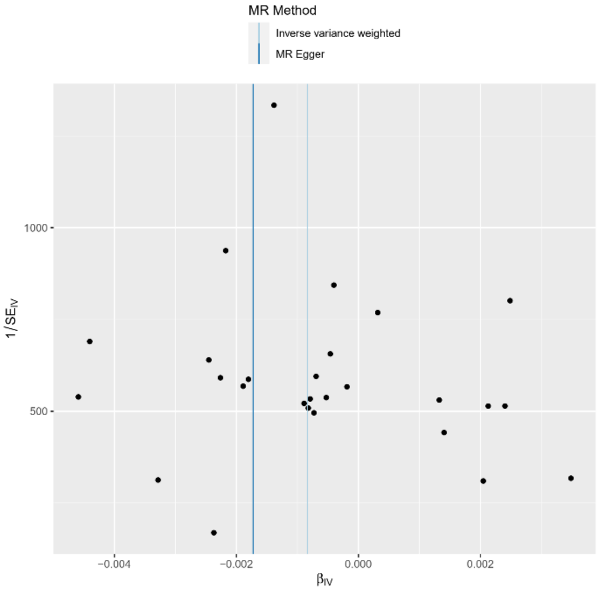

D

Figure S101 Leave-one-out analysis (A), MR effect size (B), scatter plot (C) and funnel plot (D) for Triacylglycerol (52:5) levels on acute pancreatitis for UK Biobank

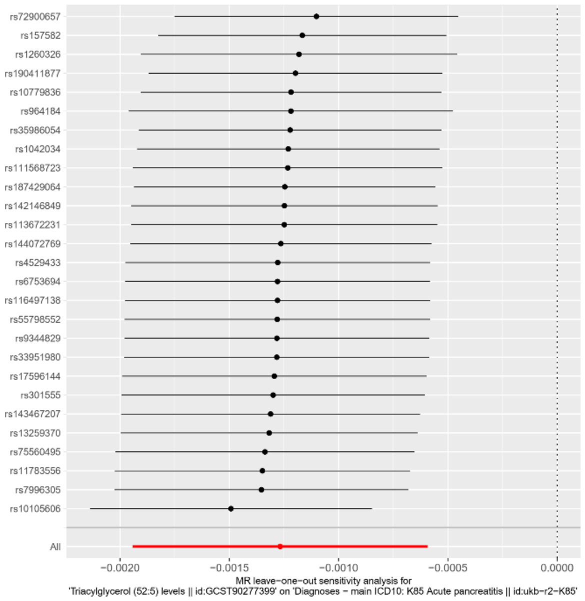

A

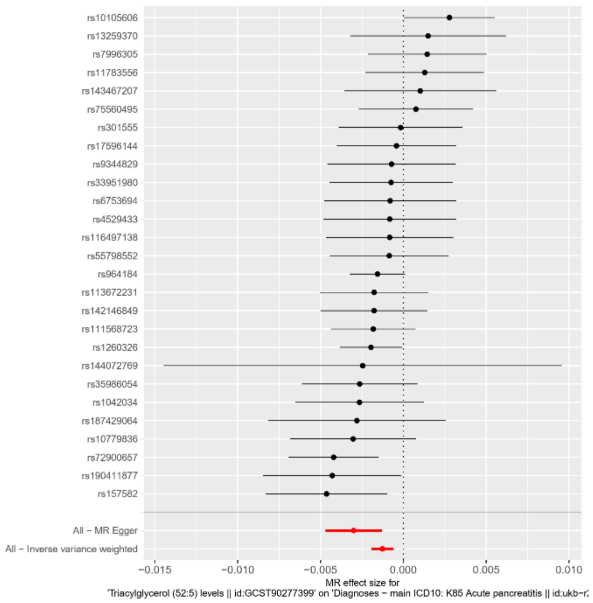

B

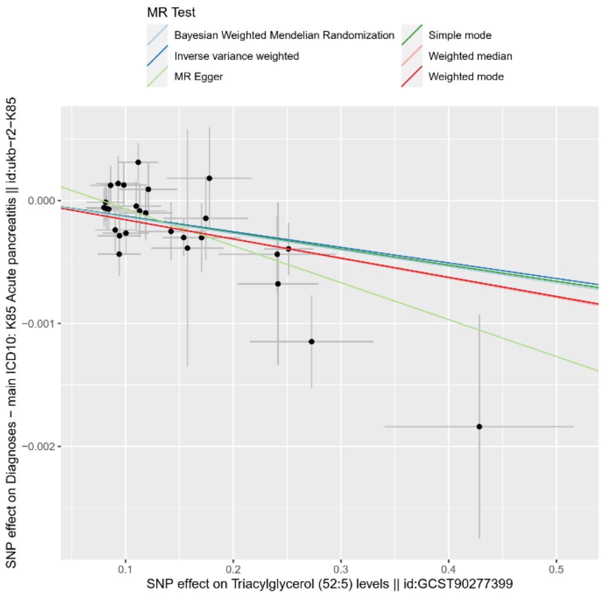

C

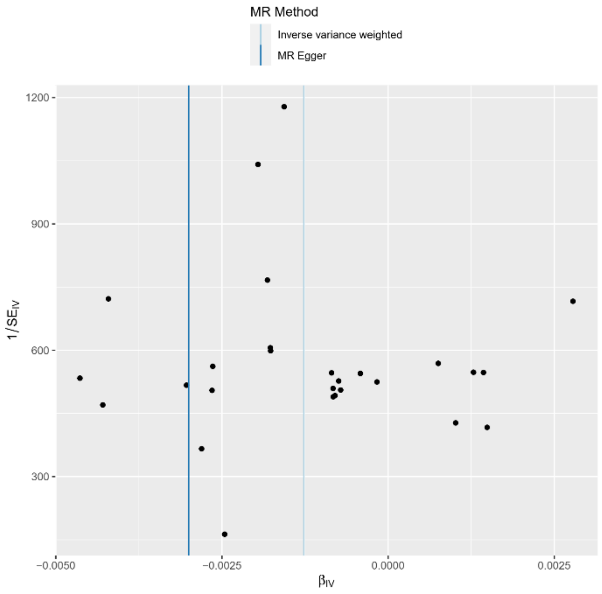

D

Figure S102 Leave-one-out analysis (A), MR effect size (B), scatter plot (C) and funnel plot (D) for Triacylglycerol (52:5) levels on acute pancreatitis after eliminating outliers for UK Biobank

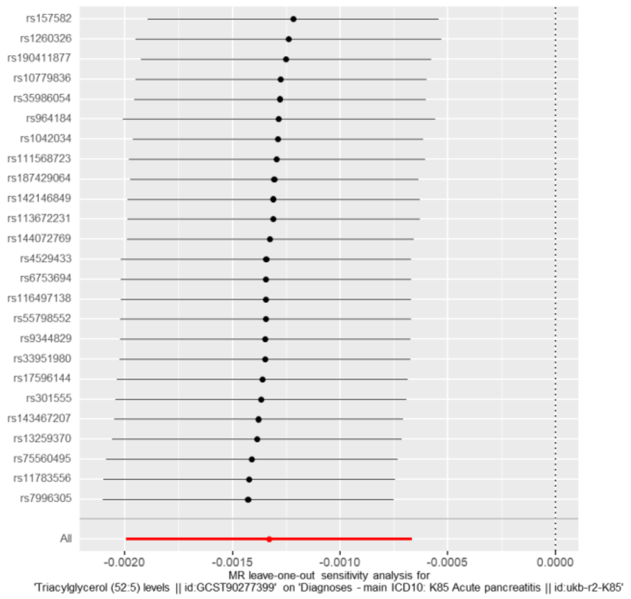

A

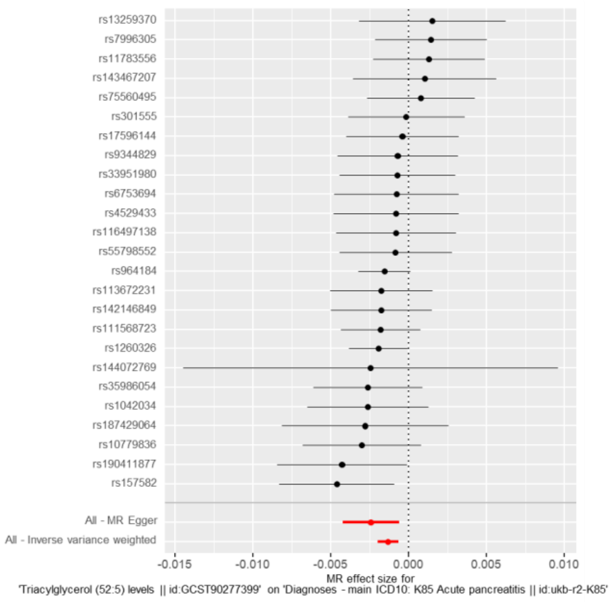

B

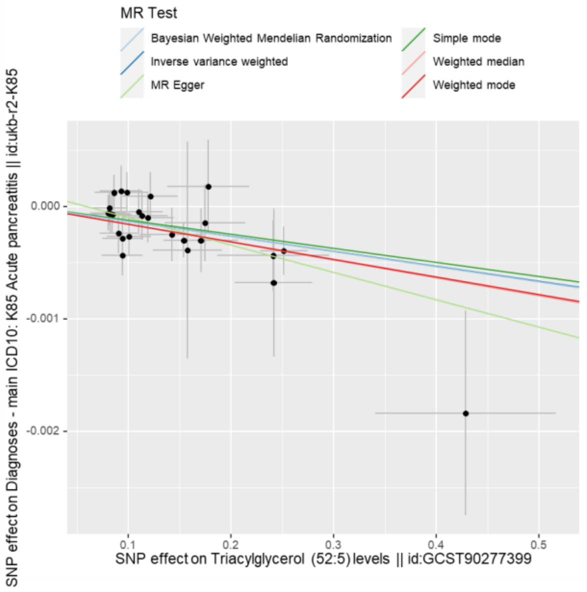

C

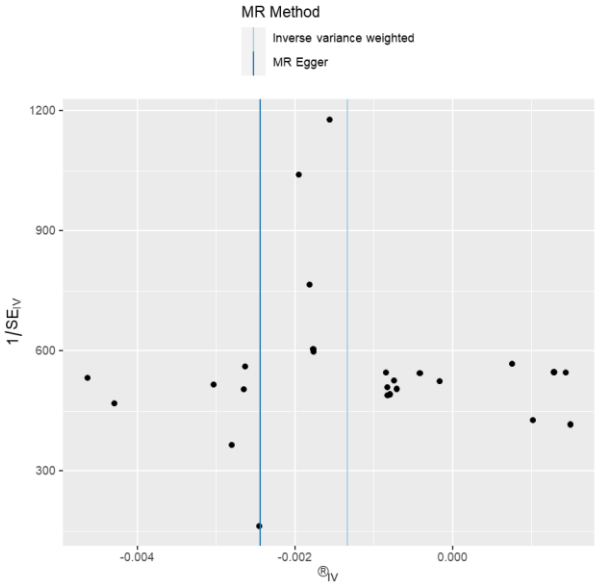

D

Figure S103 Leave-one-out analysis (A), MR effect size (B), scatter plot (C) and funnel plot (D) for Sterol ester (27:1/18:1) levels on chronic pancreatitis for UK Biobank

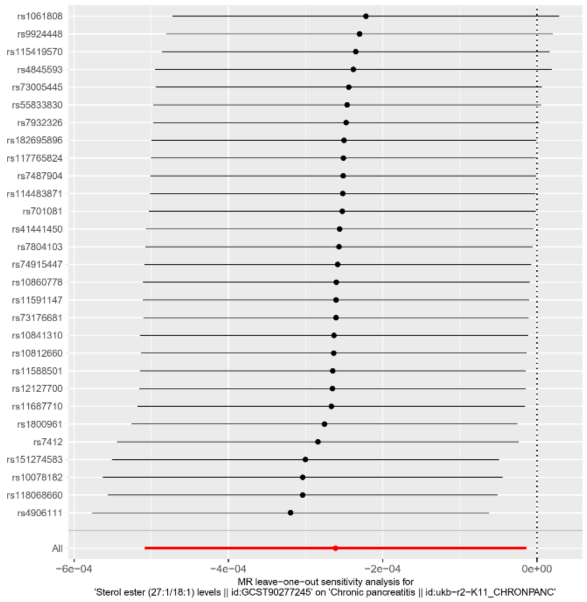

A

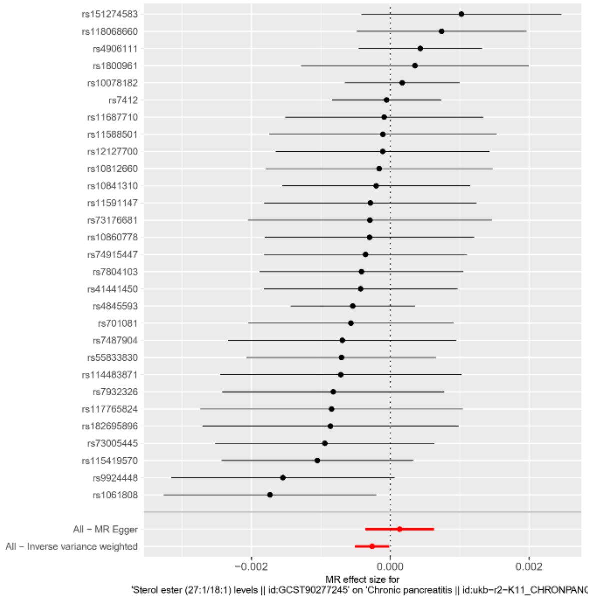

B

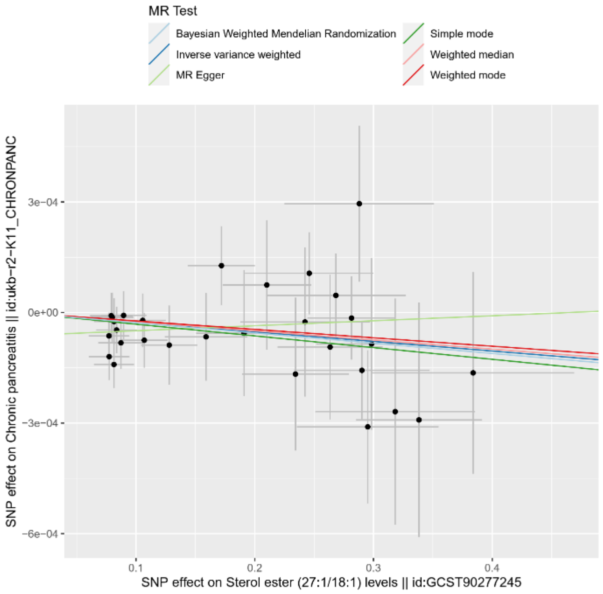

C

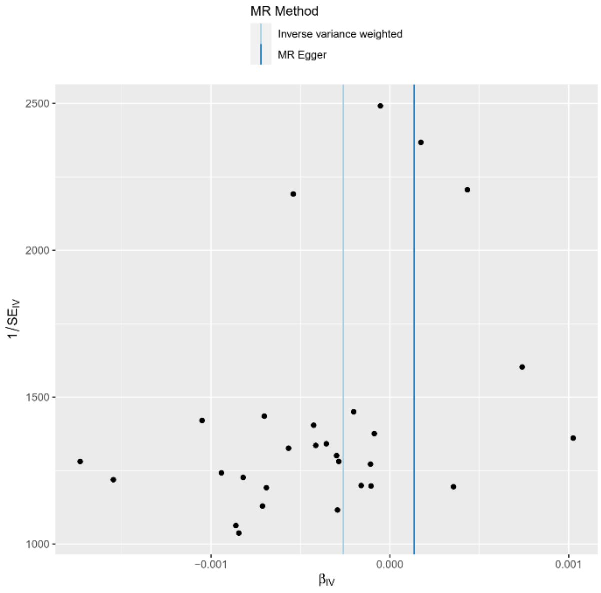

D

Figure S104 Leave-one-out analysis (A), MR effect size (B), scatter plot (C) and funnel plot (D) for Sterol ester (27:1/20:2) levels on chronic pancreatitis for UK Biobank

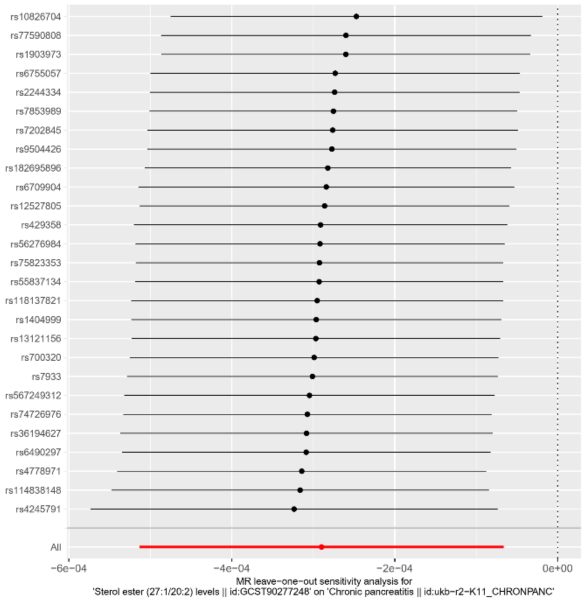

A

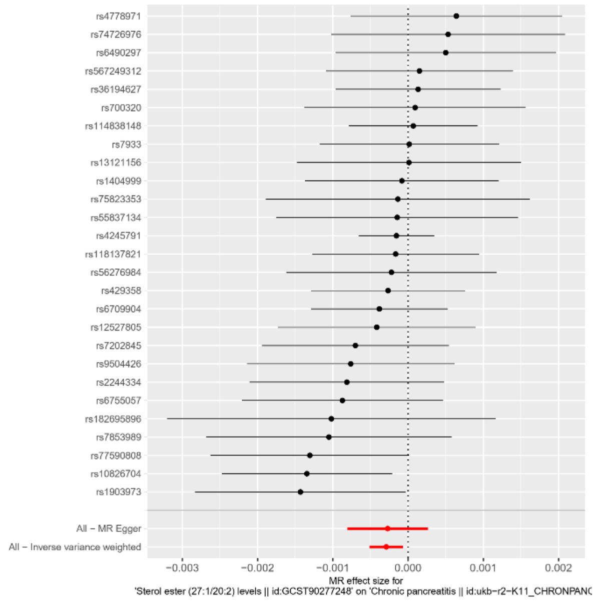

B

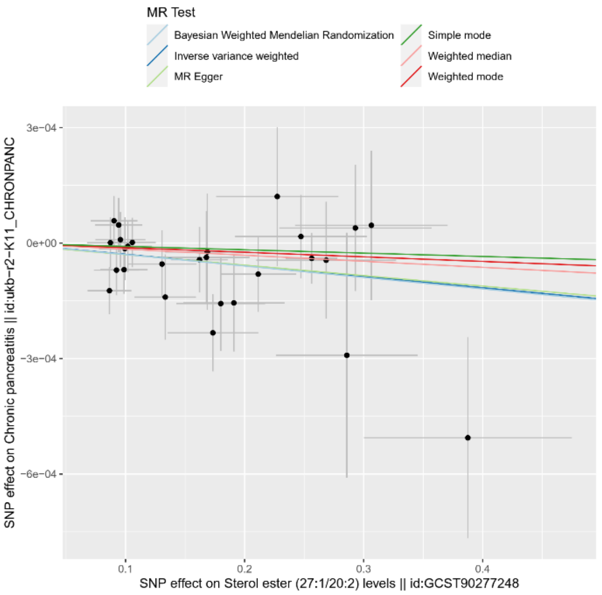

C

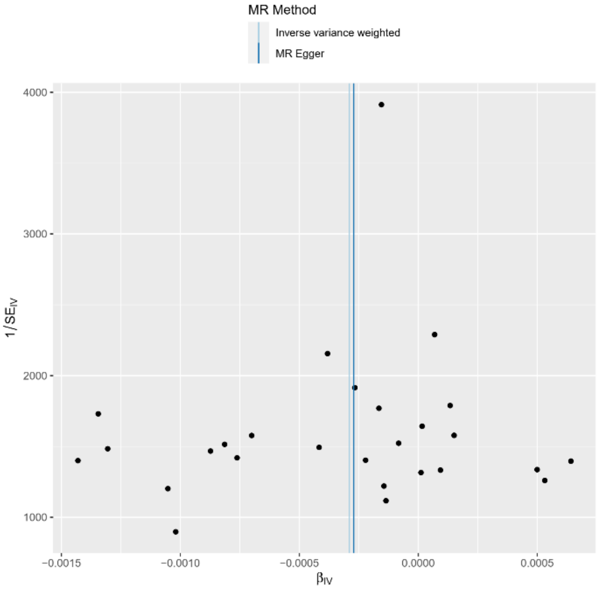

D

Figure S105 Leave-one-out analysis (A), MR effect size (B), scatter plot (C) and funnel plot (D) for Ceramide (d40:2) levels on chronic pancreatitis for UK Biobank

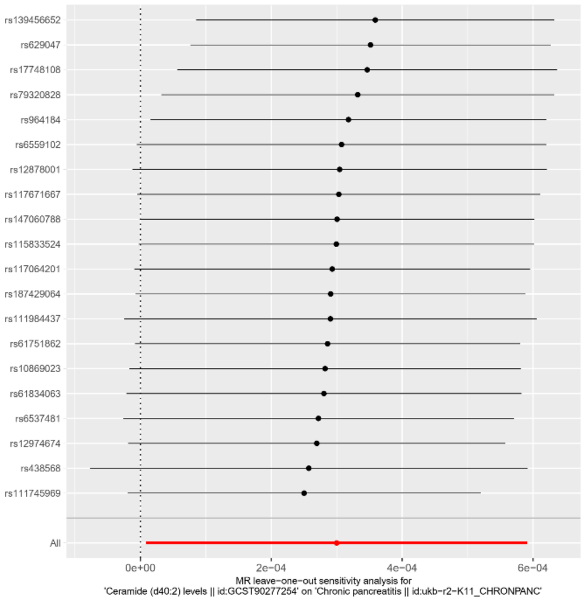

A

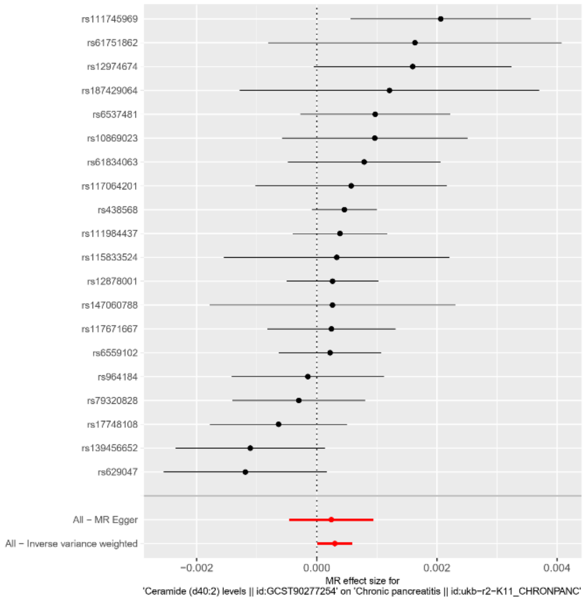

B

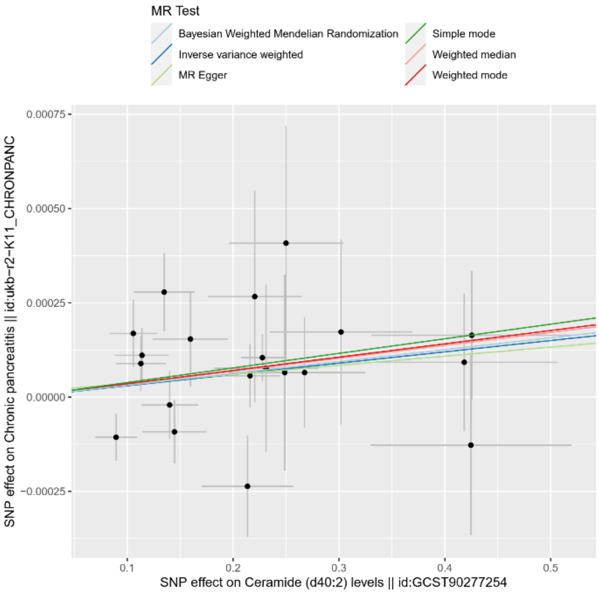

C

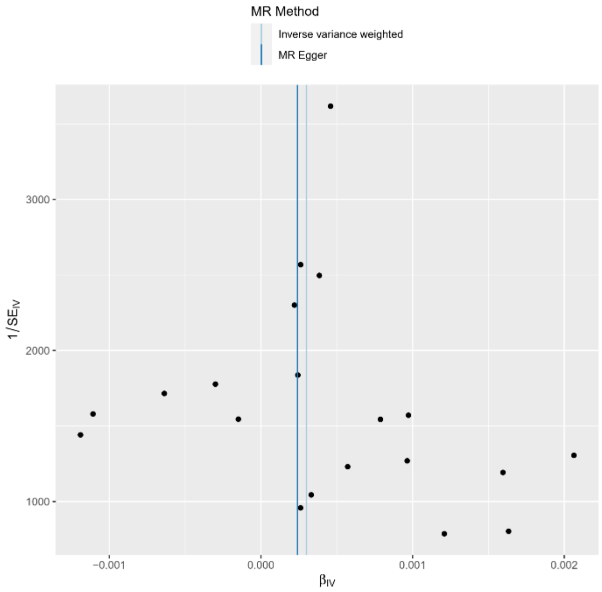

D

Figure S106 Leave-one-out analysis (A), MR effect size (B), scatter plot (C) and funnel plot (D) for Phosphatidylcholine (16:0_20:5) levels on chronic pancreatitis for UK Biobank

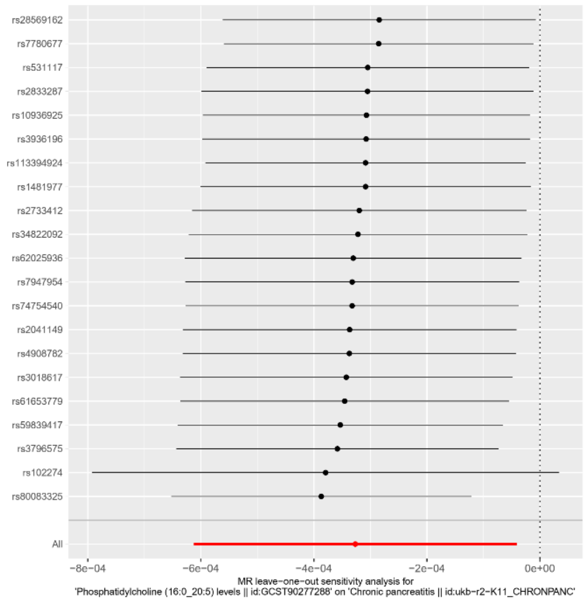

A

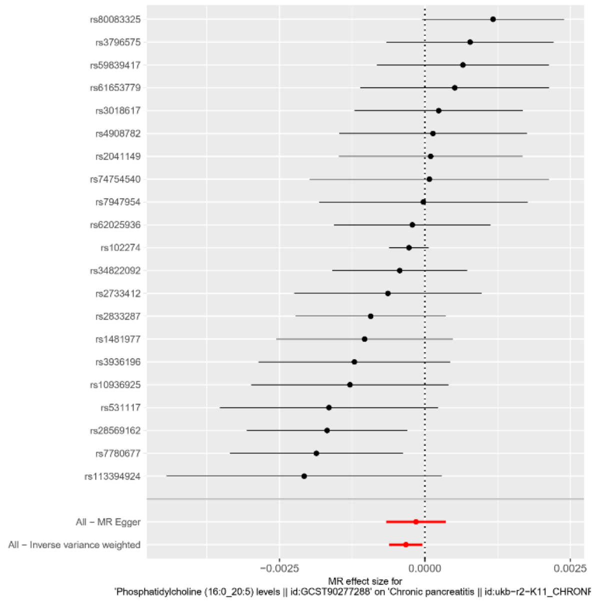

B

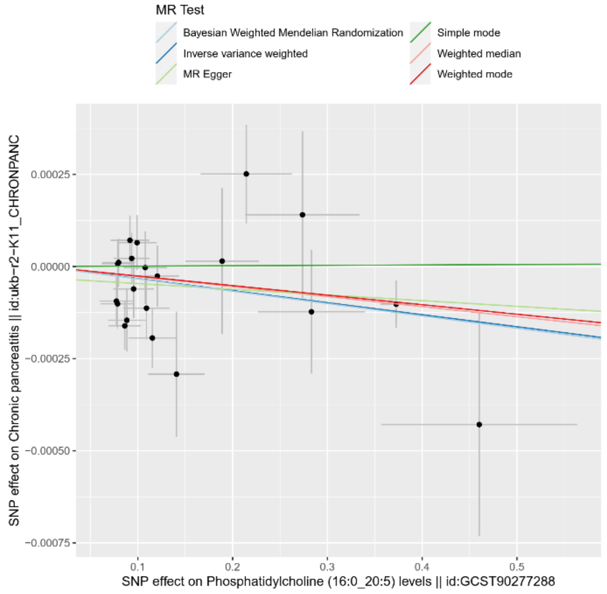

C

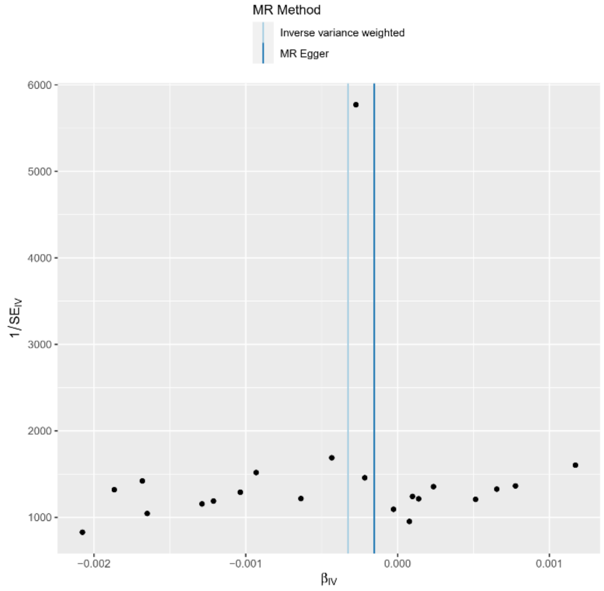

D

Figure S107 Leave-one-out analysis (A), MR effect size (B), scatter plot (C) and funnel plot (D) for Phosphatidylcholine (18:0_18:1) levels on chronic pancreatitis for UK Biobank

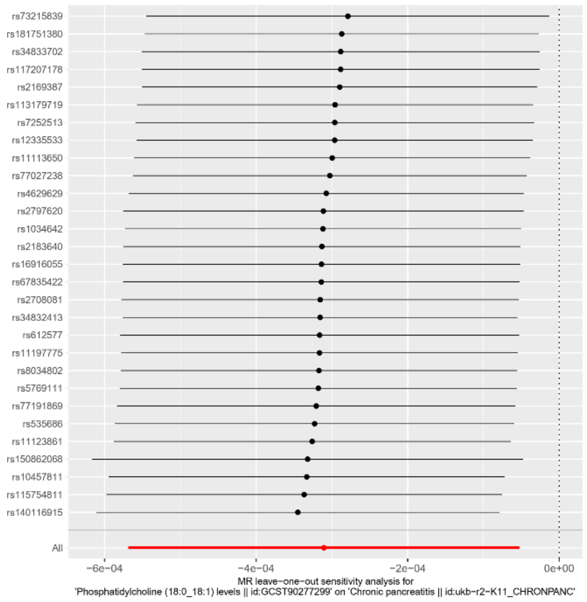

A

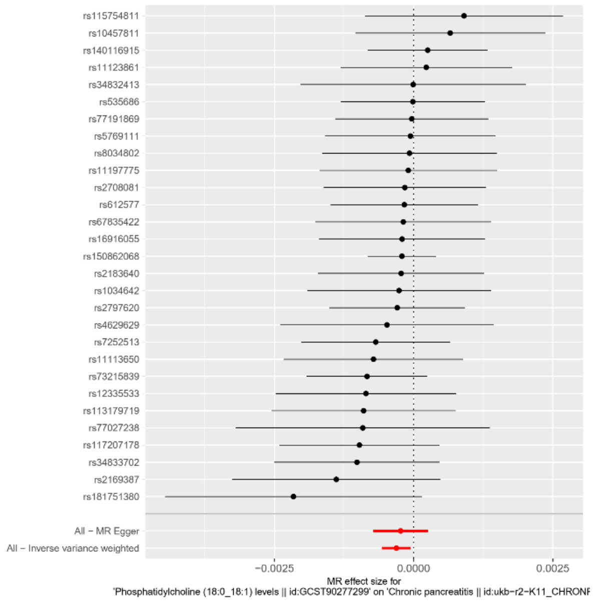

B

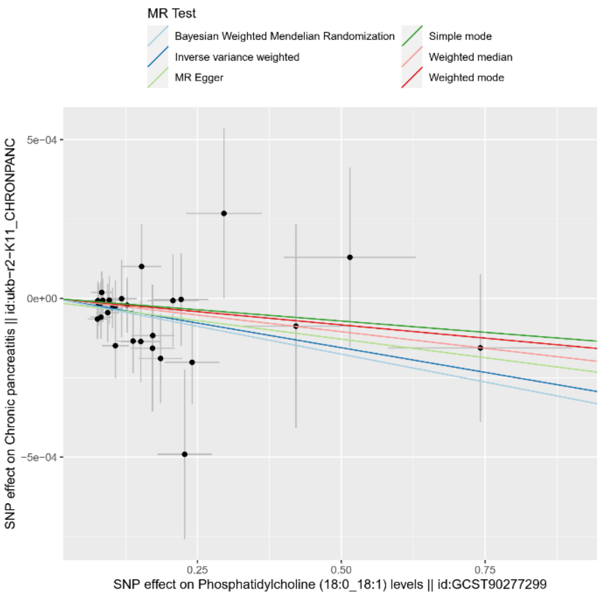

C

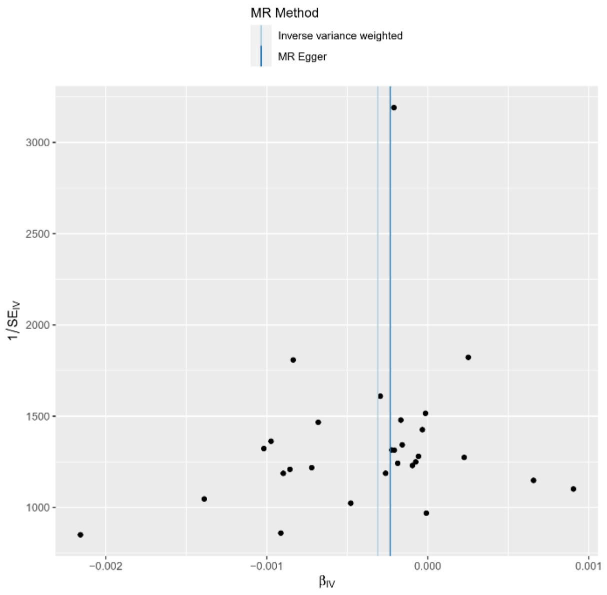

D

Figure S108 Leave-one-out analysis (A), MR effect size (B), scatter plot (C) and funnel plot (D) for Phosphatidylcholine (O-18:1_20:4) levels on chronic pancreatitis for UK Biobank

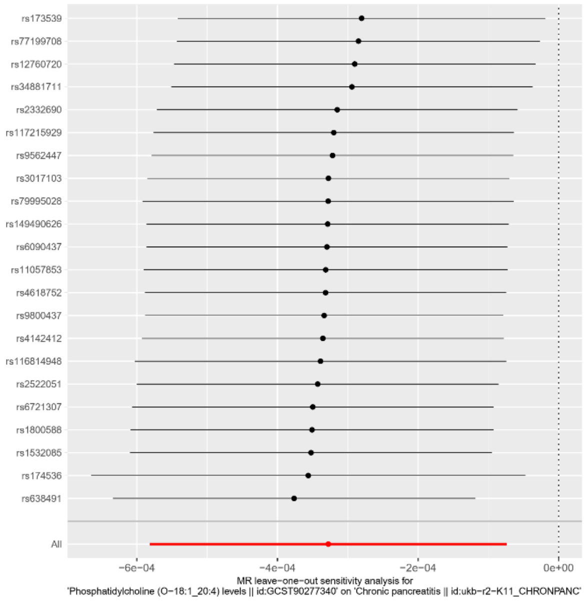

A

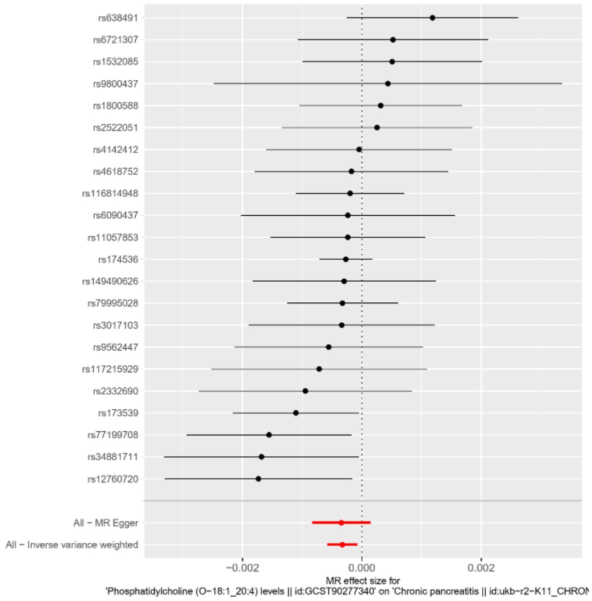

B

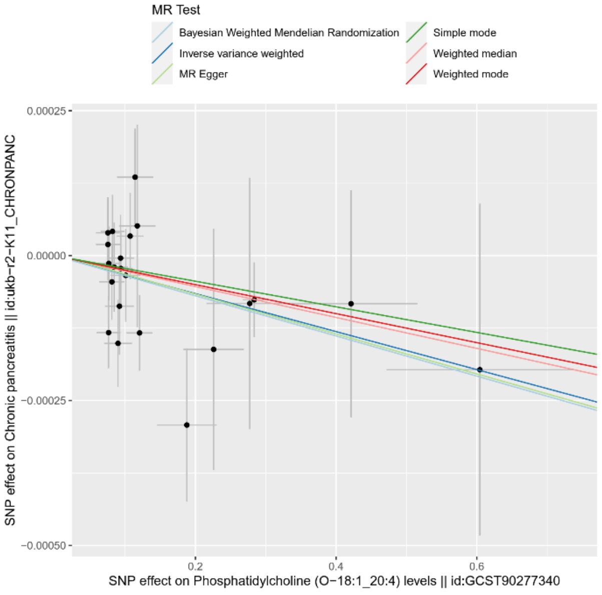

C

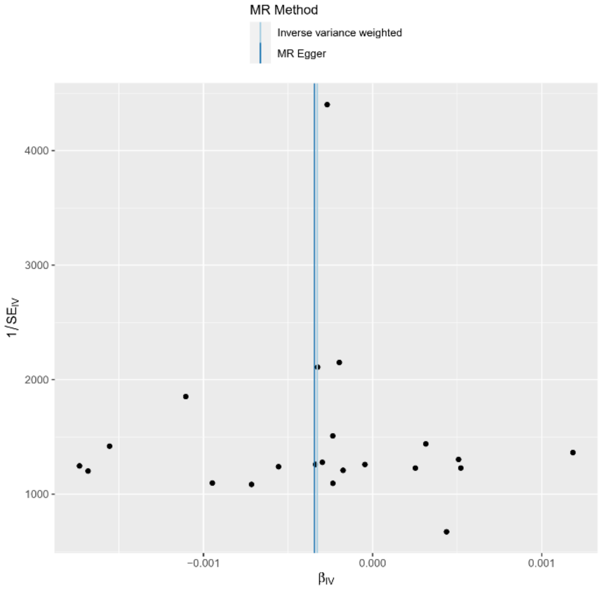

D

Figure S109 Leave-one-out analysis (A), MR effect size (B), scatter plot (C) and funnel plot (D) for Phosphatidylcholine (O-18:2_18:1) levels on chronic pancreatitis for UK Biobank

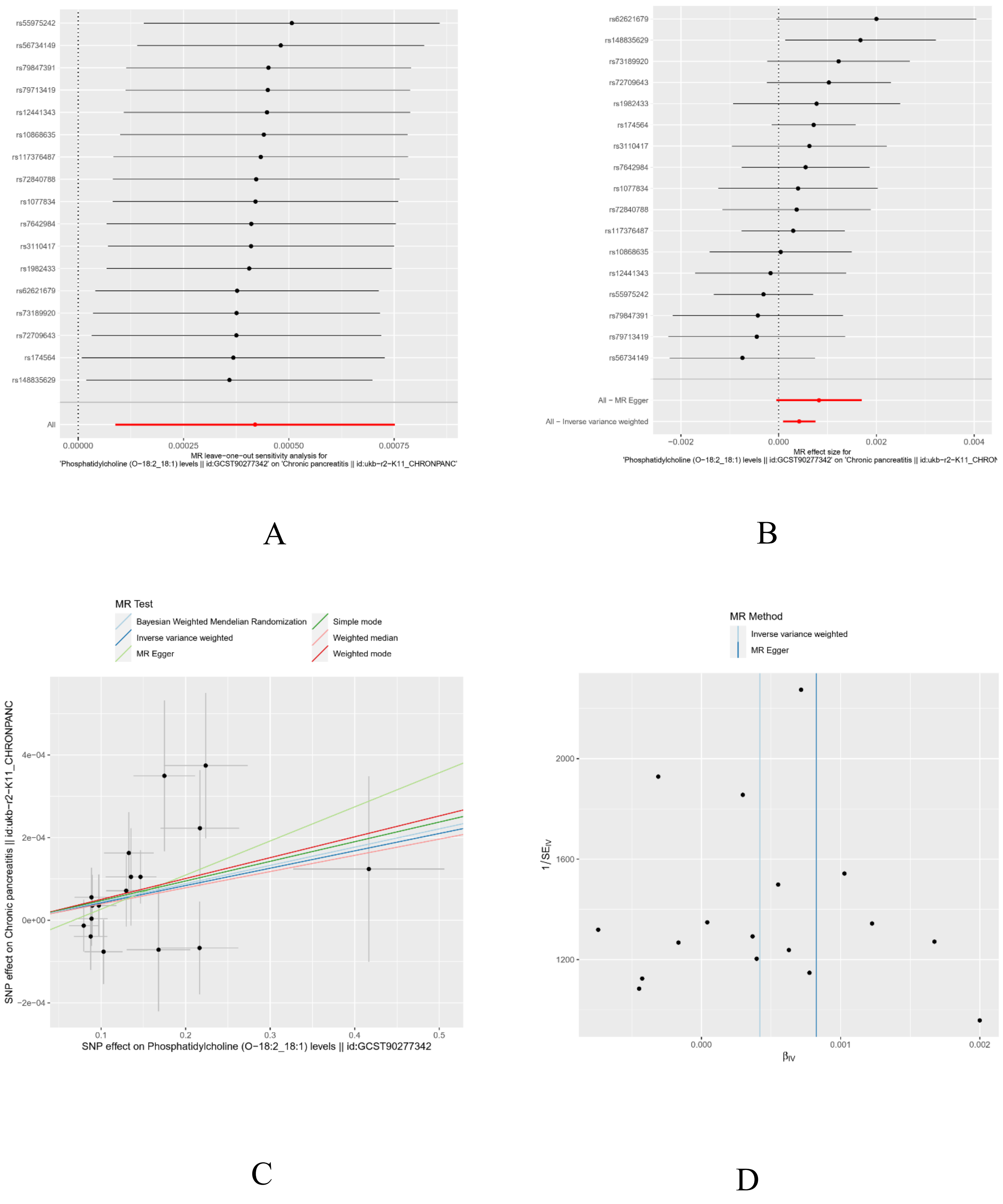

Figure S110 Leave-one-out analysis (A), MR effect size (B), scatter plot (C) and funnel plot (D) for Phosphatidylinositol (18:0_18:2) levels on chronic pancreatitis for UK Biobank

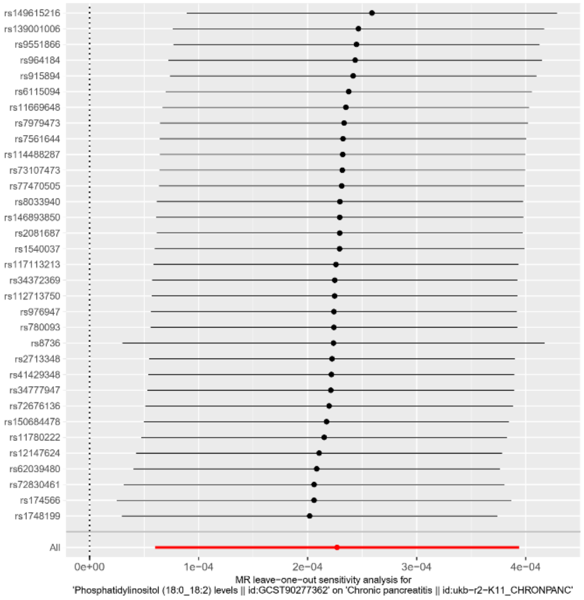

A

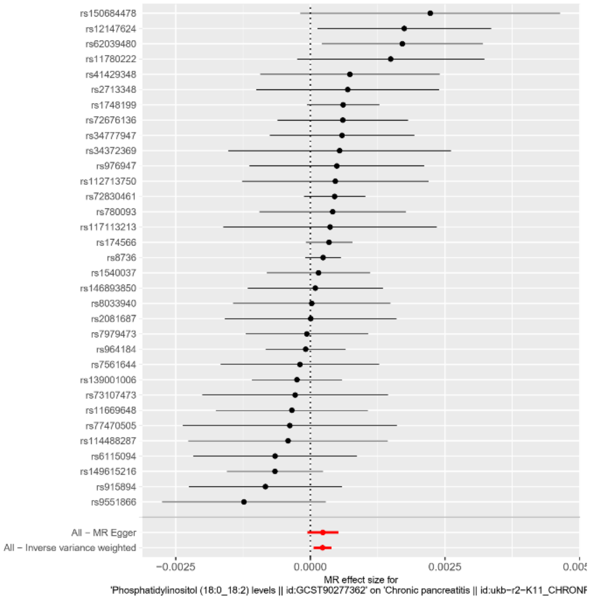

B

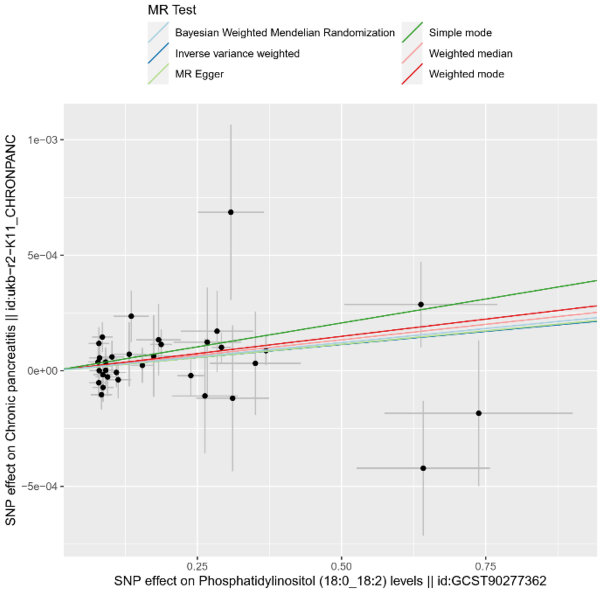

C

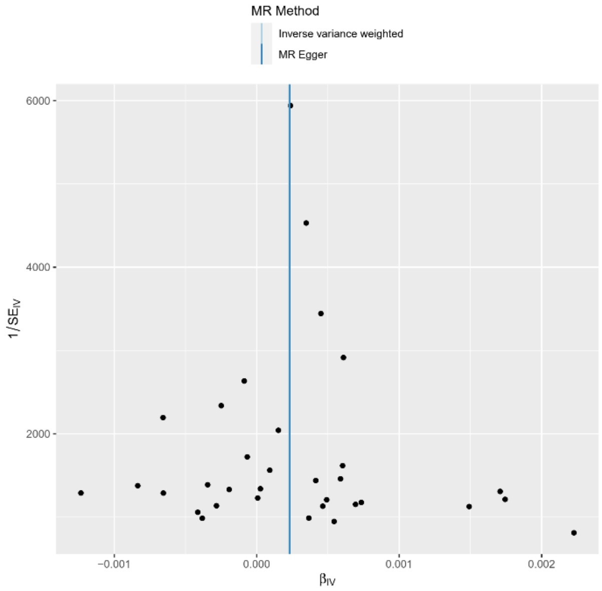

D

Figure S111 Leave-one-out analysis (A), MR effect size (B), scatter plot (C) and funnel plot (D) for Sphingomyelin (d38:1) levels on chronic pancreatitis for UK Biobank

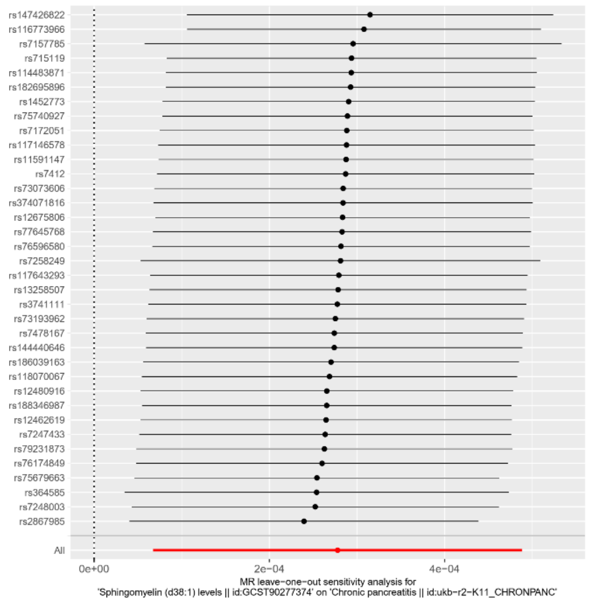

A

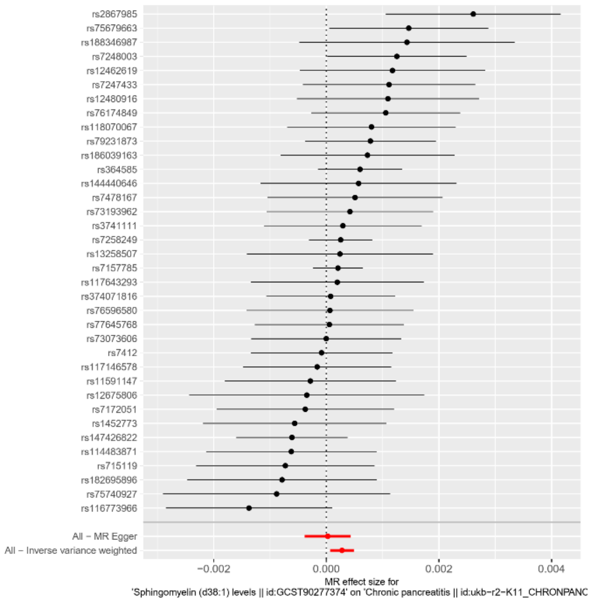

B

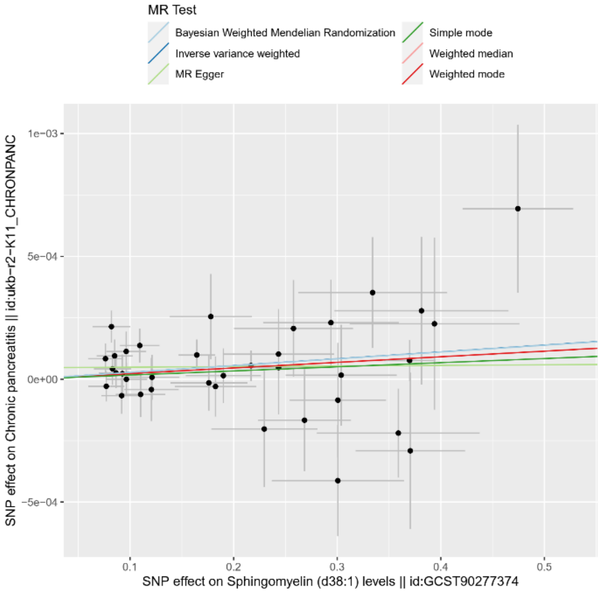

C

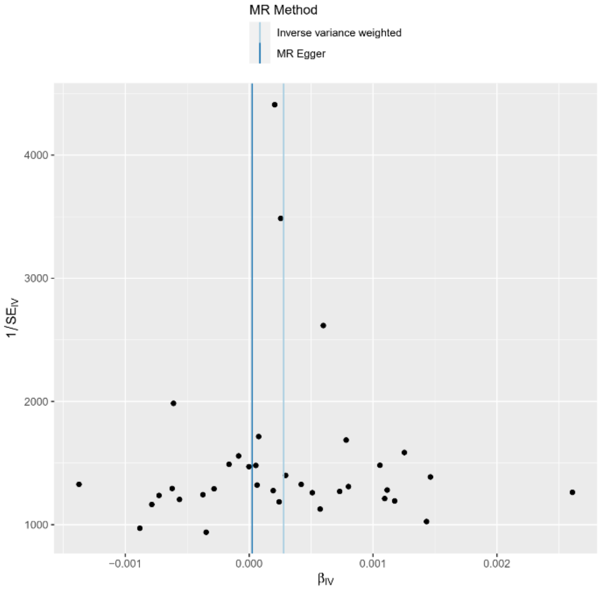

D

Figure S112 Leave-one-out analysis (A), MR effect size (B), scatter plot (C) and funnel plot (D) for Sphingomyelin (d40:2) levels on chronic pancreatitis for UK Biobank

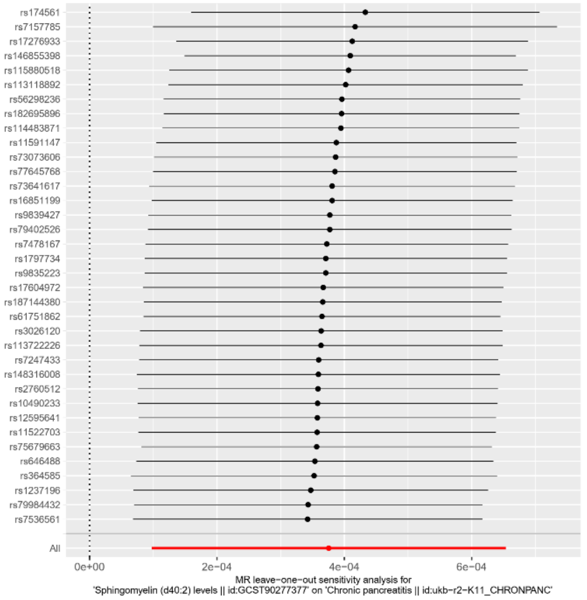

A

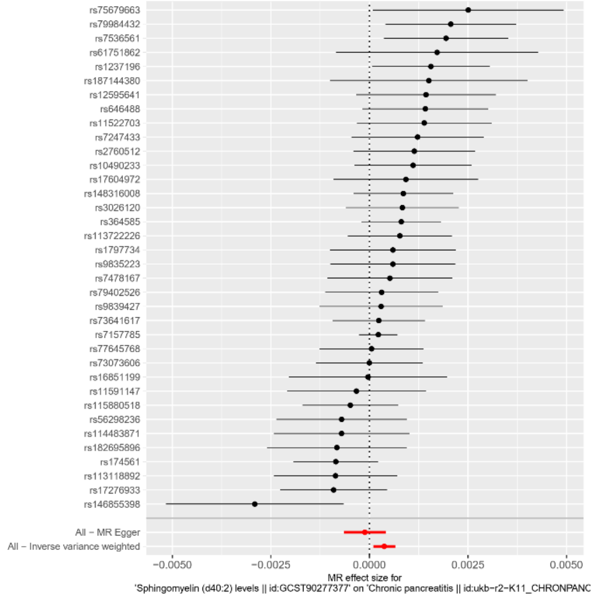

B

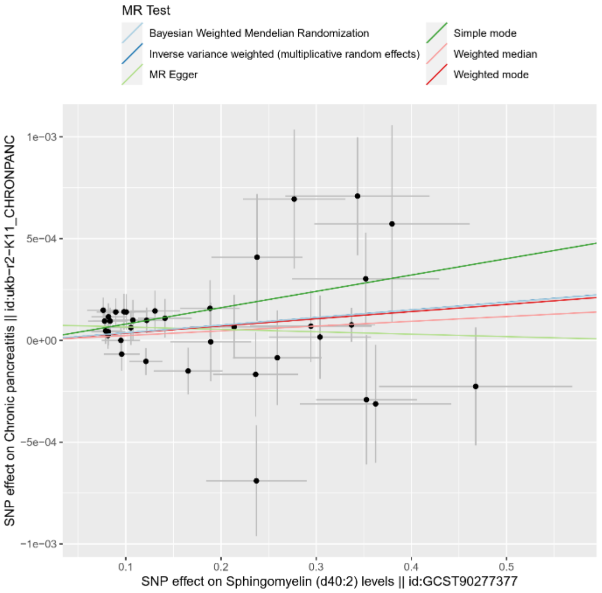

C

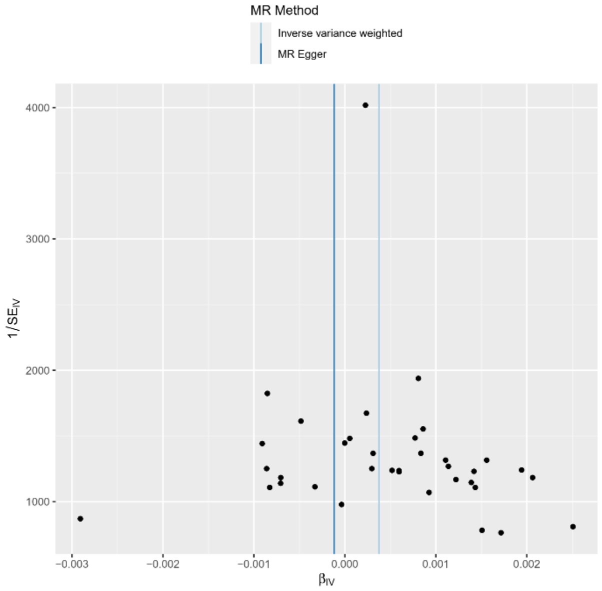

D

Figure S113 Leave-one-out analysis (A), MR effect size (B), scatter plot (C) and funnel plot (D) for Sphingomyelin (d40:2) levels on chronic pancreatitis after eliminating outliers for UK Biobank

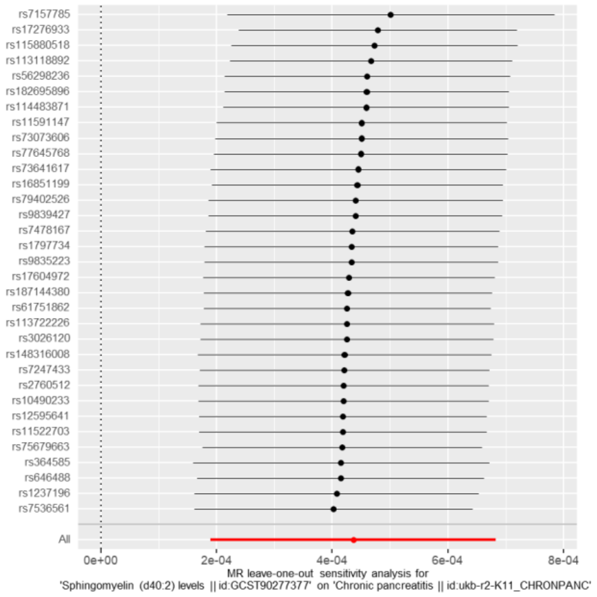

A

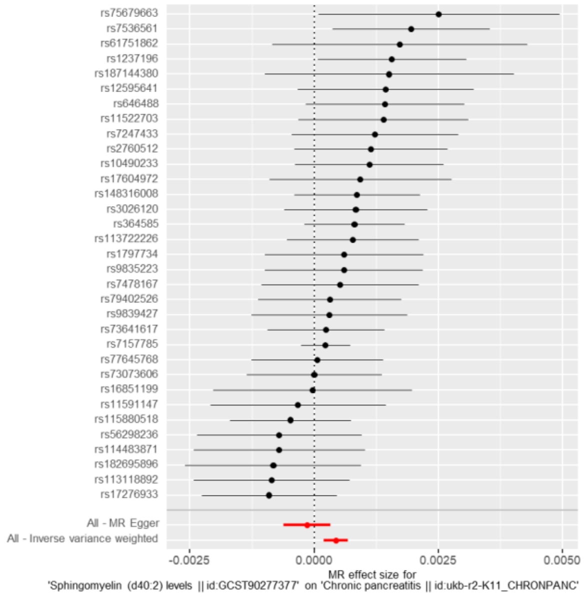

B

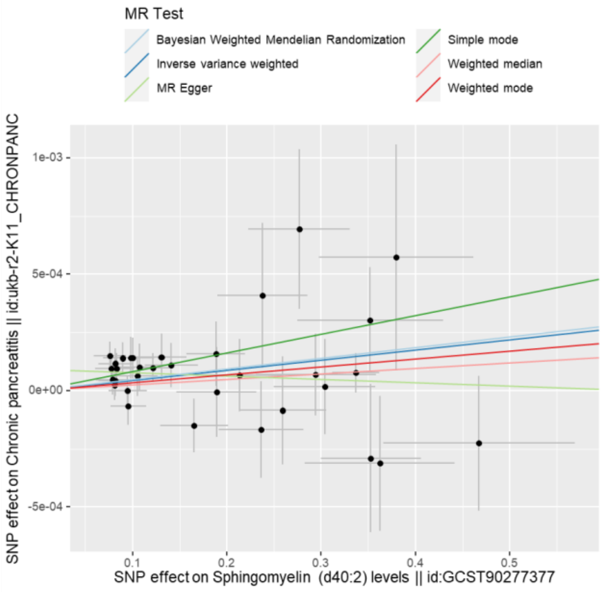

C

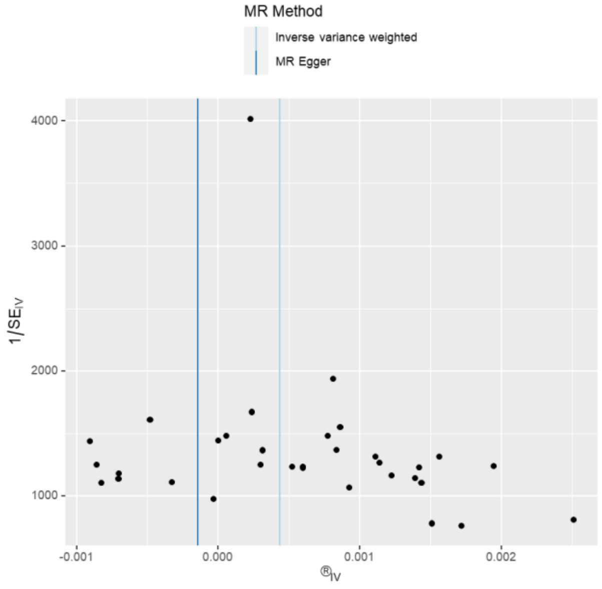

D
